# Comparative effectiveness and risk of preterm birth of local treatments for cervical intraepithelial neoplasia and stage IA1 cervical cancer: a systematic review and network meta-analysis

**DOI:** 10.1016/S1470-2045(22)00334-5

**Published:** 2022-08

**Authors:** Antonios Athanasiou, Areti Angeliki Veroniki, Orestis Efthimiou, Ilkka Kalliala, Huseyin Naci, Sarah Bowden, Maria Paraskevaidi, Marc Arbyn, Deirdre Lyons, Pierre Martin-Hirsch, Phillip Bennett, Evangelos Paraskevaidis, Georgia Salanti, Maria Kyrgiou

**Affiliations:** a Institute of Reproductive and Developmental Biology (IRDB), Department of Metabolism, Digestion and Reproduction—Surgery and Cancer, Faculty of Medicine, Imperial College London, London, UK; b Imperial College Healthcare NHS Trust, London, UK; c Knowledge Translation Program, Li Ka Shing Knowledge Institute, St Michael's Hospital, Toronto, ON, Canada; d Institute for Health Policy, Management and Evaluation, University of Toronto, Toronto, ON, Canada; e Institute of Social and Preventive Medicine, University of Bern, Bern, Switzerland; f Institute of Primary Health Care, University of Bern, Bern, Switzerland; g Department of Obstetrics and Gynaecology, University of Helsinki and Helsinki University Hospital, Helsinki, Finland; h Department of Health Policy, London School of Economics and Political Science, London, UK; i Unit of Cancer Epidemiology, Belgian Cancer Centre, Scientific Institute of Public Health, Brussels, Belgium; j Department of Gynaecologic Oncology, Lancashire Teaching Hospitals NHS Foundation Trust, Preston, UK; k Department of Obstetrics and Gynaecology, University of Ioannina and University Hospital of Ioannina, Ioannina, Greece

## Abstract

**Background:**

The trade-off between comparative effectiveness and reproductive morbidity of different treatment methods for cervical intraepithelial neoplasia (CIN) remains unclear. We aimed to determine the risks of treatment failure and preterm birth associated with various treatment techniques.

**Methods:**

In this systematic review and network meta-analysis, we searched MEDLINE, Embase, and the Cochrane Central Register of Controlled Trials database for randomised and non-randomised studies reporting on oncological or reproductive outcomes after CIN treatments from database inception until March 9, 2022, without language restrictions. We included studies of women with CIN, glandular intraepithelial neoplasia, or stage IA1 cervical cancer treated with excision (cold knife conisation [CKC], laser conisation, and large loop excision of the transformation zone [LLETZ]) or ablation (radical diathermy, laser ablation, cold coagulation, and cryotherapy). We excluded women treated with hysterectomy. The primary outcomes were any treatment failure (defined as any abnormal histology or cytology) and preterm birth (<37 weeks of gestation). The network for preterm birth also included women with untreated CIN (untreated colposcopy group). The main reference group was LLETZ for treatment failure and the untreated colposcopy group for preterm birth. For randomised controlled trials, we extracted group-level summary data, and for observational studies, we extracted relative treatment effect estimates adjusted for potential confounders, when available, and we did random-effects network meta-analyses to obtain odds ratios (ORs) with 95% CIs. We assessed within-study and across-study risk of bias using Cochrane tools. This systematic review is registered with PROSPERO, CRD42018115495 and CRD42018115508.

**Findings:**

7880 potential citations were identified for the outcome of treatment failure and 4107 for the outcome of preterm birth. After screening and removal of duplicates, the network for treatment failure included 19 240 participants across 71 studies (25 randomised) and the network for preterm birth included 68 817 participants across 29 studies (two randomised). Compared with LLETZ, risk of treatment failure was reduced for other excisional methods (laser conisation: OR 0·59 [95% CI 0·44–0·79] and CKC: 0·63 [0·50–0·81]) and increased for laser ablation (1·69 [1·27–2·24]) and cryotherapy (1·84 [1·33–2·56]). No differences were found for the comparison of cold coagulation versus LLETZ (1·09 [0·68–1·74]) but direct data were based on two small studies only. Compared with the untreated colposcopy group, risk of preterm birth was increased for all excisional techniques (CKC: 2·27 [1·70–3·02]; laser conisation: 1·77 [1·29–2·43]; and LLETZ: 1·37 [1·16–1·62]), whereas no differences were found for ablative methods (laser ablation: 1·05 [0·78–1·41]; cryotherapy: 1·01 [0·35–2·92]; and cold coagulation: 0·67 [0·02–29·15]). The evidence was based mostly on observational studies with their inherent risks of bias, and the credibility of many comparisons was low.

**Interpretation:**

More radical excisional techniques reduce the risk of treatment failure but increase the risk of subsequent preterm birth. Although there is uncertainty, ablative treatments probably do not increase risk of preterm birth, but are associated with higher failure rates than excisional techniques. Although we found LLETZ to have balanced effectiveness and reproductive morbidity, treatment choice should rely on a woman's age, size and location of lesion, and future family planning.

**Funding:**

National Institute for Health and Care Research: Research for Patient Benefit.

## Introduction

The introduction of systematic call and recall screening programmes in the UK resulted in a substantial decrease in the incidence of and mortality due to cervical cancer because preinvasive lesions (cervical intraepithelial neoplasia [CIN]) can be detected and treated.^1^ Although widespread human papillomavirus (HPV) vaccination led to a further reduction in rates of cervical cancer,^2^, ^3^ HPV-related disease burden remains high due to low coverage and a large reservoir of unvaccinated women. In England alone, more than 33 000 high-grade abnormalities are detected every year.^4^

**Research in context**

**Evidence before this study**

Local treatment techniques for cervical preinvasive and early invasive disease were previously thought to be interchangeable with regards to treatment failure rates, largely based on a 2013 Cochrane review of randomised controlled trials. However, this review was underpowered to detect differences among highly efficacious treatments and has been questioned by later evidence. The risk of prematurity in subsequent pregnancies has been known to increase with increasing treatment radicality and length of cone excised. The trade-off between treatment failure rates and preterm birth among different treatment methods remains unclear to date. We searched PubMed, with no language restrictions, for systematic reviews from database inception until March 9, 2022, using the terms “treatment” AND “cervical intraepithelial neoplasia”. We identified meta-analyses reporting on oncological or reproductive outcomes after cervical intraepithelial neoplasia (CIN) treatments, but none of these explored the trade-off between effectiveness and risk of prematurity, and no network meta-analyses have been published to date.

**Added value of this study**

To our knowledge, this is the first network meta-analysis to explore the comparative effectiveness and reproductive morbidity of different treatment methods and cone lengths, and to estimate absolute risks. We found that more aggressive treatments (ie, cold knife conisation and laser conisation) are associated with lower failure rates but a higher risk of subsequent preterm birth than more conservative treatments (ie, large loop excision of the transformation zone [LLETZ] and ablation). Conversely, ablative techniques might not increase the risk of preterm birth compared with women with untreated CIN, but there is uncertainty and they have a higher risk of treatment failure than excisional procedures, particularly in the long term and when used for treatment for CIN3. Given the paucity of data, cold coagulation cannot be considered a safe alternative to LLETZ. We provided estimates of absolute risks of treatment failure and preterm birth for all treatment methods. Results should be interpreted with caution because the evidence base consisted of mostly observational studies with inherent risks of bias.

**Implications of all the available evidence**

The ranking of alternative treatment options and the estimated absolute risks presented in this study might inform national health services, health-care professionals, and patients on the effectiveness and risks of alternative treatment options. These data could be used to counsel patients and assist in clinical decision making. The use of more radical techniques might be a favoured option in selected older women who do not plan a subsequent pregnancy, particularly in the presence of lesions extending into the endocervical canal, because of reduced risk of treatment failure. The choice of treatment should rely on a patient's age, size and location of lesion, and future family planning, because the relative weight of each outcome might vary.

Local conservative treatment for cervical preinvasive and early invasive disease removes or ablates a cone-shaped part of the cervix containing the precancerous cells. Although large loop excision of the transformation zone (LLETZ) is the most commonly used method in the UK, the preference for techniques varies internationally. For example, cold knife conisation (CKC) is still commonly practised in Germany^5^ and laser conisation remains popular in countries including Japan,^6^ whereas cold coagulation and cryotherapy are commonly used in low-income countries.^7^ There is a paucity of evidence on the comparative effectiveness and safety of different treatment methods, and no studies to our knowledge have addressed both issues.

Although complications from treatment were previously thought to be relatively mild and uncommon, evidence published just over a decade ago raised awareness of the increased risk of preterm birth after treatment, particularly for excision and increasing cone length.^8^, ^9^, ^10^, ^11^, ^12^ Subsequently, a trend has been seen towards techniques that remove less tissue.^13^ Ease of execution in an outpatient setting, low cost, and increased awareness of the risk of preterm birth with more radical or deeper techniques might have contributed to this trend. The assumed interchangeability of techniques was largely based on a 2013 Cochrane review of randomised clinical trials that did not report a difference in effectiveness between treatments.^14^ However, this review was not powered to detect differences among highly efficacious treatments. The largest randomised controlled trial recruited only 400 participants, whereas the comparison of CKC to LLETZ included only three studies and 279 participants. A population-based study from Sweden raised further concerns reporting a more than doubled standardised incidence ratio of cervical cancer after treatment in women treated during 2001–08 compared with women treated during 1971–80.^15^ Another meta-analysis published in 2017 reported lower rates of margin positivity and recurrence for knife and laser cones than for LLETZ.^16^

With some researchers advocating the minimum radicality of treatment to prevent treatment-induced reproductive morbidity,^17^ and other researchers raising concerns about the increase in the risk of future invasion,^15^, ^18^ a definite answer regarding the balance of merits against risks among the various treatment strategies is required. Ideally, treatment strategies should be assessed in large trials. Given the similar effectiveness of most treatments, a large-scale randomised controlled trial assessing the relative effectiveness and morbidity of different treatment techniques is unlikely to ever be conducted. Such a trial would require thousands of women to reach adequate power and probably would not manage to recruit a sufficient number because of selective use of treatments by clinicians in different settings. However, the ample observational data in the field provide an opportunity to complement randomised evidence through appropriately designed methods.

As part of the CIRCLE project (Cervical cancer Incidence, Recurrence of CIN and reproduction after Local Excision), we conducted a systematic review and network meta-analysis to synthesise all available evidence from randomised controlled trials and observational studies that compared local treatment techniques for CIN.^19^, ^20^, ^21^ We aimed to estimate comparative effects on treatment failure and preterm birth for all CIN treatments, and to explore the effect of treatment radicality and excised cone length on the outcomes.

## Methods

### Search strategy and selection criteria

In this systematic review and network meta-analysis, we searched MEDLINE, Embase, and the Cochrane Central Register of Controlled Trials database for publications reporting on oncological or reproductive outcomes after CIN treatments from database inception until March 9, 2022, without language restrictions. Search terms are shown in the appendix (pp 5–10).

We included studies of women diagnosed with CIN, stage IA1 cervical cancer, or glandular intraepithelial neoplasia and treated with excision (including CKC, laser conisation, and LLETZ) or ablation (including radical diathermy, laser ablation, cold coagulation [also known as thermal ablation], and cryotherapy). Because radical diathermy is a technique with very scarce evidence and is rarely used in modern practice and hence has little clinical relevance, we only present data for this technique in tables and figures for completeness but do not include them in our narrative of Results. The primary oncological outcome of interest was treatment failure throughout the study period, defined as any abnormal cytology (atypical squamous cells of undetermined significance [known as ASC-US] or worse) or histology (CIN1 or worse). The reproductive outcome assessed was preterm birth (defined as <37 weeks of gestation).

Secondary oncological outcomes assessed in this report were use of different cutoffs for the definition of treatment failure: high-grade treatment failure—ie, high-grade cytology (atypical squamous cells—cannot exclude high-grade squamous intraepithelial lesion [known as ASC-H] or worse) or high-grade histology (CIN2 or worse); histologically confirmed treatment failure (ie, CIN1 or worse); histologically confirmed high-grade treatment failure (ie, CIN2 or worse); positive high-risk HPV testing at 6 months; and invasive cervical cancer incidence after treatment (appendix pp 10–12).

In addition to the treatments listed here, for preterm birth we also included women with CIN attending hospital or clinic for colposcopy without receiving treatment (classified as the untreated colposcopy group) and untreated women without CIN (classified as the untreated external group or general population). For both outcomes, we included quasi-randomised and randomised controlled trials and non-randomised studies with at least two treatment groups. For oncological outcomes, we excluded studies that selectively used ablation for low-grade disease and excision for high-grade disease and studies in which ablation might have been performed in women with endocervical lesions or unsatisfactory colposcopy, or both, or without previous histological confirmation of the lesion. Full inclusion and exclusion criteria are in the appendix (pp 10–12).

Screening and data extraction were done independently by two reviewers (AA and IK) and discrepancies were resolved through discussion with a third reviewer (MK; more details on data extraction are in the appendix [p 12]). First, the title and abstract of identified reports were screened for relevance, and then if they met eligibility criteria the full-text report was obtained for screening and data extraction. In countries with multiple overlapping registry-based studies over the same period, we identified the largest study and only included this in the analysis to avoid multiple inclusion of patients (appendix p 39). For randomised controlled trials, we extracted group-level summary data. For observational studies, we extracted relative treatment effect estimates adjusted for potential confounders, when available.

This systematic review and network meta-analysis was done according to the Cochrane Handbook^22^ and was reported using Preferred Reporting Items for Systematic Reviews and Meta-Analyses (PRISMA) guidelines for network meta-analysis (appendix pp 3–5).^23^ Protocols have been registered with PROSPERO, CRD42018115508 and CRD42018115495, and published previously.^19^, ^20^

### Data analysis

Data were extracted manually by two reviewers (AA and IK) using an a priori developed data collection form (details are in the appendix [p 12]). We synthesised data and calculated summary odds ratios (ORs) and 95% CIs in both standard pairwise (appendix p 14) and network meta-analyses for the primary oncological and reproductive outcomes. In the standard meta-analyses for risk of preterm birth, we included both the untreated colposcopy group and the untreated external group, but in the network meta-analysis for risk of preterm birth, we included only the untreated colposcopy group (appendix p 10). We drew network plots for each outcome (appendix p 14), and we used a random-effects network meta-analysis model. Two reviewers (AA and IK) assessed within-study risk of bias separately for each outcome using RoB 2^24^ for randomised controlled trials and ROBINS-I^25^ for non-randomised studies (appendix pp 12–14).

For identifiability, we assumed heterogeneity (τ^2^) to be the same across all treatment comparisons of each network,^22^ and we estimated it using the DerSimonian and Laird method.^26^ We ranked treatments according to their P-scores; P-scores took values between zero and one, where a higher P-score indicated a better outcome (ie, lower risk of treatment failure or preterm birth).^27^ To assess heterogeneity between studies, we compared the estimated τ^2^ using Turner's empirical distribution for dichotomous data,^28^ and we also compared 95% prediction intervals to 95% CIs (appendix p 15). To assess inconsistency (ie, the difference between direct and indirect evidence), we did both a local test using back-calculation^29^ and a global test using design-by-treatment interaction.^30^

We did prespecified design-adjusted analyses to combine randomised and non-randomised evidence,^31^ in which studies with higher risk of bias were assigned less weight and studies of lower risk of bias were assigned more weight. We split studies into four groups (randomised controlled trials and non-randomised studies at low, moderate, and high risk of bias) and down-weighted the evidence of the three groups of non-randomised studies using four weighting schemes (appendix p 15). We compared the results of the adjusted analyses to those of the unadjusted network meta-analysis.

We did prespecified subgroup analyses of the primary oncological outcome and reproductive outcome according to potential effect modifiers (publication year, age, parity, smoking, method of ascertainment of exposure or outcome, level of income of country, and percentage of women treated for high-grade disease; cutoffs are in the appendix [pp 15–16]). For the outcome of treatment failure, we did post-hoc subgroup analyses on the basis of grade of the treated lesion (ie, biopsy-proven CIN2 or worse or persistent CIN1, CIN3, adenocarcinoma in situ, stage IA1 cervical cancer, or CIN1 or worse without further clarification on whether non-persistent CIN1 had been treated); location of the treated lesion (endocervical *vs* ectocervical) or visibility of transformation zone (satisfactory *vs* unsatisfactory colposcopy), or both; LLETZ technique (top-hat *vs* standard LLETZ); and follow-up duration up to 6 months and throughout the study period, according to the median follow-up duration of studies (at least 12 months, 24 months, 36 months, 48 months, and 60 months). In a separate post-hoc analysis, we included only studies in which more than 20% of women had HIV. Finally, we did two prespecified sensitivity analyses of treatment failure and preterm birth, one excluding all non-randomised and one excluding non-randomised studies at high risk of bias (appendix p 16).

We explored heterogeneity between studies and inconsistency between direct and indirect evidence using the aforementioned subgroup and sensitivity analyses. To assess the plausibility of the transitivity assumption, we examined the distribution of suspected effect modifiers across treatment comparisons (appendix p 15). We used the *netmeta*^32^ package in R (version 4.1.3). We presented the estimated relative treatment effects in league tables and plots, with the presented order of treatments being based on their presumed radicality (appendix p 15). In league tables, we show all possible pairwise comparisons, whereas for plots and for description of results in narrative we focus on comparisons versus LLETZ (the most commonly used technique) for risk of treatment failure, and on comparisons versus the untreated colposcopy group and LLETZ for risk of preterm birth. We rated the credibility of the evidence included in the network meta-analysis using CINeMA^33^ (appendix pp 16–17). For comparisons between treatments reported in at least ten studies, we visually inspected contour-enhanced funnel plots for asymmetry and applied Egger's test.^34^

We estimated absolute risks of treatment failure (for all cutoffs) and preterm birth for each treatment via a prespecified meta-analysis of proportions (appendix p 16). We created a Kilim plot to present absolute risks along with precision of the estimates.^35^ Finally, we did prespecified dose-response meta-analyses to assess the association between cone length and risk of treatment failure and preterm birth. For treatment failure, different cone lengths were compared with cone lengths of 5 mm (using a linear model), whereas for preterm birth, different cone lengths were compared with both the untreated colposcopy group and the untreated external group (using restricted cubic splines; appendix p 16).

### Role of the funding source

The funder of the study had no role in study design, data collection, data analysis, data interpretation, or writing of the report.

## Results

**Figure 1:**
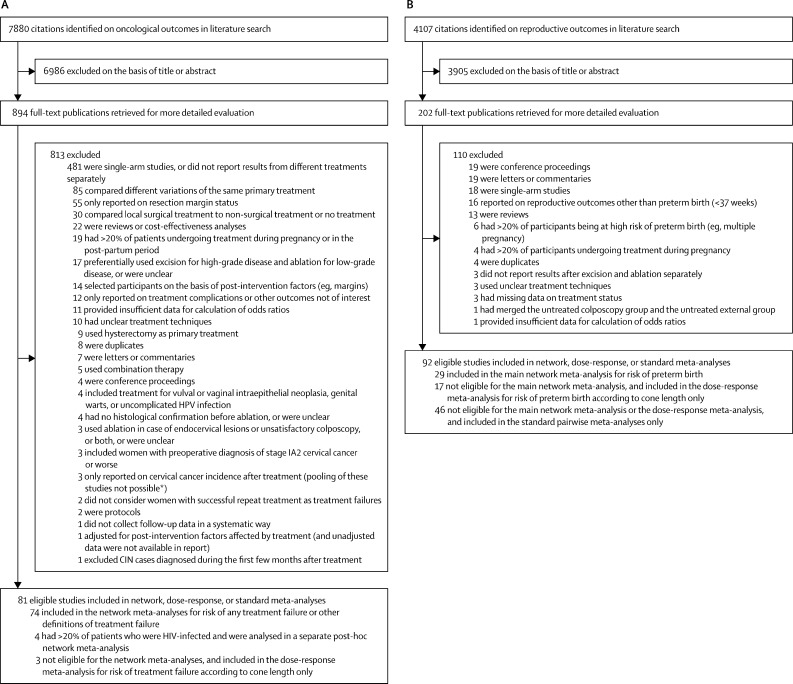
Study selection for oncological outcomes (A) and reproductive outcomes (B) after CIN treatments Where exclusion reasons include “or were unclear”, studies did not have sufficient information on the defined criterion to allow inclusion. CIN=cervical intraepithelial neoplasia. HPV=human papillomavirus. *More information is in the appendix (p 198).

**Figure 2:**
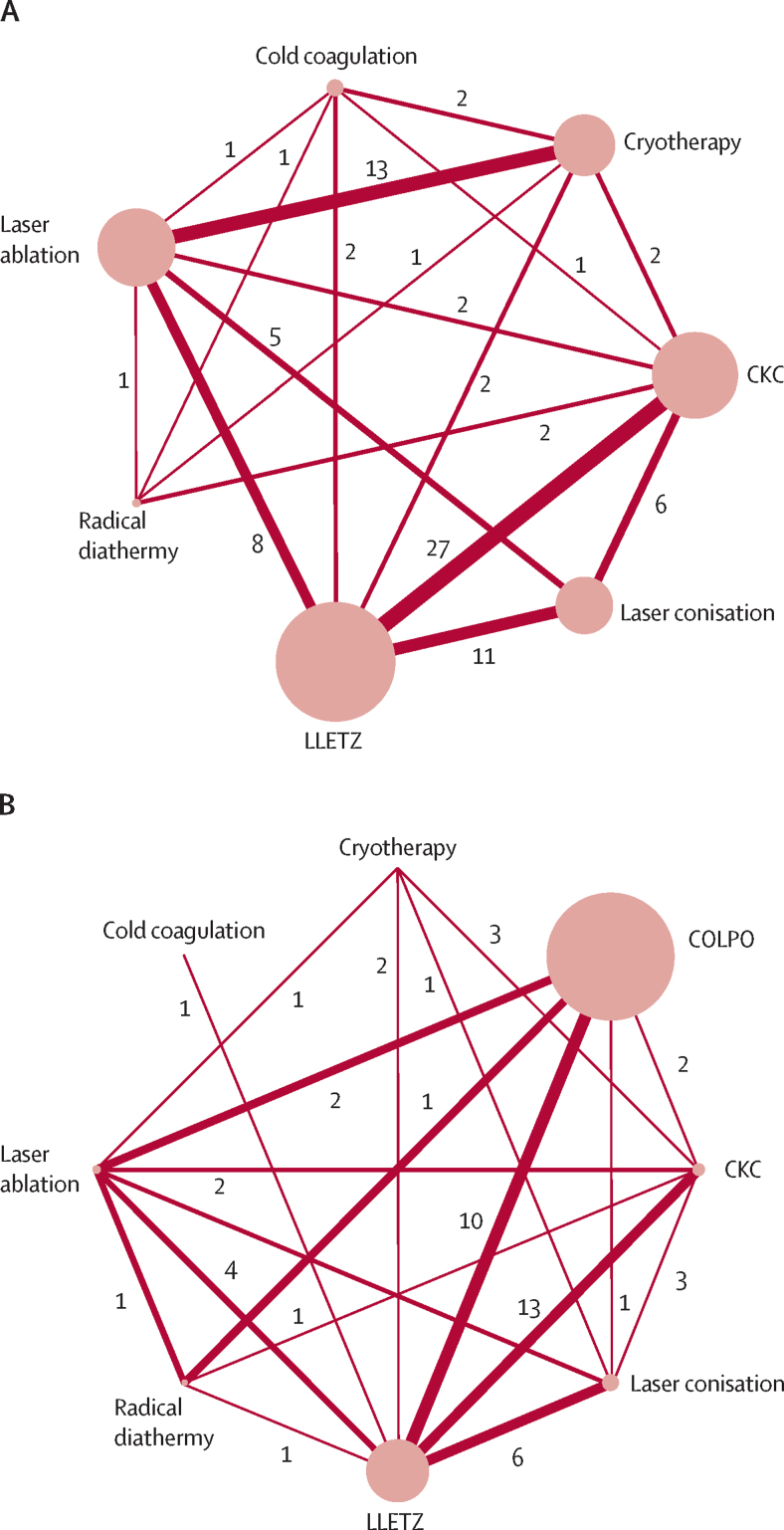
Network plots for risk of CIN treatment failure (A) and preterm birth (B) The width of each line connecting two treatments is proportional to the inverse standard error of the fixed-effect summary effect size for these two treatments (number of studies for each pairwise meta-analysis is also shown). The diameter of each node is proportional to the number of women included in this group. As shown in part A, the network for treatment failure included a total of 19 240 women across 71 studies: CKC (34 studies; n=3865); laser conisation (19 studies; n=2473); LLETZ (43 studies; n=5644); radical diathermy (four studies; n=277); laser ablation (26 studies; n=3539); cold coagulation (six studies; n=667); and cryotherapy (18 studies; n=2775). As shown in part B, the network for preterm birth included a total of 68 817 women across 29 studies: CKC (14 studies; n=2598); laser conisation (nine studies; n=3799); LLETZ (25 studies; n=19 593); radical diathermy (one study; n=760); laser ablation (seven studies; n=1586); cold coagulation (one study; n=56); cryotherapy (three studies; n=67); and COLPO (ten studies; n=40 358). CIN=cervical intraepithelial neoplasia. CKC=cold knife conisation. COLPO=untreated colposcopy group. LLETZ=large loop excision of the transformation zone.

For treatment failure, we identified 7880 potential citations, of which 81 were found to be eligible for inclusion in our systematic review; and for preterm birth, we identified 4107 potential citations, of which 92 were eligible for inclusion in our systematic review (figure 1). Study characteristics and citations are shown in the appendix (pp 18–38). The network for treatment failure included 19 240 treated women in 71 eligible studies (46 non-randomised and 25 randomised controlled trials). The network for preterm birth included 68 817 women (28 459 treated and 40 358 untreated) in 29 eligible studies (27 non-randomised and two randomised controlled trials). In both networks, LLETZ had been assessed in the most studies, whereas radical diathermy and cold coagulation were assessed in the fewest studies (figure 2). Most non-randomised studies (34 [74%] of 46 studies for treatment failure and 14 [52%] of 27 for preterm birth) and some randomised controlled trials (seven [28%] of 25 for treatment failure and none for preterm birth) were at high risk of bias. Most non-randomised studies were downgraded because of the absence of adjustment for confounders, whereas most randomised controlled trials were downgraded because of the absence of a published protocol (appendix pp 40–65). For treatment failure, the median of the median age at treatment across studies was 33 years (IQR 30–36), and the median of the median follow-up duration to diagnose treatment failure was 15 months (IQR 9–35), although this is possibly an underestimate of the true median because nine studies reported only the minimum follow-up duration, and so we assumed that median was equal to the minimum in these studies. For preterm birth, the median of the median age at pregnancy across studies was 30 years (IQR 29–30) and, due to the nature of studies, median follow-up interval could not be calculated (appendix p 27). In both networks, most women had been treated for high-grade disease (median of the proportion treated for CIN2 or worse: 89% [IQR 72–100] for treatment failure, and 83% [70–94] for preterm birth), whereas the proportion of women treated for adenocarcinoma in situ or for stage IA1 cervical cancer was less than 1% in most studies (appendix pp 18, 27).

**Figure 3:**
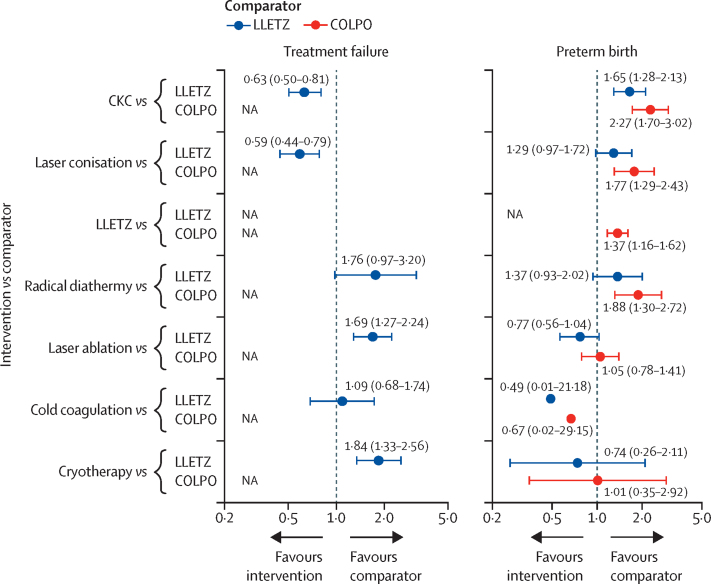
Unadjusted network meta-analyses for risk of CIN treatment failure and preterm birth, with LLETZ or COLPO as reference Data are odds ratios with 95% CIs indicated by error bars or in parentheses. In the network meta-analysis for preterm birth, 95% CIs for cold coagulation are not drawn due to very large uncertainty. CIN=cervical intraepithelial neoplasia. CKC=cold knife conisation. COLPO=untreated colposcopy group. LLETZ=large loop excision of the transformation zone. NA=not applicable.

**Figure 4:**
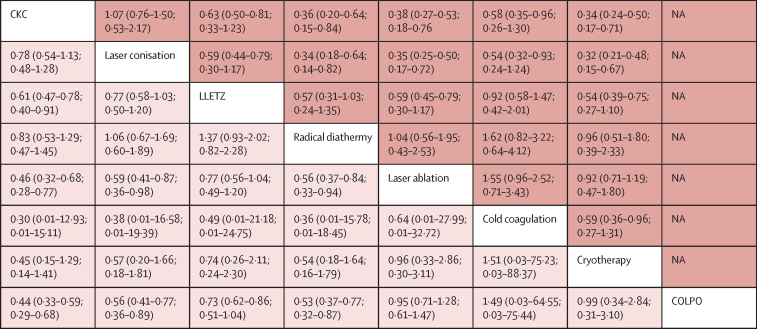
League table of unadjusted network meta-analyses for risk of CIN treatment failure and preterm birth Data are odds ratio (95% CI; 95% prediction interval). The upper half of the grid shows odds ratios for treatment failure; the lower half of the grid shows odds ratios for preterm birth. Each box represents the comparison of the row-defining treatment versus the column-defining treatment. Odds ratios of more than 1 favour the column-defining treatment and odds ratios of less than 1 favour the row-defining treatment. The comparison of the column-defining treatment versus the row-defining treatment is the reciprocal of the data shown**.** CIN=cervical intraepithelial neoplasia. CKC=cold knife conisation. COLPO=untreated colposcopy group. LLETZ=large loop excision of the transformation zone. NA=not applicable

Using the unadjusted network meta-analysis model (Figure 3, Figure 4), the risk of treatment failure was found to be lower for laser conisation (OR 0·59 [95% CI 0·44–0·79]) and CKC (0·63 [0·50–0·81]) than for LLETZ. Most ablative treatments had higher odds of treatment failure than did LLETZ (cryotherapy: 1·84 [1·33–2·56]; and laser ablation: 1·69 [1·27–2·24]), with the exception of cold coagulation, which had a similar risk (1·09 [0·68–1·74]), but only two small studies compared cold coagulation directly with LLETZ (figure 2). On the basis of P-scores, laser conisation and CKC were the highest-ranking treatments, whereas cryotherapy was the lowest-ranking treatment (appendix p 70). Heterogeneity in the network (τ^2^=0·10) was moderate compared with the empirical distribution (appendix p 15). We did not find substantial evidence of inconsistency between direct and indirect evidence (appendix pp 83–84). The distribution of suspected effect modifiers in terms of treatment failure across treatment comparisons are shown in the appendix (pp 101, 104, 107, 110–11, 114, 117, 120, 123, 126); we observed differences in the distribution for publication year (appendix p 101), age (p 104), smoking (p 107), CIN3 or worse (p 120), adenocarcinoma in situ (p 123), and cervical cancer (p 126). Estimates for most treatment comparisons were of low or very low quality mostly due to high risk of within-study bias (appendix pp 223–24). We did not find any evidence of small-study effects or publication bias (appendix pp 217–21). Results from pairwise meta-analyses were in line with the network meta-analysis (appendix pp 66–67).

The design-adjusted network meta-analyses gave broadly similar results to the unadjusted network meta-analysis. Results for laser ablation and cryotherapy were quite conclusive even when we down-weighted non-randomised studies more aggressively and completely removed high-risk studies. For the rest of the treatments, there was uncertainty precluding definitive conclusions (appendix p 93).

All subgroup (including post-hoc) analyses for CIN treatment failure are shown in the appendix (pp 143–45). Treatment failure rates were lower after CKC or laser conisation than after LLETZ in all post-hoc subgroup analyses for tumour location (ie, endocervical or ectocervical) or grade of the lesion (ie, CIN2 or worse or persistent CIN1, CIN3, or adenocarcinoma in situ); an analysis for stage IA1 cervical cancer was not possible because of the small number of cases (appendix pp 143–45). However, effect estimates in most of these analyses were uncertain (ie, the 95% CIs of the ORs crossed 1), except for the analysis restricted to endocervical lesions or unsatisfactory colposcopy, or both (CKC *vs* LLETZ: OR 0·59 [95% CI 0·39–0·91]; laser conisation *vs* LLETZ: 0·44 [0·23–0·84]). For endocervical lesions, LLETZ had a higher risk of treatment failure than did CKC or laser conisation regardless of technique used (top-hat or standard LLETZ; post hoc), although with some uncertainty (appendix p 145). In the prespecified subgroup analysis restricted to studies with a median age of participants of 33 years or older, CKC and laser conisation had a lower risk of treatment failure than did LLETZ (CKC *vs* LLETZ: OR 0·61 [95% CI 0·45–0·81]; laser conisation *vs* LLETZ: 0·40 [0·27–0·59]); no significant differences were found between LLETZ and CKC or laser conisation for women younger than 33 years (appendix pp 143–45). The post-hoc subgroup analysis of studies with potentially some clinically insignificant lesions (ie, non-persistent CIN1) as well as other prespecified subgroup analyses (publication year, smoking, ascertainment of exposure or outcome, and level of income of country) did not materially change the results between LLETZ and other excisional treatments compared with the main analysis.

In the post-hoc subgroup analyses for grade of the lesion, we found that odds ratios of treatment failure after laser ablation and cryotherapy (*vs* LLETZ) increased with increasing CIN grade, and were the highest for CIN3 (laser ablation: OR 2·38 [95% CI 1·03–5·48]; cryotherapy: 3·11 [1·29–7·49]; appendix pp 143–45). Laser ablation and cryotherapy still had higher risk of treatment failure than did LLETZ in the prespecified subgroup analysis restricted to studies of younger women (studies with a median age of <33 years), but effect estimates versus LLETZ were uncertain in studies with participants with a median age of 33 years or older (laser ablation: OR 1·17 [95% CI 0·62–2·19]; cryotherapy: 1·24 [0·34–4·49]) due to a smaller number of studies using ablation in older women (appendix pp 143–45). To control for preferential use of excision for endocervical and ablation for ectocervical lesions, we did a post-hoc subgroup analysis restricted to women with ectocervical lesions only, which confirmed the lower efficacy of laser ablation and cryotherapy than of LLETZ; results did not change when we restricted the analysis to both ectocervical lesions and studies with participants with a median age of less than 33 years (post hoc; appendix pp 143–45). In all these analyses, no differences were found between cold coagulation and LLETZ, but data on cold coagulation were scarce. The post-hoc subgroup analysis of studies with potentially some clinically insignificant lesions and other prespecified subgroup analyses (publication year, smoking, ascertainment of exposure or outcome, and level of income of country) did not materially change the results between LLETZ and ablative treatments compared with the main analysis.

In another post-hoc subgroup analysis of treatment failure, the ORs of cumulative treatment failure rates for laser ablation and cryotherapy compared with LLETZ increased with increasing follow-up duration and were the highest in studies with median follow-up duration of at least 60 months (laser ablation: OR 3·02 [95% CI 1·23–7·39]; cryotherapy: 3·17 [1·36–7·35]); for cold coagulation, the longest study had median follow-up duration of 12 months only (appendix p 192). We found no effect of the length of follow-up for excisional techniques (appendix p 192). A separate post-hoc analysis of HIV-infected women showed consistent results with the main analysis, but very little data were available for this analysis (appendix p 195). The two sensitivity analyses that excluded non-randomised studies and that excluded non-randomised studies at high risk of bias showed consistent results with the main analyses, although there was more uncertainty due to the lower number of included studies than in the main analyses (appendix pp 143–5).

We did secondary analyses of the outcome of treatment failure using different cutoffs for the definitions of treatment failure (cytological ASC-H or worse, or histological CIN2 or worse: 30 studies; histological CIN1 or worse: 22 studies; histological CIN2 or worse: 18 studies; high-risk HPV positivity rates at 6 months: eight studies). Results were consistent with the main analysis, although there was more uncertainty in the estimates due to smaller number of studies than in the main analyses (appendix p 204). An analysis comparing invasive cervical cancer incidence after different techniques was not possible because of the paucity of data (appendix p 198).

For the outcome of preterm birth, in standard meta-analyses we found that untreated women attending for colposcopy (untreated colposcopy group) had a higher risk of preterm birth than did women with no history of CIN (untreated external group; appendix pp 68–69). As such, in pairwise meta-analyses, effect estimates were higher when treatments were compared with the external group than when compared with the colposcopy group (any treatment *vs* external group: OR 1·93 [95% CI 1·70–2·20]; any treatment *vs* colposcopy group: 1·31 [1·18–1·46]; appendix pp 68–69). In the network meta-analysis, we used only the colposcopy group as our untreated comparator (appendix p 10).

Using the unadjusted network meta-analysis model for preterm birth (Figure 3, Figure 4), all excisional techniques increased the risk of preterm birth compared with the untreated colposcopy group (CKC: OR 2·27 [95% CI 1·70–3·02]; laser conisation: 1·77 [1·29–2·43]; and LLETZ: 1·37 [1·16–1·62]), whereas there was no evidence of an effect for laser ablation or cryotherapy. No conclusions could be drawn for the comparison of cold coagulation versus colposcopy due to a very wide confidence interval. When compared with LLETZ, excisional treatments had an increased risk of preterm birth (CKC: 1·65 [1·28–2·13]; similarly for laser conisation albeit with more uncertainty: 1·29 [0·97–1·72]). When compared with LLETZ, ablative techniques had a reduced risk of preterm birth but with uncertainty (laser ablation: 0·77 [0·56–1·04]; cold coagulation and cryotherapy also had a reduced risk, but estimates were much more uncertain especially for cold coagulation; figure 3). P-scores identified laser ablation, followed by cryotherapy and cold coagulation, as being the best treatments, and CKC as being the worst treatment (appendix p 77). Heterogeneity in the network (τ^2^=0·02) was low compared with the empirical distribution (appendix p 15). We found no evidence of inconsistency for risk of preterm birth between direct and indirect data (appendix pp 85–86). By looking at the effect modifiers distribution, we found imbalances for smoking (appendix p 155), but not for any of the other potential effect modifiers. Estimates for most treatment comparisons were of low or very low quality mostly due to imprecision (appendix pp 232–34). No small-study effects or publication bias were detected (appendix pp 227–30). Results from pairwise meta-analyses were largely in agreement with the network meta-analysis (appendix pp 68–69).

The design-adjusted network meta-analyses gave almost identical results to the unadjusted network meta-analysis and did not affect conclusions drawn (appendix p 99). Prespecified subgroup and sensitivity analyses for preterm birth are shown in the appendix (pp 182–83). We found that ORs for laser ablation and cryotherapy versus the untreated colposcopy group were both well above 1 (OR >1·50) in the prespecified subgroup analyses of studies where proportion of nulliparae was less than 49%, of studies where ascertainment of exposure was through registries, and of studies where proportion of women treated for CIN2 or worse was more than 83% and for CIN3 or worse was more than 61%, but effect estimates were imprecise, preventing us from drawing strong conclusions. We did not find any substantial differences between the main analyses and other subgroup or sensitivity analyses.

**Figure 5:**
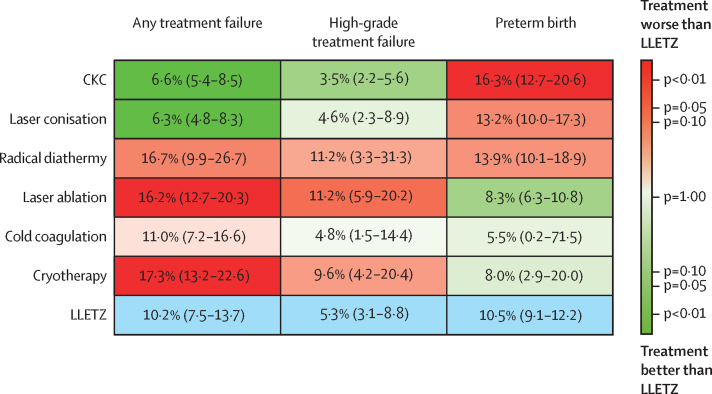
Absolute risks of CIN treatment failure and preterm birth (Kilim plot) Each box shows the absolute risk of treatment failure or preterm birth for each treatment. The colour correlates to the strength of the statistical evidence regarding the comparison of each treatment versus LLETZ. Colours correlating with a p value close to 1·00 indicate that there is paucity of evidence regarding whether the treatment is worse or better than LLETZ. LLETZ (the comparator) is shown in blue. CIN=cervical intraepithelial neoplasia. CKC=cold knife conisation. LLETZ=large loop excision of the transformation zone.

The absolute risk of any treatment failure was lowest for laser conisation and CKC, and highest for cryotherapy and laser ablation (figure 5). Cold coagulation had a similar risk of failure as LLETZ, but data on cold coagulation were limited. When we used high-grade disease as a cutoff for treatment failure, ranking of treatments was similar but there was more uncertainty in the estimates due to smaller number of studies using each treatment (figure 5). Absolute risks for other cutoffs of treatment failure are presented in the appendix (pp 206–07) . The absolute risk of preterm birth was highest for CKC, followed by laser conisation and LLETZ (figure 5). Most ablative techniques had a risk of preterm birth similar to that of the untreated colposcopy group (7·9% [95% CI 6·3–9·9]), except for cold coagulation, which had a lower risk but had much uncertainty (5·5% [0·2–71·5]) because the estimate was based on a single study of low quality.

In our dose-response meta-analyses according to length of excised cone, in a linear model based on only three studies, we found that risk of treatment failure was inversely proportional to length of excised cone (appendix p 211). Conversely, by using restricted cubic splines, rates of preterm birth increased proportionally with cone length (20 studies; appendix pp 212–13). Cone lengths up to and including 10 mm were associated with a small increase in the risk of preterm birth when compared with the untreated colposcopy group, whereas lengths over 10 mm with a greater increase in risk (10 mm [10·4% absolute risk] *vs* colposcopy [7·9%]: OR 1·31 [95% CI 1·11–1·55]; 15 mm [17·2%] *vs* colposcopy: OR 2·17 [1·69–2·80]; 20 mm [32·4%] *vs* colposcopy: 4·09 [2·68–6·23]; appendix p 212). When compared with the general population, the dose–response curve increased more steeply and risk of preterm birth was increased even for cone lengths of less than 10 mm (appendix p 213).

## Discussion

To our knowledge, this is the first network meta-analysis to explore the comparative effectiveness and reproductive morbidity for different treatments for CIN. Our findings suggest that more aggressive local CIN treatments are associated with a reduced risk of treatment failure but an increased risk of preterm birth in subsequent pregnancies. We identified previously unknown differences and reported lower rates of treatment failure among more radical excisional treatment techniques (CKC or laser conisation) than with LLETZ. Our results for treatment failure were consistent in secondary analyses using various cutoffs for definition of treatment failure and for high-risk HPV positivity rates at 6 months. Our findings are in contrast with those of a Cochrane meta-analysis of randomised controlled trials that found no difference in efficacy among different CIN treatments,^14^ probably due to the small number of studies included in each pairwise comparison. In subgroup analyses, we found that CKC was more effective than LLETZ for treatment of endocervical lesions (post hoc) and when done in older (median age of ≥33 years) women (prespecified). Our results are in agreement with a meta-analysis of 26 studies that also reported reduced rates of treatment failure for CKC when compared with LLETZ in women with unsatisfactory colposcopy,^36^ although our effect estimates had more certainty because of the increased precision of the network meta-analysis. As such, the use of more radical excision with knife or laser might be a favoured option in selected patients who are older and who do not plan to have a subsequent pregnancy, particularly in the presence of endocervical lesions.

Whether or not ablative techniques confer equal rates of treatment failure to that of excision has been a matter of controversy and debate. The American Society of Colposcopy and Cervical Pathology supports the use of excision over ablation for treatment of high-grade disease,^37^ whereas WHO does not make such a recommendation.^7^ Although similar rates of treatment failure were found between all excisional and ablative techniques in the Cochrane meta-analysis of randomised controlled trials^14^ and between LLETZ and cryotherapy in a more recent meta-analysis of randomised controlled trials and non-randomised studies,^38^ the evidence was weak because of the small number of studies in each pairwise comparison. In our network meta-analysis, we found that laser ablation and cryotherapy were associated with almost twice the risk of failure compared with LLETZ. When the analysis was restricted to younger women (median age of <33 years) with only ectocervical lesions (post hoc), laser ablation and cryotherapy remained less efficacious than LLETZ. In a post-hoc subgroup analysis restricted to women with CIN3, the ORs of treatment failure after ablative techniques versus LLETZ were even higher, in particular for cryotherapy, supporting that cryotherapy should not be recommended for high-grade disease. In another subgroup analysis restricted to studies with follow-up duration of at least 60 months, the risk of treatment failure with ablative techniques was more than three times greater than with LLETZ, raising further concerns about their long-term oncological safety; the length of follow-up did not change the results for excisional techniques. Although cold coagulation has been proposed as being oncologically safe,^39^ and our analysis suggested similar rates of treatment failure as LLETZ, direct data came from only two studies with less than 1 year of follow-up and there was uncertainty around the estimates (appendix p 192). In the absence of safety data, cold coagulation cannot be considered a safe alternative to LLETZ.

Untreated women attending for colposcopy have a higher risk of preterm birth than do women without CIN, probably explained by inherent characteristics and genetic, epigenetic, or microbiome factors.^40^, ^41^, ^42^ When compared with the untreated colposcopy group, women undergoing excisional treatment had increased rates of preterm birth in our network meta-analysis, whereas those who had ablative treatment did not. However, when the analysis was restricted to studies in which most women were being treated for CIN3 or worse, ablative treatments led to higher rates of premature birth than were seen in the untreated colposcopy group, although estimates were uncertain. Therefore, the reproductive health risks from ablative treatments might be underestimated because of the preferential use of these techniques in women with less severe disease.

Knowledge of cone length might assist in the selection of patients who should be offered antenatal surveillance. The value of antenatal interventions with cervical length screening with or without cerclage or progesterone, or both, after CIN treatment remains uncertain. A meta-analysis of 196 patients found that the existing evidence does not support cerclage or other interventions for the prevention of preterm birth (<37 weeks), although the analysis was not adjusted for cone length or treatment technique.^43^ Based on our calculated absolute risks for preterm birth, women that would probably benefit the most are those with cone lengths of more than 10 mm and particularly more than 15 mm. If cone length is unknown, women undergoing CKC, laser conisation, and large or repeat LLETZ are also likely to benefit from cervical length screening. Clinicians should attempt to limit the length of excision to 10 mm in women planning future conception who have a visible transformation zone.^44^ Conversely, inappropriately superficial excisions in an attempt to minimise adverse pregnancy sequelae might compromise long-term oncological safety^45^ and could be partly accountable for the increased post-treatment incidence of cervical cancer.^15^, ^18^

A major strength of our study was the inclusion of the entire evidence base for different treatments for CIN and that we combined randomised and observational data via state-of-the-art statistical methods. We did design-adjusted analyses in which more weight was given to randomised controlled trials and high-quality observational studies. We observed that the results did not change between design-adjusted and unadjusted analyses, or when we included only randomised evidence, increasing our confidence in the results and that these had not been distorted by studies at high risk of bias. Heterogeneity was low to moderate, and neither local nor global statistical tests found any evidence of inconsistency. We did a series of subgroup and sensitivity analyses to control for suspected confounders and presented both relative effect estimates and absolute risks.

Our study also has several limitations. Data were dominated by observational data, thus bias might be present in the estimated treatment effects because of unmeasured confounding. Little data were available for radical diathermy, cold coagulation, and cryotherapy. Possible inclusion of women with clinically insignificant lesions (ie, non-persistent CIN1) in some studies, preferential use of excision or ablation on the basis of the severity and location of the lesion, and differences in the distribution of publication year, age, adenocarcinoma in situ, cervical cancer, and CIN3 or worse across treatment comparisons might have led to further bias in our calculations for the risk of treatment failure. We did a series of subgroup and sensitivity analyses that had consistent results with the main analysis. A subgroup analysis of studies with possibly insignificant lesions showed consistent findings with the main analysis for treatment failure. Although the differences in the distribution of publication year might lead to bias due to changes in technologies and techniques over time, results were similar between the older and more recent studies, and differences are likely attributed to the later introduction of techniques like LLETZ. The magnitude of difference in efficacy between ablation and excision might have been diluted by the preferential use of excision in older women and for more severe or endocervical lesions because we saw a difference in distribution of age, adenocarcinoma in situ, and cervical cancer between excisional and ablative treatments. We excluded studies that preferentially used excision for high-grade disease and ablation for low-grade disease, and we did subgroup analyses of younger women, ectocervical lesions only, and studies excluding adenocarcinoma in situ or cervical cancer, which did not change the results. Although the distribution of CIN2 or worse was similar, the proportion of women treated for CIN3 or worse was lower in ablative than excisional techniques. Because odds ratios of treatment failure and preterm birth after ablation (*vs* LLETZ and colposcopy, respectively) were higher in the subgroup analysis restricted to patients with CIN3 or worse than in the main network meta-analyses, the magnitude of risks from ablation for severe disease might be underestimated in the main analyses, but our conclusions would not change.

The evidence on the clinical ranking of alternative treatment options, the risk according to cone length, and the absolute risks presented in this Article could be used to counsel patients, assist clinicians and public health policy makers, and select women at high risk who would benefit from intensive surveillance after treatment or antenatally, while minimising the unnecessary interventions for those at low risk of treatment failure or preterm birth.^46^ Although LLETZ seemed to have balanced effectiveness and reproductive morbidity, the choice of treatment should rely on a woman's age, size and location of lesion, and fertility wishes because the relative weight of each outcome might vary. Widespread HPV vaccination is expected to substantially reduce the burden of cervical disease and treatment-related reproductive morbidity.^2^, ^3^, ^47^ For unvaccinated women, the role of vaccination at the time of treatment is being investigated (NCT03979014).^48^

**This online publication has been corrected. The corrected version first appeared at thelancet.com/oncology on July 25, 2022**

## Data sharing

This systematic review and network meta-analysis used data from published studies; data are available from these individual published studies

## Declaration of interests

OE has received consulting fees from Biogen (payments were made to their University). All other authors declare no competing interests.
