# Correction to *Lancet Oncol* 2022; 23: 1097–108

**DOI:** 10.1016/S1470-2045(22)00455-7

**Published:** 2022-08

*Athanasiou A, Veroniki AA, Efthimiou O, et al. Comparative effectiveness and risk of preterm birth of local treatments for cervical intraepithelial neoplasia and stage IA1 cervical cancer: a systematic review and network meta-analysis.* Lancet Oncol *2022;* 23: *1097–108—* In this Article, in figure 1, one of the exclusion criteria in part B should have been written as “1 had merged the untreated colposcopy group and the untreated external group”; the second sentence of the fourth paragraph of the Data analysis section should have referred to “the grade of the treated lesion” and “the location of the treated lesion”; the eighth sentence of the first paragraph of the Results should have referred to “the median of the median follow-up duration”; and the tenth paragraph of the Results section should have started with the sentence “The design-adjusted network meta-analyses gave almost identical results to the unadjusted network meta-analysis and did not affect conclusions drawn (appendix p 99).” The appendix has also been updated. These corrections have been made to the online versions as of July 25, 2022, and the printed version is correct.

