# Supplementary material for: Comparative effectiveness and risk of preterm birth of local treatments for cervical intraepithelial neoplasia and stage IA1 cervical cancer: a systematic review and network meta-analysis
Source: Lancet Oncol. 2022 Aug;23(8):1097–108. doi: 10.1016/S1470-2045(22)00334-5 (PMC9630146; doi:10.1016/S1470-2045(22)00334-5)
Supplement: Supplementary appendix [file mmc1.pdf]

THE LANCET
Oncology

Supplementary appendix

This appendix formed part of the original submission and has been peer reviewed. We post it as supplied by the authors.

This online publication has been corrected. The corrected version first appeared at thelancet.com/oncology on July 25, 2022

Supplement to: Athanasiou A, Veroniki AA, Efthimiou O, et al. Comparative effectiveness and risk of preterm birth of local treatments for cervical intraepithelial neoplasia and stage IA1 cervical cancer: a systematic review and network meta-analysis. *Lancet Oncol* 2022; published online July 11. https://doi.org/10.1016/S1470-2045(22)00334-5.

Appendix

Table of Contents

1. Supplementary Materials & Methods *3*

1.1. PRISMA Checklist **3**

1.2. Search Strategy **5**

1.2.1. Treatment Failure 5

1.2.2. Preterm Birth 7

1.3. Selection Criteria **10**

1.4. Data Analysis **12**

1.4.1. Data Extraction 12

1.4.2. Within-Study Risk of Bias 12

1.4.2.1. Treatment Failure 12

1.4.2.2. Preterm Birth 13

1.4.3. Standard Pairwise Meta-Analyses 14

1.4.4. Network Meta-Analyses 14

1.5. Additional Analyses **16**

1.5.1. Meta-Analyses of Proportions 16

1.5.2. Dose-Response Meta-Analyses 16

1.6. Assessment of the Credibility of Evidence (CINeMA) **16**

2. Supplementary Results *18*

2.1. Characteristics of Studies **18**

2.1.1. Treatment Failure 18

2.1.2. Preterm Birth 27

2.2. Overlapping Studies **39**

2.2.1. Treatment Failure 39

2.2.2. Preterm Birth 39

2.3. Within-Study Risk of Bias **40**

2.3.1. Treatment Failure 40

2.3.2. Preterm Birth 52

2.4. Standard Meta-Analyses **66**

2.4.1. Treatment Failure 66

2.4.2. Preterm Birth 68

2.5. Network Meta-Analyses **70**

2.5.1. Treatment Failure 70

2.5.2. Preterm Birth 77

2.5.3. Trade-off between risk of treatment failure and risk of preterm birth 82

2.6. Tests for Inconsistency **83**

2.6.1. Treatment Failure 83

2.6.2. Preterm Birth 85

2.7. Design-Adjusted Network Meta-Analyses **87**

2.7.1. Treatment Failure 88

Summary Figure *93*

2.7.2. Preterm Birth 94

Summary Figure *99*

2.8. Subgroup and Sensitivity Analyses **100**

2.8.1. Treatment Failure 101

Summary Table *143*

2.8.2. Preterm Birth 146
Summary Table *182*

2.9. Effect of Follow-up Duration 184
2.9.1. Treatment Failure 184
Summary Figure *192*
2.9.2. Preterm Birth 193

2.10. HIV-Infected Women 194
2.10.1. Treatment Failure 194
2.10.2. Preterm Birth 197

2.11. Different Definitions of Outcome 198
2.11.1. Treatment Failure 198
Summary Table *204*
2.11.2. Preterm Birth 205

2.12. Absolute Risks 206
2.12.1. Treatment Failure 206
2.12.2. Preterm Birth 208

2.13. Effect of Cone Length 210
2.13.1. Treatment Failure 211
2.13.2. Preterm Birth 212

2.14. Assessment of the Credibility of Evidence (CINeMA) 214
2.14.1. Treatment Failure 215
2.14.2. Preterm Birth 225

3. Supplementary Discussion 235

4. Abbreviations 236

5. References 237

1. Supplementary Materials & Methods

1.1. PRISMA Checklist

We have followed the PRISMA-NMA guidelines for reporting.[1] Checklist tables are provided below:

Table 1.1.1: PRISMA NMA checklist of items to include when reporting a systematic review involving a network meta-analysis

Section/Topic	Item #	Checklist Item	Reported on Page #
**Title**			
Title	1	Identify the report as a systematic review incorporating a network meta-analysis (or related form of meta-analysis).	1
**Abstract**			
Structured summary	2	Provide a structured summary including, as applicable:<br>Background: main objectives<br>Methods: data sources; study eligibility criteria, participants, and interventions; study appraisal; and synthesis methods, such as network meta-analysis.<br>Results: number of studies and participants identified; summary estimates with corresponding confidence/credible intervals; treatment rankings may also be discussed. Authors may choose to summarize pairwise comparisons against a chosen treatment included in their analyses for brevity.<br>Discussion/Conclusions: limitations; conclusions and implications of findings.<br>Other: primary source of funding; systematic review registration number with registry name.	1 (We followed PRISMA checklist for NMA abstract; see next Table 1.1.2)
**Introduction**			
Rationale	3	Describe the rationale for the review in the context of what is already known, including mention of why a network meta-analysis has been conducted.	1-3
Objectives	4	Provide an explicit statement of questions being addressed, with reference to participants, interventions, comparisons, outcomes, and study design (PICOS*).	3
**Methods**			
Protocol and registration	5	Indicate whether a review protocol exists and if and where it can be accessed (e.g., Web address); and, if available, provide registration information, including registration number.	3
Eligibility criteria	6	Specify study characteristics (e.g., PICOS, length of follow-up) and report characteristics (e.g., years considered, language, publication status) used as criteria for eligibility, giving rationale. Clearly describe eligible treatments included in the treatment network, and note whether any have been clustered or merged into the same node (with justification).	3; Appendix 1.3
Information sources	7	Describe all information sources (e.g., databases with dates of coverage, contact with study authors to identify additional studies) in the search and date last searched.	3; Appendix 1.2
Search	8	Present full electronic search strategy for at least one database, including any limits used, such that it could be repeated.	Appendix 1.2
Study selection	9	State the process for selecting studies (i.e., screening, eligibility, included in systematic review, and, if applicable, included in the meta-analysis).	3
Data collection process	10	Describe method of data extraction from reports (e.g., piloted forms, independently, in duplicate) and any processes for obtaining and confirming data from investigators.	3; Appendix 1.4.1
Data items	11	List and define all variables for which data were sought (e.g., PICOS, funding sources) and any assumptions and simplifications made.	Appendix 1.4.1
Geometry of the network	S1	Describe methods used to explore the geometry of the treatment network under study and potential biases related to it. This should include how the evidence base has been graphically summarized for presentation, and what characteristics were compiled and used to describe the evidence base to readers.	3-4; Appendix 1.4.4
Risk of bias within individual studies	12	Describe methods used for assessing risk of bias of individual studies (including specification of whether this was done at the study or outcome level), and how this information is to be used in any data synthesis.	4; Appendix 1.4.2
Summary measures	13	State the principal summary measures (e.g., risk ratio, difference in means). Also describe the use of additional summary measures assessed, such as treatment rankings and surface under the cumulative ranking curve (SUCRA) values, as well as modified approaches used to present summary findings from meta-analyses.	3-4
Planned methods of analysis	14	Describe the methods of handling data and combining results of studies for each network meta-analysis. This should include, but not be limited to:<br>• Handling of multi-arm trials;<br>• Selection of variance structure;<br>• Selection of prior distributions in Bayesian analyses; and<br>• Assessment of model fit.	3-5

Assessment of Inconsistency	S2	Describe the statistical methods used to evaluate the agreement of direct and indirect evidence in the treatment network(s) studied. Describe efforts taken to address its presence when found.	4
Risk of bias across studies	15	Specify any assessment of risk of bias that may affect the cumulative evidence (e.g., publication bias, selective reporting within studies).	5; Appendix 1.6
Additional analyses	16	Describe methods of additional analyses if done, indicating which were pre-specified. This may include, but not be limited to, the following:<br>• Sensitivity or subgroup analyses;<br>• Meta-regression analyses;<br>• Alternative formulations of the treatment network; and<br>• Use of alternative prior distributions for Bayesian analyses (if applicable).	5-6; Appendix 1.4.4 and 1.5
**Results**			
Study selection	17	Give numbers of studies screened, assessed for eligibility, and included in the review, with reasons for exclusions at each stage, ideally with a flow diagram.	6; Figure 1
Presentation of network structure	S3	Provide a network graph of the included studies to enable visualization of the geometry of the treatment network.	Figure 2
Summary of network geometry	S4	Provide a brief overview of characteristics of the treatment network. This may include commentary on the abundance of trials and randomized patients for the different interventions and pairwise comparisons in the network, gaps of evidence in the treatment network, and potential biases reflected by the network structure.	6
Study characteristics	18	For each study, present characteristics for which data were extracted (e.g., study size, PICOS*, follow-up period) and provide the citations.	Appendix 2.1
Risk of bias within studies	19	Present data on risk of bias of each study and, if available, any outcome level assessment.	6; Appendix 2.3
Results of individual studies	20	For all outcomes considered (benefits or harms), present, for each study: 1) simple summary data for each intervention group, and 2) effect estimates and confidence intervals. Modified approaches may be needed to deal with information from larger networks.	Appendix Tables 2.5.1.2 and 2.5.2.2
Synthesis of results	21	Present results of each meta-analysis done, including confidence/credible intervals. In larger networks, authors may focus on comparisons versus a particular comparator (e.g. placebo or standard care), with full findings presented in an appendix. League tables and forest plots may be considered to summarize pairwise comparisons. If additional summary measures were explored (such as treatment rankings), these should also be presented.	6 and 8; Figures 3 and 4; Appendix 2.5
Exploration for inconsistency	S5	Describe results from investigations of inconsistency. This may include such information as measures of model fit to compare consistency and inconsistency models, P values from statistical tests, or summary of inconsistency estimates from different parts of the treatment network.	6 and 8; Appendix 2.6
Risk of bias across studies	22	Present results of any assessment of risk of bias across studies for the evidence base being studied.	6 and 8; Appendix 2.14
Results of additional analyses	23	Give results of additional analyses, if done (e.g., sensitivity or subgroup analyses, meta-regression analyses, alternative network geometries studied, alternative choice of prior distributions for Bayesian analyses, and so forth).	7–9; Figure 5; Appendix 2.7–2.13
**Discussion**			
Summary of evidence	24	Summarize the main findings, including the strength of evidence for each main outcome; consider their relevance to key groups (e.g., healthcare providers, users, and policy-makers).	9–10
Limitations	25	Discuss limitations at study and outcome level (e.g., risk of bias), and at review level (e.g., incomplete retrieval of identified research, reporting bias). Comment on the validity of the assumptions, such as transitivity and consistency. Comment on any concerns regarding network geometry (e.g., avoidance of certain comparisons).	10; Appendix Section 3
Conclusions	26	Provide a general interpretation of the results in the context of other evidence, and implications for future research.	10-11
**Funding**			
Funding	27	Describe sources of funding for the systematic review and other support (e.g., supply of data); role of funders for the systematic review. This should also include information regarding whether funding has been received from manufacturers of treatments in the network and/or whether some of the authors are content experts with professional conflicts of interest that could affect use of treatments in the network.	6 and 11

*PICOS: population, intervention, comparators, outcomes, study design

Table 1.1.2: PRISMA checklist for NMA abstract

Section and Topic	Item #	Checklist item	Reported (Yes/No)
**Title**			
Title	1	Identify the report as a systematic review.	Yes
**Background**			
Objectives	2	Provide an explicit statement of the main objective(s) or question(s) the review addresses.	Yes

**Methods**			
Eligibility criteria	3	Specify the inclusion and exclusion criteria for the review.	Yes
Information sources	4	Specify the information sources (e.g. databases, registers) used to identify studies and the date when each was last searched.	Yes
Risk of bias	5	Specify the methods used to assess risk of bias in the included studies.	Yes
Synthesis of results	6	Specify the methods used to present and synthesise results.	Yes
**Results**			
Included studies	7	Give the total number of included studies and participants and summarise relevant characteristics of studies.	Yes
Synthesis of results	8	Present results for main outcomes, preferably indicating the number of included studies and participants for each. If meta-analysis was done, report the summary estimate and confidence/credible interval. If comparing groups, indicate the direction of the effect (i.e. which group is favoured).	Yes
**Discussion**			
Limitations of evidence	9	Provide a brief summary of the limitations of the evidence included in the review (e.g. study risk of bias, inconsistency and imprecision).	Yes
Interpretation	10	Provide a general interpretation of the results and important implications.	Yes
**Other**			
Funding	11	Specify the primary source of funding for the review.	Yes
Registration	12	Provide the register name and registration number.	Yes

1.2. Search Strategy

We searched MEDLINE, Embase and CENTRAL for randomised controlled trials (RCTs) and non-randomised studies (NRS). We also searched www.clinicaltrials.gov, the International Clinical Trials Registry Platform (ICTRP), ZETOC (http://zetoc.jisc.ac.uk/) for conference proceedings from British Society of Colposcopy and Cervical Pathology (BSCCP), International Federation of Cervical Pathology and Colposcopy (IFCPC), European Federation of Colposcopy (EFC) and American Society of Colposcopy and Cervical Pathology (ASCCP), and WorldCat for dissertations and theses. Finally, we hand-searched the citations of identified studies, we checked the list of 'Similar articles' provided by MEDLINE, and we contacted experts in the field to identify studies that were possibly missed by our search algorithm. We did not identify any unpublished data.There was no time or language restriction.

Search algorithms for MEDLINE, Embase and CENTRAL are provided below:

1.2.1. Treatment Failure

MEDLINE Ovid – RCTs only

1 exp Cervical Intraepithelial Neoplasia/
2 CIN.mp.
3 (cervi* and (intraepithel* or epithel*)).mp.
4 (cervi* and dysplasia).mp.
5 (cervi* and carcinoma in situ).mp.
6 (cervi* and cancer in situ).mp.
7 (cervi* and (precancer* or pre-cancer*)).mp.
8 or/1–7
9 surgery.fs.
10 exp Gynecologic Surgical Procedures/
11 (surg* or ablat* or excis* or cryotherapy or laser* or cone or conisation or biopsy or transformation zone or LLETZ or LEEP).mp.
12 or/9–11
13 8 and 12
14 randomized controlled trial.pt.
15 controlled clinical trial.pt.
16 randomized.ab.
17 placebo.ab.
18 clinical trials as topic.sh.
19 randomly.ab.
20 trial.ti.
21 or/14–20
22 13 and 21

MEDLINE Ovid – All Studies

1 exp Cervical Intraepithelial Neoplasia/
2 CIN.mp.

3 (cervi* and (intraepithel* or epithel*)).mp.
4 (cervi* and dysplasia).mp.
5 (cervi* and carcinoma in situ).mp.
6 (cervi* and cancer in situ).mp.
7 (cervi* and (precancer* or pre-cancer*)).mp.
8 or/1–7
9 surgery.fs.
10 exp Gynecologic Surgical Procedures/
11 (surg* or ablat* or excis* or cryotherapy or laser* or cone or conisation or biopsy or transformation zone or LLETZ or LEEP).mp.
12 or/9–11
13 8 and 12
14 randomized controlled trial.pt.
15 controlled clinical trial.pt.
16 randomized.ab.
17 placebo.ab.
18 clinical trials as topic.sh.
19 randomly.ab.
20 trial.ti.
21 groups.ab.
22 exp cohort studies/
23 exp case-control studies/
24 (cohort* or prospective* or retrospective* or (case* and (control* or series))).mp.
25 or/12–24
26 (animals not (humans and animals)).sh.
27 25 not 26
28 13 and 27

Embase Ovid – RCTs only

1 exp Uterine Cervix Carcinoma in Situ/
2 CIN.mp.
3 (cervi* and (intraepithel* or epithel*)).mp.
4 (cervi* and dysplasia).mp.
5 (cervi* and carcinoma in situ).mp.
6 (cervi* and cancer in situ).mp.
7 (cervi* and (precancer* or pre-cancer*)).mp.
8 or/1–7
9 su.fs.
10 exp gynecologic surgery/
11 (surg* or ablat* or excis* or cryotherapy or laser* or cone or conisation or biopsy or transformation zone or LLETZ or LEEP).mp.
12 or/9–11
13 8 and 12
14 crossover procedure/
15 double-blind procedure/
16 randomized controlled trial/
17 single-blind procedure/
18 random*.mp.
19 factorial*.mp.
20 (crossover* or cross over* or cross-over*).mp.
21 placebo*.mp.
22 (double* adj blind*).mp.
23 (singl* adj blind*).mp.
24 assign*.mp.
25 allocat*.mp.
26 volunteer*.mp.
27 or/14–26
28 13 and 27

Embase Ovid – All Studies

1 exp Uterine Cervix Carcinoma in Situ/
2 CIN.mp.
3 (cervi* and (intraepithel* or epithel*)).mp.
4 (cervi* and dysplasia).mp.
5 (cervi* and carcinoma in situ).mp.
6 (cervi* and cancer in situ).mp.
7 (cervi* and (precancer* or pre-cancer*)).mp.
8 or/1–7
9 su.fs.
10 exp gynecologic surgery/
11 (surg* or ablat* or excis* or cryotherapy or laser* or cone or conisation or biopsy or transformation zone or LLETZ or LEEP).mp.
12 or/9–11
13 8 and 12
14 exp controlled clinical trial/
15 randomized.ab.
16 randomly.ab.
17 trial.ab.
18 groups.ab.
19 exp cohort analysis/
20 cohort*.mp.
21 exp retrospective study/
22 exp prospective study/
23 (case* and series).mp.
24 or/14–23
25 13 and 24

CENTRAL

1 MeSH descriptor **Cervical Intraepithelial Neoplasia** explode all trees
2 CIN
3 cervi* and (intraepithel* or epithel*)
4 cervi* and dysplasia
5 cervi* and carcinoma in situ
6 cervi* and cancer in situ
7 cervi* and (precancer* or pre-cancer*)
8 or/1–7
9 Any MeSH descriptor with qualifier(s): [Surgery – SU]
10 MeSH descriptor Gynecologic **Surgical Procedures** explode all trees
11 surg* or ablat* or excis* or cryotherapy or laser* or cone or conisation or biopsy or transformation zone or LLETZ or LEEP
12 or/9–11
13 8 and 12

1.2.2. Preterm Birth

MEDLINE Ovid

1 exp Uterine Cervical Neoplasms/
2 (cervi* and (cancer* or tumor* or tumour* or neoplas* or malignan* or carcinom*)).mp.
3 exp Cervical Intraepithelial Neoplasia/
4 CIN.mp.
5 (cervi* and (intraepithel* or epithel* or dysplasia or pre-cancer* or precancer*)).mp.
6 or/1–5
7 exp Conization/
8 (conisation or conization).mp.
9 exp Laser Therapy/
10 laser.mp.
11 exp Cryotherapy/
12 cryotherapy.mp.
13 cold coagulation.mp.
14 exp Diathermy/

15 diatherm*.mp.
16 cone biopsy.mp.
17 loop.mp.
18 LLETZ.mp.
19 LEEP.mp.
20 ablat*.mp.
21 excision*.mp.
22 transformation zone.mp.
23 (CKC or LA or LC or CC or RD or TZ).mp.
24 (conservative and (method* or treatment* or intervention* or management)).mp.
25 or/7–24
26 6 and 25
27 exp Premature Birth/
28 (preterm or premature).mp.
29 exp Infant, Low Birth Weight/
30 birth weight.mp.
31 exp Perinatal Mortality/
32 perinatal mortality.mp.
33 exp Intensive Care, Neonatal/
34 (neonatal and intensive care).mp.
35 exp Fertility/
36 fertil*.mp.
37 conception.mp.
38 exp Pregnancy/
39 pregnancy.mp.
40 gestation*.mp.
41 exp Abortion, Spontaneous/
42 miscarriage*.mp.
43 exp Cesarean Section/
44 (cesarean or caesarean).mp.
45 exp Obstetric Labor, Premature/
46 exp Labor, Obstetric/
47 (labor or labour).mp.
48 exp Fetal Membranes, Premature Rupture/
49 pPROM.mp.
50 or/27–49
51 26 and 50

Embase Ovid

1 exp uterine cervix tumor/
2 (cervi* and (cancer* or tumor* or tumour* or neoplas* or malignan* or carcinom*)).mp.
3 uterine cervix carcinoma in situ/
4 CIN.mp.
5 (cervi* and (intraepithel* or epithel* or dysplasia or pre-cancer* or precancer*)).mp.
6 or/1–5
7 uterine cervix conization/
8 (conisation or conization).mp.
9 low level laser therapy/
10 laser.mp.
11 exp cryotherapy/
12 cryotherapy.mp.
13 cold coagulation.mp.
14 diathermy/
15 diatherm*.mp.
16 cone biopsy.mp.
17 loop.mp.
18 LLETZ.mp.
19 LEEP.mp.
20 ablat*.mp.
21 excision*.mp.

22 transformation zone.mp.
23 (CKC or LA or LC or CC or RD or TZ).mp.
24 (conservative and (method* or treatment* or intervention* or management)).mp.
25 or/7–24
26 6 and 25
27 prematurity/
28 (preterm or premature).mp.
29 exp low birth weight/
30 birth weight.mp.
31 perinatal mortality/
32 perinatal mortality.mp.
33 newborn intensive care/
34 (neonat* and intensive care).mp.
35 female fertility/
36 fertil*.mp.
37 conception/
38 conception.mp.
39 exp pregnancy/
40 pregnancy.mp.
41 gestation*.mp.
42 spontaneous abortion/
43 miscarriage*.mp.
44 cesarean section/
45 (cesarean or caesarean).mp.
46 premature labor/
47 (labor or labour).mp.
48 premature fetus membrane rupture/
49 pPROM.mp.
50 or/27–49
51 26 and 50

CENTRAL

1 MeSH descriptor Uterine Cervical Neoplasms explode all trees
2 cervi* and (cancer* or tumor* or tumour* or neoplas* or malignan* or carcinom*)
3 MeSH descriptor Cervical Intraepithelial Neoplasia explode all trees
4 CIN
5 cervi* and (intraepithel* or epithel* or dysplasia or pre-cancer* or precancer*)
6 or/1–5
7 MeSH descriptor Conization explode all trees
8 conisation or conization
9 MeSH descriptor Laser Therapy explode all trees
10 laser
11 MeSH descriptor Cryotherapy explode all trees
12 cryotherapy
13 cold coagulation
14 MeSH descriptor Diathermy explode all trees
15 diatherm*
16 cone biopsy
17 loop
18 LLETZ
19 LEEP
20 ablat*
21 excision*
22 transformation zone
23 CKC or LA or LC or CC or RD or TZ
24 conservative and (method* or treatment* or intervention* or management)
25 or/7–24
26 6 and 25
27 MeSH descriptor Premature Birth explode all trees
28 preterm or premature

29 MeSH descriptor Infant, Low Birth Weight explode all trees
30 birth weight
31 MeSH descriptor Perinatal Mortality explode all trees
32 perinatal mortality
33 MeSH descriptor Intensive Care, Neonatal explode all trees
34 neonat* and (intensive care)
35 MeSH descriptor Fertility explode all trees
36 fertil*
37 conception
38 MeSH descriptor Pregnancy explode all trees
39 pregnancy
40 gestation*
41 MeSH descriptor Abortion, Spontaneous explode all trees
42 miscarriage*
43 MeSH descriptor Cesarean Section explode all trees
44 cesarean or caesarean
45 MeSH descriptor Obstetric Labor, Premature explode all trees
46 MeSH descriptor Labor, Obstetric explode all trees
47 labor or labour
48 MeSH descriptor Fetal Membranes, Premature Rupture explode all trees
49 pPROM
50 or/24–49
51 26 and 50

1.3. Selection Criteria

Participants

We included studies of women treated for cervical intraepithelial neoplasia (CIN), cervical glandular intraepithelial neoplasia (CGIN) or stage IA1 cervical cancer. We excluded studies where >20% of women were infected with human immunodeficiency virus (HIV), were treated during pregnancy, or were at high risk for preterm birth (i.e. women with history of preterm birth or late miscarriage, women with multiple pregnancy, or women that had conceived through assisted technology). Studies with HIV-infected women in over 20% of their population were analysed separately. We also excluded studies which recruited patients according to post-intervention variables such as margin status (e.g. recruited patients only with clear or involved margins).

Interventions

We considered studies examining excisional or ablative methods. Excisional methods were CKC, laser conisation, and LLETZ. Ablative techniques were radical diathermy, laser ablation, cold coagulation (also known as thermal ablation) and cryotherapy. Due to radical diathermy being a technique with very scarce evidence and little clinical relevance as it is rarely performed at present, in main text radical diathermy was presented only in tables and figures but not in narrative. Two additional excisional treatment methods were the needle excision of the transformation zone (NETZ) and the Fischer cone biopsy excision (FCBE), but no eligible study reported on either technique, and these were not listed in the main text. We excluded studies or subgroup of patients undergoing hysterectomy or a combination of treatments. For oncological outcomes we also excluded studies that selectively used ablative techniques for less severe CIN grades than those treated with excisional techniques, as well as studies where ablation might have been performed in women with endocervical lesions and/or unsatisfactory colposcopy, or without prior histological confirmation of the lesion.

Comparisons

In addition to the treatments listed above, for preterm birth we also included a control group of women with CIN attending for colposcopy without treatment (untreated colposcopy group). For preterm birth there were two further possible control groups: women with no history of CIN (i.e. external comparison of pregnancies after treatment to pregnancies of general population) and women with pregnancies before treatment (i.e. internal comparison of pregnancies after treatment to pregnancies before treatment). The latter two groups were only included in standard meta-analyses but not in the NMA, because they would violate the assumption of 'joint randomisability' required by the NMA model.[2]

Outcomes

We have previously published two protocols for two systematic reviews and network meta-analyses on oncological[3] and reproductive[4] outcomes after CIN treatments. In this manuscript we focused our presentation of results on the primary outcomes of these analyses with an aim to describe the trade-off between efficacy and

reproductive morbidity. Only secondary outcomes that supported our primary analyses were also presented in this manuscript. The inclusion of all secondary outcomes would not be possible due to a very large volume of data and would not be required to support the objective of this manuscript.

The primary oncological outcome in our protocol and in this manuscript (Outcome 1) was any treatment failure, defined as any (residual or recurrent) abnormal cytology (atypical squamous cells of undetermined significance [ASC-US], or worse) or any abnormal histology (CIN1 or worse).

In this manuscript we presented additional secondary oncological outcomes (also shown in the protocol) that supported our primary outcome: These included:

- Treatment failure defined as high-grade cytology (atypical squamous cells — cannot exclude high-grade squamous intraepithelial lesion [ASC-H], or worse) or high-grade histology (CIN2 or worse) (Outcome 2)
- Treatment failure defined as histologically confirmed CIN1 or worse (Outcome 3)
- Treatment failure defined as histologically confirmed CIN2 or worse (Outcome 4)
- hrHPV (high-risk human papilloma virus) positivity rates (Outcome 5)

Secondary oncological outcomes included in this manuscript but not previously shown in the protocol:

- Cervical cancer rates (Outcome 6)

Secondary oncological outcomes shown in the protocol but not included in this manuscript:

- Involved margins rates (incomplete excision of the lesion) (Outcome 7)

In our protocol we had additionally included outcomes on complications (not shown in this manuscript):

- Peri-operative or post-operative bleeding rates (Outcome 8)
- Cervical stenosis rates (Outcome 9)

High-grade cytology in Outcome 2 was defined in this manuscript as ASC-H+. This had been defined in our protocol as high-grade squamous intraepithelial lesion (HSIL), or worse. Prevalence of ASC-H is very low,[5] thus this deviation from the protocol was trivial. For Outcome 5 we considered hrHPV positivity rates at 6 months (m). If visit at 6m was not reported, we included the visit at 3–9m (whichever visit closest to 6m was reported). We also considered cervical cancer rates after different CIN treatments (Outcome 6). We previously published a separate meta-analysis on the risk of cervical cancer after CIN treatments.[6] We examined the studies from our previous meta-analysis, with an update of additional studies retrieved from our updated literature search. We explored a network meta-analysis for cervical cancer rates which was not possible (see next page for the eligibility criteria for the cervical cancer analysis).

For some studies it was possible to extract two or more cut-offs for definition of treatment failure; in this case we used the lowest possible cut-off for the main analysis to include both histologically and cytologically confirmed lesions and both low-grade and high-grade lesions if possible. Studies reporting only high-grade treatment failures, for which it was not possible to extract low-grade treatment failures, were still included in the main analysis. If cytological and histological treatment failures were reported separately but not in combination (i.e. the study did not report how many women had abnormal cytology or abnormal histology or both), then we used histology in preference to cytology in the main analysis.

The primary reproductive outcome in this manuscript and our protocol was preterm birth less than 37 weeks (w) of gestation. We presented no data on secondary reproductive outcomes in this manuscript.

Secondary reproductive outcomes shown in the protocol but not presented in this manuscript:

- Spontaneous preterm birth (less than 37w of gestation)
- Severe preterm birth (less than 32/34w of gestation)
- Extreme preterm birth (less than 28/30w of gestation)
- (Preterm) premature rupture of membranes ([p]PROM)
- Low birth weight (less than 2500 grams [g])
- Neonatal intensive care unit admission
- Perinatal mortality
- Total pregnancy rate (number of pregnancies occurring from CIN treatment until study completion)
- Rates of women requiring more than 12m to conceive
- First trimester miscarriage rates

- Second trimester miscarriage rates

Outcomes - cervical cancer analysis
Women treated for CIN remain at increased for cervical cancer for at least 20 years (y) after treatment.[6,7] In order to compare the post-treatment incidence of cervical cancer across different treatment modalities, we included only population-based studies with median (or mean) follow-up [f-u] duration of at least 5y, since studies with a shorter f-u duration might underestimate the incidence of cervical cancer after treatment. Additionally, we included studies that reported numbers of events per women-years in order to take f-u duration into account in the analysis. Finally, we included only studies that used a 'lag period' of at least 6m, i.e. we excluded cancers diagnosed during the first 6m after treatment, since cancers diagnosed shortly after treatment most likely represent missed cancers at the time of the initial treatment.

Studies
We included RCTs, quasi-RCTs and NRS with at least two arms. There were no time or language restrictions.

1.4. Data Analysis

1.4.1. Data Extraction

Data from the eligible studies were abstracted by two reviewers (AA and IK) independently using an a priori developed data collection form. We extracted the following data: author, year, country, study design including randomisation technique, inclusion/exclusion criteria, method of ascertainment of exposure and outcome, participant characteristics (median [or mean if median not reported] age, percentage of nulliparae, percentage of smokers), lesion characteristics (CIN grade, location [endocervical or ectocervical]), treatment technique, outcome measures (number of events and sample size per comparison group; for NRS we also extracted adjusted odds ratios [ORs], or risk ratios [RRs] if ORs not reported, along with 95% confidence intervals [CIs]), f-u duration, and number lost to f-u. In countries with multiple overlapping registry-based studies over the same period, we marked the largest study in order to include only this in the analysis and to avoid multiple inclusion of patients. If additional data were needed, we contacted the corresponding author up to 3 times. We contacted 6 corresponding authors; we received no replies. Several old studies did not include an email address.

1.4.2. Within-Study Risk of Bias

RoB2[8] was utilised to evaluate risk of bias (RoB) in RCTs based on the following domains: randomisation process, deviation from intended interventions, missing data, measurement of the outcome and selection of reported results. Each domain, as well as the overall study quality, was rated as 'low risk', 'some concerns' (i.e. 'moderate risk'), or 'high risk', based on the answers in the signalling questions of each domain ('Yes', 'Potentially Yes', 'Potentially No', 'No', or 'No Information'). ROBINS-I tool[9] was utilised to evaluate RoB in NRS based on the following domains: confounding, selection of participants into the study, classification of interventions, deviation from intended interventions, missing data, measurement of the outcome and selection of the reported results. Each domain, as well as the overall study quality, was rated as 'low', 'moderate', 'serious' (i.e. 'high risk'), 'critical' (i.e. 'high risk'), or 'No Information', based on the answers of the signalling questions in each domain ('Yes', 'Potentially Yes', 'Potentially No', 'No', or 'No Information'). The overall rating of studies as 'low', 'moderate', 'serious', or 'critical', coincided with the ranking of the worst-rated domain.

Methodology on how we rated each domain is reported below:

1.4.2.1. Treatment Failure

Confounding (RCTs), i.e. randomisation process
Studies where allocation to treatment was random and concealed, were assessed at low RoB. Studies where allocation to treatment was random but there was no information about concealment method, or studies without any details about randomisation process, were assessed at moderate RoB. Studies without allocation concealment (e.g. allocation according to hospital number or date of birth) were assessed at high RoB.

Confounding (NRS)
The most important confounding factors were considered to be age, smoking and CIN grade. Studies controlling for all confounding factors were assessed at low RoB, studies controlling for two out of three confounding factors were assessed at moderate RoB, and studies controlling for only one or no factor were assessed at high (i.e. serious or critical) RoB. Studies controlling for only one or no confounding factor but where allocation to treatment arms depended on treatment year, or where allocation was reported to be random for most but not all patients, were assessed at moderate RoB. In studies where authors adjusted for post-intervention variables affected by intervention (e.g. margins), we used the unadjusted data in our analysis and if this was not possible, the study was excluded.

Selection of participants (NRS)
We excluded studies where participants were selected according to post-intervention factors (e.g. margin status), and all remaining eligible studies were assessed at low RoB.

Classification of interventions (NRS)
Ascertainment of exposure was through hospital records or registries, and all studies were assessed at low RoB.

Deviation from intended interventions (RCTs and NRS)
Due to surgical intervention, blinding was not possible.
All NRS were assessed at low RoB.
RCTs where patients adhered to allocated intervention, or where only a few patients (<5) did not adhere to allocated intervention and authors used an intention-to-treat (ITT) analysis, were assessed at low RoB. Studies which did not report how many patients changed intervention, or where only a few patients (<5) did not adhere to allocated intervention and authors used a per-protocol or as-treated analysis, or where many patients (>5) did not adhere to allocated intervention and authors used an ITT analysis, were assessed at moderate RoB. Studies where many patients (>5) did not adhere to allocated intervention and authors did not use an appropriate analysis (ITT), were assessed at high RoB.

Missing data (NRS and RCTs)
RCTs with <10% missing data were assessed at low RoB. RCTs with >10% missing data were assessed at moderate or high RoB, but because we did not think that missingness depended on true value of outcome, all RCTs with >10% missing data were assessed at moderate RoB.
Generally, NRS with <10% missing data were assessed at low RoB, NRS with 10–20% missing at moderate RoB, NRS with 20–40% missing data at serious RoB, and NRS with >40% missing data at critical RoB. If missing data were additionally not balanced in treatments arms, studies were further downgraded by one scale (i.e. a study with 30% missing data which were not balanced across treatments arms, was assessed at critical RoB).

Measurement of the outcome (NRS and RCTs)
Method of ascertainment of outcome was through hospital records or registries, and most studies were assessed at low RoB. Studies where f-u might have differed across different treatments arms, were assessed at moderate RoB.

Selection of reported results (NRS and RCTs)
RCTs with a published or approved protocol prior to initiation of study, were assessed at low RoB. RCTs which did not have a protocol or did not mention whether they had a protocol, or whose protocol was published after completion of study and it was not reported when protocol was approved, were assessed at moderate RoB. Studies which presented a subgroup analysis for some but not all treatment arms, or which had some inconsistencies between described methodology in 'Materials & Methods' and 'Results' (e.g. reported f-u duration differed between 'Materials & Methods' and 'Results'), were assessed at moderate RoB. Studies which reported baseline characteristics or main results for some but not all treatments arms, or which reported results only for part of the f-u duration, were assessed at high RoB.

Overall
The worst-ranking domain determined the overall ranking.

1.4.2.2. Preterm Birth
Confounding (NRS)
In order to assess RoB due to confounding in NRS, we considered age, parity and smoking as the three most important measurable confounders and we chose the 'best' untreated comparison group in each study in this order: colposcopy (i.e. untreated women attending for colposcopy but not receiving treatment) > external (with control for confounders) > internal (i.e. pregnancies of women after treatment were compared to pregnancies of women before treatment) > external (without control for confounders). Risk was initially assessed to be moderate. Subsequently, of NRS with an external group as the 'best' control (or without an untreated group), those controlling for all aforementioned confounders remained at moderate RoB, those controlling for some of them were downgraded to serious risk, and those controlling for none of them were downgraded to critical risk. NRS with a colposcopy group as the best 'control' were assessed at no worse than moderate risk due to the fact that the choice of an untreated colposcopy group as control could eliminate immeasurable, probably more important, confounders (i.e. factors that render women vulnerable to human papilloma virus [HPV] infection

and might also be associated with obstetric outcomes); NRS with a colposcopy group that also controlled for all three most important measurable confounders (age, parity, smoking), were upgraded to low risk. NRS with an internal group as the 'best' control, were evaluated at serious RoB, since, although internal controls might eliminate inherent immeasurable confounders on the one hand, some measurable confounding factors (such as age) might have changed between post-treatment and pre-treatment pregnancies on the other hand; NRS with an internal comparison group that also controlled for age, were assessed at moderate RoB.

Confounding (RCTs), i.e. randomisation process
We downgraded quasi-RCTs, RCTs with improper randomisation process, RCTs with baseline imbalances, or RCTs with missing information on randomisation process.

Selection of participants (NRS)
All NRS were assessed at low risk.

Classification of interventions (NRS)
Studies where participants self-reported intervention, were downgraded to serious risk, and studies where participants self-reported intervention but this was subsequently confirmed from hospital records, were downgraded to moderate risk. Risk in all other studies, where confirmation of exposure was through hospital records or registries, was assessed to be low.

Deviation from intended interventions (NRS and RCTs)
All NRS were assessed at low risk. We downgraded RCTs with concerns regarding exclusion of eligible patients after randomisation.

Missing data (NRS and RCTs)
Generally, studies with <10% of missing data were assessed at low risk, studies with 10–20% of missing data were downgraded to moderate risk, studies with 20–40% of missing data were downgraded to serious risk, and studies with >40% of missing data were downgraded to critical risk.

Measurement of the outcome (NRS and RCTs)
Studies where both cases and controls self-reported outcome, were downgraded to serious risk, while studies where ascertainment of outcome was through self-reporting for cases but through hospital records or registries for controls (differential misclassification), were downgraded to critical risk. Risk in all other studies, where confirmation of outcome was through hospital records or registries, was assessed to be low.

Selection of reported results (NRS and RCTs)
NRS whose authors did not specify all the factors they adjusted for, or which reported adjusted effect estimates for some but not all treatment comparisons, were downgraded to serious risk. RCTs whose authors did not mention whether there was a protocol, were downgraded to moderate risk. Risk in all other studies was assessed to be low.

Overall
The overall ranking of studies as low, moderate, serious, or critical, coincided with the ranking of the worst-rated domain.

1.4.3. Standard Pairwise Meta-Analyses
We synthesized data in standard pairwise meta-analyses using a random-effects model when three or more studies were available for a specific comparison; for meta-analyses with only two studies, we used a fixed-effect model.[10] We calculated summary ORs and 95% CIs with the inverse variance method for fixed-effect meta-analyses, and the Hartung-Knapp-Sidik-Jonkman adjustment for random-effects meta-analyses.[11,12] In random-effects meta-analysis we calculated the between-study variance (τ^2) with the restricted maximum likelihood estimator (REML),[13,14] and its 95% CI with the Q-profile approach.[15] We also calculated the percentage of variation that can be attributed to heterogeneity rather than chance using the I^2 statistic.[16] We performed pairwise meta-analyses using the *meta*[17] package in R v4.1.3.[18]

1.4.4. Network Meta-Analyses
Network plots
For network meta-analyses we drew network plots for each outcome. The width of each line connecting two treatments was proportional to the inverse standard error of the fixed-effect summary effect size. The diameter of each node was proportional to the number of women included in this group.

Heterogeneity
To assess heterogeneity, we compared the estimated between-study variance of random effects in the network (τ^2 — assumed to be common for all treatment comparisons in the network) with Turner's empirical distribution for dichotomous data[19]; median variance for non-pharmacological treatments and semi-objective outcomes is 0·06, interquartile range (IQR)=0·00–2·35. To assess heterogeneity, we also compared prediction intervals (PIs) to CIs as part of CINeMA (see Section 1.6).

Transitivity assumption
For both outcomes we assessed potential breaches in the transitivity assumption required for NMA, by checking the distribution of potential effect modifiers across studies grouped by treatment comparison. Potential effect modifiers included publication year of study, age, smoking, CIN grade, method of ascertainment of exposure/outcome and level of income of country. In the analysis of preterm birth, we also considered parity as a potential effect modifier. We plotted the distribution of these effect modifiers across different treatment comparisons (boxplots for continuous variables, stacked bar plots for discrete variables), and visually inspected the plots for important differences.

Order of treatments
The estimated relative treatments effects from the NMA were presented in league tables and plots (the presented order of treatments was based on their presumed radicality). The order of treatments throughout the analyses was as follows:
Analyses for treatment failure: cold knife conisation (CKC) – laser conisation (LC) – radical diathermy (RD) – laser ablation (LA) – cold coagulation (CC) – cryotherapy (CT) – large loop excision of the transformation zone (LLETZ)*
Analyses for preterm birth: CKC – LC – LLETZ – RD – LA – CC – CT – Untreated Colposcopy Group (COLPO)
*In the analyses for treatment failure there was no untreated group. In these analyses LLETZ acted as the comparator and was presented last.

Design-adjusted analyses
We used a design-adjusted approach to combine randomised and non-randomised evidence[20] and considered four separate study designs (RCTs, NRS at low RoB, NRS at moderate RoB, and NRS at high RoB) and gave different weights to the different designs. The highest weight was given to RCTs and the lowest weight to NRS at high RoB. We chose arbitrary weights with the aim to explore the impact of incorporating studies with high RoB in the NMA. We conducted four design-adjusted analyses in total. In the first design-adjusted model, the variance of NRS at low RoB, NRS at moderate RoB and NRS at high RoB was inflated through division with 0·8, 0·6 and 0·4, respectively, corresponding to increasingly smaller weights. In the other three design-adjusted models the variance of NRS was further inflated by steps of 0·2 each time. The weight of RCTs remained unchanged in all models. We investigated whether the results of the design-adjusted differed from the results of the 'traditional' or 'naïve' NMA (i.e. the NMA where variances of studies remained unchanged). In the design-adjusted analyses, the amount of heterogeneity (τ^2) in the network was manually set at the same value as in the unadjusted analysis.

Subgroup analyses
For both outcomes we performed prespecified subgroup analyses according to potential effect modifiers: publication year of study, age, smoking, method of ascertainment of exposure/outcome (hospital records; region- or nation-wide registries; self-reporting), level of income of country, and lesion grade (percentage of women treated for CIN2+; percentage treated for CIN3+; percentage treated for AIS [adenocarcinoma in situ]; percentage treated for cervical cancer). For preterm birth we also performed subgroup analyses according to parity. In the analysis of treatment failure we considered the age of woman and her smoking status at the time of treatment. In the analysis of preterm birth we considered the age of woman and her smoking status at the time of pregnancy. For each discrete variable we performed analyses using all possible different values with at least two studies. For each continuous variable we first calculated the overall median for all studies in the network (e.g. for age we calculated the median of the study-specific median [or mean if median not reported] age of participants). Then, we performed a subgroup analysis, where each study was included in a group according to whether the variable was above or below the overall median. The cut-offs we used to group the studies in the network of treatment failure were: publication year: 1997; median (or mean if median not reported) participants' age: 33y; percentage of smokers: 35%; percentage of women treated for CIN2+: 89%; percentage treated for CIN3+: 58%; percentage treated for AIS: 0%; percentage treated for cervical cancer: 0%. The cut-offs we used to group the studies in the network of preterm birth were: publication year: 2011; median (or mean if median not

reported) participants' age: 30y; percentage of nulliparae: 49%; percentage of smokers: 16%; percentage of women treated for CIN2+: 83%; percentage treated for CIN3+: 61%; percentage treated for AIS: 0%; percentage treated for cervical cancer: 0%.

For treatment failure we performed further post-hoc subgroup analyses according to grade of lesion (treatment for biopsy-proven CIN2+ or persistent CIN1; treatment only for CIN3; treatment only for AIS; treatment only for stage IA1 cervical cancer; treatment for CIN1 or worse without further clarification on whether non-persistent CIN1 had been treated), location of lesion (endocervical vs ectocervical) and/or visibility of transformation zone (TZ; satisfactory vs unsatisfactory colposcopy), and LLETZ technique (top-hat LLETZ where an additional excision of the endocervical canal occurs after the main excision of the TZ, vs standard LLETZ where no additional endocervical tissue is excised). Because endocervical lesions might be more common in older women, we performed further post-hoc group analyses stratified for both location of lesion and age (i.e. ectocervical lesions and median age ≥33y; ectocervical lesions and median age <33y; endocervical lesions and age ≥33y; endocervical lesions and age <33y). For treatment failure we also performed post-hoc subgroup analyses according to f-u duration, where the analysis of the treatment failure rates throughout the study period was restricted to studies with median (or mean if median not reported) f-u duration of at least 12, 24, 36, 48 and 60m, respectively. We additionally performed an analysis of the treatment failure rates up to 6m. We chose 6m since this is usually the time point when the first f-u visit after treatment takes place. Abnormal cytology or histology at this point is more likely to represent true residual disease or recurrence, rather than acquisition of a new HPV infection. If visit at 6m was not reported, we included the visit at 3–9m (whichever visit closest to 6m was reported).

Sensitivity analyses
To examine whether the inclusion of NRS changed the results, we conducted prespecified sensitivity analyses where we excluded all NRS and NRS at high RoB, respectively. Our main analysis for preterm birth included studies regardless of whether these reported overall (i.e. both iatrogenic and spontaneous) or only spontaneous preterm birth rates. For this reason we performed a post-hoc sensitivity analysis restricted to studies reporting overall preterm birth rates.

1.5. Additional Analyses

1.5.1. Meta-Analyses of Proportions

We estimated absolute risks using the relative treatment effects derived from our NMA. Specifically, for treatment failure we first estimated the 'baseline risk' as the risk under LLETZ via a meta-analysis of proportions using the GLMM model[21] of the *meta*[17] package in R v4.1.3.[18] Then, the NMA point estimate of each relative treatment effect versus (vs) LLETZ was combined with the average baseline risk (assumed fixed). We used the same approach for preterm birth, using the risk under colposcopy group as the baseline.

1.5.2. Dose-Response Meta-Analyses

To assess the relationship between length of excised cone and risk of treatment failure or preterm birth, we performed a dose-response meta-analysis. For treatment failure we fitted a linear model where different cone lengths of excisional treatments were compared to a cone length of 5 millimetres [mm]; all excisional treatments were grouped together irrespective of the technique used. For preterm birth we performed two separate dose-response meta-analyses where different cone lengths were compared to the untreated colposcopy and external group, respectively, using restricted cubic splines. We used splines for treatment failure but a linear model for preterm birth due to data restrictions in treatment failure (see Section 2.13). Dose-response meta-analyses were performed using the *dosresmeta*[22] package in R v4.1.3.[18]

1.6. Assessment of the Credibility of Evidence (CINeMA)

We used the online software CINeMA (http://cinema.ispm.ch/) to assess the credibility of evidence.[23] CINeMA consists of the following six domains:

Within-study bias
We downgraded within-study bias to 'some concerns' if most of the evidence was at moderate risk, and to 'major concerns' if the majority was at high RoB.

Reporting bias, small-study effects, publication bias
This domain was evaluated separately with the ROB-MEN tool, which assessed for within- and across-study reporting bias as well as for small-study effects.[24] For pairwise comparisons with at least ten studies, we visually inspected contour-enhanced funnel plots for asymmetry and we also applied Egger's test[25] of the *metafor*[26] package in R v4.1.3.[18]

Indirectness
All studies were assessed as relevant to our research question (risk of treatment failure or reproductive morbidity after treatment for CIN), and we did not downgrade any studies.

Imprecision
To assess inconsistence, we examined CIs. We considered OR<0·80 or >1·25 to be clinically important (i.e. 0·80–1·25 to be the range of equivalence), and we used the defaults of the online software.

Heterogeneity
To assess heterogeneity, we compared PIs to CIs. We considered 0·80–1·25 to be the range of equivalence, and we used the defaults of the online software.

Incoherence
To assess incoherence, we used the global test[27] for comparisons with only direct or indirect evidence, and the local test[28] for comparisons with mixed (i.e. both direct and indirect) evidence. For comparisons with mixed evidence, we examined whether direct and indirect evidence differed (we considered 0·80–1·25 to be the range of equivalence). We used the defaults of the online software.

Overall
The overall confidence in the evidence for each comparison was assessed as 'very low', 'low', 'moderate' or 'high'. Each comparison was initially assessed to be of high quality and was downgraded by one level for each domain with 'some concerns' and by two levels for each domain with 'major concerns'. Because 'imprecision', 'heterogeneity' and 'incoherence' are interconnected, we considered only the worst-rated of these three domains and we downgraded only once.

2. Supplementary Results

2.1. Characteristics of Studies

2.1.1. Treatment Failure

The search for oncological outcomes yielded 7,880 records. After screening, there were 81 studies reporting on treatment failure rates. Of these, 46[29-74] were retrospective and seven[75-81] were prospective cohorts, 27[82-107] were RCTs or quasi-RCTs (one report[104] consisted of two separate cohorts and was treated as two separate studies), and one[108] was a pooled analysis of two RCTs and one prospective cohort. Characteristics of studies are reported in the table below (Table 2.1.1.1). The main network for treatment failure (defined as cytological ASC-US+ or histological CIN1+) included 19,234 women in 71 eligible studies. In the network there were 38[30-44,46-66,70,72] retrospective and seven[75-81] prospective cohorts, 25[82-104,107] (quasi-) RCTs, and one[108] pooled analysis. The network included women treated with CKC, LC, RD, LA, CC, CT, or LLETZ; no studies reported on NETZ or FCBE. The median of the median f-u duration was 15m (IQR=9–35), although this is possibly an underestimate of the true median because nine studies reported only the minimum follow-up duration, and so we assumed that median was equal to the minimum in these studies. The median of the median age at treatment across studies was 33y (IQR=30–36), while the median of the percentage treated for CIN2+, CIN3+, AIS, and cervical cancer was 89% (IQR=72–100), 58% (IQR=41–87), 0% (IQR=0–0), and 0% (IQR=0–1), respectively.

Table 2.1.1.1: Characteristics of studies reporting on risk of CIN treatment failure

Study (Country)	Study Design	Procedure (N treated)	Indications for treatment & immune status (if reported)	Outcomes	F-u duration	F-u scheme	Definition of treatment failure
Wright 1981 (Canada)†	Retrospective cohort	LA (131); CT (152)	Biopsy-proven CIN, with agreement between cytology/colposcopy/histology; endocervical lesions were excluded	Treatment failure (9m, 12m, overall)	12-42m	Cytology and colposcopy at 3m and every 6m thereafter	Histological CIN
Townsend 1983 (USA)†	Quasi-RCT	LA (100); CT (100)	Biopsy-proven CIN with satisfactory colposcopy and negative ECC	Treatment failure (overall)	≥1y	Cytology and colposcopy at 3m and 6m; if both negative, every 6m thereafter until termination of study; if abnormal, PB and ECC	Not reported
Jobson 1984 (USA)†	(quasi-) RCT	LA (42); CT (39)	Biopsy-proven CIN with satisfactory colposcopy and negative ECC	Treatment failure (4m)	12–24m (patients with less than 12m f-u were excluded)	Cytology and colposcopy at 4m, 8m, 12m, 18m and 24m (PB if indicated)	Not reported
Lele 1984 (USA)†	Retrospective cohort	CKC (25); CT (35)	CT: satisfactory colposcopy, ectocervical lesion, correlation within one degree between cytology and histology, and normal ECC; CKC: disparity between cytology and colposcopy, unsatisfactory colposcopy, or abnormal ECC	Treatment failure (3m, overall)	Mean: 35m	Cytology at 3m; intervals thereafter not reported	Abnormal cytology
Ferenczy 1985 (Canada)†	Prospective cohort	LA (147); CT (147)	Biopsy-proven CIN with or without limited extension (up to 5mm) into endocervical canal, and negative ECC; alternate treatment with CT or LA; matching for CIN grade, lesion size and distribution (endocervix or ectocervix)	Treatment failure (12m)	12m	Cytology and colposcopy every 4m for 12m	Histological CIN
Helmerhorst 1985 (the Netherlands)†	(quasi-) RCT	LA (85); CT (81)	Biopsy-proven CIN; patients with endocervical lesions or spontaneous remission after PB were excluded	Treatment failure (overall)	Median: 26m (range: 3–48m)	Cytology and colposcopy at 3m, 6m, 9m, 12m, 18m and annual thereafter	Cytological or colposcopic CIN
Hussein 1985 (UK)†	Retrospective cohort	CKC (92); RD (69); CC (65)	Biopsy-proven CIN or cancer IA1; RD/CC only if satisfactory colposcopy without suspicion of (micro-)invasion	Treatment failure (4m)	Up to 2y	Cytology every 4m; colposcopy at 4m was performed for all women after CC, mostly for extensive lesions after RD, and for incomplete excisions after CKC	Cytological or colposcopic CIN
Kirwan 1985 (UK)†	(quasi-) RCT	LA (71); CT (35)	Biopsy-proven CIN3 with satisfactory colposcopy	Treatment failure (overall)	17–24m	Cytology and colposcopy at 4m and 10m (PB of lesions); annual cytology thereafter	Cytological or histological CIN

Kwikkel 1985 (the Netherlands)†	(quasi-) RCT	LA (53); CT (52)	Biopsy-proven CIN with satisfactory colposcopy and fully visible lesion; patients with spontaneous (cytological and colposcopic) remission after PB were excluded	Treatment failure (overall)	9–18m	Cytology and colposcopy at 3w and every 3m thereafter until 18m (PB if needed)	two or more abnormal smears or one abnormal PB
Seshadri 1985 (Australia)†	Retrospective cohort	CKC (592); RD (153)	Histologically confirmed CIN3; RD only if fully visible lesion	Treatment failure (overall)	1–10y	F-u at 6w; annual cytology thereafter; in CKC group, 117/592 underwent subsequent hysterectomy: 81 due to positive or uncertain margins, and 36 due to age >40y (without wish for fertility) or other reasons; in LLETZ group, 12/153 underwent subsequent hysterectomy (reasons not reported), and 31/141 underwent subsequent CKC because of abnormal cytology	Histological CIN3+
Baggish 1986 (USA)†	Retrospective cohort	LC (220); LA (100)	Biopsy-proven CIN; LA only for ectocervical lesions with cytology-histology agreement and no suspicion of invasion; LC could be performed in any case	Treatment failure (overall)	1–3y	Cytology and colposcopy at 3m and every 6m thereafter	Treatment cure was defined as normal cytology and colposcopy for at least 1y; treatment failure was probably defined as abnormal cytology or colposcopy
Bostofte 1986 (Denmark)†	(quasi-) RCT	CKC (64); LC (59)	Not reported (however, all patients had biopsy-proven CIN1–3, except for one patient with normal PB and abnormal cytology)	Treatment failure (overall)	Mean: 36m (28–48m)	Cytology and colposcopy at 3w and 3m; cytology every 6m for 2y thereafter; annual cytology thereafter (for up to 5y in total)	Not reported
O'Shea 1986 (Australia)†	Quasi-RCT	RD (27); CT (30)	Biopsy-proven CIN with satisfactory colposcopy	Treatment failure (overall)	≥36m	Cytology and colposcopy at 3m; cytology at 6m and 12m; if previous tests normal, annual cytology thereafter	Not reported
Need 1988 (Australia)†	Quasi-RCT	RD (28); LA (33)	Biopsy-proven CIN with satisfactory colposcopy	Treatment failure (overall)	18–42m	Cytology and colposcopy at 4m; cytology at 8m and 12m, 18m and 24m; if previous tests normal, annual cytology thereafter	Not reported
Singh 1988 (Singapore)†	Quasi-RCT	CC (91); CT (67)	Biopsy-proven CIN with satisfactory colposcopy	Treatment failure (overall)	3m–4y	Cytology and colposcopy at 3m, 6m and every 6m thereafter; discharge to GP for annual smears after 2y, but some patients preferred to continue f-u in the clinic	Not reported
Partington 1989 (UK)†	RCT	LC (50); LA (50)	Biopsy-proven CIN1–3 and satisfactory colposcopy with lesion extending less than 5mm intro endocervical canal	Treatment failure (overall)	Mean: 9·3m (2–17m)	Cytology/colposcopy at 2-3m, 6m, 12m and 24m	Cytological or histological CIN; histological CIN
Yliskoski 1989 (Finland)†	Prospective cohort & quasi-RCT	LA (77); CT (42)	Women with biopsy-proven HPV or CIN1–2, satisfactory colposcopy and agreement between cytology/colposcopy/histology were randomised (by birth date) to LA or CT; women with HPV/CIN plus VaIN were treated with LA	Treatment failure (overall)	Mean 14m	Cytology and colposcopy at 4m and every 6m thereafter (PB if needed)	Histological CIN; histological CIN2+

Gunasekera 1990 (UK)†	Quasi-RCT	LLETZ* (98); LA (101)<br><br>*3-6 passes were needed to excise TZ	Biopsy-proven CIN2–3 with satisfactory colposcopy	Treatment failure (3m and 6m)	6m	Cytology and colposcopy at 3m and 6m (PB if needed)	Not reported
Hellberg 1990 (Sweden)†	Retrospective cohort	CKC (628); CT (104)	Biopsy-proven CIN1–3; CKC was the routine treatment until 1975, but from 1975 onwards, patients <35y with normal ECC were treated with CT	Treatment failure (1y, 2y, 5y, 10y, 15y, overall)	Mean: 10·3y (6m up to more than 20y)	Cytology and colposcopy every 6m for 2y, and annually thereafter	Biopsy-proven CIN; biopsy-proven CIN2+
Tabor 1990 (Denmark)†	Retrospective cohort	CKC (201); LC (224)	Biopsy-proven CIN1–3, with unsatisfactory colposcopy or abnormal ECC	Treatment failure (3m, 9m, 15m, 21m, overall)	Median: 68m (range: 35–93)	Cytology at 3m, 9m, 15m and 21m; colposcopy at 21m; ECC and PBs in case of abnormal cytology; discharge to GP for annual f-u after 21m	Abnormal cytology; biopsy-proven CIN
Berget 1991 (Denmark)†	(quasi-) RCT	LA (103); CT (101)	Biopsy-proven CIN1 at ≥2 PBs taken at least 3-6m apart; biopsy-proven CIN2 at a single PB; biopsy-proven CIN3 at a single PB with extension into crypts of no more than 3mm; patients with unsatisfactory colposcopy, extension of CIN of more than 12·5mm from the orifice or extension of CIN to vagina were excluded	Treatment failure (3m, 9m, 15m, 21m, 33m, 45m, 80m, overall)	Mean: 50m	Cytology and colposcopy at 3m, 9m, 15m, 21m and annual thereafter (PB if needed)	Histological CIN
Goodman 1991 (UK)†	Quasi-RCT	LA (77); CC (78)	Biopsy-proven CIN; according to British clinical practice, ablation was to be used only in case of satisfactory colposcopy	Treatment failure (4m)	4m	Cytology at 4m	Abnormal cytology
Martel 1992 (France)†	Retrospective cohort	LC (59); LA (25)	Biopsy-proven CIN3	Treatment failure (12m, overall)	Mean: 25m	Cytology and colposcopy every 4m during the first year, every 6m during the second year, and annually thereafter; additional f-u at 1m if incomplete excision	Cytological, histological or colposcopic CIN (no other details)
Guijon 1993 (Canada)†	Prospective cohort	LA (160); CT (276)	Biopsy-proven CIN with satisfactory colposcopy and agreement between cytology/histology/colposcopy	Treatment failure (overall)	Up to 4y	Cytology and colposcopy every 4-6m for up to 4y (PB of lesions)	Biopsy-proven CIN; biopsy-proven CIN2+
Oyesanya 1993 (cohort study) (UK)†	Retrospective cohort	CKC (43); LLETZ* (43)<br><br>*excision in one piece	Moderate, severe or recurrent mild cytology, with unsatisfactory colposcopy	Treatment failure (12m)	12m	Cytology and colposcopy at 4m, 8m and 12 (PB of lesions)	Histological CIN
Oyesanya 1993 (RCT) (UK)†	RCT	LLETZ* (150); LC (150)<br><br>*in 8% excision was in more than one piece	Biopsy-proven CIN (upper limit of colposcopy was visible)	Treatment failure (12m)	12m	Cytology and colposcopy every 3m for 1y (PB if needed)	CIN1+; CIN2+ (not reported whether these refer to histological CIN)
Alvarez 1994 (USA)†	RCT	LLETZ (195); LA (180)	Cytologic HSIL or persistent (≥2) ASC-US/LSIL, with satisfactory colposcopy; PBs were not taken from patients randomised to LLETZ; PBs were taken from patients randomised to LA and treatment was performed only when indicated based on PB findings	Treatment failure (3m, 6m)	6m	Cytology and pelvic examination at 3m and 6m	Abnormal cytology
Kuppers 1994 (Germany)‡	Retrospective cohort	LC (6); LA (4)	Biopsy-proven CIN1–3; LA only for ectocervical lesions; LC for ectocervical or endocervical lesions<br><br>Only women with HIV were included; 60% received antiretroviral therapy (zidovudine); 50% had <200 CD4 cells per mcL	Treatment failure (overall)	1–3y	Regular cytology and colposcopy; the frequency of f-u intervals depended on CIN grade and count of CD4 cells	Not reported
Sideri 1994 (Italy)†	Retrospective cohort	CKC (50); LLETZ (124)	Biopsy-proven CIN3 (CKC was used more commonly in women with unsatisfactory colposcopy than LLETZ)	Treatment failure (overall)	Median: 35m	Cytology and colposcopy at 2m, 4m and every 6m for the first 2y thereafter (PB if needed)	Abnormal cytology; histological CIN

Diakomanolis 1995 (Greece)†	Retrospective cohort	LC (85); LA (228)	Biopsy-proven CIN1; LA if satisfactory colposcopy and no suspicion of invasion; LC if unsatisfactory colposcopy or discrepancy between cytology/colposcopy/histology	Treatment failure (12m, overall)	Mean: 57m (42–76m)	Cytology and colposcopy every 3m for the first year and every 6m thereafter (PB if needed)	Not reported
Baldauf 1996 (France)†	Retrospective cohort	LC (255); LLETZ* (277)<br><br>*top-hat LLETZ when endocervical limit was not visible	LC until 1992: biopsy-proven CIN2–3, or CIN1 with unsatisfactory colposcopy; LLETZ after 1992: biopsy-proven CIN1-3, with satisfactory or unsatisfactory colposcopy	Treatment failure (overall)	Mean: 26·5m	Cytology and colposcopy at 3-6m, 12m and annually thereafter for 4y; PB in case of abnormal colposcopy; ECC in case of abnormal cytology; referral to GP/gynaecologist after 4y for annual cytology	Histological CIN (on PB or ECC)
Santos 1996 (Peru)†	(quasi-) RCT	LC (145); LLETZ* (149)<br><br>*multiple passes (including for the endocervical canal) were needed in most cases	Suspicion of CIN based on cytology and colposcopy regardless of TZ type; women with extensive lesion were excluded	Treatment failure (overall)	Mean: 335 days	Cytology and colposcopy at 3m, 6m, 9m, 12m, 16m, 20m and 24m (PB if needed)	Not reported
Urbaniak 1996 (New Zealand)†	Retrospective cohort	LC (158); LLETZ (333)	Biopsy-proven or cytological/colposcopic CIN	Treatment failure (4-6m)	4–6m	Cytology at 4-6m	Abnormal cytology; cytological LSIL+; cytological HSIL
Varawalla 1996 (UK)†	Retrospective cohort	LLETZ (200); LA (200); CT (191)	Histological CIN (on PB for CT/LA; on cone for LLETZ); according to British clinical practice, ablation was to be used only in case of satisfactory colposcopy	Treatment failure (4m, 10m, 24m, 36m, 48m, 60m, overall)	Mean: 3·8y	Cytology at 4m and 10m in colposcopy clinic and annually thereafter in GP practice for up to 5y; if abnormal, colposcopy and PB; discharge to genera population screening after 5y	Histological CIN
Widrich 1996 (USA)†	Retrospective cohort	CKC (25); LC (3); LLETZ* (18)<br><br>*top-hat LLETZ	AIS	Treatment failure (overall)	Mean: 54·9m (3–177m)	11/46 and 6/46 underwent hysterectomy and repeat conisation, respectively; no other details reported	AIS+
Wolf 1996 (USA)†	Retrospective cohort	CKC (47); LLETZ (7); LC (1)	AIS, alone or with CIN	Treatment failure (overall)	Median: 57m (range: 17–132m)	most patients (44/55) underwent subsequent hysterectomy; no other details reported	Histological CGIN+
Gonzalez-Bosquet 1997 (Spain)†	Prospective cohort	CKC (25); LLETZ (58); LA (40)	Biopsy-proven CIN1–3, with satisfactory colposcopy; patients with CIN1–2 received LLETZ or LA; patients with CIN3 received LLETZ, LA or CKC	Treatment failure (overall)	Mean: 15m	Cytology and colposcopy every 3m for at least a year (PB if needed)	Histological CIN; histological CIN2+
Mitchell 1998 (USA)†	RCT	LLETZ* (130); LA (121); CT (139)<br><br>*one pass if diameter of cervix less than 4 cm; two passes if diameter of cervix more than 4cm	Biopsy-proven CIN with satisfactory colposcopy, negative ECC and agreement between cytology and histology	Treatment failure (6m, overall)	Median: 15·1m; mean: 16m (6–37m)	Cytology and colposcopy at 1m, 4m, 8m, 12m, 16m, 20m and 24m (PB of lesions)	Histological CIN
Simmons 1998 (USA)†	Retrospective cohort	CKC (50); LLETZ* (45)<br><br>*one or more passes; authors could not determine if any passes involved the endocervical canal	Discrepancy between cytology and histology; unsatisfactory colposcopy; abnormal ECC	Treatment failure (overall)	Median: 11m (range: 1–47m) for LLETZ; median: 9m (range: 3–56m) for CKC	Cytology (intervals not reported)	Abnormal cytology; cytological LSIL+; cytological HSIL

Bornstein 1999 (Israel)†	Retrospective cohort	CKC (22); LLETZ (52); LA (13)	Cytological HSIL (including women with unsatisfactory colposcopy)	Treatment failure (overall)	Not reported	Cytology and colposcopy at 3m, 6m and 12m (PB/ECC if needed); annual f-u thereafter	Not reported
Duggan 1999 (USA)†	RCT	CKC (89); LLETZ* (91)<br><br>*top-hat LLETZ	Biopsy-proven CIN with unsatisfactory colposcopy; abnormal ECC; suspicion of Ca on the basis of cytology/colposcopy but unconfirmed by PB; two-grade discrepancy between cytology and histology<br><br>Women with HIV were excluded	Treatment failure (12m)	12m (mean: 10·7m)	Cytology and colposcopy at 3m, 6m and 12m (PB/ECC if needed); women with positive margins were advised to undergo repeat conisation or hysterectomy	Not reported
Giacalone 1999 (France)†	RCT	CKC (38); LLETZ* (28)<br><br>*the size of the loop was chosen to ensure the excision of the lesion in one piece	Biopsy-proven CIN3; biopsy-proven CIN2 with unsatisfactory colposcopy	Treatment failure (3m)	3m	Cytology and colposcopy at 3m (PB if needed)	Histological CIN
Ioffe 1999 (USA)†	Retrospective cohort	CKC (24); LLETZ* (76)<br><br>*in 57% multiple fragments	Cytological or histological CIN for most cases	Treatment failure (overall)	29–40m	Not reported	Cytological or histological CIN
Takac 1999 (Slovenia)†	(quasi-) RCT	CKC (120); LLETZ (120)	Biopsy-proven CIN2–3; biopsy-proven persistent CIN1; discrepancy between cytology and histology (including women with unsatisfactory colposcopy)	Treatment failure (3m)	3m	In Methods authors report that cytology and, if abnormal, colposcopy/PB and ECC, were performed at 3m. However, in Results authors report that colposcopy and ECC were performed in all patients with involved margins, regardless of cytology result.	Not reported
Vejerslev_A 1999 (Denmark)†	RCT	LC (55); LLETZ* (67)<br><br>*larger lesions required two or more passes	Biopsy-proven CIN2–3; biopsy-proven persistent CIN1	Treatment failure (3m, 9m)	9m	Cytology and colposcopy at 3m and 9m	Abnormal cytology
Vejerslev_B 1999 (Denmark)†	RCT	LC (51); LLETZ* (49)<br><br>*larger lesions required two or more passes	Biopsy-proven CIN2–3; biopsy-proven persistent CIN1	Treatment failure (3m, 6m)	6m	Cytology and colposcopy at 3m and 6m	Abnormal cytology
Husseinzadeh 2000 (USA)†	Retrospective cohort	CKC (60); LLETZ* (77)<br><br>*top-hat LLETZ	Abnormal ECC; unsatisfactory colposcopy; suspicion of invasion; discrepancy between cytology and histology (by two or more grades)	Treatment failure (3m, 6m)	6m	Cytology and colposcopy every 3-4m	Positive endocervical margin and abnormal ECC (residual disease); abnormal cytology (recurrent disease)
Persad 2001 (Canada)†	Retrospective cohort	LA (1126); CT (1114)	Biopsy-proven CIN, satisfactory colposcopy and agreement between cytology, colposcopy and histology; women with abnormal ECC were excluded; CT mostly for CIN1 and LA mostly for CIN2/3	Treatment failure (overall)	Median: 60m	Cytology and colposcopy at 3m, then every 6m for 3 visits and once a year thereafter	CIN1+; CIN2+ (not reported whether these refer to histological CIN)
Dey 2002 (UK)†	RCT	LLETZ (155); LA (134)	Satisfactory colposcopy without suspicion of invasion; PB in all but one woman treated with LA and in 101/155 women treated with LLETZ	Treatment failure (overall)	Median number of adequate smears: 4 (median f-u: ~42m)	Cytology and colposcopy at 6m; if normal, discharge and annual smears	Abnormal cytology; cytological LSIL+; cytological HSIL

Mathevet 2003 (France)†	RCT	CKC (37); LC (37); LLETZ* (36)<br><br>*excision in one piece	Cytological and histological HSIL; cytological and histological LSIL with unsatisfactory colposcopy	Treatment failure (2m, overall)	Mean: 65m (38–118m); patients with f-u <36m were excluded for overall treatment failure	Cytology and colposcopy at 2m and 6m (PB of suspicious areas); cytology and colposcopy at regular intervals in the department or private practitioner thereafter (no other details)	Not reported
Omnes 2003 (France)†	Retrospective cohort	CKC (5); LLETZ (3)	Histological AIS, alone or with CIN	Treatment failure (overall)	Median: 49m; mean: 96m (12–286m)	Not reported; 2/8 received a second procedure	AIS+
Zielinski 2003 (the Netherlands)†	Prospective cohort	CKC (23); LLETZ (85)	Histologically confirmed CIN3	Treatment failure (overall)	Median: 29m (range: 2–65m)	Cytology at 3m, 6m, 12m and 24m; annually thereafter until two consecutive normal smears; HPV DNA test at 3m	Histological CIN2+
Murta 2004 (Brazil)†	Retrospective cohort	CKC (245); LLETZ (102)	Biopsy-proven CIN2–3; LLETZ when small lesion, satisfactory colposcopy and wish for fertility	Treatment failure (overall)	≥2y	Cytology and colposcopy every 6m for 5y and annually thereafter	Not reported
Lu 2006 (China)§	Retrospective cohort	NETZ/LLETZ (449)	Women underwent treatment due to biopsy-proven CIN2+, discrepancy of more than two grades between cytology, histology or colposcopy, or suspicion of (micro-)invasion; only women with confirmed CIN3 on excised cone were included	Treatment failure (overall)	Up to 2y	Cytology every 3m for 2y; colposcopy/PB if abnormal cytology	Histological CIN
Dalrymple 2008 (Australia)†	Retrospective cohort	CKC (38); LC (44)	AIS on cytology or excised cone, alone or with CIN/CIS	Treatment failure (overall)	Mean: 5·9y (1–10y)	Patients with positive margins received second procedure (CKC: 6/8; LC: 5/6); no other details reported	Histological AIS+; histological AIS+ or CIN2+; histological AIS+ or CIN; histological or cytological CIN/AIS+
Park 2008 (South Korea)†¶	Retrospective cohort	CKC (77); LLETZ (159)	CIN on excised cone	Treatment failure (overall); hrHPV HPV DNA test (performed at 3-6m after treatment)	Median: 15m; mean: 17m (6–56m)	Cytology and HPV DNA test between 3m and 6m; subsequent f-u was individualised and depended on f-u results and severity of the dysplasia	Histological CIN; histological CIN2+
Gallwas 2010 (Germany)¶	Retrospective cohort	CKC (20); LLETZ (87)	Biopsy-proven CIN2–3; Pap test III or IV	hrHPV DNA test (performed at 4·8m after treatment on average)	Mean: 4·8m	–	–
Ostojic 2010 (Croatia)†	Retrospective cohort	CKC (151); LLETZ (110)	Abnormal cytology and colposcopy	Treatment failure (24m)	Up to 2y	All patients had at least 3 smears or repeat treatment within 2y; HPV DNA test was also performed (time point not reported)	Histological CIN; histological CIN2+
Ang 2011 (UK)§	Retrospective cohort	LLETZ (1558)	CIN2–3 on cone	Treatment failure (overall)	Median: 77m	F-u was according to local and national guidelines; if abnormal cytology, colposcopy (PB if needed)	Histological CIN
Kocken 2011 (the Netherlands)†	Pooled analysis of two RCTs and one prospective cohort	LLETZ (358); CKC (77)	CIN2–3	Treatment failure (5y, 10y)	Mean: 96m	Cytology and HPV DNA test at 6m, 12m and 24m; colposcopy if any abnormal (PB of lesions); screening as per national guidelines after discharge (i.e. once every 5y); all women were invited for additional cytology and HPV DNA test in 2009	Histological CIN2+

Kietpeerakool 2012 (Thailand)†	Retrospective cohort	CKC (23); LLETZ (37)	AIS on excised cone, alone or with LSIL/HSIL	Treatment failure (overall)	Median f-u for those without repeat treatment: 60m (range: 10–144m); median f-u for those with repeat treatment: 6w	54/60 received a second procedure and 6/60 continued f-u with cytology	Histological AIS or CIN2+
Serati 2012 (Italy)†	Retrospective cohort	CKC (68); LLETZ (214)	Biopsy-proven CIN2–3 or persistent (>2y) CIN1; suspicion of micro-invasion; suspicion of CGIN in the absence of endometrial pathology<br><br>2·5% were immunocompromised (1·4% due to HIV infection and 1·1% due to treatment with corticosteroids)	Treatment failure (overall)	Median: 26·7m (range: 6–100m)	CIN1: cytology and colposcopy every 6m for at least 2y; CIN2/3: cytology and colposcopy at 3m and then every 6m for at least 5y; micro-invasion: strict f-u or hysterectomy when no wish for fertility	Histological CIN
Van Hanegem 2012 (the Netherlands)†	Retrospective cohort	CKC (58); LLETZ* (54)<br><br>*excision in one piece	AIS on cytology, PB or excised cone; selection of treatment was based on size of cervix/TZ/lesion (LLETZ when lesions were small enough to enable LLETZ in one pass)	Treatment failure (overall)	Mean: 32m	Negative margins: cytology every 3-4m until at least 4 normal smears and annually thereafter; ECC in case of insufficient endocervical sample or cervical stenosis; HPV DNA test became part of post-treatment f-u during the last years of study; Women with positive margins underwent a second procedure	AIS+
Zeng 2012 (China)†	Retrospective cohort	CKC (869); LLETZ (74)	Biopsy-proven CIN; women without CIN on excised cone were excluded	Treatment failure (overall)	Mean: 29m (12–78m)	1st year: pelvic examination/cytology every 3m, colposcopy every 6m, HPV DNA test at 8-12m; 2nd year onwards: annual cytology and colposcopy	Histological CIN; histological CIN2+
Taylor 2014 (USA)†	Retrospective cohort	CKC (33); LLETZ (15)	Biopsy-proven AIS (confirmation with positive p16 and negative progesterone immunostaining for difficult cases)	Treatment failure (overall)	Mean: 32m (1·3–146m)	Cytology/ECC at varying intervals; some patients underwent repeat treatment: 8 out of 16 with positive margins after CKC); 2 out of 17 with negative margins after CKC; 4 out of 6 with positive margins after LLETZ; 3 out of 9 with negative margins after LLETZ	Histological AIS+
Babkina 2015 (USA)‡	Retrospective cohort	CKC (17); LLETZ (27)	Biopsy-proven CIN2–3<br><br>Only women with HIV were included; authors do not report how many women received antiretroviral therapy or had <200 CD4 cells per mcL	Treatment failure (6m)	6m	Not reported	Biopsy-proven CIN2+
Cai 2015 (China)†	Retrospective cohort	CKC (51); LLETZ (64)	Biopsy-proven CIN1–3	Treatment failure (6m)	6m	Cytology at 6m	Abnormal cytology
Kiuchi 2016 (Japan)†	Retrospective cohort	LC (405); LLETZ* (146)<br><br>*two or more passes when the ectocervical lesion was very wide	CIN or cancer IA1	Treatment failure (12m)	12m	Positive endocervical margin: cytology and colposcopy at 6-8w, 3m, 6m and 12m (PB of lesions); negative endocervical margin: not reported	Histological CIN

Mariya 2016 (Japan)†	Prospective cohort	LC (101); LA (137)	CIN3; LA was performed when no discrepancy between cytology and histology and satisfactory colposcopy; LC was performed when unsatisfactory colposcopy, discrepancy between cytology and histology, or no wish for fertility	Treatment failure (2m, 5m, overall)	≥5m	Cytology and HPV DNA test at 2m, 5m and every 6m thereafter until at least 2y of recurrence-free observation; after at least 2y of recurrence-free survival, f-u was extended for one more year; if abnormal cytology at any visit, colposcopy and PB were performed	Histological CIN2+
Hansen 2017 (Germany)†¶	Retrospective cohort	LLETZ (153); LA (113)	Biopsy-proven CIN or cancer IA1; according to German guidelines, ablation was to be used only in case of satisfactory colposcopy	Treatment failure (at the time of ToC*, overall); hrHPV DNA test (at the time of ToC*)<br><br>*time point of ToC not reported	Median: 25·5m (range: 1–44m)	Cytology, HPV DNA test, colposcopy/PB	Abnormal cytology; histological CIN; histological CIN2+
Papoutsis 2017 (UK)†	Retrospective cohort	LLETZ* (233); CC (178)<br><br>*in 29% excision was in more than one piece	Biopsy-proven CIN2–3; patients with CGIN were excluded	Treatment failure (6m)	12m	Cytology at 6m in colposcopy clinic; cytology at 12m in primary care setting	Cytological LSIL+; cytological HSIL
Smith 2017 (South Africa)‡	RCT	LLETZ (86); CT (80)	Biopsy-proven CIN2–3; patients with endocervical lesions were excluded<br><br>Only women with HIV were included; 90% received combined antiretroviral therapy; 13% had <200 CD4 cells per mcL	Treatment failure (6m, 12m)	12m	Cytology, HPV DNA test, visual inspection with acetic acid, and colposcopy with biopsies at 6 and 12m; if colposcopy was normal, biopsies were taken at 6 and 12 o' clock	Biopsy-proven CIN1+; biopsy-proven CIN2+; cytological LSIL+; cytological HSIL+
Wyse 2017 (Ireland)†¶	Prospective cohort	LLETZ (200); CC (200)	Biopsy-proven CIN2–3 or persistent CIN1	Treatment failure (6m); hrHPV DNA test (6m)	6m	Cytology and HPV DNA test at 6m	Abnormal cytology; cytological LSIL+; cytological HSIL
Byun 2018 (South Korea)†	Retrospective cohort	CKC (90); LLETZ (82)	CIN2–3	Treatment failure (overall)	Mean: 34m	Cytology every 3m during first year and annually thereafter; HPV testing every 6m during the first year and annually thereafter	Histological CIN2+
Greene 2019 (Kenya)‡	RCT	LLETZ (200); CT (200)	Biopsy proven CIN2–3; patients with endocervical lesions were excluded<br><br>Only women with HIV were included; 97% received antiretroviral therapy; 30% had <200 CD4 cells per mcL	Treatment failure (24m)	24m	Cytology at 6m, 12m, 18m and 24m; if <HSIL, women were scheduled for next f-u visit; if ≥HSIL, women underwent PB and LLETZ	Biopsy-proven CIN2+; cytological HSIL+
Bogani 2020 (Italy)†¶	Retrospective cohort	LC (567; propensity-matched cohort: 500); LLETZ (2399; propensity-matched cohort: 2399)	HSIL/CIN2–3; patients with glandular lesions were excluded	Treatment failure (overall); hrHPV DNA test (6m)	5y	F-u (cytology +/- colposcopy +/- PB every 6m for the first 2y and annually thereafter until 5y. First colposcopy was performed at 6m if margins were negative and at 3m if margins were positive. HPV DNA testing was usually performed at 6m.	New HSIL/CIN2+ requiring secondary conisation or hysterectomy
Lara-Penaranda 2020 (Spain)§	Retrospective cohort	LLETZ* (256)<br><br>*top-hat LLETZ in case of TZ type 2 or 3	Biopsy-proven CIN2–3; patients with cyto-histological discordance were excluded	Treatment failure (6m)	12–18m	F-u at 6m and 12-18m	Abnormal cytology

Sun 2020 (China)†	Retrospective cohort	CKC (22); LLETZ* (107)<br><br>*top-hat LLETZ	Biopsy-proven CIN2–3; post-menopausal women with confirmed CIN3 on cone specimen proceeded with hysterectomy after conisation within ≤6m	Treatment failure (overall)	≤6m	Hysterectomy was performed within ≤6m after conisation	Residual CIN2+ on hysterectomy specimen
Duan 2021 (China)†¶	RCT	CC (74); CT (75)	Biopsy-proven CIN2–3 with satisfactory colposcopy	Treatment failure (4m, 8m); hrHPV DNA test (4m, 6m*)<br><br>*Because authors did not report cumulative rates at 6m, we used 4m as our time point in the analysis	8m	Cytology and HPV DNA test at 4 and 6m; colposcopy +/- PB if abnormal cytology or positive HPV DNA test	Biopsy-proven CIN2+
Zang 2021 (China)¶	Retrospective cohort	CKC (414); LLETZ (136)	CIN1 persisting for ≥2y, or CIN2–3 (colposcopic diagnosis); only women with confirmed CIN2-3 on cone specimen were included	hrHPV DNA test (6m)	6m	–	–
Armstrong 2022 (UK)¶	Retrospective cohort	LLETZ (732); CC (909)	Biopsy-proven CIN2–3; CC only in case of satisfactory colposcopy, no glandular lesion and no suspicion of invasion	hrHPV DNA test (6m)	6m	–	–

†This study was included in the main network (risk of treatment failure). ‡This study was included only in the network of HIV-infected women. §This study was included in the dose-response meta-analysis for cone length. ¶This study was included in the network for risk of positive hrHPV testing.

2.1.2. Preterm Birth

The search for reproductive outcomes yielded 4,107 records. After screening, there were 92 studies reporting on preterm birth rates. Of these, 81[34,109-188] were retrospective and eight[78,189-195] were prospective cohorts, one[196] was a case-control study, and two[94,197] were RCTs. Most compared a single treatment (or a merged group of excisional or ablative treatments) to an untreated external or internal comparator and were not included in the network. Characteristics of studies are reported in the table below (Table 2.1.2.1). The network for preterm birth included 72,256 women in 29 eligible studies. In the network there were 22[34,109,121,123,130,136,140-142,145,147,149,152,157,159,162,163,166,171,179-181] retrospective and five[78,189,190,193,195] prospective cohorts, and two[94,197] (quasi-) RCTs. The network included CKC, LC, LLETZ RD, LA, CC, CT, as well as untreated women attending for colposcopy (colposcopy group); no studies reported on NETZ or FCBE. See Section 2.2.2 on how we managed overlapping studies. The majority of studies recruited women at the time of their pregnancy or delivery; only a minority of studies recruited women at the time of CIN treatment. The median of the median age at pregnancy across studies was 30y (IQR=29–30), while the median of the percentage treated for CIN2+, CIN3+, AIS, and cervical cancer was 83% (IQR=70–94), 61% (IQR=48–74), 0% (IQR=0–0), and 0% (IQR=0–0), respectively.

Table 2.1.2.1: Characteristics of studies reporting on risk of preterm birth after CIN treatments

Study (Country)	Study Design	Procedure	N Treated†	CIN grade	Comparison Group	N Untreated†	Source of data	Pregnancies included & immune status (if reported)	Outcomes
Jones 1979 (UK)‡	Retrospective cohort	CKC	66	Not reported	External: matching for age, parity, social class, date of delivery	264	Exposure: Cardiff Cervical Cytology Study<br><br>Outcome: Cardiff Birth Survey	Singleton pregnancies >28w	Preterm birth
Praest 1979 (Denmark)‡	Retrospective cohort	CKC	63	Mostly high-grade lesions (CIN2+: 95%; CIN3: 85%)	Internal	115	Exposure: Records of Aalborh Hospital<br><br>Outcome: not reported	All pregnancies (no information about multiple pregnancies)	Preterm birth
Leiman 1980 (South Africa)¶	Retrospective cohort	CKC	88	Cytological HSIL	–	–	Exposure & Outcome: Records of Baragwanath Hospital	All pregnancies (no information about multiple pregnancies)	Preterm birth
Buller 1982 (USA)‡	Retrospective cohort	CKC	88	CIN or cancer IA1 on cone histology (CIN3+: 48%)	Internal	106	Exposure: Records of University of California Hospital and Kaiser Hospital<br><br>Outcome: not reported	All pregnancies (including multiple pregnancies)	Preterm birth
Hemmingsson 1982 (Sweden)‡	Retrospective cohort	CT	115	Not reported	Internal	65	Exposure: Records of University Hospital of Uppsala<br><br>Outcome: not reported	Pregnancies ≥28w (no information about multiple pregnancies)	Preterm birth
Larsson 1982 (Sweden)‡	Retrospective cohort	CKC	294	Not reported	Internal	341	Not reported	All pregnancies (including multiple pregnancies)	Preterm birth
Ludviksson 1982 (Sweden)‡	Retrospective cohort	CKC	83	Not reported	External: matching for age, parity, date of delivery	79	Outcome: Records of Regional Hospital of Orebro<br><br>Outcome: not reported	Deliveries (elective caesarean sections were excluded; no information about multiple pregnancies)	Preterm birth
Moinian 1982 (Sweden)‡	Retrospective cohort	CKC	122	Not reported	Internal	801	Exposure: Records of East Hospital of Gothenburg<br><br>Outcome: not reported	All pregnancies (terminated pregnancies were excluded; no information about multiple pregnancies)	Preterm birth

Anderson 1984 (UK)‡	Retrospective cohort	LA	68	Not reported	External: matching for age, parity, race, history of miscarriages/terminated pregnancies	70	Exposure: Records of Samaritan Hospital for Women (London)<br><br>Outcome: postal questionnaires and private records for treated women; records of St Mary's Hospital (London) for untreated women	Deliveries (no information about multiple pregnancies)	Preterm birth
Kristensen 1985 (Denmark)‡	Retrospective cohort	Excision	85	Not reported	External: matching for age, parity	12792	Exposure: Registry of the county of Funen (located at the data processing unit at Odense University Hospital)<br><br>Outcome: Registry of the county of Funen for treated women (or questionnaires for treated women having left the country); Records of Odense University Hospital for untreated women	Singleton pregnancies >28w	Preterm birth
Kuoppala 1986 (Finland)‡	Retrospective cohort	CKC	62	Up to CIN3 on cone histology (no CIN: 1%; CIN3: 68%)	External: matching for age, parity, date of delivery, singleton pregnancy	62	Exposure: Records of University Central Hospital of Tampere<br><br>Outcome: not reported	Deliveries >28w (multiple pregnancies were possibly included)	Preterm birth
Saunders 1986 (UK)‡	Retrospective cohort	LA	97	Biopsy-proven CIN	External: matching for age, parity, race, year of delivery, singleton pregnancy	97	Exposure & Outcome: Hospital records; local general practitioners (Sheffield)	Pregnancies >12w (multiple pregnancies were possibly included)	Preterm birth
Wakita 1990 (Japan)§	Retrospective cohort	LC; LA	36 (LC: 10; LA: 26)	CIN or cancer IA1 (CIN2+: 94%; CIN3+: 78%)	–	–	Exposure: Hospital records<br><br>Outcome: not reported	All pregnancies (no information about multiple pregnancies)	Preterm birth
Kasum 1991 (Croatia)‡	Retrospective cohort	Excision	68	CIN or cancer IA1 on cone histology (no CIN: 1%; CIN3+: 96%)	External: matching for date of delivery	68	Exposure: Records of University Hospital of Zagreb<br><br>Outcome: postal questionnaires and interview (for treated women); records of University Hospital of Zagreb (for untreated women)	All pregnancies (terminated pregnancies were excluded; no information about multiple pregnancies)	Preterm birth
Gunasekera 1992 (UK)§	Prospective cohort	LLETZ; LA	140 (LLLETZ: 23; LA: 117)	Not reported	External: matching for age, parity, smoking, race, gestational age at the time of pregnancy diagnosis	140	Exposure: Records of Watford General Hospital<br><br>Outcome: Prospective f-u	All pregnancies (no information about multiple pregnancies)	Preterm birth
Loizzi 1992 (UK)§	Retrospective cohort	CKC; CT	43 (CKC: 34; CT: 9)	CIN1–3	–	–	Exposure: Hospital records<br><br>Outcome: not reported	All pregnancies (no information about multiple pregnancies)	Preterm birth
Blomfield 1993 (UK)‡	Retrospective cohort	LLETZ	40	Up to CIN3 (no CIN: 23%; CIN2+: 58%)	External: matching for age, parity, ethnic group, date of delivery	80	Exposure & Outcome: Records of Dudley Road Hospital	Deliveries (including multiple pregnancies)	Preterm birth
Haffenden 1993 (UK)‡	Retrospective cohort	LLETZ	152	LSIL+ or persistent ASC-US on cytology	External: matching for age, parity, date of delivery	152	Exposure & Outcome: Records of Gloucestershire Royal Hospital	Deliveries >24w (no information about multiple pregnancies)	Preterm birth

Hagen 1993 (Norway)‡	Retrospective cohort	LC	56	Not reported	A) External: matching for age, parity, date of delivery; regression for sociodemographic or pregnancy-related factors (such as smoking) did not change ORs<br><br>B) Internal	A) 112<br><br>B) 35	Exposure: Records of University Hospital of Trondheim<br><br>Outcome: not reported	Singleton deliveries >22w (stillbirths were excluded)	Preterm birth
Braet 1994 (UK)‡	Retrospective cohort	LLETZ	78	Not reported	External: matching for age, parity, smoking	78	Exposure: Records of Rotherham District General Hospital<br><br>Outcome: not reported	Singleton viable pregnancies >24w	Preterm birth
Cruickshank 1995 (UK)‡	Retrospective cohort	LLETZ	149	Not reported	A) External: age, parity, smoking, partner's social class, height<br><br>B) Internal	A) 298<br><br>B) 133	Exposure: Records of Wellbeing Centre for the Prevention of Cervical Cancer (Aberdeen)<br><br>Outcome: Aberdeen Maternity and Neonatal Databank	Singleton pregnancies >20w	Preterm birth
Sagot 1995 (France)‡	Retrospective cohort	LC	71	CIN3: 45%	Internal	82	Exposure: Records of Mere-Enfant Hospital (Nantes)<br><br>Outcome: not reported	All pregnancies (including multiple pregnancies)	Preterm birth
Spitzer 1995 (Jamaica)§	Retrospective cohort	LC; LA	277 (LC: 67; LA: 210)	Not reported	Internal: matching for age, parity	177	Exposure: records of Colposcopy Clinic of Queens Hospital Centre; private practice records<br><br>Outcome: Postal questionnaires; phone or in-person interview	All pregnancies (no information about multiple pregnancies)	Preterm birth
Bekassy 1996 (Sweden)‡	Retrospective cohort	LC (‘mini-conisation’)	250	Not reported	A) External: matching for age, parity, date of delivery<br><br>B) Internal	A) 250<br><br>B) 148	Exposure: Records of University Hospital of Lund<br><br>Outcome: National Medical Birth Registry	Deliveries (including multiple pregnancies)	Preterm birth
Raio 1997 (Switzerland)¶	Retrospective cohort	LC	64	CIN1–3 (CIN2+: 86%; CIN3+: 52%)	A) External: matching for age, parity, smoking, marital status, social class, history of preterm birth<br><br>B) Internal	A) 64<br><br>B) 26	Exposure: Records of Kantonsspital (Münesterlingen)<br><br>Outcome: not reported	Singleton deliveries	Preterm birth
Andersen 1999 (Denmark)¶	Retrospective cohort	LC	75	Not reported	External: matching for age, parity, date of delivery	150	Exposure: Records of Aalborh Hospital<br><br>Outcome: not reported	Pregnancies >27w (no information about multiple pregnancies)	Preterm birth

El-Bastawissi 1999 (USA)‡	Retrospective cohort	Excision (CKC, LC, LLETZ); Ablation (LA, CT)	1244 (Excision: 1153; Ablation: 91)	CIN3	A) External: matching for age, country of origin; regression for parity, smoking, race, marital status, history of terminated pregnancies (adjusted ORs are reported only for excision)<br><br>B) Untreated CIN3: no matching; no regression	A) 9201<br><br>B) 330	Exposure: Cancer Surveillance System<br><br>Outcome: Washington State Birth Certificates	Singleton deliveries	Preterm birth
van Rooijen 1999 (Sweden)‡	Retrospective cohort	LA	236	Biopsy-proven CIN1–3 (CIN2+: 62%)	External: matching for age, parity, year of delivery	472	Exposure: Records of Karolinska Hospital<br><br>Outcome: not reported	Deliveries (no information about multiple pregnancies)	Preterm birth
Mathevet 2003 (France)§	RCT	CKC; LC; LLETZ	50 (CKC: 13; LC: 25; LLETZ: 12)	HSIL or LSIL with unsatisfactory colposcopy	–	–	Exposure & Outcome: Prospective f-u	All pregnancies (no information about multiple pregnancies)	Spontaneous preterm birth
Sadler 2004 (New Zealand)§¶	Retrospective cohort	LC; LLETZ; LA	606 (LC: 105; LLETZ: 278; LA: 223)	Up to cancer IA1 on histology (CIN2+: 63%)	Colposcopy without treatment: regression for age, parity, smoking, ethnicity, socioeconomic status, history of preterm birth, antepartum haemorrhage, interhospital transfer (variables were manually removed if they were not found to be important confounders)	426	Exposure & Outcomes: Records of national Women's Hospital	Singleton pregnancies >20w	Preterm birth
Tan 2004 (UK)‡	Retrospective cohort	LLETZ	119	Not reported	External: matching for age, parity	119	Exposure & Outcome: Records of Basildon District Hospital	All pregnancies (no information about multiple pregnancies)	Preterm birth
Samson 2005 (Canada)‡	Retrospective cohort	LLETZ	571	Not reported	External: matching for age, parity, smoking, year of delivery	571	Exposure: Provincial Cytology/Colposcopy Registry<br><br>Outcome: Nova Scotia Atlee Perinatal Database (only deliveries in IWK Health Centre were included)	Singleton pregnancies >20w	Preterm birth
Crane 2006 (Canada)§	Prospective cohort‖	CKC; LLETZ; CT	132 (CKC: 21; LLETZ: 75; CT: 36)	CIN1–3 (CIN2+: 62%; CIN3: 39%)	External: regression for age, parity, smoking, antpartum haemorrhage (>20w), history of spontaneous preterm birth, gestational age at the time of ultrasound scan (only variables with P<0·10 were kept in the final model; adjusted OR was not reported for CT)	81	Exposure: not reported<br><br>Outcome: Prospective f-u	Singleton pregnancies >24w	Spontaneous preterm birth
Klaritsch 2006 (Austria)‡	Retrospective cohort	CKC	76	CIN1–3 on cone histology (CIN2+: 98%; CIN3: 87%)	External: no matching; no regression	29711	Exposure: Hospital records<br><br>Outcome: Records of University Hospital of Graz	Singleton deliveries	Preterm birth

Bruinsma 2007 (Australia)§	Retrospective cohort	CKC; LLETZ; RD; LA	1905 (CKC: 71; LLETZ: 69; RD: 760; LA: 1005)	CIN2+: 58%	Colposcopy without treatment: regression for age, parity, marital status, maternal medical conditions, country of origin, history of miscarriage, history of preterm birth, illicit drug use (parity and country of origin were not included in the final model because of non-significant contribution)	3484	Exposure: Records of the Royal Women's Hospital<br><br>Outcome: Victorian Perinatal Data Collection Unit	Singleton pregnancies >20w	Preterm birth
Bull-Phelps 2007 (USA)§	Retrospective cohort	CKC; LLETZ	49 (CKC: 39; LLETZ: 10)	AIS	–	–	Exposure & Outcome: Records of Brigham and Women's Hospital, University of Texas Southwestern Medical Centre at Dallas, and University of North Carolina at Chapel Hill	All pregnancies (no information about multiple pregnancies)	Preterm birth
Himes 2007 (USA)§	Retrospective cohort	LLETZ	114	Not reported	PB without treatment: no matching; no regression	962	Exposure and Outcome: Records of Magee-Womens Hospital	Singleton non-anomalous pregnancies >20w	Preterm birth
Jakobsson 2007 (Finland)‡	Retrospective cohort	Excision; (CKC, LC, LLETZ); Ablation (LA, CT, electrocoagulation); Other	8210 (Excision: 4846; Ablation: 3576; Other: 242)	Not reported	External: regression for age, parity, smoking	1056855	Exposure: Hospital Discharge Register<br><br>Outcome: Finnish Medical Birth Register	Singleton deliveries	Preterm birth
Albrechtsen 2008 (Norway)‡	Retrospective cohort	Excision (CKC, LC, LLETZ)	15108	Not reported	A) External: regression for age, parity<br><br>B) Internal: regression for age, parity	A) 2164006<br><br>B) 57136	Exposure: Cancer Registry of Norway<br><br>Outcome: Medical Birth Registry of Norway	Pregnancies >16w (no information about multiple pregnancies)	Preterm birth
Patrelli 2008 (Italy)§	Retrospective cohort	CKC; LC; LLETZ	80 (CKC: 32; LC: 3; LLETZ: 45)	Up to CIN3 on cone histology (no CIN: 12%; CIN2+: 70%)	-	-	Exposure & Outcome: Records of University Hospital of Parma	Singleton pregnancies	Preterm birth
Jakobsson 2009 (Finland)‡	Retrospective cohort	LLETZ	258	no CIN: 10%; CIN2+: 52%; CIN3+: 20%	A) External<br><br>B) Internal<br><br>In both: regression for age, parity (only unadjusted RRs were reported because adjustment for these confounders did not change the results)	A) 554507<br><br>B) 258	Exposure: Hospital Discharge Register<br><br>Outcome: Finnish Medical Birth Register	Singleton deliveries	Preterm birth
Michelin 2009 (Brazil)§	Retrospective cohort	CKC; LLETZ	42 (CKC: 23; LLETZ: 19)	Not reported	–	–	Exposure: Records of Research Institute of Oncology<br><br>Outcome: not reported	All pregnancies (no information about multiple pregnancies)	Preterm birth
Noehr 2009 (Denmark) (AJOG§ & Obstet Gynecol¶)	Retrospective cohort	LLETZ; Ablation	10207 (LLETZ: 8180; Ablation: 2027)	Up to CIN3 on cone histology (no CIN: 6%; CIN2+: 85%; CIN3: 67%)	A) External<br><br>B) PB without treatment<br><br>In both: regression for age, smoking, marital status, year of delivery	A) 510841<br><br>B) 31630	Exposure: Danish Registry of Pathology; National Patient Registry<br><br>Outcome: Medical Birth Registry; National Patient Registry	Singleton deliveries 21–45w	Spontaneous preterm birth

Shanbhag 2009 (UK)‡	Retrospective cohort	Excision (CKC, LC, LLETZ); Ablation (LA, CC, diathermy coagulation)	1388 (Excision: 1103; Ablation: 285)	CIN3	A) External: no matching; no regression<br><br>B) Untreated CIN3: regression for age, smoking, deprivation level, year of delivery, malpresentation, birth weight, preterm birth*, spontaneous preterm birth*, pPROM*, caesarean section*<br><br>*Adjustment depended on outcome (e.g. for preterm birth or spontaneous preterm birth there was adjustment for pPROM); because of adjustment for post-intervention factors, we used unadjusted data	A) 119216<br><br>B) 87	Exposure: Scottish Cancer Registry<br><br>Outcome: Scottish Morbidity Record (SMR02)	Pregnancies 24–43w (and birth weight >350g)	Preterm birth
Zornoza-Garcia 2009 (Spain)‡	Retrospective cohort	LLETZ*<br><br>*some excisions were performed in two passes	46	Up to CIN3 on cone histology (no CIN: 24%; CIN2+: 67%; CIN3: 43%)	External: no matching; no regression	2759	Exposure & Outcome: Records of Leon Hospital	Singleton pregnancies	Preterm birth
Fischer 2010 (USA)‡	Prospective cohort‖	Excision (CKC, LLETZ)	85 (CKC: 48; LLETZ: 68; both: 2)	Not reported	External: matching for age, race, history of vaginal deliveries (≥20w), gestational age at the time of ultrasound scan	85	Exposure: referral records or self-reporting<br><br>Outcome: Prospective f-u	Singleton pregnancies >15–22w	Preterm birth
Nam 2010 (South Korea)§	Retrospective cohort	CKC; LLETZ*<br><br>*one to three fragments	65 (CKC: 14; LLETZ: 51)	Not reported	–	–	Exposure: Medical Records<br><br>Outcome: Records of Yonsei University Health System (Seoul)	Singleton deliveries (stillbirths were excluded)	Preterm birth
Ortoft 2010 (Denmark)‡	Retrospective cohort	CKC; LLETZ*; electric knife<br><br>*top-hat LLETZ rarely performed	746 (CKC: 67; LLETZ: 572; electric knife: 71)	CIN3+: 89%	A) External: regression for age, parity, smoking, education, marital status<br><br>B) Untreated HSIL: regression for age, parity, smoking, education, marital status (adjusted RRs were not reported separately for each treatment technique)<br><br>C) Internal	A) 72899<br><br>B) 383<br><br>C) 170	Exposure: Danish nationwide pathology database (only specimens examined at University Hospital of Aarhus included)<br><br>Outcome: Records of Aarhus University Hospital; questionnaires for previous pregnancies	Singleton deliveries	Spontaneous preterm birth
van de Vijver 2010 (Belgium)¶	Retrospective cohort	Excision (LC, LLETZ)	55 (LC: 5; LLETZ: 50)	CIN1–3 on cone histology; CIN2+: 75%; CIN3: 65%)	External: matching for age, parity, year of delivery	55	Exposure: Records of University Hospital of Leuven<br><br>Outcome: Questionnaires for treated women; records of University Hospital of Leuven for untreated women	Pregnancies >22w (including multiple pregnancies)	Preterm birth

Werner 2010 (USA)‡	Retrospective cohort	LLETZ*<br><br>*an additional pass was occasionally performed for excisional of additional ectocervical or distal endocervical tissue	842	Not reported	A) External: no matching, no regression (authors performed regression for age, parity and race, but adjusted effect estimates were not reported)<br><br>B) Internal	A) 240348<br><br>B) 511	Exposure & Outcome: Records of Parkland Health & Hospital System	Singleton deliveries	Preterm birth
Andia 2011 (Spain)‡	Retrospective cohort	LLETZ	189	Not reported	A) External: matching for age, parity<br><br>B) Internal: matching for age, parity	A) 189<br><br>B) 189	Exposure: Records of the 5 main hospitals of the Basque County<br><br>Outcome: Basque Country Health Service	Singleton deliveries	Preterm birth
Lima 2011 (Portugal)§¶	Retrospective cohort	LC; LLETZ	29 (LC: 11; LLETZ: 18)	Up to CIN3 on cone histology (no CIN: 7%; CIN2+: 86%; CIN3: 69%)	External: matching for date of delivery	58	Exposure & Outcome: Records of Dr Alfredo Da Costa Maternity (Lisbon)	Deliveries (no information about multiple pregnancies)	Preterm birth
Castanon 2012 (UK)‡	Retrospective cohort	Excision (CKC, LC, LLETZ)	4776	Not reported	A) External (general population): no matching; no regression<br><br>B) PB without treatment: regression for age, parity, hospital<br><br>C) Internal: regression for age, parity, hospital	A) 510660<br><br>B) 4770<br><br>C) 1045	Exposure: Records of 12 Hospitals<br><br>Outcome: Hospital episode statistics of inpatient obstetric records (whole of England)	Singleton deliveries 20–43w (stillbirths excluded)	Preterm birth
Khalid 2012 (UK)¶	Retrospective cohort	LLETZ	321	Not reported	–	–	Exposure & Outcome: Records of Coombe Women & Infants University Hospital	Singleton pregnancies	Preterm birth
Poon 2012 (UK)‡	Prospective cohort‖	LLETZ	473	Not reported	External: regression for parity, race, smoking, history of preterm birth, history of miscarriages	25772	Exposure: Questionnaires<br><br>Outcome: Prospective f-u	Singleton pregnancies >20–24w	Spontaneous preterm birth
Reilly 2012 (UK)§	Retrospective cohort	LLETZ; Ablation (LA, CC, CT); other	2202 (LLETZ: 1546; Ablation: 534; other: 40; multiple: 82)	Not reported	A) External (negative cytology)<br><br>B) Colposcopy without treatment<br><br>In both: regression for age, parity, smoking, social deprivation, time to conception, history of adverse pregnancy outcomes	A) 38983<br><br>B) 2534	Exposure: Cervical Screening Wales programme database<br><br>Outcome: National Community Child Health Database and All Wales Perinatal Survey	Singleton pregnancies ≥24w	Preterm birth
Simoens 2012 (Belgium)§¶	Prospective cohort‖	LC; LLETZ; Excision (CKC, LC, LLETZ, unknown)	88 (CKC: 8; LC: 24; LLETZ: 52; unknown: 4)	CIN1–3 on cone histology (CIN2+: 81%; CIN3: 57%)	External: matching for hospital, date of delivery; regression for age, parity, smoking, ethnicity, education, HIV	176	Exposure: Questionnaires along with checking of medical records<br><br>Outcome: Prospective f-u	Singleton pregnancies >20w<br><br>3% were HIV-positive	Preterm birth

Van Hentenryck 2012 (Belgium)‡	Retrospective cohort	Excision (CKC, LC, LLETZ)	106	Histological CIN1–3 (CIN2+: 88%; CIN3: 61%)	External: matching for age, parity, smoking, HIV, history of gestation	212	Exposure & Outcome: Records of Erasme University Hospital	Deliveries (no information about multiple pregnancies)<br><br>Women with HIV were included; authors matched for HIV status but did not report how many women were HIV-positive	Preterm birth
Berretta 2013 (Italy)¶	Retrospective cohort	CKC; LLETZ	45 (CKC: 8; LLETZ: 37)	Histological CIN1-3 (CIN2+: 96%; CIN3: 44%)	–	–	Exposure: Records of University Hospital of Parma<br><br>Outcome: Interview	Pregnancies >24w (no information about multiple pregnancies)	Spontaneous preterm birth
Frega 2013 (Italy)‡	Prospective cohort	LLETZ	475	CIN2–3	External: control for parity, race and smoking (only nulliparous, white women and non-smokers were included)	441	Exposure: Records of university teaching hospitals and country hospitals across Italy (probably six hospitals located in Rome and Aviano participated)<br><br>Outcome: Prospective f-u	Singleton pregnancies<br><br>Women with any major disease, including HIV infection, were excluded	Preterm birth
Frey 2013 (USA)§	Retrospective cohort	LLETZ	598	Not reported	A) External with prior cervical smear: matching for age, year of treatment/smear<br><br>B) PB without treatment: matching for age, year of treatment/PB	A) 588<br><br>B) 552	Exposure & Outcome: Records of 9 participating hospitals	Singleton pregnancies >20w	Preterm birth
Guo 2013 (China)§	Prospective cohort	CKC; LLETZ	84 (CKC: 36; LLETZ: 48)	CIN1–3 (CIN2+: 86%; CIN3: 45%)	PB (CIN1 or less) without treatment: control for smoking (only non-smokers were included)	68	Exposure: Records of the First Affiliated Hospital of Zhengzhou University<br><br>Outcome: Prospective f-u	Ectopic pregnancies, terminated pregnancies and 1st trimester miscarriages were excluded; multiple pregnancies were included	Preterm birth
Castanon 2014 (UK)¶	Case-control	Excision (CKC, LC, LLETZ*)<br><br>*27% had a piecemeal excision	1114	Not reported	PB without treatment: regression for age, parity, deprivation, hospital	484	Exposure: Records of 12 Hospitals<br><br>Outcome: Outcome: Hospital episode statistics of inpatient obstetric records (whole of England)	Singleton deliveries 20–43w (stillbirths were excluded)	Preterm birth
Kitson 2014 (UK)§	Retrospective cohort	LLETZ	278	CIN2+ on cone histology: 81%	PB without treatment: matching for age, parity, smoking	278	Exposure & Outcome: Records of a single tertiary referral hospital	Singleton pregnancies >20w	Preterm birth
Liu 2014 (China)§	(quasi-) RCT	CKC; LLETZ	CKC: 120; LLETZ: 124	CIN2–3	–	–	Exposure & Outcome: Prospective f-u	Singleton pregnancies	Preterm birth

Sozen 2014 (Turkey)‡	Retrospective cohort	CKC	15	Not reported	External: matching for age, parity, obstetric history	24	Exposure: Records of the Zeynep Kamil Women's Hospital<br><br>Outcome: Hospital records (probably of the same hospital)	Deliveries >20w (no information about multiple pregnancies)	Preterm birth
Cai 2015 (China)§	Retrospective cohort	CKC; LLETZ	101 (CKC: 45; LLETZ: 56)	CIN1–3 (CIN2+: 83%; CIN3: 61%)	External: control for parity (only primiparous women were included)	71	Exposure: Hospital records<br><br>Outcome: not reported	All pregnancies (no information about multiple pregnancies)	Preterm birth
Kirn 2015 (Germany)‡	Retrospective cohort	Excision	135	Not reported	External: matching for age, parity, smoking	135	Exposure: not reported<br><br>Outcome: Records of University Hospital of the Ludwig-Maximilians University	Singleton deliveries	Preterm birth
Martyn 2015 (Ireland)§	Retrospective cohort	LLETZ	434	Not reported	Colposcopy without treatment: matching for age	296	Exposure: Records of the National Maternity Hospital<br><br>Outcome: Postal questionnaires	Pregnancies ≥24w (no information about multiple pregnancies)	Preterm birth
Miller 2015 (USA)‡	Retrospective cohort	Excision	1356	Not reported	A) External<br><br>B) History of CIN without treatment<br><br>In both: regression for age, race/ethnicity, BMI	A) 14149<br><br>B) 3023	Exposure: Patient's prenatal records<br><br>Outcome: Records of Northwestern Memorial Hospital	Singleton pregnancies >18–24w	Preterm birth
Aleman 2016 (Belgium)‡	Retrospective cohort	Excision	18	Not reported	External: regression for smoking, alcohol, chronic disorders, gynaecological disorders	325	Exposure: Questionnaires<br><br>Outcome: Records of Antwerp University Hospital	Singleton pregnancies 24–42w	Preterm birth
Anwar 2016 (UK)‡	Retrospective cohort	FCBE	28	CIN or cancer IA1 on cone histology (no CIN: 18%; CIN2+: 88%); main indication for excision was glandular lesions (44%)	Internal	59	Exposure & Outcome: Records of Diana Princess of Wales Hospital	All pregnancies (no information about multiple pregnancies)	Preterm birth
Bjorge 2016 (Norway)§	Retrospective cohort	CKC; LC; LLETZ; LA; CT; diathermy excision; radiowave excision	9554 (CKC: 61; LC: 3587; LLETZ: 4361; LA: 96; CT: 24; diathermy excision: 1370; radiowave excision: 55)	histological CIN2+: 98%; histological CIN3+: 85%	External: regression for age, parity, smoking, marital status, education, country of origin, year of delivery, history of cytology screening	933767	Exposure: Cancer Registry of Norway<br><br>Outcome: Medical Birth Registry of Norway	Singleton pregnancies >16w	Preterm birth
Brie 2016 (France)¶	Retrospective cohort	FCBE	39	Up to CIN3 on cone histology (no CIN: 6%; CIN2+: 84%; CIN3: 52%)	External: matching probably for date of delivery	78	Exposure & Outcome: Records of a single University Hospital	Pregnancies >22w (no information about multiple pregnancies)	Preterm birth

Jancar 2016 (Slovenia)§	Retrospective cohort	CKC; LLETZ	4581 (CKC: 2083; LLETZ: 2498)	Not reported	External: adjustment for age, parity, smoking, education	188150	Exposure: Medical History<br><br>Outcome: Medical Birth Registry - National Perinatal Information System of Slovenia (NPIS)	Spontaneous singleton deliveries	Spontaneous preterm birth
Liverani 2016 (Italy)¶	Retrospective cohort	LLETZ*<br><br>*excision was performed in two steps if necessary	501	CIN2–3	–	–	Exposure & Outcome: Records of Policlinic Hospital, University of Milano	Singleton deliveries<br><br>Women with HIV, hepatitis B or hepatitis C infection were excluded	Preterm birth
Mariya 2016 (Japan)§	Prospective cohort	LC; LA	29 (LC: 14; LA: 15)	CIN3	–	–	Exposure: Records of NTT East Sapporo Medical Centre<br><br>Outcome: Prospective f-u	Deliveries	Preterm birth
Chevreau 2017 (France)¶	Retrospective cohort	LLETZ	115	Biopsy-proven CIN2+ or persistent cytological abnormality (18-month persistent LSIL or ASC-US; 12-month persistent HSIL or ASC-H with normal colposcopy); CIN2+ on cone histology: 83%	–	–	Exposure & Outcome: Records of University Hospital of Amiensm	Pregnancies >14w (no information about multiple pregnancies)	Preterm birth
van Velthoven 2017 (Belgium)¶	Retrospective cohort	LLETZ*<br><br>*26% underwent top-hat LLETZ	120	CIN2+: 80%; CIN3+: 61%	External: matching for age, parity, singleton pregnancy	120	Exposure: Records of University Hospital of Leuven<br><br>Outcome: not reported	Pregnancies >22w (including multiple pregnancies)	Preterm birth
Weinmann 2017 (USA)¶	Retrospective cohort	Excision*; Ablation<br><br>*some women underwent top-hat LLETZ	322 (Excision: 229; Ablation: 93)	Not reported	A) External: matching for age, year of delivery<br><br>B) Colposcopy without treatment<br><br>In both: regression for age, parity, smoking, race, BMI, history of preterm birth	A) 4307<br><br>B) 847	Exposure: Electronic Medical Records (EMR)<br><br>Outcome: Kaiser Permanente Northwest (KPNW)	Singleton deliveries (stillbirths were excluded)	Preterm birth
Zebitay 2017 (Turkey)‡	Retrospective cohort	CKC	20	Biopsy-proven CIN1–3 (CIN2+: 85%; CIN3: 35%)	External: control for smoking (only non-smokers were included)	38872	Exposure & Outcome: Records of Haseki Education and Research Hospital (Istanbul) & Suleymaniye Maternity and Women's Disease Education and Research Hospital (Istanbul)	Singleton pregnancies	Preterm birth

Heinonen 2018 (Finland)§	Retrospective cohort	LLETZ	797	CIN1	A) External: regression for age, parity, smoking, year of birth, history of preterm birth, living environment<br><br>B) Women with untreated CIN1: regression for age, socioeconomic status, marital status, history of preterm birth<br><br>C) Internal	A) 631314<br><br>B) 2220<br><br>C) 1698	Exposure: Hospital Discharge Register<br><br>Outcome: Finnish Medical Birth Register	Singleton deliveries	Preterm birth
Papoutsis 2018 (UK)§	Retrospective cohort	LLETZ*; CC<br><br>*33% had multiple fragments	161 (LLETZ: 86; CC: 75)	LLETZ: biopsy-proven CIN2–3, or HSIL cytology and colposcopy (see-and-treat), or persistent LSIL; CC: biopsy-proven CIN with no evidence of invasion or crypt involvement by CIN2–3	External: regression for age, parity, smoking, pre-treatment cytology, pre-treatment colposcopy; number of PBs; maximum depth of PBs	55094	Exposure & Outcome: Records of Shrewsbury and Telford Hospital NHS Trust	Singleton pregnancies (ectopic pregnancies and stillbirths were excluded)	Spontaneous preterm birth
Wittmaack 2019 (USA)‡	Retrospective cohort	Excision (CKC, LC, LLETZ)	505	Not reported	External: regression for parity, smoking, pregnancy-related hypertension, pPROM, placenta previa, bacterial vaginosis, history of preterm birth, ethnicity, BMI*<br><br>*Because authors adjusted for pPROM (a post-intervention outcome affected by treatment), we used only unadjusted data	4800	Exposure: Medical records (ICD-9 diagnoses)<br><br>Outcome: Records of the University of Virginia Hospital	Singleton pregnancies ≥24w	Preterm birth
Zhang 2020 (China)§	Retrospective cohort	CKC; LLETZ	57 (CKC: 39; LLETZ: 18)	Histological CIN1–3	–	–	Exposure: Records of Sir Run Run Shaw Hospital (Zhejiang)<br><br>Outcome: not reported	All pregnancies (no information about multiple pregnancies)	Preterm birth
Collins 2021 (UK)¶	Retrospective cohort	LLETZ	624	Not reported	–	–	Exposure: Pathology reports<br><br>Outcome: Records of University Hospitals of Leicester NHS Trust	Singleton pregnancies ≥16w	Preterm birth

Loopik 2021 (Denmark)‡	Retrospective cohort	Excision	9412	Histological diagnosis of CIN or AIS	A) External: matching for age, year, urbanisation<br><br>B) Women with untreated CIN<br><br>In both: regression for age, year, urbanisation, ethnicity, diabetes mellitus, maternal infection, epilepsy, psychiatric diseases, history of abortion, history of preterm birth, pregnancy through in vitro fertilisation, parity, pre-eclampsia, gestational diabetes, placental abruption, placenta or vasa previa, congenital diseases, intrauterine growth restriction, macrosomia, stillbirth, foetal distress	A) 29907<br><br>B) 5940	Exposure: Dutch pathology registry (PALGA)<br><br>Outcome: Dutch perinatal database (Perined)	Singleton pregnancies ≥16w	Spontaneous preterm birth
Nitahara 2021 (Japan)‡	Retrospective cohort	LA	114	Not reported	External: regression for age, maternal weight, smoking during pregnancy, hypertension, impaired glucose tolerance, use of public welfare support	3245	Exposure: not reported<br><br>Outcome: Hospital records	Singleton deliveries	Preterm birth
Mosseri 2022 (France)¶	Retrospective cohort	LC	71	Not reported	–	–	Exposure: Single centre's hospital records<br><br>Outcome: not reported	Singleton pregnancies >22w	Preterm birth
Panelli 2022 (USA)¶	Retrospective cohort	LLETZ	134	Not reported	–	–	Exposure & Outcome: Records of Brigham and Women's Hospital or Massachusetts General Hospital	Pregnancies ≥16w	Preterm birth
Wiik 2022 (Sweden)¶	Retrospective cohort	Excision	3250	Not reported	A) External<br><br>B) Women with untreated CIN diagnosed during pregnancy<br><br>In both: regression for age, parity, smoking, year, BMI, marital status, country of birth, infant's sex, income, education, assisted reproduction	A) 42398<br><br>B) 1380	Exposure: Swedish National Cervical Screening Registry/ Analysis; Swedish Cancer Registry<br><br>Outcome: Swedish Medical Birth Register	Singleton deliveries<br><br>Women with chronic inflammatory diseases, organ transplantation or HIV were excluded	Preterm birth

†N refers to number of pregnancies. ‡This study was eligible for inclusion only in the standard pairwise meta-analyses. §This study was included in the network meta-analysis. ¶This study was included in the dose-response meta-analysis for cone length. ||Women were recruited during their pregnancy; f-u was prospective from recruitment until delivery but retrospective from CIN treatment until recruitment.

2.2. Overlapping Studies

In this section we describe how we managed potentially overlapping studies.

2.2.1. Treatment Failure

We identified no overlapping studies for treatment failure.

2.2.2. Preterm Birth

For preterm birth there were many population-based studies with the risk of overlapping with other population- or hospital-based studies from the same country.

Denmark

Three studies partially overlapped (Noehr 2009,[149] Ortoft 2010,[153] Loopik 2021[186]). A fourth study (Noehr 2007[198]) was excluded because of complete overlapping with the larger study by Noehr 2009. Ortoft 2010 partially overlapped with the larger studies by Noehr 2009 and Loopik 2021; we used Ortoft 2010 only in the analyses with an internal comparison group since Noehr 2009 and Loopik 2021 had no internal comparison group. There was no overlapping between Noehr 2009 and Loopik 2021 since the former study included women treated up to 2005, whilst the latter study included women treated after 2005.

Finland

Three studies partially overlapped (Jakobsson 2007,[143] Jakobsson 2009,[146] Heinonen 2018[180]). A fourth study (Heinonen 2013[199]) was excluded because of complete overlapping with the larger study by Jakobsson 2007. Only Heinonen 2018 was eligible for inclusion in the NMA. All studies had an external group and we used Jakobsson 2007 in the pairwise comparison 'Treatment vs External', because this was the largest study. Both Jakobsson 2009 and Heinonen 2018 had an internal group, but we used the former in the comparisons 'Treatment vs Internal' and 'Internal vs External', because this was the largest study. Only Heinonen 2018 had a colposcopy group and was used for the comparisons 'Treatment vs Colposcopy' and 'Internal vs Colposcopy'.

Norway

Two studies partially overlapped (Albrechtsen 2008,[144] Bjorge 2016[109]). Two other studies (Acharya 2005,[200] Sjoborg 2007[201]) were excluded because of complete overlapping with the former two larger studies. Only Bjorge 2016 was eligible for the NMA. We used Albrechtsen 2008 in the pairwise comparisons 'Treatment vs External', 'Treatment vs Internal' and 'Internal vs External', because this was the largest study.

Belgium

Two studies partially overlapped (Simoens 2012,[193] Van Hentenryck 2012[160]). Only Simoens 2012 was eligible for the NMA. We used Van Hentenryck 2012 in the pairwise comparison 'Treatment vs External', because this was the largest study.

UK

Three studies partially overlapped (Castanon 2012,[158] Castanon 2014,[196] Castanon 2015[202]). We used Castanon 2014 for the dose-response meta-analysis (i.e. the meta-analysis according to cone length) and Castanon 2012 for all other analyses. Castanon 2015 focused on the research question whether the increased risk of preterm birth is limited only to the first pregnancy after treatment, and was smaller than Castanon 2012/2014, therefore it was excluded.

2.3. Within-Study Risk of Bias

2.3.1. Treatment Failure

RoB was low in one[75] NRS and one[106] RCT, moderate in 13[31,50,52,54,58,62,65,68,70,72,77,80,203] NRS and 18[83-87,90,92-95,98-100,102-104,107] RCTs, and high (i.e. serious or critical) in 40[29,30,32-49,51,53,55-57,59-61,63,64,66,67,69,73,74,76,78,79,81,108] NRS and eight[82,88,89,91,96,97,101,105] RCTs. Detailed RoB is presented below (Table 2.3.1.1). Of the 71 studies included in the network, RoB was low in one[75] NRS, moderate in 11[31,50,52,54,58,62,65,70,72,77,80] NRS and 18[83-87,90,92-95,98-100,102-104,107] RCTs, and high in 34[30,32-44,46-49,51,53,55-57,59-61,63,64,66,76,78,79,81,108] NRS and seven[82,88,89,91,96,97,101] RCTs.

Table 2.3.1.1: Risk of bias in studies reporting on risk of CIN treatment failure

Study	RoB due to confounding (for RCTs this domain assessed the randomisation process) (Part 1)	RoB in selection of participants (Part 2) (only for NRS)	RoB in classification of interventions (Part 3) (only for NRS)	RoB due to deviations from intended interventions (Part 4)	RoB due to missing data (Part 5)	RoB in measurement of outcomes (Part 6)	RoB in selection of reported results (Part 7)	Overall RoB
Wright 1981*	1.1:Y; 1.2:N; 1.4:PY; 1.5:PY; 1.6:N Moderate RoB (CIN grade did not differ between groups; no control for age or smoking; because choice of treatment was based on year of treatment, RoB was considered to be moderate)	2.1:N; 2.4:Y Low RoB	3.1:Y; 3.2:Y; 3.3:N Low RoB	4.1:N Low RoB	5.1:Y; 5.2:PN; 5.3:PN Low RoB (all patients had at least 2 f-u visits, i.e. at least 12m f-u)	6.1:PN; 6.2:PY; 6.3:PY; 6.4:N Low RoB	7.1:N; 7.2:N; 7.3:N Low RoB	Moderate
Townsend 1983*	1.1:NI; 1.2:NI; 1.2:NI Some concerns (method of randomisation not reported; the percentage of each CIN grade was identical between groups; mean age per group not reported)	-	-	4.1:NI; 4.2:Y; 4.3:NI; 4.6:NI; 4.7:NI Some concerns (number of people changing intervention or type of analysis not reported)	5.1:Y Low RoB	6.1:N; 6.2:N; 6.3:NI; 6.4:PN; 6.5:PN Low RoB	7.1:NI; 7.2:N; 7.3:N Some concerns	Some concerns
Jobson 1984*	1.1:NI; 1.2:NI; 1.3:PN Some concerns (method of randomisation not reported; CIN grades were balanced between groups, but mean age in each group not reported)	-	-	4.1:NI; 4.2:Y; 4.3:NI; 4.6:NI; 4.7:NI Some concerns (number of people changing intervention or type of analysis not reported)	5.1:N; 5.2:N; 5.3:PY; 5.4:N; 5.5:PN Some concerns (54% had less than 1y f-u)	6.1:N; 6.2:N; 6.3:NI; 6.4:PN; 6.5:PN Low RoB	7.1:NI; 7.2:PY; 7.3:N High (although f-u lasted for 2y, results during the second year not reported)	High
Lele 1984*	1.1:Y; 1.2:N; 1.4:PN; 1.6:N Serious RoB (only CIN2+ lesions were included, but 20% in CT and only 8% in CKC group were treated for CIN2; no control for age or smoking)	2.1:N; 2.4:Y Low RoB	3.1:Y; 3.2:Y; 3.3:N Low RoB	4.1:N Low RoB	5.1:Y; 5.2:PN; 5.3:PN Low RoB (10% lost to f-u, but none of them before 9m)	6.1:PN; 6.2:PY; 6.3:PY; 6.4:N Low RoB	7.1:N; 7.2:N; 7.3:N Low RoB	Serious
Ferenczy 1985*	1.1:Y; 1.2:N; 1.4:Y; 1.5:Y; 1.6:N Low RoB (alternate allocation to treatment along with matching for CIN grade; however, authors did not give details how they combined random allocation with matching)	2.1:N; 2.4:Y Low RoB	3.1:Y; 3.2:Y; 3.3:N Low RoB	4.1:N Low RoB	5.1:Y; 5.2:PN; 5.3:PN Low RoB (5% lost to f-u before 12m)	6.1:PN; 6.2:NI; 6.3:PY; 6.4:N Low RoB	7.1:N; 7.2:N; 7.3:N Low RoB	Low

Helmerhorst 1985*	1.1:NI; 1.2:NI; 1.3:N Some concerns (method of randomisation not reported)	-	-	4.1:NI; 4.2:Y; 4.3:NI; 4.6:NI; 4.7:NI Some concerns (number of people changing intervention or type of analysis not reported)	5.1:Y Low RoB	6.1:N; 6.2:N; 6.3:NI; 6.4:PN; 6.5:PN Low RoB	7.1:NI; 7.2:N; 7.3:N Some concerns	Some concerns
Hussein 1985*	1.1:Y; 1.2:N; 1.4:PN; 1.6:N Serious RoB (CIN grade distribution was similar between groups; authors also stratified results according to CIN grade, but not reported whether there was any statistically significant difference; no control for age or smoking	2.1:N; 2.4:Y Low RoB	3.1:Y; 3.2:Y; 3.3:N Low RoB	4.1:N Low RoB	5.1:NI; 5.2:PN; 5.3:PN NI about RoB	6.1:PN; 6.2:PY; 6.3:N; 6.4:N Moderate RoB (all women treated with CC underwent colposcopy at 4m; in women treated with CKC, colposcopy at 4m was performed mostly when there was doubt about complete extension; in women treated with RD, colposcopy was performed mostly in cases of extensive initial lesion)	7.1:N; 7.2:N; 7.3:N Low RoB	Serious
Kirwan 1985*	1.1:NI; 1.2:NI; 1.3:PN Some concerns (method of randomisation not reported; LA group was twice as large as CT; authors did not explicitly report that allocation ratio was 2:1, but this was probably an omission)	-	-	4.1:NI; 4.2:Y; 4.3:NI; 4.6:NI; 4.7:NI Some concerns (number of people changing intervention or type of analysis not reported)	5.1:PY Low RoB	6.1:N; 6.2:N; 6.3:NI; 6.4:PN; 6.5:PN Low RoB	7.1:NI; 7.2:N; 7.3:N Some concerns	Some concerns
Kwikkel 1985*	1.1:NI; 1.2:NI; 1.3:N Some concerns (method of randomisation not reported)	-	-	4.1:NI; 4.2:Y; 4.3:NI; 4.6:NI; 4.7:NI Some concerns (number of people changing intervention or type of analysis not reported)	5.1:Y Low RoB	6.1:N; 6.2:N; 6.3:NI; 6.4:PN; 6.5:PN Low RoB	7.1:NI; 7.2:N; 7.3:N Some concerns	Some concerns
Seshadri 1985*	1.1:Y; 1.2:N; 1.4:PN; 1.6:N Serious RoB (only CIN3 lesions were included; 45% in RD but only 29% in CKC group were <30y, but authors did not adjust for age; no control for smoking)	2.1:N; 2.4:Y Low RoB	3.1:Y; 3.2:Y; 3.3:N Low RoB	4.1:N Low RoB	5.1:N; 5.2:PN; 5.3:PN; 5.4:NI; 5.5:N Serious RoB (24% lost to f-u immediately after post-operative check-up)	6.1:PN; 6.2:PY; 6.3:PY; 6.4:N Low RoB	7.1:N; 7.2:N; 7.3:N Low RoB	Serious
Baggish 1986*	1.1:Y; 1.2:N; 1.4.N; 1.6:N Critical RoB (LA was performed more often for CIN1 and less often for CIN3; results were stratified according to CIN grade, but not reported whether there were any statistically significant differences; no control for age or smoking)	2.1:N; 2.4:Y Low RoB	3.1:Y; 3.2:Y; 3.3:N Low RoB	4.1:N Low RoB	5.1:NI; 5.2:PN; 5.3:PN NI about RoB (only women with at least 12m f-u were reported in Results; not clear whether authors excluded women with less than 12m f-u, or whether all women had indeed at least 12m f-u)	6.1:PN; 6.2:PY; 6.3:PY; 6.4:N Low RoB	7.1:N; 7.2:N; 7.3:N Low RoB	Critical

Bostofte 1986*	1.1:NI; 1.2:NI; 1.3:PN<br>Some concerns<br>(method of randomisation not reported; CIN grades were balanced between groups, but mean age in each group not reported)	-	-	4.1:NI; 4.2:Y; 4.3:NI; 4.6:NI; 4.7:NI<br>Some concerns (number of people changing intervention or type of analysis not reported)	5.1:Y<br>Low RoB	6.1:N; 6.2:N; 6.3:NI; 6.4:PN; 6.5:PN<br>Low RoB	7.1:NI; 7.2:N; 7.3:N<br>Some concerns	Some concerns
O'Shea 1986*	1.1:N; 1.2:N; 1.3:PY<br>High RoB<br>(randomisation via medical record number; higher percentage of CIN1/2 in CT group and higher percentage of CIN3 in RD group)	-	-	4.1:NI; 4.2:Y; 4.3:NI; 4.6:NI; 4.7:NI<br>Some concerns (number of people changing intervention or type of analysis not reported)	5.1:Y<br>Low RoB	6.1:N; 6.2:N; 6.3:NI; 6.4:PN; 6.5:PN<br>Low RoB	7.1:NI; 7.2:N; 7.3:N<br>Some concerns	High
Need 1988*	1.1:N; 1.2:N; 1.3:N<br>High RoB<br>(randomisation via medical record number)	-	-	4.1:NI; 4.2:Y; 4.3:NI; 4.6:NI; 4.7:NI<br>Some concerns (number of people changing intervention or type of analysis not reported)	5.1:Y<br>Low RoB	6.1:N; 6.2:N; 6.3:NI; 6.4:PN; 6.5:PN<br>Low RoB	7.1:NI; 7.2:N; 7.3:N<br>Some concerns	High
Singh 1988*	1.1:N; 1.2:N; 1.3:N<br>High RoB<br>(randomisation via hospital number)	-	-	4.1:NI; 4.2:Y; 4.3:NI; 4.6:NI; 4.7:NI<br>Some concerns (number of people changing intervention or type of analysis not reported)	5.1:Y<br>Low RoB	6.1:N; 6.2:N; 6.3:NI; 6.4:PN; 6.5:PN<br>Low RoB	7.1:NI; 7.2:N; 7.3:N<br>Some concerns	High
Partington 1989*	1.1:Y; 1.2:NI; 1.3:N<br>Some concerns<br>(patients were randomised by drawing envelopes, but method of concealment was not reported)	-	-	4.1:NI; 4.2:Y; 4.3:NI; 4.6:NI; 4.7:NI<br>Low RoB	5.1:PY<br>Low RoB<br>(7% were lost to f-u)	6.1:N; 6.2:N; 6.3:NI; 6.4:PN; 6.5:PN<br>Low RoB	7.1:NI; 7.2:N; 7.3:N<br>Some concerns	Some concerns
Yliskoski 1989*	1.1:Y; 1.2:N; 1.4:PY; 1.5:PY; 1.6:N<br>Moderate RoB<br>(patients with CIN were randomly allocated by birth date to LA or CT; a few patients with both CIN and VaIN were allocated only to LA)	2.1:N; 2.4:Y<br>Low RoB	3.1:Y; 3.2:Y; 3.3:N<br>Low RoB	4.1:N<br>Low RoB	5.1:Y; 5.2:PN; 5.3:PN<br>Low RoB<br>(2% lost to f-u)	6.1:PN; 6.2:NI; 6.3:PY; 6.4:N<br>Low RoB	7.1:N; 7.2:N; 7.3:N<br>Low RoB	Moderate
Gunasekera 1990*	1.1:N; 1.2:N; 1.3:PY<br>High RoB<br>(randomisation via hospital number; mean age differed between groups)	-	-	4.1:Y; 4.2:Y; 4.3:NI; 4.6:NI; 4.7:NI<br>Some concerns (number of people changing intervention or type of analysis not reported)	5.1:NI; 5.2:N; 5.3:PY; 5.4:NI; 5.5:PN<br>Some concerns<br>(not reported how many patients were lost to f-u)	6.1:N; 6.2:N; 6.3:NI; 6.4:PN; 6.5:PN<br>Low RoB	7.1:NI; 7.2:N; 7.3:N<br>Some concerns	High
Hellberg 1990*	1.1:Y; 1.2:N; 1.4:PN; 1.6:N<br>Serious RoB<br>(mostly CIN3 lesions were included, but the exact percentage of CIN3 lesions was 96·5% in CKC and 83·7% in CT group; no control for age or smoking)	2.1:N; 2.4:Y<br>Low RoB	3.1:Y; 3.2:Y; 3.3:N<br>Low RoB	4.1:N<br>Low RoB	5.1:Y; 5.2:PN; 5.3:PN<br>Low RoB<br>(all patients had at least 6m f-u; records of patients who moved to other part of country were retrieved from their new hospital)	6.1:PN; 6.2:PY; 6.3:PY; 6.4:N<br>Low RoB	7.1:N; 7.2:N; 7.3:N<br>Low RoB	Serious

Tabor 1990*	1.1:Y; 1.2:N; 1.4:PY; 1.5:PY; 1.6:N<br>Moderate RoB<br>(CIN grades did not differ between LC and CKC; no control for age or smoking; because authors reported that choice of treatment was based on year of treatment, RoB was considered to be moderate)	2.1:N; 2.4:Y<br>Low RoB	3.1:Y; 3.2:Y; 3.3:N<br>Low RoB	4.1:N<br>Low RoB	5.1:Y; 5.2:PN; 5.3:PN<br>Low RoB	6.1:PN; 6.2:PY; 6.3:PY; 6.4:N<br>Low RoB	7.1:N; 7.2:N; 7.3:N<br>Low RoB	Moderate
Berget 1991*	1.1:NI; 1.2:NI; 1.3:N<br>Some concerns<br>(method of randomisation not reported)	-	-	4.1:NI; 4.2:Y; 4.3:NI; 4.6:NI; 4.7:NI<br>Some concerns (number of people changing intervention or type of analysis not reported)	5.1:Y<br>Low RoB	6.1:N; 6.2:N; 6.3:NI; 6.4:PN; 6.5:PN<br>Low RoB	7.1:NI; 7.2:N; 7.3:N<br>Some concerns	Some concerns
Goodman 1991*	1.1:N; 1.2:N; 1.3:N<br>High RoB<br>(randomisation via alternation)	-	-	4.1:NI; 4.2:Y; 4.3:N; 4.6:N; 4.7:N<br>Some concerns<br>(two patients were allocated to CC but underwent LA because they wanted for their intrauterine contraceptive device to remain in situ; authors performed an as-treated analysis, but because only two patients changed intervention, bias is probably not significant)	5.1:PN; 5.2:N; 5.3:PY; 5.4:N; 5.5:PN<br>Some concerns<br>(15% lost to f-u)	6.1:N; 6.2:N; 6.3:NI; 6.4:PN; 6.5:PN<br>Low RoB	7.1:NI; 7.2:N; 7.3:N<br>Some concerns	High
Martel 1992*	1.1:Y; 1.2:N; 1.4:PN; 1.6:N<br>Serious RoB<br>(only CIN3 lesions were included; no control for age or smoking)	2.1:N; 2.4:Y<br>Low RoB	3.1:Y; 3.2:Y; 3.3:N<br>Low RoB	4.1:N<br>Low RoB	5.1:PN; 5.2:PN; 5.3:PN; 5.4:NI; 5.5:N<br>Moderate RoB<br>(12% lost to f-u)	6.1:PN; 6.2:PY; 6.3:PY; 6.4:N<br>Low RoB	7.1:N; 7.2:N; 7.3:N<br>Low RoB	Serious
Guijon 1993*	1.1:Y; 1.2:N; 1.4:NI; 1.6:N<br>Moderate RoB<br>(age did not affect risk of recurrence; CIN grade affected risk of recurrence; no control for smoking; because authors reported that selection of treatment was “random”, RoB was considered to be moderate)	2.1:N; 2.4:Y<br>Low RoB	3.1:Y; 3.2:Y; 3.3:N<br>Low RoB	4.1:N<br>Low RoB	5.1:NI; 5.2:PN; 5.3:PN<br>NI about RoB<br>(prospective cohort; patients who were not expected to comply with f-u were excluded; not reported how many were excluded for this reason or how many of the included patients were lost to f-u)	6.1:PN; 6.2:PY; 6.3:PY; 6.4:N<br>Low RoB	7.1:N; 7.2:N; 7.3:N<br>Low RoB	NI (probably moderate)
Oyesanya 1993 (cohort study)*	1.1:Y; 1.2:N; 1.4:PY; 1.5:Y; 1.6:N<br>Moderate RoB<br>(matching for age and severity of cytology; no control for smoking)	2.1:N; 2.4:Y<br>Low RoB	3.1:Y; 3.2:Y; 3.3:N<br>Low RoB	4.1:N<br>Low RoB	5.1:PY; 5.2:PN; 5.3:PN<br>Moderate RoB<br>(all patients attended at least one f-u visit, but not reported how many attended more than one f-u visits)	6.1:PN; 6.2:PY; 6.3:PY; 6.4:N<br>Low RoB	7.1:N; 7.2:N; 7.3:N<br>Low RoB	Moderate
Oyesanya 1993 (RCT)*	1.1:Y; 1.2:Y; 1.3:N<br>Low RoB	-	-	4.1:NI; 4.2:Y; 4.3:NI; 4.6:NI; 4.7:NI<br>Some concerns (number of people changing intervention or type of analysis not reported)	5.1:Y<br>Low RoB	6.1:N; 6.2:N; 6.3:NI; 6.4:PN; 6.5:PN<br>Low RoB	7.1:NI; 7.2:N; 7.3:N<br>Some concerns	Some concerns

Alvarez 1994*	1.1:Y; 1.2:Y; 1.3:N<br>Low RoB	-	-	4.1:NI; 4.2:Y; 4.3:N; 4.6:N; 4.7:Y<br>High RoB<br>(patients randomised to LLETZ were treated without prior PB; patients randomised to LA underwent PB and were treated only when indicated based on PB findings, meaning that 63% of patients randomised to LA were excluded)	5.1:N; 5.2:N; 5.3:PY; 5.4:NI; 5.5:PN<br>Some concerns<br>(31% lost to f-u at 3m; 61% lost to f-u at 6m)	6.1:N; 6.2:N; 6.3:NI; 6.4:PN; 6.5:PN<br>Low RoB	7.1:NI; 7.2:N; 7.3:N<br>Some concerns	High
Kuppers 1994	1.1:Y; 1.2:N; 1.4:N; 1.6:N<br>Critital RoB<br>(no control for confounders)	2.1:N; 2.4:Y<br>Low RoB	3.1:Y; 3.2:Y; 3.3:N<br>Low RoB	4.1:N<br>Low RoB	5.1:PY; 5.2:PN; 5.3:PN<br>Low RoB<br>(all ten patients were followed-up for at least a year)	6.1:PN; 6.2:PY; 6.3:Y; 6.4:N<br>Low RoB	7.1:N; 7.2:N; 7.3:N<br>Low RoB	Critical
Sideri 1994*	1.1:Y; 1.2:N; 1.4:PN; 1.6:N<br>Serious RoB<br>(only biopsy-proven CIN3 lesions have been included, but micro-invasion was diagnosed on 6% of CKC and only 0·8% of LLETZ cones; no control for age or smoking)	2.1:N; 2.4:Y<br>Low RoB	3.1:Y; 3.2:Y; 3.3:N<br>Low RoB	4.1:N<br>Low RoB	5.1:NI; 5.2:PN; 5.3:PN<br>NI about RoB<br>(patients with less than 6m f-u were excluded, but not reported how many were excluded)	6.1:PN; 6.2:PY; 6.3:PY; 6.4:N<br>Low RoB	7.1:N; 7.2:N; 7.3:N<br>Low RoB	Serious
Diakomanolis 1995*	1.1:Y; 1.2:N; 1.4:PN; 1.6:N<br>Serious RoB<br>(only LSIL lesions have been included; no control for age or smoking)	2.1:N; 2.4:Y<br>Low RoB	3.1:Y; 3.2:Y; 3.3:N<br>Low RoB	4.1:N<br>Low RoB	5.1:Y; 5.2:PN; 5.3:PN<br>Low RoB<br>(f-u ranged from 42 to 76m)	6.1:PN; 6.2:PY; 6.3:PY; 6.4:N<br>Low RoB	7.1:N; 7.2:N; 7.3:N<br>Low RoB	Serious
Baldauf 1996*	1.1:Y; 1.2:N; 1.4:PY; 1.5:Y; 1.6:N<br>Moderate RoB<br>(women treated with LC had in general higher rates of CIN2+ than LLETZ, but difference was not statistically significant; no difference in age between groups; no control for smoking)	2.1:N; 2.4:Y<br>Low RoB	3.1:Y; 3.2:Y; 3.3:N<br>Low RoB	4.1:N<br>Low RoB	5.1:Y; 5.2:PN; 5.3:PN<br>Low RoB<br>(5% were excluded because they were lost to f-u or were treated just before the end of the study period)	6.1:PN; 6.2:PY; 6.3:PY; 6.4:N<br>Low RoB	7.1:N; 7.2:N; 7.3:N<br>Low RoB	Moderate
Santos 1996*	1.1:NI; 1.2:NI; 1.3:N<br>Some concerns<br>(method of randomisation not reported)	-	-	4.1:NI; 4.2:Y; 4.3:NI; 4.6:NI; 4.7:NI<br>Some concerns (number of people changing intervention or type of analysis not reported)	5.1:PN; 5.2:N; 5.3:PY; 5.4:NI; 5.5:PN<br>Some concerns<br>(mean f-u was less than 1y, although protocol specified a 24-month f-u duration)	6.1:N; 6.2:N; 6.3:NI; 6.4:PN; 6.5:PN<br>Low RoB	7.1:NI; 7.2:N; 7.3:N<br>Some concerns	Some concerns
Urbaniak 1996*	1.1:Y; 1.2:N; 1.4:N; 1.5:N<br>Critical RoB<br>(patients treated with LC had more severe CIN grades; age distribution was not comparable between groups; no control for smoking)	2.1:N; 2.4:Y<br>Low RoB	3.1:Y; 3.2:Y; 3.3:N<br>Low RoB	4.1:N<br>Low RoB	5.1:Y; 5.2:PN; 5.3:PN<br>Low RoB<br>(3% lost to f-u at 4-6m)	6.1:PN; 6.2:PY; 6.3:PY; 6.4:N<br>Low RoB	7.1:N; 7.2:N; 7.3:N<br>Low RoB	Critical

Varawalla 1996*	1.1:Y; 1.2:N; 1.4:PY; 1.5:Y; 1.6:N Moderate RoB (age did not affect risk of recurrence; percentage of CIN1 was slightly higher in CT group than LA/LLETZ, and percentage of CIN3 was slightly lower in CT than LA/LLETZ; CIN grade affected risk of recurrence only in CT group; no control for smoking)	2.1:N; 2.4:Y Low RoB	3.1:Y; 3.2:Y; 3.3:N Low RoB	4.1:N Low RoB	5.1:Y; 5.2:PN; 5.3:PN Low RoB (<5% had less than 1y f-u)	6.1:PN; 6.2:PY; 6.3:PY; 6.4:N Low RoB	7.1:N; 7.2:N; 7.3:N Low RoB	Moderate
Widrich 1996*	1.1:Y; 1.2:N; 1.4:PN; 1.5:N Serious RoB (only AIS lesions have been included; no control for age or smoking)	2.1:N; 2.4:Y Low RoB	3.1:Y; 3.2:Y; 3.3:N Low RoB	4.1:N Low RoB	5.1:NI; 5.2:PN; 5.3:PN NI about RoB	6.1:PN; 6.2:PY; 6.3:PY; 6.4:N Low RoB	7.1:N; 7.2:N; 7.3:N Low RoB	Serious
Wolf 1996*	1.1:Y; 1.2:N; 1.4:PN; 1.5:N Serious RoB (only AIS lesions have been included; no control for age or smoking)	2.1:N; 2.4:Y Low RoB	3.1:Y; 3.2:Y; 3.3:N Low RoB	4.1:N Low RoB	5.1:N; 5.2:NI; 5.3:NI; 5.4:NI; 5.5:N Serious RoB (35% were excluded because of incomplete clinical or pathological data)	6.1:PN; 6.2:PY; 6.3:PY; 6.4:N Low RoB	7.1:N; 7.2:N; 7.3:N Low RoB	Serious
Gonzalez-Bosquet 1997*	1.1:Y; 1.2:N; 1.4:PN; 1.6:N Serious RoB (women with CIN1/2 were randomly treated with LA or LLETZ; women with CIN3 were treated with LA, LLETZ or CKC; CIN grade did not affect the risk of recurrence for women treated with LA or LLETZ; no control for age or smoking	2.1:N; 2.4:Y Low RoB	3.1:Y; 3.2:Y; 3.3:N Low RoB	4.1:N Low RoB	5.1:Y; 5.2:PN; 5.3:PN Low RoB (4% lost to f-u)	6.1:PN; 6.2:PY; 6.3:PY; 6.4:N Low RoB	7.1:N; 7.2:N; 7.3:N Low RoB	Serious
Mitchell 1998*	1.1:Y; 1.2:Y; 1.3:N Low RoB	-	-	4.1:NI; 4.2:Y; 4.3:N; 4.6:N; 4.7:N Some concerns (one patient did not receive allocated treatment by mistake; authors performed an per-protocol analysis, but because only one patient changed intervention, bias is probably not significant)	5.1:N; 5.2:N; 5.3:PY; 5.4:NI; 5.5:PN Some concerns (28% lost to f-u)	6.1:N; 6.2:PN; 6.3:NI; 6.4:PN; 6.5:PN Low RoB (pathologists were blinded, but not reported whether colposcopists were blinded as well)	7.1:PN; 7.2:N; 7.3:N Some concerns (In 'Materials and Methods' authors report that f-u was up to 24m, but in 'Results' f-u was up to 37m)	Some concerns
Simmons 1998*	1.1:Y; 1.2:N; 1.4:PN; 1.5:N Serious RoB (more patients treated with CKC were diagnosed with invasion on cone compared to LLETZ; no control for age or smoking)	2.1:N; 2.4:Y Low RoB	3.1:Y; 3.2:Y; 3.3:N Low RoB	4.1:N Low RoB	5.1:N; 5.2:PN; 5.3:PN; 5.4:Y; 5.5:N Critical RoB (45% lost to f-u: 44% in LLETZ and 46% in CKC group)	6.1:PN; 6.2:PY; 6.3:PY; 6.4:N Low RoB	7.1:N; 7.2:N; 7.3:N Low RoB	Critical

Bornstein 1999*	1.1:Y; 1.2:N; 1.4:PN; 1.6:N Serious RoB (only women with HSIL cytology have been included, but women with biopsy-proven CIN3 were treated more often with CKC than LLETZ/LA, and women with biopsy-proven CIN2 were treated more often with LLETZ/LA than CKC; no control for age or smoking)	2.1:N; 2.4:Y Low RoB	3.1:Y; 3.2:Y; 3.3:N Low RoB	4.1:N Low RoB	5.1:NI; 5.2:PN; 5.3:PN NI about RoB	6.1:PN; 6.2:PY; 6.3:PY; 6.4:N Low RoB	7.1:N; 7.2:N; 7.3:N Low RoB	Serious
Duggan 1999*	1.1:Y; 1.2:NI; 1.3:N Some concerns (computer-generated randomisation but not reported whether concealed)	-	-	4.1:NI; 4.2:Y; 4.3:N; 4.6:Y Low RoB	5.1:PN; 5.2:N; 5.3:PY; 5.4:N; 5.5:PN Some concerns (11% lost to f-u)	6.1:N; 6.2:N; 6.3:NI; 6.4:PN; 6.5:PN Low RoB	7.1:NI; 7.2:N; 7.3:N Some concerns	Some concerns
Giacalone 1999*	1.1:Y; 1.2:Y; 1.3:N Low RoB	-	-	4.1:NI; 4.2:Y; 4.3:NI; 4.6:NI; 4.7:NI Some concerns (number of people changing intervention or type of analysis not reported)	5.1:PN; 5.2:N; 5.3:PY; 5.4:NI; 5.5:PN Some concerns (15% lost to f-u)	6.1:N; 6.2:N; 6.3:N Low RoB	7.1:NI; 7.2:N; 7.3:N Some concerns	Some concerns
Ioffe 1999*	1.1:Y; 1.2:N; 1.4:N; 1.6:N Critical RoB (no control for confounding factors)	2.1:N; 2.4:Y Low RoB	3.1:Y; 3.2:Y; 3.3:N Low RoB	4.1:N Low RoB	5.1:PY; 5.2:PN; 5.3:PN; 5.4:NI; 5.5:N Moderate RoB (19% lost to f-u)	6.1:PN; 6.2:PY; 6.3:PY; 6.4:N Low RoB	7.1:N; 7.2:N; 7.3:N Low RoB	Critical
Takac 1999*	1.1:NI; 1.2:NI; 1.3:N Some concerns (method of randomisation not reported)	-	-	4.1:NI; 4.2:Y; 4.3:NI; 4.6:NI; 4.7:NI Some concerns (number of people changing intervention or type of analysis not reported)	5.1:Y Low RoB	6.1:N; 6.2:PN; 6.3:NI; 6.4:PN; 6.5:PN Low RoB	7.1:PN; 7.2:N; 7.3:N Some concerns (described f-u scheme differs between ‘Materials and Methods’ and ‘Results’)	Some concerns
Vejerslev_A 1999*	1.1:Y; 1.2:NI; 1.3:PN Some concerns (computer-generated randomisation but not reported whether concealed)	-	-	4.1:NI; 4.2:Y; 4.3:NI; 4.6:NI; 4.7:NI Some concerns (number of people changing intervention or type of analysis not reported)	5.1:Y Low RoB	6.1:N; 6.2:N; 6.3:NI; 6.4:PN; 6.5:PN Low RoB	7.1:NI; 7.2:N; 7.3:N Some concerns	Some concerns
Vejerslev_B 1999*	1.1:Y; 1.2:NI; 1.3:N Some concerns (computer-generated randomisation but not reported whether concealed)	-	-	4.1:NI; 4.2:Y; 4.3:NI; 4.6:NI; 4.7:NI Some concerns (number of people changing intervention or type of analysis not reported)	5.1:PN; 5.2:N; 5.3:PY; 5.4:Y; 5.5:PN Some concerns (14% lost to f-u at 6m: 2% in LLETZ and 25% in LC group)	6.1:N; 6.2:N; 6.3:NI; 6.4:PN; 6.5:PN Low RoB	7.1:NI; 7.2:N; 7.3:N Some concerns	Some concerns
Husseinzadeh 2000*	1.1:Y; 1.2:N; 1.4:PN; 1.6:N Critical RoB (referral cytology was HSIL in 97% of women treated with CKC, but only in 77% of women treated with LLETZ; no control for age or smoking)	2.1:N; 2.4:Y Low RoB	3.1:Y; 3.2:Y; 3.3:N Low RoB	4.1:N Low RoB	5.1:PY; 5.2:PN; 5.3:PN Low RoB (8% lost to f-u)	6.1:PN; 6.2:PY; 6.3:PY; 6.4:N Low RoB	7.1:N; 7.2:N; 7.3:N Low RoB	Critical

Persad 2001*	1.1:Y; 1.2:N; 1.4:N; 1.6:N<br>Critical RoB<br>(CT mostly for CIN1 and LA for CIN2/3 lesions; in women with CIN1, age differed between treatment groups, but age did not differ in women with CIN2 or CIN3; no control for smoking)	2.1:N; 2.4:Y<br>Low RoB	3.1:Y; 3.2:Y; 3.3:N<br>Low RoB	4.1:N<br>Low RoB	5.1:Y; 5.2:PN; 5.3:PN<br>Low RoB<br>(2% lost to f-u)	6.1:PN; 6.2:PY; 6.3:PY; 6.4:N<br>Low RoB	7.1:N; 7.2:N; 7.3:N<br>Low RoB	Critical
Dey 2002*	1.1:Y; 1.2:Y; 1.3:N<br>Low RoB	-	-	4.1:NI; 4.2:Y; 4.3:N; 4.6:Y<br>Low RoB	5.1:Y<br>Low RoB	6.1:N; 6.2:PN; 6.3:NI; 6.4:PN; 6.5:PN<br>Low RoB	7.1:NI; 7.2:N; 7.3:N<br>Some concerns	Some concerns
Mathevet 2003*	1.1:Y; 1.2:NI; 1.3:N<br>Some concerns<br>(randomisation was performed by drawing envelops, but not reported whether envelops were opaque or concealed in a box)	-	-	4.1:NI; 4.2:Y; 4.3:NI; 4.6:NI; 4.7:NI<br>Some concerns (number of people changing intervention or type of analysis not reported)	5.1:Y<br>Low RoB	6.1:N; 6.2:PN; 6.3:NI; 6.4:PN; 6.5:PN<br>Low RoB	7.1:NI; 7.2:N; 7.3:N<br>Some concerns	Some concerns
Omnes 2003*	1.1:Y; 1.2:N; 1.4:PN; 1.6:N<br>Serious RoB<br>(only CIN2+ lesions have been included; no control for age or smoking)	2.1:N; 2.4:Y<br>Low RoB	3.1:Y; 3.2:Y; 3.3:N<br>Low RoB	4.1:N<br>Low RoB	5.1:NI; 5.2:PN; 5.3:PN<br>NI about RoB	6.1:PN; 6.2:PY; 6.3:PY; 6.4:N<br>Low RoB	7.1:N; 7.2:N; 7.3:N<br>Low RoB	Serious
Zielisnki 2003*	1.1:Y; 1.2:N; 1.4:PN; 1.6:N<br>Serious RoB<br>(only CIN3 lesions have been included; no control for age or smoking)	2.1:N; 2.4:Y<br>Low RoB	3.1:Y; 3.2:Y; 3.3:N<br>Low RoB	4.1:N<br>Low RoB	5.1:NI; 5.2:PN; 5.3:Y; 5.4:NI; 5.5:N<br>NI about RoB<br>(not reported how many patients were lost to f-u; three patients without post-treatment HPV testing were excluded)	6.1:PN; 6.2:PY; 6.3:PY; 6.4:N<br>Low RoB	7.1:N; 7.2:N; 7.3:N<br>Low RoB	Serious
Murta 2004*	1.1:Y; 1.2:N; 1.4:PN; 1.6:N<br>Serious RoB<br>(only CIN2+ lesions have been included; no control for age or smoking)	2.1:N; 2.4:Y<br>Low RoB	3.1:Y; 3.2:Y; 3.3:N<br>Low RoB	4.1:N<br>Low RoB	5.1:N; 5.2:PN; 5.3:PN; 5.4:N; 5.5:N<br>Serious RoB<br>(48% had less than 2y f-u and were excluded: 56% in CKC and 30% in LLETZ group)	6.1:PN; 6.2:PY; 6.3:PY; 6.4:N<br>Low RoB	7.1:N; 7.2:N; 7.3:N<br>Low RoB	Critical
Lu 2006	1.1:Y; 1.2:N; 1.4:PN; 1.6:Y<br>Serious RoB<br>(only CIN3 lesions have been included; authors adjusted for age, but we used the unadjusted data, because in the multivariate analysis there was also adjustment for endocervical margin status, i.e. for a post-intervention factor that might be correlated with cone length; no control for smoking)	2.1:N; 2.4:Y<br>Low RoB	3.1:Y; 3.2:Y; 3.3:N<br>Low RoB	4.1:N<br>Low RoB	5.1:N; 5.2:PN; 5.3:Y; 5.4:NI; 5.5:N<br>Serious RoB<br>(33% were excluded because of missing f-u data, 0·8% because of fragmentation; and 0·5% because of missing ECC data)	6.1:PN; 6.2:PY; 6.3:PY; 6.4:N<br>Low RoB	7.1:N; 7.2:N; 7.3:N<br>Low RoB	Serious
Dalrymple 2008*	1.1:Y; 1.2:N; 1.4:PN; 1.6:N<br>Serious RoB<br>(only AIS+ lesions have been included, and the difference in invasive lesions between groups was not statistically significant; no control for age or smoking)	2.1:N; 2.4:Y<br>Low RoB	3.1:Y; 3.2:Y; 3.3:N<br>Low RoB	4.1:N<br>Low RoB	5.1:PY; 5.2:PN; 5.3:PN<br>Low RoB<br>(f-u was through hospital records and population-based registries; 8.5% lost to f-u)	6.1:PN; 6.2:PY; 6.3:PY; 6.4:N<br>Low RoB	7.1:N; 7.2:N; 7.3:N<br>Low RoB	Serious

Park 2008*	1.1:Y; 1.2:N; 1.4:PY; 1.5:Y; 1.6:N Moderate RoB (age or CIN grade did not affect risk of positive HPV testing after treatment; no control for smoking)	2.1:N; 2.4:Y Low RoB	3.1:Y; 3.2:Y; 3.3:N Low RoB	4.1:N Low RoB	5.1:NI; 5.2:PN; 5.3:NI NI about RoB (patients without HPV testing after treatment were excluded, but not reported how many were excluded; there is no suspicion that this percentage might have differed between groups)	6.1:PN; 6.2:PY; 6.3:PN; 6.4:N Moderate RoB (f-u after 3-6m was personalised according to CIN grade and previous f-u results)	7.1:N; 7.2:N; 7.3:N Low RoB	NI (probably moderate)
Gallwas 2010	1.1:Y; 1.2:N; 1.4:N; 1.6:N Critical RoB (no control for confounding factors)	2.1:N; 2.4:Y Low RoB	3.1:Y; 3.2:Y; 3.3:N Low RoB	4.1:N Low RoB	5.1:NI; 5.2:PN; 5.3:PN NI about RoB	6.1:PN; 6.2:PY; 6.3:PN; 6.4:N Moderate RoB (date of first HPV DNA test varied from 1 to 10.7m after treatment)	7.1:N; 7.2:N; 7.3:N Low RoB	Critical
Ostojic 2010*	1.1:Y; 1.2:N; 1.4:N; 1.6:N Critical RoB (no control for confounding factors)	2.1:N; 2.4:Y Low RoB	3.1:Y; 3.2:Y; 3.3:N Low RoB	4.1:N Low RoB	5.1:N; 5.2:PN; 5.3:PN; 5.4:NI; 5.5:N Serious RoB (14% had no f-u visits; 42% did not comply with full f-u schedule)	6.1:PN; 6.2:PY; 6.3:PY; 6.4:N Low RoB	7.1:N; 7.2:N; 7.3:N Low RoB	Critical
Ang 2011	1.1:Y; 1.2:N; 1.4:PN; 1.6:Y Serious RoB (only CIN2+ lesions have been included; authors adjusted for age, but we used the unadjusted data, because in the multivariate analysis there was also adjustment for endocervical margin status, i.e. for a post-intervention factor that might be correlated with cone length; no control for smoking)	2.1:N; 2.4:Y Low RoB	3.1:Y; 3.2:Y; 3.3:N Low RoB	4.1:N Low RoB	5.1:PY; 5.2:NI; 5.3:PN NI about RoB (3% were lost to f-u and 4% were excluded because of uncertain margin status; patients with missing cone length were excluded, but not reported how many these patients were)	6.1:PN; 6.2:PY; 6.3:PY; 6.4:N Low RoB	7.1:N; 7.2:N; 7.3:N Low RoB	Serious
Kocken 2011*	1.1:Y; 1.2:N; 1.4:PN; 1.6:N Serious RoB (CIN grade did not affect risk of recurrence; risk of recurrence was greater for smokers than non-smokers, but not reported whether this difference was statistically significant; no control for age)	2.1:N; 2.4:Y Low RoB	3.1:Y; 3.2:Y; 3.3:N Low RoB	4.1:N Low RoB	5.1:PY; 5.2:PN; 5.3:PN Low RoB (because ascertainment of outcome was through hospital records and population-based registries, RoB is probably low)	6.1:PN; 6.2:PY; 6.3:PY; 6.4:N Low RoB	7.1:N; 7.2:N; 7.3:N Low RoB	Serious
Kietpeerakool 2012*	1.1:Y; 1.2:N; 1.4:PN; 1.6:N Serious RoB (only women with AIS have been included; no control for age or smoking)	2.1:N; 2.4:Y Low RoB	3.1:Y; 3.2:Y; 3.3:N Low RoB	4.1:N Low RoB	5.1:Y; 5.2:PN; 5.3:PN Low RoB (2% lost to f-u)	6.1:PN; 6.2:PY; 6.3:Y; 6.4:N Low RoB	7.1:N; 7.2:N; 7.3:N Low RoB	Serious
Serati 2012*	1.1;Y; 1.2:N; 1.4:Y; 1.5:Y; 1.6:Y Moderate RoB (in univariate analysis, age was found to be marginally statistically significant, while smoking and CIN grade were not, therefore age was added in the multi-variate analysis; however, we did not use the adjusted HRs of the multivariate analysis, because authors adjusted for margin status as well)	2.1:N; 2.4:Y Low RoB	3.1:Y; 3.2:Y; 3.3:N Low RoB	4.1:N Low RoB	5.1:Y; 5.2:PN; 5.3:PN Low RoB (0·7% lost to f-u before 6m)	6.1:PN; 6.2:PY; 6.3:Y; 6.4:N Low RoB	7.1:N; 7.2:N; 7.3:N Low RoB	Moderate

van Hanegem 2012*	1.1:Y; 1.2:N; 1.4:PY; 1.5:Y; 1.6:N Moderate RoB (only women ≤30y with were included; no control for smoking)	2.1:N; 2.4:Y Low RoB	3.1:Y; 3.2:Y; 3.3:N Low RoB	4.1:N Low RoB	5.1:N; 5.2:PN; 5.3:PN; 5.4:NI; 5.5:N Serious RoB (16% lost to f-u before 3m; 39% lost to f-u before 1y)	6.1:PN; 6.2:PY; 6.3:PY; 6.4:N Low RoB	7.1:N; 7.2:N; 7.3:N Low RoB	Serious
Zeng 2012*	1.1:Y; 1.2:N; 1.4:N; 1.6:N Critical RoB (no control for confounding factors)	2.1:N; 2.4:Y Low RoB	3.1:Y; 3.2:Y; 3.3:N Low RoB	4.1:N Low RoB	5.1:PY; 5.2:PN; 5.3:PN Low RoB (6·5% lost to f-u)	6.1:PN; 6.2:PY; 6.3:PY; 6.4:N Low RoB	7.1:N; 7.2:N; 7.3:N Low RoB	Critical
Taylor 2014*	1.1:Y; 1.2:N; 1.4:PN; 1.6:N Serious RoB (only women with AIS have been included; no control for age or smoking)	2.1:N; 2.4:Y Low RoB	3.1:Y; 3.2:Y; 3.3:N Low RoB	4.1:N Low RoB	5.1:PY; 5.2:PN; 5.3:PN Low RoB (8% lost to f-u)	6.1:PN; 6.2:PY; 6.3:NI; 6.4:N Moderate RoB (f-u was at varying intervals, with no other details)	7.1:N; 7.2:N; 7.3:N Low RoB	Serious
Babkina 2015	1.1:Y; 1.2:N; 1.4:PY; 1.5:Y; 1.6:N Moderate RoB (all patients had been treated for CIN2+; older and multiparous women had a higher risk of recurrence but not at statistical significance; smoking was not associated with an increased risk of recurrence)	2.1:N; 2.4:Y Low RoB	3.1:Y; 3.2:Y; 3.3:N Low RoB	4.1:N Low RoB	5.1:Y; 5.2:PN; 5.3:PN Low RoB (8% were lost to f-u)	6.1:PN; 6.2:PY; 6.3:Y; 6.4:N Low RoB	7.1:N; 7.2:N; 7.3:N Low RoB	Moderate
Cai 2015*	1.1:Y; 1.2:N; 1.4:PY; 1.5:Y; 1.6:N Moderate RoB (CIN grades and age were similar between groups; no control for smoking)	2.1:N; 2.4:Y Low RoB	3.1:Y; 3.2:Y; 3.3:N Low RoB	4.1:N Low RoB	5.1:NI; 5.2:PN; 5.3:PN NI about RoB	6.1:PN; 6.2:PY; 6.3:PY; 6.4:N Low RoB	7.1:PY; 7.2:N; 7.3:N Serious RoB (although in methodology authors describe that cytology was performed at 12m, cytology results are given at 6m)	Serious
Kiuchi 2016*	1.1:Y; 1.2:N; 1.4:N; 1.6:N Critical RoB (no control for confounding factors)	2.1:N; 2.4:Y Low RoB	3.1:Y; 3.2:Y; 3.3:N Low RoB	4.1:N Low RoB	5.1:PN; 5.2:PN; 5.3:PN; 5.4:NI; 5.5:N Serious RoB (24% of LLETZ were excluded because of post-operative diagnosis of cancer or less than 12m f-u, but not reported how many women were excluded for each reason; NI for LC)	6.1:PN; 6.2:PY; 6.3:PY; 6.4:N Moderate RoB (not totally clear whether patients treated with LC followed the same f-u schedule as patients treated with LLETZ)	7.1:N; 7.2:N; 7.3:PY Serious RoB (some results or baseline characteristics are reported only for LLETZ and not for LC)	Critical
Mariya 2016*	1.1:Y; 1.2:N; 1.4:PN; 1.6:Y Serious RoB (only women with CIN3 have been included; although an adjusted RR for age and post-treatment HPV positivity is provided for long-term recurrence rates, this was not used because it did not consider the short-term recurrence rates; no control for smoking)	2.1:N; 2.4:Y Low RoB	3.1:Y; 3.2:Y; 3.3:N Low RoB	4.1:N Low RoB	5.1:PY; 5.2:PN; 5.3:Y; 5.4:NI; 5.5:N Moderate RoB (4% had less than 5m f-u, but long-term drop rates not reported; 8% were excluded because of missing information on HPV genotype)	6.1:PN; 6.2:PY; 6.3:Y; 6.4:N Low RoB	7.1:N; 7.2:N; 7.3:N Low RoB	Serious

Hansen 2017*	1.1:Y; 1.2:N; 1.4:N; 1.6:N<br>Critical RoB<br>(no control for confounding factors; LA was performed more often for CIN1/2 and less often for CIN3 than LLETZ)	2.1:N; 2.4:Y<br>Low RoB	3.1:Y; 3.2:Y; 3.3:N<br>Low RoB	4.1:N<br>Low RoB	5.1:NI; 5.2:PN; 5.3:PN<br>NI about RoB	6.1:PN; 6.2:PY; 6.3:Y; 6.4:N<br>Low RoB	7.1:N; 7.2:N; 7.3:N<br>Low RoB	Critical
Papoutsis 2017*	1.1:Y; 1.2:N; 1.4:PN; 1.6:N<br>Serious RoB<br>(although authors adjusted for important confounding factors, they also adjusted for highly correlated variables (e.g. for both pre-treatment cytology and colposcopy); due to existence of collinearity and potential inflation of variance, we downgraded to 'Serious')	2.1:N; 2.4:Y<br>Low RoB	3.1:Y; 3.2:Y; 3.3:N<br>Low RoB	4.1:N<br>Low RoB	5.1:PN; 5.2:PN; 5.3:NI; 5.4:N; 5.5:N<br>Serious RoB<br>(14% had at least one f-u visit but were lost at 12m: 24% in LLETZ and 5% in CC group; percentage of women with no f-u visits not reported)	6.1:PN; 6.2:PY; 6.3:Y; 6.4:N<br>Low RoB	7.1:N; 7.2:N; 7.3:N<br>Low RoB	Serious
Smith 2017	1.1:N; 1.2:PN; 1.3:PY<br>High RoB<br>(randomisation via alternation; HIV viral load differed between groups)	-	-	4.1:PY; 4.2:Y; 4.3:N; 4.6:Y<br>Low RoB	5.1:Y<br>Low RoB<br>(7% were lost to f-u at 6m, with equal rates between group)	6.1:N; 6.2:N; 6.3:PY; 6.4:PN<br>Low RoB	7.1:Y; 7.2:N; 7.3:N<br>Low RoB	High
Wyse 2017*	1.1:Y; 1.2:N; 1.4:N; 1.6:N<br>Critical RoB<br>(CC was compared to a random sample of LLETZ, without controlling for confounders)	2.1:N; 2.4:Y<br>Low RoB	3.1:Y; 3.2:Y; 3.3:N<br>Low RoB	4.1:N<br>Low RoB	5.1:NI; 5.2:NI; 5.3:NI<br>NI about RoB	6.1:PN; 6.2:PY; 6.3:Y; 6.4:N<br>Low RoB	7.1:N; 7.2:N; 7.3:N<br>Low RoB	Critical
Byun 2018*	1.1:Y; 1.2:N; 1.4:PY; 1.5:Y; 1.6:N<br>Moderate RoB<br>(only CIN2+ lesions have been included; age did not affect risk of recurrence; no control for smoking)	2.1:N; 2.4:Y<br>Low RoB	3.1:Y; 3.2:Y; 3.3:N<br>Low RoB	4.1:N<br>Low RoB	5.1:PN; 5.2:PN; 5.3:Y; 5.4:NI; 5.5:N<br>Serious RoB<br>(20% were lost to f-u and were excluded; an additional 26% were excluded because HPV testing was not performed before and after treatment)	6.1:PN; 6.2:PY; 6.3:Y; 6.4:N<br>Low RoB	7.1:N; 7.2:N; 7.3:N<br>Low RoB	Serious
Greene 2019	1.1:Y; 1.2:N; 1.3:N<br>Low RoB	-	-	4.1:Y; 4.2:Y; 4.3:N; 4.6:Y<br>Low RoB	5.1:Y<br>Low RoB<br>(7·5% were lost to f-u, withdrew consent or died: 9% in LLETZ group and 6% in CT group)	6.1:N; 6.2:N; 6.3:N<br>Low RoB<br>(colposcopists were aware of the intervention, whilst pathologists were not)	7.1:Y; 7.2:N; 7.3:N<br>Low RoB	Low
Bogani 2020*	1.1:Y; 1.2:N; 1.4:PY; 1.5:Y; 1.6:N<br>Moderate RoB<br>(a propensity-matched analysis was performed; age and CIN grade did not differ between groups; no control for smoking)	2.1:N; 2.4:Y<br>Low RoB	3.1:Y; 3.2:Y; 3.3:N<br>Low RoB	4.1:N<br>Low RoB	5.1:Y; 5.2:PN; 5.3:PN<br>Low RoB<br>(only 1·3% were excluded due to f-u less than 5y)	6.1:PN; 6.2:PY; 6.3:Y; 6.4:N<br>Low RoB	7.1:N; 7.2:N; 7.3:N<br>Low RoB	Moderate
Lara-Penaranda 2020	1.1:Y; 1.2:N; 1.4:PY; 1.5:Y; 1.6:N<br>Moderate RoB<br>(only women with high-grade disease have been included; age did not have an impact on the results; no adjustment for smoking)	2.1:N; 2.4:Y<br>Low RoB	3.1:Y; 3.2:Y; 3.3:N<br>Low RoB	4.1:N<br>Low RoB	5.1:Y; 5.2:PN; 5.3:Y<br>Low RoB<br>(9% were excluded due to missing data on cone length or because they were lost to f-u)	6.1:PN; 6.2:PY; 6.3:Y; 6.4:N<br>Low RoB	7.1:N; 7.2:N; 7.3:N<br>Low RoB	Moderate

Sun 2020*	1.1:Y; 1.2:N; 1.4:Y; 1.5:Y; 1.6:N<br>Low RoB<br>(all women were post-menopausal, were non-smokers, and had been treated for CIN3)	2.1:N; 2.4:Y<br>Low RoB	3.1:Y; 3.2:Y; 3.3:N<br>Low RoB	4.1:N<br>Low RoB	5.1:PY; 5.2:PN; 5.3:PN<br>Moderate RoB<br>(14% of women did not proceed with hysterectomy after conisation, thus they were excluded)	6.1:PN; 6.2:PY; 6.3:Y; 6.4:N<br>Low RoB	7.1:N; 7.2:N; 7.3:N<br>Low RoB	Moderate
Duan 2021*	1.1:Y; 1.2:Y; 1.3:N<br>Low RoB	-	-	4.1:PY; 4.2:Y; 4.3:N; 4.6:Y<br>Low RoB<br>(people randomised to CT were referred for CC if the lesion was too large; an ITT analysis was conducted)	5.1:Y<br>Low RoB<br>(9% were lost to f-u, with equal rates between groups)	6.1:N; 6.2:N; 6.3:N<br>Low RoB	7.1:NI; 7.2:N; 7.3:N<br>Some concerns	Some concerns
Zang 2021	1.1:Y; 1.2:N; 1.4:PN; 1.6:N<br>Serious RoB<br>(CIN grade did not affect risk of positive HPV test; no adjustment for age or smoking)	2.1:N; 2.4:Y<br>Low RoB	3.1:Y; 3.2:Y; 3.3:N<br>Low RoB	4.1:N<br>Low RoB	5.1:PY; 5.2:PN; 5.3:PY<br>Moderate RoB<br>(14% were excluded because they had irregular f-u, were lost to f-u, or general information or HPV DNA test results were missing)	6.1:PN; 6.2:PY; 6.3:Y; 6.4:N<br>Low RoB	7.1:N; 7.2:N; 7.3:N<br>Low RoB	Serious
Armstrong 2022	1.1:Y; 1.2:N; 1.4:PN; 1.6:N<br>Serious RoB<br>(only women with high-grade disease were included; no adjustment for age or smoking)	2.1:N; 2.4:Y<br>Low RoB	3.1:Y; 3.2:Y; 3.3:N<br>Low RoB	4.1:N<br>Low RoB	5.1:PY; 5.2:PN; 5.3:PN<br>Moderate RoB<br>(12% were excluded due to missing HPV DNA test results at 6m after treatment; rates of women lost to f-u did not differ between treatments)	6.1:PN; 6.2:PY; 6.3:Y; 6.4:N<br>Low RoB	7.1:N; 7.2:N; 7.3:N<br>Low RoB	Serious

*This study was included in the main network (risk of treatment failure).

Abbreviations used to answer signalling questions: **N**: no; **NI**: no information; **PN**: potentially no; **PY**: potentially yes; **Y**: yes

2.3.2. Preterm Birth

RoB was low in ten[136,148,149,153,159,163,173,180,188,195] NRS, moderate in 24[109,126-128,132,134,135,138,140,142,143,158,160,162,165,167,171,172,178,184,186,189,193,196] NRS and two[94,197] RCTs, and high in 56[34,78,110-125,129-131,133,137,139,141,144-147,150-152,154-157,161,164,166,168-170,174-177,179,181-183,185,187,190-192,194] NRS. Detailed RoB is presented below (Table 2.3.2.1). Of the 29 studies included in the network, RoB was low in six[136,149,159,163,180,195] NRS, moderate in seven[109,140,142,162,171,189,193] NRS and two[94,197] RCTs, and high in 14[34,78,121,123,130,141,145,147,152,157,166,179,181,190] NRS.

Table 2.3.2.1: Risk of bias in studies reporting on risk of preterm birth after CIN treatments

Study	RoB due to confounding (for RCTs this domain assessed the randomisation process) (Part 1)	RoB in selection of participants (Part 2) (only for NRS)	RoB in classification of interventions (Part 3) (only for NRS)	RoB due to deviation from intended interventions (Part 4)	RoB due to missing data (Part 5)	RoB in measurement of the outcome (Part 6)	RoB in selection of reported results (Part 7)	Overall RoB
Jones 1979	1.1:Y; 1.2:N; 1.4:PN; 1.6:N Serious RoB (no control for smoking)	2.1:N; 2.4:Y Low RoB	3.1:Y; 3.2:Y; 3.3:N Low RoB	4.1:N Low RoB	5.1:PY; 5.2:PN; 5.3:NI Low RoB (probably no or few missing data due to use of registry)	6.1:N; 6.2:NI; 6.3:Y; 6.4:N Low RoB	7.1:N; 7.2:N; 7.3:N Low RoB	Serious
Praest 1979	1.1:Y; 1.2:N; 1.4:PN; 1.6:N Serious RoB (internal comparison group)	2.1:N; 2.4:Y Low RoB	3.1:Y; 3.2:Y; 3.3:N Low RoB	4.1:N Low RoB	5.1:Y; 5.2:PN; 5.3:PN Low RoB (1% of treated women lost to f-u)	6.1:NI; 6.2:NI; 6.3:NI; 6.4:NI NI about RoB (NI about method of ascertainment of outcome)	7.1:N; 7.2:N; 7.3:N Low RoB	Serious
Leiman 1980	1.1:Y; 1.2:N; 1.4:N; 1.6:N Critical RoB (no control for confounding factors)	2.1:N; 2.4:Y Low RoB	3.1:Y; 3.2:Y; 3.3:N Low RoB	4.1:N Low RoB	5.1:NI; 5.2:NI; 5.3:PN NI about RoB	6.1:N; 6.2:NI; 6.3:Y; 6.4:N Low RoB	7.1:N; 7.2:N; 7.3:N Low RoB	Critical
Buller 1982	1.1:Y; 1.2:N; 1.4:PN; 1.6:N Serious RoB (internal comparison group)	2.1:N; 2.4:Y Low RoB	3.1:Y; 3.2:Y; 3.3:N Low RoB	4.1:N Low RoB	5.1:PY; 5.2:PN; 5.3:PN Moderate RoB (19% of treated women lost to f-u within 12m; NI about percentage lost to f-u after 12m)	6.1:NI; 6.2:NI; 6.3:NI; 6.4:NI NI about RoB (NI about method of ascertainment of outcome)	7.1:N; 7.2:N; 7.3:N Low RoB	Serious
Hemmingsson 1982	1.1:Y; 1.2:N; 1.4:PN; 1.6:N Serious RoB (internal comparison group)	2.1:N; 2.4:Y Low RoB	3.1:Y; 3.2:Y; 3.3:N Low RoB	4.1:N Low RoB	5.1:NI; 5.2:PN; 5.3:PN NI about RoB	6.1:NI; 6.2:NI; 6.3:NI; 6.4:NI NI about RoB (NI about method of ascertainment of outcome)	7.1:N; 7.2:N; 7.3:N Low RoB	Serious
Larsson 1982	1.1:Y; 1.2:N; 1.4:PN; 1.6:N Serious RoB (internal comparison group)	2.1:N; 2.4:Y Low RoB	3.1:NI; 3.2:NI; 3.3:NI NI about RoB (NI about method of ascertainment of exposure)	4.1:N Low RoB	5.1:NI; 5.2:PN; 5.3:PN NI about RoB	6.1:NI; 6.2:NI; 6.3:NI; 6.4:NI NI about RoB (NI about method of ascertainment of outcome)	7.1:N; 7.2:N; 7.3:N Low RoB	Serious
Ludviksson 1982	1.1:Y; 1.2:N; 1.4:PN; 1.6:N Serious RoB (no control for smoking)	2.1:N; 2.4:Y Low RoB	3.1:Y; 3.2:Y; 3.3:N Low RoB	4.1:N Low RoB	5.1:NI; 5.2:PN; 5.3:NI NI about RoB	6.1:NI; 6.2:NI; 6.3:NI; 6.4:NI NI about RoB (NI about method of ascertainment of outcome)	7.1:N; 7.2:N; 7.3:N Low RoB	Serious
Moinian 1982	1.1:Y; 1.2:N; 1.4:PN; 1.6:N Serious RoB (internal comparison group)	2.1:N; 2.4:Y Low RoB	3.1:Y; 3.2:Y; 3.3:N Low RoB	4.1:N Low RoB	5.1:NI; 5.2:PN; 5.3:PN NI about RoB	6.1:NI; 6.2:NI; 6.3:NI; 6.4:NI NI about RoB (NI about method of ascertainment of outcome)	7.1:N; 7.2:N; 7.3:N Low RoB	Serious
Anderson 1984	1.1:Y; 1.2:N; 1.4:PN; 1.6:N Serious RoB (no control for smoking)	2.1:N; 2.4:Y Low RoB	3.1:Y; 3.2:Y; 3.3:N Low RoB	4.1:N Low RoB	5.1:N; 5.2:PN; 5.3:NI; 5.4: NA; 5.5:N Serious RoB (25% of treated women did not respond to postal questionnaires)	6.1:PY; 6.2:PY; 6.3:N; 6.4:Y Critical RoB (postal questionnaires and private records were used for treated women; hospital records were used for untreated women)	7.1:N; 7.2:N; 7.3:N Low RoB	Critical

Kristensen 1985	1.1:Y; 1.2:N; 1.4:PN; 1.6:N<br>Serious RoB<br>(no control for smoking)	2.1:N; 2.4:Y<br>Low RoB	3.1:PY; 3.2:Y; 3.3:N<br>Low RoB	4.1:N<br>Low RoB	5.1:Y; 5.2:PN; 5.3:PN<br>Low RoB<br>(questionnaires were sent to women who had left the county, and all replied)	6.1:PN; 6.2:NI; 6.3:PY; 6.4:N<br>Low RoB<br>(ascertainment of outcome was through registries, or postal questionnaires for women who had left the county; percentage of women who had moved was not given but was probably low)	7.1:N; 7.2:N;<br>7.3:N<br>Low RoB	Serious
Kuoppala 1986	1.1:Y; 1.2:N; 1.4:PN; 1.6:N<br>Serious RoB<br>(no control for smoking)	2.1:N; 2.4:Y<br>Low RoB	3.1:Y; 3.2:Y; 3.3:N<br>Low RoB	4.1:N<br>Low RoB	5.1:NI; 5.2:PN; 5.3:NI<br>NI about RoB	6.1:NI; 6.2:NI; 6.3:NI; 6.4:NI<br>NI about RoB<br>(NI about method of ascertainment of outcome)	7.1:N; 7.2:N;<br>7.3:N<br>Low RoB	Serious
Saunders 1986	1.1:Y; 1.2:N; 1.4:PN; 1.6:N<br>Serious RoB<br>(no control for smoking)	2.1:N; 2.4:Y<br>Low RoB	3.1:PY; 3.2:NI;<br>3.3:PN<br>NI about RoB<br>(ascertainment of exposure was through hospital records and contact with GPs; not clearly described how GPs had acquired the information regarding history of cervical treatments)	4.1:N<br>Low RoB	5.1:NI; 5.2:NI; 5.3:NI<br>NI about RoB	3.1:PY; 3.2:NI; 3.3:PN<br>NI about RoB<br>(ascertainment of outcome was through hospital records and contact with GPs; not clearly described how GPs had acquired the information regarding history of cervical treatments)	7.1:N; 7.2:N;<br>7.3:N<br>Low RoB	Serious
Wakita 1990*	1.1:Y; 1.2:N; 1.4:N; 1.6:N<br>Critical RoB<br>(no control for confounding factors)	2.1:N; 2.4:Y<br>Low RoB	3.1:Y; 3.2:Y; 3.3:N<br>Low RoB	4.1:N<br>Low RoB	6.1:NI; 6.2:PN; 6.3:PN<br>NI about RoB	6.1:NI; 6.2:NI; 6.3:NI; 6.4:NI<br>NI about RoB<br>(NI about method of ascertainment of outcome)	7.1:N; 7.2:N;<br>7.3:N<br>Low RoB	Critical
Kasum 1991	1.1:Y; 1.2:N; 1.4:N; 1.6:N<br>Critical RoB<br>(no control for confounding factors)	2.1:N; 2.4:Y<br>Low RoB	3.1:Y; 3.2:Y; 3.3:N<br>Low RoB	4.1:N<br>Low RoB	5.1:N; 5.2:PN; 5.3: PN;<br>5.4:NA; 5.5:N<br>Critical RoB<br>(only 50% of treated women responded to postal questionnaires)	6.1:PY; 6.2:Y; 6.3:N; 6.4:Y<br>Critical RoB<br>(postal questionnaires and interview were used for treated women; hospital records were used for untreated women)	7.1:N; 7.2:N;<br>7.3:N<br>Low RoB	Critical
Gunasekera 1992*	1.1:Y; 1.2:N; 1.4:PY; 1.5:Y;<br>1.6:N<br>Moderate RoB<br>(matching for age, parity, smoking)	2.1:N; 2.4:Y<br>Low RoB	3.1:Y; 3.2:Y; 3.3:N<br>Low RoB	4.1:N<br>Low RoB	5.1:NI; 5.2:PN; 5.3:NI<br>NI about RoB<br>(because of prospective f-u, RoB is probably low)	6.1:N; 6.2:NI; 6.3:Y; 6.4:N<br>Low RoB	7.1:N; 7.2:N;<br>7.3:N<br>Low RoB	NI (probably moderate)
Loizzi 1992*	1.1:Y; 1.2:N; 1.4:N; 1.6:N<br>Critical RoB<br>(no control for confounding factors)	2.1:N; 2.4:Y<br>Low RoB	3.1:Y; 3.2:Y; 3.3:N<br>Low RoB	4.1:N<br>Low RoB	5.1:PY; 5.2:PN; 5.3:PN<br>Moderate RoB<br>(17% lost to f-u within 12m, but NI about how many were lost thereafter)	6.1:NI; 6.2:NI; 6.3:NI; 6.4:NI<br>NI about RoB<br>(NI about method of ascertainment of outcome)	7.1:N; 7.2:N;<br>7.3:N<br>Low RoB	Critical
Blomfield 1993	1.1:Y; 1.2:N; 1.4:PN; 1.6:N<br>Serious RoB<br>(no control for smoking)	2.1:N; 2.4:Y<br>Low RoB	3.1:Y; 3.2:Y; 3.3:N<br>Low RoB	4.1:N<br>Low RoB	5.1:NI; 5.2:PN; 5.3:NI<br>NI about RoB	6.1:N; 6.2:NI; 6.3:Y; 6.4:N<br>Low RoB	7.1:N; 7.2:N;<br>7.3:N<br>Low RoB	Serious
Haffenden 1993	1.1:Y; 1.2:N; 1.4:PN; 1.6:N<br>Serious RoB<br>(no control for smoking)	2.1:N; 2.4:Y<br>Low RoB	3.1:Y; 3.2:Y; 3.3:N<br>Low RoB	4.1:N<br>Low RoB	5.1:NI; 5.2:PN; 5.3:NI<br>NI about RoB	6.1:N; 6.2:NI; 6.3:Y; 6.4:N<br>Low RoB	7.1:N; 7.2:N;<br>7.3:N<br>Low RoB	Serious

Hagen 1993	1.1:Y; 1.2:N; 1.4:PY; 1.5:Y; 1.6:N<br>Moderate RoB<br>(matching for age, parity, smoking during 1st trimester)	2.1:N; 2.4:Y<br>Low RoB	3.1:Y; 3.2:Y; 3.3:N<br>Low RoB	4.1:N<br>Low RoB	5.1:PY; 5.2:PN; 5.3:NI<br>Low RoB<br>(2% of treated women lost to f-u, but it was not clear whether this applied to immediate post-treatment f-u or long-term f-u for reproductive outcomes)	6.1:N; 6.2:NI; 6.3:Y; 6.4:N<br>Low RoB	7.1:N; 7.2:N; 7.3:N<br>Low RoB	Moderate
Braet 1994	1.1:Y; 1.2:N; 1.4:PY; 1.5:Y; 1.6:N<br>Moderate RoB<br>(matching for age, parity, smoking)	2.1:N; 2.4:Y<br>Low RoB	3.1:Y; 3.2:Y; 3.3:N<br>Low RoB	4.1:N<br>Low RoB	5.1:NI; 5.2:PN; 5.3:NI<br>NI about RoB	6.1:NI; 6.2:NI; 6.3:NI; 6.4:NI<br>NI about RoB<br>(NI about method of ascertainment of outcome)	7.1:N; 7.2:N; 7.3:N<br>Low RoB	NI (probably moderate)
Cruickshank 1995	1.1:Y; 1.2:N; 1.4:PY; 1.5:Y; 1.6:N<br>Moderate RoB<br>(matching for age, parity, smoking)	2.1:N; 2.4:Y<br>Low RoB	3.1:Y; 3.2:Y; 3.3:N<br>Low RoB	4.1:N<br>Low RoB	5.1:PY; 5.2:PN; 5.3:NI<br>Low RoB<br>(use of registry)	6.1:N; 6.2:NI; 6.3:Y; 6.4:N<br>Low RoB	7.1:N; 7.2:N; 7.3:N<br>Low RoB	Moderate
Sagot 1995	1.1:Y; 1.2:N; 1.4:PN; 1.6:N<br>Serious RoB<br>(internal comparison group)	2.1:N; 2.4:Y<br>Low RoB	3.1:Y; 3.2:Y; 3.3:N<br>Low RoB	4.1:N<br>Low RoB	5.1:N; 5.2:PN; 5.3:PN; 5.4:NA; 5.5:N<br>Serious RoB<br>(22% of treated women could not be contacted)	6.1:NI; 6.2:NI; 6.3:NI; 6.4:NI<br>NI about RoB<br>(NI about method of ascertainment of outcome)	7.1:N; 7.2:N; 7.3:N<br>Low RoB	Serious
Spitzer 1995*	1.1:Y; 1.2:N; 1.4:PY; 1.5:PY; 1.6:N<br>Moderate RoB<br>(matching for age, parity; because an internal comparison group was used, we did not downgrade to 'Serious' for lack of direct control for smoking)	2.1:N; 2.4:Y<br>Low RoB	3.1:Y; 3.2:Y; 3.3:N<br>Low RoB	4.1:N<br>Low RoB	5.1:N; 5.2:PN; 5.3:NI; 5.4:NI; 5.5:N<br>Critical RoB<br>(only 48% responded to questionnaires)	6.1:PY; 6.2:Y; 6.3:Y; 6.4:N<br>Serious RoB<br>(ascertainment of outcome was through questionnaires)	7.1:N; 7.2:N; 7.3:N<br>Low RoB	Critical
Bekassy 1996	1.1:Y; 1.2:N; 1.4:PN; 1.6:N<br>Serious RoB<br>(no control for smoking)	2.1:N; 2.4:Y<br>Low RoB	3.1:Y; 3.2:Y; 3.3:N<br>Low RoB	4.1:N<br>Low RoB	5.1:PY; 5.2:PN; 5.3:NI<br>Low RoB<br>(use of registry)	6.1:N; 6.2:NI; 6.3:Y; 6.4:N<br>Low RoB	7.1:N; 7.2:N; 7.3:N<br>Low RoB	Serious
Raio 1997	1.1:Y; 1.2:N; 1.4:PY; 1.5:Y; 1.6:N<br>Moderate RoB<br>(matching for age, parity, smoking)	2.1:N; 2.4:Y<br>Low RoB	3.1:Y; 3.2:Y; 3.3:N<br>Low RoB	4.1:N<br>Low RoB	5.1:PY; 5.2:PN; 5.3:NI<br>Moderate RoB<br>(11% of treated women lost to f-u)	6.1:NI; 6.2:NI; 6.3:NI; 6.4:NI<br>NI about RoB<br>(NI about method of ascertainment of outcome)	7.1:N; 7.2:N; 7.3:N<br>Low RoB	Moderate
Andersen 1999	1.1:Y; 1.2:N; 1.4:PN; 1.6:N<br>Serious RoB<br>(no control for smoking)	2.1:N; 2.4:Y<br>Low RoB	3.1:Y; 3.2:Y; 3.3:N<br>Low RoB	4.1:N<br>Low RoB	5.1:NI; 5.2:PN; 5.3:NI<br>NI about RoB	6.1:NI; 6.2:NI; 6.3:NI; 6.4:NI<br>NI about RoB<br>(NI about method of ascertainment of outcome)	7.1:N; 7.2:N; 7.3:N<br>Low RoB	Serious
El-Bastawissi 1999	1.1:Y; 1.2:N; 1.4:PY; 1.5:PY; 1.6:N<br>Moderate RoB<br>(matching and regression only for external but not for untreated HSIL group; because women with untreated HSIL were used as controls, we did not downgrade to 'Serious' for lack of direct control for confounders)	2.1:N; 2.4:Y<br>Low RoB	3.1:Y; 3.2:Y; 3.3:N<br>Low RoB	4.1:N<br>Low RoB	5.1:PY; 5.2:PN; 5.3:NI<br>Moderate RoB<br>(gestational length was missing in the registry for 18% of women with CIN3 and 13% of external comparison group)	6.1:N; 6.2:NI; 6.3:Y; 6.4:N<br>Low RoB	7.1:N; 7.2:N; 7.3:N<br>Low RoB	Moderate

van Rooijen 1999	1.1:Y; 1.2:N; 1.4:PY; 1.5:Y; 1.6:N<br>Moderate RoB<br>(matching for age, parity; smoking during early pregnancy did not differ between cases and controls)	2.1:N; 2.4:Y<br>Low RoB	3.1:Y; 3.2:Y; 3.3:N<br>Low RoB	4.1:N<br>Low RoB	5.1:NI; 5.2:PN; 5.3:NI<br>NI about RoB	6.1:NI; 6.2:NI; 6.3:NI; 6.4:NI<br>NI about RoB<br>(NI about method of ascertainment of outcome)	7.1:N; 7.2:N; 7.3:N<br>Low RoB	NI (probably moderate)
Mathevet 2003*	1.1:Y; 1.2:NI; 1.3:N<br>Some concerns	-	-	4.1:PY; 4.2:Y; 4.3:PN; 4.6:PY<br>Low RoB	5.1:Y<br>Low RoB<br>(6% lost to f-u)	6.1:N; 6.2:N; 6.3:NI; 6.4:PN<br>Low RoB	7.1:NI; 7.2:N; 7.3:N<br>Some concerns	Some concerns
Sadler 2004*	1.1:Y; 1.2:N; 1.4:Y; 1.5:Y; 1.6:N<br>Low RoB<br>(colposcopy group without treatment was used as the comparator; regression for age, parity, smoking during early pregnancy)	2.1:N; 2.4:Y<br>Low RoB	3.1:Y; 3.2:Y; 3.3:N<br>Low RoB	4.1:N<br>Low RoB	5.1:PY; 5.2:N; 5.3:N<br>Low RoB<br>(only 1% of pregnancies were excluded because of missing treatment status; probably no or few missing pregnancy outcomes due to use of registry)	6.1:N; 6.2:NI; 6.3:Y; 6.4:N<br>Low RoB	7.1:N; 7.2:N; 7.3:N<br>Low RoB	Low
Tan 2004	1.1:Y; 1.2:N; 1.4:PN; 1.6:N<br>Serious RoB<br>(no control for smoking)	2.1:N; 2.4:Y<br>Low RoB	3.1:Y; 3.2:Y; 3.3:N<br>Low RoB	4.1:N<br>Low RoB	5.1:N; 5.2:PN; 5.3:NI; 5.4:NA; 5.5:N<br>Serious RoB<br>(29% of pregnant women after treatment were excluded because of missing notes)	6.1:N; 6.2:NI; 6.3:Y; 6.4:N<br>Low RoB	7.1:N; 7.2:N; 7.3:N<br>Low RoB	Serious
Samson 2005	1.1:Y; 1.2:N; 1.4:PY; 1.5:Y; 1.6:N<br>Moderate RoB<br>(matching for age, parity, smoking)	2.1:N; 2.4:Y<br>Low RoB	3.1:Y; 3.2:Y; 3.3:N<br>Low RoB	4.1:N<br>Low RoB	5.1:PY; 5.2:PN; 5.3:Y; 5.4:NA; 5.5:N<br>Moderate RoB<br>(16% of pregnancies were excluded because these women had moved out of country; <8% of pregnancies were excluded because of missing data on data such as age, parity or delivery date)	6.1:N; 6.2:NI; 6.3:Y; 6.4:N<br>Low RoB	7.1:N; 7.2:N; 7.3:N<br>Low RoB	Moderate
Crane 2006*	1.1:Y; 1.2:N; 1.4:PY; 1.5:Y; 1.6:N<br>Moderate RoB (adjustment for age, parity, smoking)	2.1:N; 2.4:Y<br>Low RoB	3.1:NI; 3.2:NI; 3.3:NI<br>NI about RoB<br>(NI about method of ascertainment of exposure)	4.1:N<br>Low RoB	5.1:NI; 5.2:PN; 5.3:NI<br>NI about RoB<br>(because of prospective f-u, the percentage of pregnancies with missing data is probably low)	6.1:N; 6.2:NI; 6.3:Y; 6.4:N<br>Low RoB	7.1:N; 7.2:Y; 7.3:N<br>Serious RoB<br>(adjusted effect estimate was not reported for the comparison of CT vs untreated women, because this comparison was statistically non-significant)	Serious
Klaritsch 2006	1.1:Y; 1.2:N; 1.4:PN; 1.6:N<br>Serious RoB<br>(smoking did not differ between groups; no control for age, parity)	2.1:N; 2.4:Y<br>Low RoB	3.1:Y; 3.2:Y; 3.3:N<br>Low RoB	4.1:N<br>Low RoB	5.1:NI; 5.2:PN; 5.3:NI<br>NI about RoB	6.1:N; 6.2:NI; 6.3:Y; 6.4:N<br>Low RoB	7.1:N; 7.2:N; 7.3:N<br>Low RoB	Serious

Bruinsma 2007*	1.1:Y; 1.2:N; 1.4:PY; 1.5:PY; 1.6:N<br>Moderate RoB<br>(colposcopy group without treatment was used as the comparator; regression for age, parity; no regression for smoking because smoking status (at time of colposcopy referral) was recorded in <10% of women, but in those for whom this information was available, rates of preterm birth did not differ between smokers and non-smokers)	2.1:N; 2.4:Y<br>Low RoB	3.1:Y; 3.2:Y; 3.3:N<br>Low RoB	4.1:N<br>Low RoB	5.1:Y; 5.2:PN; 5.3:N<br>Low RoB<br>(gestational age was missing in 1% of treated and 3% of untreated women)	6.1:N; 6.2:NI; 6.3:Y; 6.4:N<br>Low RoB	7.1:N; 7.2:N; 7.3:N<br>Low RoB	Moderate
Bull-Phelps 2007*	1.1:Y; 1.2:N; 1.4:N; 1.6:N<br>Critical RoB<br>(no control for confounding factors)	2.1:N; 2.4:Y<br>Low RoB	3.1:Y; 3.2:Y; 3.3:N<br>Low RoB	4.1:N<br>Low RoB	5.1:NI; 5.2:PN; 5.3:PN<br>NI about RoB	6.1:NI; 6.2:NI; 6.3:NI; 6.4:NI<br>NI about RoB<br>(NI about method of ascertainment of outcome)	7.1:N; 7.2:N; 7.3:N<br>Low RoB	Critical
Himes 2007*	1.1:Y; 1.2:N; 1.4:PY; 1.5:PY; 1.6:N<br>Moderate RoB<br>(smoking did not differ between cases and controls; because colposcopy group without treatment was used as the comparator, we did not downgrade to 'Serious' for lack of direct control for age or parity)	2.1:N; 2.4:Y<br>Low RoB	3.1:Y; 3.2:Y; 3.3:N<br>Low RoB	4.1:N<br>Low RoB	5.1:NI; 5.2:PN; 5.3:NI<br>NI about RoB	6.1:N; 6.2:NI; 6.3:Y; 6.4:N<br>Low RoB	7.1:N; 7.2:N; 7.3:N<br>Low RoB	NI (probably moderate)
Jakobsson 2007	1.1:Y; 1.2:N; 1.4:PY; 1.5:Y; 1.6:N<br>Moderate RoB<br>(adjustment for age, parity, smoking during pregnancy)	2.1:N; 2.4:Y<br>Low RoB	3.1:Y; 3.2:Y; 3.3:N<br>Low RoB	4.1:N<br>Low RoB	5.1:Y; 5.2:N; 5.3:N<br>Low RoB<br>(<0·1% of pregnancies were missing from the registry; in Finland only few procedures are performed in private clinics outside hospital settings which would not have been captured by the database)	6.1:N; 6.2:NI; 6.3:Y; 6.4:N<br>Low RoB	7.1:N; 7.2:N; 7.3:N<br>Low RoB	Moderate
Albrechtsen 2008	1.1:Y; 1.2:N; 1.4:PN; 1.6:N<br>Serious RoB<br>(no control for smoking)	2.1:N; 2.4:Y<br>Low RoB	3.1:Y; 3.2:Y; 3.3:N<br>Low RoB	4.1:N<br>Low RoB	5.1:Y; 5.2:PN; 5.3:N<br>Moderate RoB<br>(gestational age was missing in 5% of pregnancies; data on birth weight were almost complete; more than 80% of treatments are recorded in population-based registry, but private gynaecologists do not routinely send treatment notifications to the registry)	6.1:N; 6.2:NI; 6.3:Y; 6.4:N<br>Low RoB	7.1:N; 7.2:N; 7.3:N<br>Low RoB	Serious
Patrelli 2008*	1.1:Y; 1.2:N; 1.4:N; 1.6:N<br>Critical RoB<br>(no control for confounders)	2.1:N; 2.4:Y<br>Low RoB	3.1:Y; 3.2:Y; 3.3:N<br>Low RoB	4.1:N<br>Low RoB	5.1:NI; 5.2:PN; 5.3:PN<br>NI about RoB	6.1:N; 6.2:NI; 6.3:Y; 6.4:N<br>Low RoB	7.1:N; 7.2:N; 7.3:N<br>Low RoB	Critical

Jakobsson 2009	1.1:Y; 1.2:N; 1.4:PN; 1.6:N Serious RoB (adjustment for age, parity; no control for smoking)	2.1:N; 2.4:Y Low RoB	3.1:Y; 3.2:Y; 3.3:N Low RoB	4.1:N Low RoB	5.1:Y; 5.2:N; 5.3:N Low RoB (<0·1% of pregnancies are missing from the registry; in Finland, only few procedures are performed in private clinics outside hospital settings which would not have been captured by the database)	6.1:N; 6.2:NI; 6.3:Y; 6.4:N Low RoB	7.1:N; 7.2:N; 7.3:N Low RoB	Serious
Michelin 2009*	1.1:Y; 1.2:N; 1.4:N; 1.6:N Critical RoB (no control for confounders)	2.1:N; 2.4:Y Low RoB	3.1:Y; 3.2:Y; 3.3:N Low RoB	4.1:N Low RoB	5.1:NI; 5.2:PN; 5.3:PN NI about RoB	6.1:NI; 6.2:NI; 6.3:NI; 6.4:NI NI about RoB (NI about method of ascertainment of outcome)	7.1:N; 7.2:N; 7.3:N Low RoB	Critical
Noehr 2009 (AJOG* & Obstet Gynecol)	1.1:Y; 1.2:N; 1.4:Y; 1.5:Y; 1.6:N Low RoB (colposcopy group without treatment was used as the comparator; adjustment for age, smoking during pregnancy; parity did not differ between cases and controls)	2.1:N; 2.4:Y Low RoB	3.1:PY; 3.2:Y; 3.3:N Low RoB (authors were not able to discriminate between LC and LLETZ, but LC has become rare in Denmark)	4.1:N Low RoB	5.1:Y; 5.2:N; 5.3:N Low RoB	6.1:N; 6.2:NI; 6.3:Y; 6.4:N Low RoB	7.1:N; 7.2:N; 7.3:N Low RoB	Low
Shanbhag 2009	1.1:Y; 1.2:N; 1.4:PY; 1.5:PY; 1.6:Y Moderate RoB (authors adjusted for post-intervention factors (e.g. for birth weight in the analysis of preterm birth), thus we used unadjusted data; because women with untreated CIN3 were used as controls, we did not downgrade to 'Serious' for lack of direct control for confounders in the unadjusted data)	2.1:N; 2.4:Y Low RoB	3.1:Y; 3.2:Y; 3.3:N Low RoB	4.1:N Low RoB	5.1:PY; 5.2:Y; 5.3:N; 5.4:NI; 5.5:N Serious RoB (treatment status was missing in 53% of women with CIN3; due to use of a large population-based registry, missing data are probably completely at random, thus we did not downgrade to 'critical')	6.1:N; 6.2:NI; 6.3:Y; 6.4:N Low RoB	7.1:N; 7.2:N; 7.3:N Low RoB	Serious
Zornoza-Garcia 2009	1.1:Y; 1.2:N; 1.4:PN; 1.6:N Serious RoB (smoking did not affect risk of adverse obstetric outcomes; no control for age or parity)	2.1:N; 2.4:Y Low RoB	3.1:Y; 3.2:Y; 3.3:N Low RoB	4.1:N Low RoB	5.1:NI; 5.2:PN; 5.3:PN NI about RoB	6.1:N; 6.2:NI; 6.3:Y; 6.4:N Low RoB	7.1:N; 7.2:N; 7.3:N Low RoB	Serious

Fischer 2010	1.1:Y; 1.2:N; 1.4:PN; 1.6:N Serious RoB (matching for age; parity did not differ between groups; no control for smoking)	2.1:N; 2.4:Y Low RoB	3.1:PY; 3.2:N; 3.3:PY Serious RoB (ascertainment of exposure was through referral records for women who were referred to undergo ultrasound scan because of previous cervical treatment, or through self-reporting for women who disclosed their history of cervical treatments during ultrasound scan for other indication)	4.1:N Low RoB	5.1:PN; 5.2:PY; 5.3:NI; 5.4:NA; 5.5:N Serious RoB (women with a mid-trimester loss before ultrasound scan were missed by this study; women who did not disclose history of cervical procedures might have been missed)	6.1:N; 6.2:Y; 6.3:Y; 6.4:N Low RoB	7.1:N; 7.2:N; 7.3:N Low RoB	Serious
Nam 2010*	1.1:Y; 1.2:N; 1.4:PN; 1.6:Y Serious RoB (authors adjusted for post-intervention factors (e.g. for cone volume), thus we used unadjusted data; age and parity did not affect risk of preterm birth; no control for smoking)	2.1:N; 2.4:Y Low RoB	3.1:Y; 3.2:Y; 3.3:N Low RoB	4.1:N Low RoB	5.1:NI; 5.2:PN; 5.3:NI NI about RoB	6.1:N; 6.2:NI; 6.3:Y; 6.4:N Low RoB	7.1:N; 7.2:N; 7.3:N Low RoB	Serious
Ortoft 2010	1.1:Y; 1.2:N; 1.4:Y; 1.5:Y; 1.6:N Low RoB (women with untreated HSIL were used as controls; adjustment for age, parity, smoking)	2.1:N; 2.4:Y Low RoB	3.1:Y; 3.2:Y; 3.3:N Low RoB	4.1:N Low RoB	5.1:Y; 5.2:N; 5.3:N Low RoB (gestational age or birth weight were missing for 0·5% of controls and 0% of cases)	6.1:N; 6.2:NI; 6.3:Y; 6.4:N Low RoB	7.1:N; 7.2:N; 7.3:N Low RoB	Low
van de Vijver 2010	1.1:Y; 1.2:N; 1.4:PN; 1.6:N Serious RoB (matching for age, parity; no control for smoking)	2.1:N; 2.4:Y Low RoB	3.1:Y; 3.2:Y; 3.3:N Low RoB	4.1:N Low RoB	5.1:PN; 5.2:PN; 5.3:NI; 5.4:NA; 5.5:N Serious RoB (questionnaires were used for ascertainment of outcome; NI about how many responded, but the percentage of non-respondents might have been high)	6.1:PY; 6.2:Y; 6.3:N; 6.4:Y Critical RoB (ascertainment of outcome was through questionnaires for treated women and hospital records for untreated women)	7.1:N; 7.2:N; 7.3:N Low RoB	Critical
Werner 2010	1.1:Y; 1.2:N; 1.4:PN; 1.6:N Serious RoB (internal comparison group)	2.1:N; 2.4:Y Low RoB	3.1:Y; 3.2:Y; 3.3:N Low RoB	4.1:N Low RoB	5.1:NI; 5.2:PN; 5.3:NI NI about RoB	6.1:N; 6.2:NI; 6.3:Y; 6.4:N Low RoB	7.1:N; 7.2:N; 7.3:N Low RoB	Serious
Andia 2011	1.1:Y; 1.2:N; 1.4:PN; 1.6:N Serious RoB (no control for smoking)	2.1:N; 2.4:Y Low RoB	3.1:Y; 3.2:Y; 3.3:N Low RoB	4.1:N Low RoB	5.1:PY; 5.2:PN; 5.3:NI Low RoB (probably no or few missing date due to use of registry)	6.1:N; 6.2:NI; 6.3:Y; 6.4:N Low RoB	7.1:N; 7.2:N; 7.3:N Low RoB	Serious
Lima 2011*	1.1:Y; 1.2:N; 1.4:PN; 1.6:N Serious RoB (age and parity did not differ between cases and controls; no control for smoking)	2.1:N; 2.4:Y Low RoB	3.1:Y; 3.2:Y; 3.3:N Low RoB	4.1:N Low RoB	5.1:NI; 5.2:PN; 5.3:PN NI about RoB	6.1:N; 6.2:NI; 6.3:Y; 6.4:N Low RoB	7.1:N; 7.2:N; 7.3:N Low RoB	Serious

Castanon 2012	1.1:Y; 1.2:N; 1.4:PY; 1.5:PY; 1.6:N<br>Moderate RoB<br>(adjustment for age, parity; because colposcopy group without treatment was used as the comparator, we did not downgrade to 'Serious' for lack of direct control for smoking)	2.1:N; 2.4:Y<br>Low RoB	3.1:PY; 3.2:Y; 3.3:N<br>Low RoB<br>(some women with ablation might have been included in the untreated colposcopy group, but ablation has become rare in the UK)	4.1:N<br>Low RoB	5.1:N; 5.2:Y; 5.3:N; 5.4:PY; 5.5:N<br>Moderate RoB<br>(gestational age was missing in 30% of pregnancies; treatment status was missing in 19% of women; due to use of a large population-based registry, missing data are probably completely at random, thus we did not downgrade to 'serious')	6.1:N; 6.2:NI; 6.3:Y; 6.4:N<br>Low RoB	7.1:N; 7.2:N; 7.3:N<br>Low RoB	Moderate
Khalid 2012	1.1:Y; 1.2:N; 1.4:PY; 1.5:Y; 1.6:N<br>Moderate RoB<br>(Relationship between age/parity/smoking and risk of preterm birth was found to be non-significant in a multivariate regression analysis)	2.1:N; 2.4:Y<br>Low RoB	3.1:Y; 3.2:Y; 3.3:N<br>Low RoB	4.1:N<br>Low RoB	5.1:PY; 5.2:PY; 5.3:NI<br>Low RoB<br>(7% excluded because of incomplete data)	6.1:N; 6.2:NI; 6.3:Y; 6.4:N<br>Low RoB	7.1:N; 7.2:N; 7.3:N<br>Low RoB	Moderate
Poon 2012	1.1:Y; 1.2:N; 1.4:PN; 1.6:N<br>Serious RoB<br>(no control for age)	2.1:N; 2.4:Y<br>Low RoB	3.1:PY; 3.2:N; 3.3:PY<br>Serious RoB<br>(ascertainment of exposure was through questionnaires)	4.1:N<br>Low RoB	5.1:NI; 5.2:BNI; 5.3:NI<br>NI about RoB	6.1:N; 6.2:NI; 6.3:Y; 6.4:N<br>Low RoB	7.1:N; 7.2:N; 7.3:N<br>Low RoB	Serious
Reilly 2012*	1.1:Y; 1.2:N; 1.4:Y; 1.5:PY; 1.6:N<br>Low RoB<br>(colposcopy group without treatment was used as the comparator; adjustment for age, parity, smoking; smoking status was missing for 32% of treated and 40% of untreated women; due to use of a large population-based registry, missing smoking data were probably completely at random and did not cause bias)	2.1:N; 2.4:Y<br>Low RoB	3.1:Y; 3.2:Y; 3.3:N<br>Low RoB	4.1:N<br>Low RoB	5.1:Y; 5,2:PN; 5.3:N<br>Low RoB<br>(6% in treated and 10% in colposcopy group were lost to f-u; gestational age was unknown in 3% of pregnancies)	6.1:N; 6.2:NI; 6.3:Y; 6.4:N<br>Low RoB	7.1:N; 7.2:N; 7.3:N<br>Low RoB	Low
Simoens 2012*	1.1:Y; 1.2:N; 1.4:PY; 1.5:Y; 1.6:N<br>Moderate RoB (regression for age, parity, smoking)	2.1:N; 2.4:Y<br>Low RoB	3.1:PY; 3.2:PY; 3.3:PN<br>Moderate RoB<br>(ascertainment of exposure was through questionnaires along with confirmation from medical records; because of double-checking, RoB is probably not serious)	4.1:N<br>Low RoB	5.1:NI; 5.2:NI; 5.3:NI<br>NI about RoB<br>(because of prospective f-u, the percentage of pregnancies with missing data is probably low)	6.1:N; 6.2:NI; 6.3:Y; 6.4:N<br>Low RoB	7.1:N; 7.2:N; 7.3:N<br>Low RoB	NI (probably moderate)
Van Hentenryck 2012	1.1:Y; 1.2:N; 1.4:PY; 1.5: Y; 1.6:N<br>Moderate RoB<br>(matching for age, parity, smoking)	2.1:N; 2.4:Y<br>Low RoB	3.1:Y; 3.2:Y; 3.3:N<br>Low RoB	4.1:N<br>Low RoB	5.1:NI; 5.2:PN; 5.3:NI<br>NI about RoB	6.1:N; 6.2:NI; 6.3:Y; 6.4:N<br>Low RoB	7.1:N; 7.2:N; 7.3:N<br>Low RoB	NI (probably moderate)

Berretta 2013	1.1:Y; 1.2:N; 1.4:PN; 1.6:N<br>Serious RoB<br>(age and parity did not affect risk of preterm birth; no control for smoking)	2.1:N; 2.4:Y<br>Low RoB	3.1:Y; 3.2:Y; 3.3:N<br>Low RoB	4.1:N<br>Low RoB	5.1:N; 5.2:PN; 5.3:PN; 5.4:NI; 5.5:N<br>Serious RoB<br>(35% refused to answer the questionnaire)	6.1:PY; 6.2:Y; 6.3:Y; 6.4:N<br>Serious RoB<br>(ascertainment of outcome was through questionnaires)	7.1:N; 7.2:N; 7.3:N<br>Low RoB	Serious
Frega 2013	1.1:Y; 1.2:N; 1.4:PN; 1.6:N<br>Serious RoB<br>(no control for age)	2.1:N; 2.4:Y<br>Low RoB	3.1:Y; 3.2:Y; 3.3:N<br>Low RoB	4.1:N<br>Low RoB	5.1:Y; 5.2:PN; 5.3:PN<br>Low RoB<br>(9% of treated women refused to participate; 4% of treated and 5% of untreated pregnant women were lost to f-u)	6.1:N; 6.2:NI; 6.3:Y; 6.4:N<br>Low RoB	7.1:N; 7.2:N; 7.3:N<br>Low RoB	Serious
Frey 2013*	1.1:Y; 1.2:N; 1.4:PY; 1.5:PY; 1.6:N<br>Moderate RoB<br>(matching for age; parity did not differ between cases and controls; because colposcopy group without treatment was used as the comparator, we did not downgrade to 'Serious' for lack of direct control for smoking)	2.1:N; 2.4:Y<br>Low RoB	3.1:Y; 3.2:Y; 3.3:N<br>Low RoB	4.1:N<br>Low RoB	5.1:NI; 5.2:PN; 5.3:NI<br>NI about RoB<br>(NI about how many women agreed to participate in the structured phone interview regarding pregnancy outcomes; responses of the participating women were confirmed from medical records with 'minimal' missing data)	6.1:N; 6.2:NI; 6.3:Y; 6.4:N<br>Low RoB	7.1:N; 7.2:N; 7.3:N<br>Low RoB	NI (probably moderate)
Guo 2013*	1.1:Y; 1.2:N; 1.4:Y; 1.5:Y; 1.6:N<br>Low RoB<br>(only non-smokers were included; age and parity did not differ between cases and controls; colposcopy group without treatment the comparator)	2.1:N; 2.4:Y<br>Low RoB	3.1:Y; 3.2:Y; 3.3:N<br>Low RoB	4.1:N<br>Low RoB	5.1:NI; 5.2:PN; 5.3:NI<br>NI about RoB<br>(because of prospective f-u, RoB is probably low)	6.1:N; 6.2:NI; 6.3:Y; 6.4:N<br>Low RoB	7.1:N; 7.2:N; 7.3:N<br>Low RoB	NI (probably low)
Castanon 2014	1.1:Y; 1.2:N; 1.4:PY; 1.5:PY; 1.6:N<br>Moderate RoB<br>(adjustment for age, parity; because colposcopy group without treatment was used as the comparator, we did not downgrade to 'Serious' for lack of direct control for smoking)	2.1:N; 2.4:Y<br>Low RoB	3.1:PY; 3.2:Y; 3.3:N<br>Low RoB<br>(some women with ablation might have been included in the untreated colposcopy group, but ablation has become rare in the UK)	4.1:N<br>Low RoB	5.1:PY; 5.2:PY; 5.3:Y<br>Moderate RoB<br>(gestational age was missing for 17% of pregnancies in the registry; code length was missing for 13% of treated women)	6.1:N; 6.2:NI; 6.3:Y; 6.4:N<br>Low RoB	7.1:N; 7.2:N; 7.3:N<br>Low RoB	Moderate
Kitson 2014*	1.1:Y; 1.2:N; 1.3:Y; 1.5:Y; 1.6:N<br>Low RoB<br>(colposcopy group without treatment was used as the comparator; matching for age, parity, smoking)	2.1:N; 2.4:Y<br>Low RoB	3.1:Y; 3.2:Y; 3.3:N<br>Low RoB	4.1:N<br>Low RoB	5.1:NI; 5.2:PN; 5.3:NI<br>NI about RoB	6.1:N; 6.2:NI; 6.3:Y; 6.4:N<br>Low RoB	7.1:N; 7.2:N; 7.3:N<br>Low RoB	NI (probably low)

Liu 2014*	1.1:NI; 1.2:NI; 1.3:N<br>Some concerns	-	-	4.1:PY; 4.2:Y; 4.3:N; 4.6:PN<br>Some concerns (authors recruited women with expectancy of spontaneous pregnancy within 3y; all recruited patients either became pregnant or sterile and none of them seem not to have conceived out of choice; a post-randomisation exclusion of eligible trial participants seems likely)	5.1:Y<br>Low RoB<br>(4% in CKC and 5% in LLETZ group lost to f-u)	6.1:N; 6.2:N; 6.3:NI; 6.4:PN<br>Low RoB	7.1:NI; 7.2:N; 7.3:N<br>Some concerns	Some concerns
Sozen 2014	1.1:Y; 1.2:N; 1.4:PN; 1.6:N<br>Serious RoB<br>(no control for smoking)	2.1:N; 2.4:Y<br>Low RoB	3.1:Y; 3.2:Y; 3.3:N<br>Low RoB	4.1:N<br>Low RoB	5.1:NI; 5.2:PN; 5.3:NI<br>NI about RoB	6.1:N; 6.2:NI; 6.3:Y; 6.4:N<br>Low RoB	7.1:N; 7.2:N; 7.3:N<br>Low RoB	Serious
Cai 2015*	1.1:Y; 1.2:N; 1.4:PN; 1.6:N<br>Serious RoB<br>(no control for age or smoking)	2.1:N; 2.4:Y<br>Low RoB	3.1:Y; 3.2:Y; 3.3:N<br>Low RoB	4.1:N<br>Low RoB	6.1:NI; 6.2:PN; 6.3:PN<br>NI about RoB	6.1:NI; 6.2:NI; 6.3:NI; 6.4:NI<br>NI about RoB<br>(NI about method of ascertainment of outcome)	7.1:N; 7.2:N; 7.3:N<br>Low RoB	Serious
Kirn 2015	1.1:Y; 1.2:N; 1.3:PY; 1.5:Y; 1.6:N<br>Moderate RoB (matching for age, parity, smoking)	2.1:N; 2.4:Y<br>Low RoB	3.1:NI; 3.2:NI; 3.3:NI<br>NI about RoB (NI about method of ascertainment of exposure)	4.1:N<br>Low RoB	5.1:NI; 5.2:PN; 5.3:Y; 5.4;NA; 5.5:N<br>NI about RoB<br>(4% of treated women were excluded because no suitable matched partner could be found; NI about how many women were excluded because of missing treatment status or missing pregnancy outcomes)	6.1:N; 6.2:NI; 6.3:Y; 6.4:N<br>Low RoB	7.1:N; 7.2:N; 7.3:N<br>Low RoB	NI (probably moderate)
Martyn 2015*	1.1:Y; 1.2:N; 1.4:PY; 1.5:PY; 1.6:N<br>Moderate RoB<br>(matching for age; because colposcopy group without treatment was used as the comparator, we did not downgrade to 'serious' for lack of direct control for parity and smoking)	2.1:N; 2.4:Y<br>Low RoB	3.1:NI; 3.2:NI; 3.3:NI<br>NI about RoB (NI about method of ascertainment of exposure)	4.1:N<br>Low RoB	5.1:N; 5.2:PN; 5.3:NI; 5.4:N; 5.5:N<br>Critical RoB<br>(46% of treated women and 30% of controls responded to questionnaires)	6.1:PY; 6.2:Y; 6.3:Y; 6.4:N<br>Serious RoB<br>(ascertainment of outcome was through questionnaires)	7.1:N; 7.2:N; 7.3:N<br>Low RoB	Critical
Miller 2015	1.1:Y; 1.2:N; 1.4:PY; 1.5:PY; 1.6:N<br>Moderate RoB<br>(regression for age; smoking during pregnancy did not differ between cases and controls; because untreated women with history of CIN were used as controls, we did not downgrade to 'serious' for lack of direct control for parity)	2.1:N; 2.4:Y<br>Low RoB	3.1:PY; 3.2:PY; 3.3:PN<br>NI about RoB<br>(ascertainment of exposure was through patient's prenatal records; unclear how history of cervical procedures had been confirmed at the time of obstetric admission history)	4.1:N<br>Low RoB	5.1:NI; 5.2:PN; 5.3:N<br>NI about RoB<br>(if a variable was missing from a patient, this individual variable was removed from analysis; NI about how many patients had missing treatment status or missing pregnancy outcomes)	6.1:N; 6.2:NI; 6.3:Y; 6.4:N<br>Low RoB	7.1:N; 7.2:N; 7.3:N<br>Low RoB	NI (probably moderate)

Aleman 2016	1.1:Y; 1.2:N; 1.4:PN; 1.6:N<br>Serious RoB<br>(no control for age and parity; adjustment for ‘chronic and gynaecological disorders’, without specifying which these disorders were and/or why they were considered confounding factors)	2.1:N; 2.4:Y<br>Low RoB	3.1:PY; 3.2:N; 3.3:PY<br>Serious RoB<br>(ascertainment of exposure was through questionnaires)	4.1:N<br>Low RoB	5.1:NI; 5.2:PN; 5.3:NI<br>NI about RoB	6.1:N; 6.2:NI; 6.3:Y; 6.4:N<br>Low RoB	7.1:N; 7.2:N; 7.3:N<br>Low RoB	Serious
Anwar 2016	1.1:Y; 1.2:N; 1.4:PN; 1.6:N<br>Serious RoB<br>(internal comparison group)	2.1:N; 2.4:Y<br>Low RoB	3.1:Y; 3.2:Y; 3.3:N<br>Low RoB	4.1:N<br>Low RoB	5.1:NI; 5.2:PN; 5.3:PN<br>NI about RoB	6.1:N; 6.2:NI; 6.3:Y; 6.4:N<br>Low RoB	7.1:N; 7.2:N; 7.3:N<br>Low RoB	Serious
Bjorge 2016*	1.1:Y; 1.2:N; 1.4:PY; 1.5:Y; 1.6:N<br>Moderate RoB<br>(adjustment for age, parity, smoking)	2.1:N; 2.4:Y<br>Low RoB	3.1:Y; 3.2:Y; 3.3:N<br>Low RoB	4.1:N<br>Low RoB	5.1:Y; 5.2:PN; 5.3:N<br>Moderate RoB<br>(1·2% of pregnancies in treated group and 1·5% in untreated group had missing data about gestational duration; more than 80% of treatments are recorded in population-based registry, but private gynaecologists do not routinely send treatment notifications to the registry)	6.1:N; 6.2:NI; 6.3:Y; 6.4:N<br>Low RoB	7.1:N; 7.2:N; 7.3:N<br>Low RoB	Moderate
Brie 2016	1.1:Y; 1.2:N; 1.4:PN; 1.6:N<br>Serious RoB<br>(parity did not differ between groups; no control for age or smoking)	2.1:N; 2.4:Y<br>Low RoB	3.1:Y; 3.2:Y; 3.3:N<br>Low RoB	4.1:N<br>Low RoB	5.1:NI; 5.2:PN; 5.3:PN<br>NI about RoB	6.1:N; 6.2:NI; 6.3:Y; 6.4:N<br>Low RoB	7.1:N; 7.2:N; 7.3:N<br>Low RoB	Serious
Jancar 2016*	1.1:Y; 1.2:N; 1.4:PY; 1.5:Y; 1.6:N<br>Moderate RoB<br>(adjustment for age, parity, smoking)	2.1:N; 2.4:Y<br>Low RoB	3.1:PY; 3.2:PY; 3.3:PN<br>NI about RoB<br>(ascertainment of exposure was through patient’s medical history records; unclear how history of cervical procedures had been confirmed at the time of history taking; patients with both CKC and LLETZ were classified into CKC group, but only 1% had received both procedures)	4.1:N<br>Low RoB	5.1:Y; 5.2:NI; 5.3:N<br>Low RoB<br>(99·9% of deliveries took place in a hospital and were recorded in the population-based registry)	6.1:N; 6.2:NI; 6.3:Y; 6.4:N<br>Low RoB	7.1:N; 7.2:N; 7.3:N<br>Low RoB	Moderate
Liverani 2016	1.1:Y; 1.2:N; 1.4:PN; 1.6:N<br>Serious RoB<br>(relationship between smoking and preterm birth was found to be non-significant in a multivariate regression analysis; mean cone length did not differ amongst age groups; no control for smoking)	2.1:N; 2.4:Y<br>Low RoB	3.1:Y; 3.2:Y; 3.3:N<br>Low RoB	4.1:N<br>Low RoB	5.1:NI; 5.2:NI; 5.3:NI<br>NI about RoB	6.1:N; 6.2:NI; 6.3:Y; 6.4:N<br>Low RoB	7.1:N; 7.2:N; 7.3:N<br>Low RoB	Serious

Mariya 2016*	1.1:Y; 1.2:N; 1.4:PN; 1.6:N Serious RoB (age and parity did not differ between groups; no control for smoking)	2.1:N; 2.4:Y Low RoB	3.1:Y; 3.2:Y; 3.3:N Low RoB	4.1:N Low RoB	5.1:PY; 5.2:PN; 5.3:PN Moderate RoB (4% lost to f-u within 5m, but NI about how many were lost thereafter)	6.1:N; 6.2:NI; 6.3:Y; 6.4:N Low RoB	7.1:N; 7.2:N; 7.3:N Low RoB	Serious
Chevreau 2017	1.1:Y; 1.2:N; 1.4:PN; 1.6:N Serious RoB (mean cone length or risk of preterm birth did not differ between age groups; smoking during pregnancy differed between age groups but there was no adjustment for age; no control for parity)	2.1:N; 2.4:Y Low RoB	3.1:Y; 3.2:Y; 3.3:N Low RoB	4.1:N Low RoB	5.1:PY; 5,2:PY; 5.3:PN Low RoB (5% lost to f-u; cone length was missing for 8% of pregnancies)	6.1:N; 6.2:NI; 6.3:Y; 6.4:N Low RoB	7.1:N; 7.2:N; 7.3:N Low RoB	Serious
van Velthoven 2017	1.1:Y; 1.2:N; 1.4:PY; 1.5:Y; 1.6:N Moderate RoB (matching for age, parity; smoking during pregnancy did not differ between groups)	2.1:N; 2.4:Y Low RoB	3.1:Y; 3.2:Y; 3.3:N Low RoB	4.1:N Low RoB	5.1:NI; 5.2:PN; 5.3:NI NI about RoB	6.1:NI; 6.2:NI; 6.3:NI; 6.4:NI NI about RoB (NI about method of ascertainment of outcome)	7.1:N; 7.2:N; 7.3:N Low RoB	NI (probably moderate)
Weinmann 2017	1.1:Y; 1.2:N; 1.4:Y; 1.5:Y; 1.6:N Low RoB (matching for age; adjustment for age, parity, smoking (ever); colposcopy group without treatment was used as the comparator)	2.1:N; 2.4:Y Low RoB	3.1:Y; 3.2:Y; 3.3:N Low RoB	4.1:N Low RoB	5.1:PY; 5.2:PN; 5.3:Y; 5.4:Y; 5.5:N Low RoB (probably no or few missing date due to use of registry; 9% of cases and 7% of controls were not included in regression analysis because of missing data on confounders)	6.1:N; 6.2:NI; 6.3:Y; 6.4:N Low RoB	7.1:N; 7.2:N; 7.3:N Low RoB	Low
Zebitay 2017	1.1:Y; 1.2:N; 1.4:PN; 1.6:N Serious RoB (no control for age or parity)	2.1:N; 2.4:Y Low RoB	3.1:Y; 3.2:Y; 3.3:N Low RoB	4.1:N Low RoB	5.1:NI; 5.2:PN; 5.3:NI NI about RoB	6.1:N; 6.2:NI; 6.3:Y; 6.4:N Low RoB	7.1:N; 7.2:N; 7.3:N Low RoB	Serious
Heinonen 2018*	1.1:Y; 1.2:N; 1.4:Y: 1.5:PY; 1.6:N Low RoB (for the comparison of LLETZ to untreated women with CIN1: regression for age and smoking which in Finland is strongly related to smoking; no regression for parity but parity did not differ between groups and additionally, subgroup analyses according to parity did not significantly change the results)	2.1:N; 2.4:Y Low RoB	3.1:Y; 3.2:Y; 3.3:N Low RoB	4.1:N Low RoB	5.1:Y; 5.2:N; 5.3:N Low RoB (<0·1% of deliveries had missing data)	6.1:N; 6.2:NI; 6.3:Y; 6.4:N Low RoB	7.1:N; 7.2:N; 7.3:N Low RoB	Low

Papoutsis 2018*	1.1:Y; 1.2:N; 1.4:PN; 1.6:N Serious RoB (although authors adjusted for age, parity and smoking, they also adjusted for highly correlated variables (e.g. for both pre-treatment cytology and colposcopy); due to existence of collinearity and potential inflation of variance, we downgraded to 'Serious')	2.1:N; 2.4:Y Low RoB	3.1:Y; 3.2:Y; 3.3:N Low RoB	4.1:N Low RoB	5.1:NI; 5.2:PN; 5.3:NI NI about RoB	6.1:N; 6.2:NI; 6.3:Y; 6.4:N Low RoB	7.1:N; 7.2:N; 7.3:N Low RoB	Serious
Wittmaack 2019	1.1:Y; 1.2:N; 1.4:N; 1.6:Y Critical RoB (authors adjusted for pPROM, which is a post-intervention outcome affected by treatment, thus we used unadjusted data)	2.1:N; 2.4:Y Low RoB	3.1:Y; 3.2:Y; 3.3:N Low RoB	4.1:N Low RoB	5.1:NI; 5.2:PN; 5.3:NI NI about RoB (women with incomplete data were excluded, but authors do not report how many these women were)	6.1:PN; 6.2:PY; 6.3:Y; 6.4:N Low RoB	7.1:N; 7.2:N; 7.3:N Low RoB	Critical
Zhang 2020*	1.1:Y; 1.2:N; 1.4:N; 1.6:N Critical RoB (no adjustment for age, parity or smoking)	2.1:N; 2.4:Y Low RoB	3.1:Y; 3.2:Y; 3.3:N Low RoB	4.1:N Low RoB	5.1:NI; 5.2:PN; 5.3:PN NI about RoB	6.1:NI; 6.2:NI; 6.3:NI; 6.4:NI NI about RoB (NI about method of ascertainment of outcome)	7.1:N; 7.2:N; 7.3:N Low RoB	Critical
Collins 2021	1.1:Y; 1.2:N; 1.4:PN; 1.6:N Serious RoB (age did not have an impact on the risk of preterm birth; no adjustment for parity or smoking in the analysis of cone length)	2.1:N; 2.4:Y Low RoB	3.1:Y; 3.2:Y; 3.3:N Low RoB	4.1:N Low RoB	5.1:N; 5.2:PN; 5.3:Y Serious RoB (cone length was missing for 22%)	6.1:N; 6.2:NI; 6.3:Y; 6.4:N Low RoB	7.1:N; 7.2:N; 7.3:N Low RoB	Serious
Loopik 2021	1.1:Y; 1.2:N; 1.4:PY; 1.5:Y; 1.6:N Moderate RoB (authors adjusted for age and parity, and they also used women with untreated CIN as a comparison group; no adjustment for smoking)	2.1:N; 2.4:Y Low RoB	3.1:Y; 3.2:Y; 3.3:N Low RoB	4.1:N Low RoB	5.1:PY; 5.2:PN; 5.3:PN Low RoB	6.1:PN; 6.2:PY; 6.3:Y; 6.4:N Low RoB	7.1:N; 7.2:N; 7.3:N Low RoB	Moderate RoB
Nitahara 2021	1.1:Y; 1.2:N; 1.4:PY; 1.5:Y; 1.6:N Serious RoB (adjustment for age, smoking during pregnancy; no control for parity)	2.1:N; 2.4:Y Low RoB	3.1:Y; 3.2:Y; 3.3:N Low RoB	4.1:N Low RoB	5.1:Y; 5.2:PN; 5.3:PN Low RoB	6.1:PN; 6.2:PY; 6.3:Y; 6.4:N Low RoB	7.1:N; 7.2:N; 7.3:N Low RoB	Serious RoB
Mosseri 2022	1.1:Y; 1.2:N; 1.4:PN; 1.6:N Critical RoB (no adjustment for age, parity or smoking)	2.1:N; 2.4:Y Low RoB	3.1:Y; 3.2:Y; 3.3:N Low RoB	4.1:N Low RoB	5.1:N; 5.2:PN; 5.3:PN Moderate RoB (14% had no data on duration of pregnancy and were excluded; 3% had no data on cone length)	6.1:PN; 6.2:PY; 6.3:Y; 6.4:N Low RoB	7.1:N; 7.2:N; 7.3:N Low RoB	Critical
Panelli 2022	1.1:Y; 1.2:N; 1.4:PY: 1.5:Y; 1.6:N Moderate RoB (adjustment for age, parity, smoking)	2.1:N; 2.4:Y Low RoB	3.1:Y; 3.2:Y; 3.3:N Low RoB	4.1:N Low RoB	5.1:NI; 5.2:PN; 5.3:NI NI about RoB	6.1:PN; 6.2:PY; 6.3:Y; 6.4:N Low RoB	7.1:N; 7.2:N; 7.3:N Low RoB	NI (probably moderate)

Wiik 2022	1.1:Y; 1.2:N; 1.4:PY; 1.5:Y; 1.6:N<br>Low RoB<br>(adjustment for age, parity, smoking; authors used women with untreated CIN as controls)	2.1:N; 2.4:Y<br>Low RoB	3.1:Y; 3.2:Y; 3.3:N<br>Low RoB	4.1:N<br>Low RoB	5.1:PY; 5.2:PN; 5.3:PN<br>Low RoB	6.1:PN; 6.2:PY; 6.3:Y; 6.4:N<br>Low RoB	7.1:N; 7.2:N; 7.3:N<br>Low RoB	Low RoB

*This study was included in the main network (risk of preterm birth).

Abbreviations used to answer signalling questions: **N**: no; **NI**: no information; **PN**: potentially no; **PY**: potentially yes; **Y**: yes

2.4. Standard Meta-Analyses

In the following tables, the treatment listed before '–' indicates the first comparator, while the treatment listed after '–' indicates the second comparator of the pairwise meta-analysis, meaning that ORs <1 favour the first treatment (i.e. the first treatment has a lower risk of treatment failure or preterm birth) while ORs >1 favour the second treatment.

2.4.1. Treatment Failure

Table 2.4.1.1: Pairwise meta-analyses for risk of CIN treatment failure

Comparison	N studies	n1/N1 (%)	n2/N2 (%)	N patients (total)	OR (95% CI)	p-value (for OR)	τ^2 (95% CI)	I^2 (95% CI)
CKC–LC	6	68/398 (17·1%)	38/361 (10·5%)	759	1·37 (1·11–1·69)	0·012	0·00 (0·00–0·00)	0% (0–75)
CKC–RD	2	44/684 (6·4%)	40/222 (18·0%)	906	0·34 (0·21–0·54)	<0·0001	0·00 (NA)	0% (NA)
CKC–LA	2	4/47 (8·5%)	8/53 (15·1%)	100	0·49 (0·11–2·08)	0·33	0·00 (NA)	0% (NA)
CKC–CC	1	8/92 (8·7%)	7/65 (10·8%)	157	0·79 (0·27–2·30)	0·66	–	–
CKC–CT	2	24/653 (3·7%)	21/139 (15·1%)	792	0·24 (0·12–0·46)	<0·0001	0·00 (NA)	0% (NA)
CKC–LLETZ	27	153/2236 (6·8%)	355/2173 (16·3%)	4409	0·64 (0·50–0·80)	0·0005	0·00 (0·00–0·44)	0% (0–43)
LC–LA	5	22/412 (5·3%)	51/535 (9·5%)	947	0·55 (0·27–1·16)	0·090	0·00 (0·00–5·95)	0% (0–79)
LC–LLETZ	11	98/1740 (5·6%)	244/2217 (11·0%)	3957	0·56 (0·31–1·02)	0·057	0·49 (0·06–1·91)	63% (29–81)
RD–LA	1	2/28 (7·1%)	1/33 (3·0%)	61	2·46 (0·21–28·69)	0·47	–	–
RD–CC	1	16/69 (23·2%)	7/65 (10·8%)	134	2·50 (0·95–6·55)	0·062	–	–
RD–CT	1	3/27 (11·1%)	9/30 (30·0%)	57	0·29 (0·07–1·22)	0·092	–	–
LA–CC	1	15/67 (22·4%)	12/65 (18·5%)	132	1·27 (0·54–2·98)	0·58	–	–
LA–CT	13	280/2408 (11·6%)	278/2465 (11·3%)	4873	1·01 (0·69–1·49)	0·94	0·17 (0·01–1·05)	50% (6–74)
LA–LLETZ	8	196/817 (24·0%)	142/1023 (13·9%)	1840	1·77 (1·22–2·57)	0·0083	0·10 (0·00–0·54)	34% (0–71)

CC–CT	2	16/159<br>(10·1%	20/141<br>(14·2%)	300	0·59<br>(0·28–1·22)	0·15	0·00<br>(NA)	0<br>(NA)
CC–LLETZ	2	42/378<br>(11·1%)	39/402<br>(9·7%)	780	1·02<br>(0·61–1·70)	0·93	1·25<br>(NA)	82%<br>(26–96)
CT–LLETZ	2	87/330<br>(26·4%)	47/330<br>(14·2%)	660	2·15<br>(1·45–3·19)	0·0001	0·04<br>(NA)	31%<br>(NA)

2.4.2. Preterm Birth

Table 2.4.2.1: Pairwise meta-analyses for risk of preterm birth after CIN treatments

Comparison	N studies	n1/N1 (%)	n2/N2 (%)	N patients (total)	OR (95% CI)	p-value (for OR)	τ^2 (95% CI)	I^2 (95% CI)
CKC–LC	3	NA/102 (NA)	NA/3607 (NA)	3709	1·15 (0·79–1·68)	0·26	0·00 (0·00–2·89)	0% (0–90)
CKC–LLETZ	13	NA/2569 (NA)	NA/7372 (NA)	9941	1·64 (1·35–2·00)	0·0001	0·00 (0·00–1·11)	5% (0–59)
CKC–RD	1	11/71 (15·5%)	109/760 (14·3%)	831	0·91 (0·45–1·83)	0·79	–	–
CKC–LA	2	NA/132 (NA)	NA/1101 (NA)	1233	1·64 (0·88–3·05)	0·12	0·00 (NA)	0% (NA)
CKC–CT	3	NA/111 (NA)	NA/67 (NA)	178	2·16 (0·18–25·33)	0·31	0·00 (0·00–48·34)	0% (0–90)
CKC–COLPO	2	25/107 (23·4%)	323/3552 (9·1%)	3659	1·95 (1·14–3·34)	0·015	0·00 (NA)	0% (NA)
LC–LLETZ	6	NA/3747 (NA)	NA/4763 (NA)	8510	1·24 (0·76–2·04)	0·31	0·08 (0·00–2·48)	6% (0–76)
LC–LA	5	NA/3744 (NA)	NA/472 (NA)	4216	1·46 (0·77–2·77)	0·18	0·00 (0·00–3·59)	0% (0–79)
LC–CT	1	NA/3587 (NA)	NA/24 (NA)	3611	1·40 (0·35–5·54)	0·63	–	–
LC–COLPO	1	20/105 (19·0%)	52/426 (12·2%)	531	1·36 (0·74–2·50)	0·33	–	–
LLETZ–RD	1	11/69 (15·9%)	109/760 (14·3%)	829	0·95 (0·47–1·93)	0·88	–	–
LLETZ–LA	4	NA/4730 (NA)	NA/1433 (NA)	6163	1·49 (1·01–2·19)	0·047	0·00 (0·00–1·53)	0% (0–85)
LLETZ–CC	1	4/60 (6·7%)	5/56 (8·9%)	116	2·04 (0·05–87·13)	0·71	–	–
LLETZ–CT	2	NA/4436 (NA)	NA/60 (NA)	4496	1·51 (0·48–4·77)	0·48	0·84 (NA)	51% (0–87)
LLETZ–COLPO	10	961/12186 (7·9%)	2211/40358 (5·5%)	52544	1·41 (1·17–1·71)	0·0027	0·03 (0·00–0·26)	48% (0–75)

RD–LA	1	109/760<br>(14·3%)	92/1005<br>(9·2%)	1765	1·77<br>(1·30–2·42)	0·0003	–	–
RD–COLPO	1	109/760<br>(14·3%)	309/3484<br>(8·9%)	4244	1·88<br>(1·47–2·41)	<0·0001	–	–
LA–CT	1	NA/96<br>(NA)	NA/24<br>(NA)	120	0·94<br>(0·17–5·25)	0·94	–	–
LA–COLPO	2	115/1228<br>(9·4%)	361/3910<br>(9·2%)	5138	0·99<br>(0·79–1·24)	0·95	0·01<br>(NA)	18%<br>(NA)
Treatment–COLPO	17	2936/33679<br>(8·7%)	3159/55314<br>(5·7%)	88993	1·31<br>(1·18–1·46)	0·0001	0·01<br>(0·00–0·15)	46%<br>(4–69)
Treatment–External	53	6976/65114<br>(10·7%)	275483/5065632<br>(5·4%)	5130746	1·93<br>(1·70–2·20)	<0·0001	0·09<br>(0·05–0·27)	89%<br>(86–91)
Treatment–Internal	18	2915/20531<br>(14·2%)	3844/60891<br>(6·3%)	81422	1·9<br>(1·39–2·58)	0·0004	0·17<br>(0·03–0·76)	75%<br>(61–84)
COLPO–External	10	2658/48975<br>(5·4%)	66563/1270288<br>(5·2%)	1319263	1·29<br>(1·16–1·43)	0·0004	0·01<br>(0·00–0·11)	51%<br>(0–76)
Internal–External	10	3786/59442<br>(6·4%)	206894/3534832<br>(5·9%)	3594274	1·09<br>(1·04–1·13)	0·0011	0·00<br>(0·00–0·30)	6%<br>(0–65)
Internal–COLPO	3	167/2913<br>(5·7%)	527/7373<br>(7·1%)	10286	0·92<br>(0·75–1·12)	0·21	0·00<br>(0·00–1·06)	0%<br>(0–90)

2.5. Network Meta-Analyses

In the following league tables, each box represents the comparison of the row-defining treatment vs the column-defining treatment. OR is reported first, followed by 95% CI and 95% PI. ORs>1 favour the column-defining treatment, while ORs<1 favour the row-defining treatment. At the end of the section we present a summary figure for both outcomes.

2.5.1. Treatment Failure

Table 2.5.1.1: Network meta-analysis for risk of CIN treatment failure (N=71 studies)

**CKC<br>(P-score: 0·89)**	1·07<br>(0·76–1·50)<br>(0·53–2·17)	0·36<br>(0·20–0·64)<br>(0·15–0·84)	0·38<br>(0·27–0·53)<br>(0·18–0·76)	0·58<br>(0·35–0·96)<br>(0·26–1·30)	0·34<br>(0·24–0·50)<br>(0·17–0·71)	0·63<br>(0·50–0·81)<br>(0·33–1·23)
0·93<br>(0·67–1·31)<br>(0·46–1·89)	**LC<br>(P-score: 0·94)**	0·34<br>(0·18–0·64)<br>(0·14–0·82)	0·35<br>(0·25–0·50)<br>(0·17–0·72)	0·54<br>(0·32–0·93)<br>(0·24–1·24)	0·32<br>(0·21–0·48)<br>(0·15–0·67)	0·59<br>(0·44–0·79)<br>(0·30–1·17)
2·79<br>(1·57–4·94)<br>(1·19–6·51)	2·98<br>(1·57–5·67)<br>(1·21–7·33)	**RD<br>(P-score: 0·19)**	1·04<br>(0·56–1·95)<br>(0·43–2·53)	1·62<br>(0·82–3·22)<br>(0·64–4·12)	0·96<br>(0·51–1·80)<br>(0·39–2·33)	1·76<br>(0·97–3·20)<br>(0·74–4·19)
2·67<br>(1·89–3·75)<br>(1·31–5·42)	2·86<br>(2·00–4·08)<br>(1·39–5·85)	0·96<br>(0·51–1·78)<br>(0·40–2·32)	**LA<br>(P-score: 0·22)**	1·55<br>(0·96–2·52)<br>(0·71–3·43)	0·92<br>(0·71–1·19)<br>(0·47–1·80)	1·69<br>(1·27–2·24)<br>(0·85–3·34)
1·72<br>(1·04–2·83)<br>(0·77–3·82)	1·84<br>(1·08–3·13)<br>(0·81–4·19)	0·62<br>(0·31–1·22)<br>(0·24–1·56)	0·64<br>(0·40–1·04)<br>(0·29–1·42)	**CC<br>(P-score: 0·54)**	0·59<br>(0·36–0·96)<br>(0·27–1·31)	1·09<br>(0·68–1·74)<br>(0·50–2·37)
2·91<br>(2·01–4·21)<br>(1·41–6·00)	3·12<br>(2·08–4·66)<br>(1·48–6·54)	1·04<br>(0·56–1·96)<br>(0·43–2·54)	1·09<br>(0·84–1·42)<br>(0·56–2·14)	1·70<br>(1·04–2·78)<br>(0·77–3·76)	**CT<br>(P-score: 0·12)**	1·84<br>(1·33–2·56)<br>(0·91–3·72)
1·58<br>(1·24–2·01)<br>(0·81–3·07)	1·69<br>(1·26–2·26)<br>(0·85–3·36)	0·57<br>(0·31–1·03)<br>(0·24–1·35)	0·59<br>(0·45–0·79)<br>(0·30–1·17)	0·92<br>(0·58–1·47)<br>(0·42–2·01)	0·54<br>(0·39–0·75)<br>(0·27–1·10)	**LLETZ<br>(P-score: 0·60)**

Heterogeneity: τ^2=0·10; I^2=30% (6–48)

Table 2.5.1.2: Results of individual studies included in the main network meta-analysis for risk of CIN treatment failure (N=71 studies)

Study	Comparison	n1/N1	n2/N2	OR (95% CI)	Effect estimate adjusted for confounding factors
Wright 1981	LA–CT	4/131	22/152	0·19 (0·06–0·56)	no
Townsend 1983	LA–CT	11/100	7/100	1·65 (0·61–4·48)	no (RCT)
Jobson 1984	LA–CT	4/42	4/39	0·92 (0·22–3·94)	no (RCT)
Lele 1984	CKC–CT	1/25	7/35	0·17 (0·02–1·44)	no
Ferenczy 1985	LA–CT	6/147	13/147	0·44 (0·16–1·20)	no
Helmerhorst 1985	LA–CT	18/84	10/81	1·93 (0·83–4·49)	no (RCT)
Hussein 1985	CKC–RD	8/92	16/69	0·32 (0·13–0·80)	no
Hussein 1985	CKC–CC	8/92	7/65	0·79 (0·27–2·27)	no
Hussein 1985	RD–CC	16/69	7/65	2·51 (0·96–6·56)	no
Kirwan 1985	LA–CT	8/71	6/35	0·61 (0·20–1·91)	no (RCT)
Kwikkel 1985	LA–CT	15/51	7/50	2·56 (0·94–6·96)	no (RCT)
Seshadri 1985	CKC–RD	36/592	24/153	0·35 (0·20–0·60)	no
Baggish 1986	LC–LA	3/119	6/100	0·41 (0·10–1·67)	no
Bostofte 1986	CKC–LC	6/57	4/56	1·52 (0·41–5·66)	no (RCT)
O'Shea 1986	RD–CT	3/27	9/30	0·29 (0·07–1·22)	no (RCT)
Need 1988	RD–LA	2/28	1/33	2·46 (0·21–28·50)	no (RCT)

Singh 1988	CC–CT	14/90	15/67	0·64<br>(0·29–1·42)	no (RCT)
Partington 1989	LC–LA	4/48	5/45	0·73<br>(0·18–2·92)	no (RCT)
Yliskoski 1989	LA–CT	8/77	4/42	1·11<br>(0·32–3·87)	no
Gunasekera 1990	LA–LLETZ	8/101	5/98	1·60<br>(0·50–5·09)	no (RCT)
Hellberg 1990	CKC–CT	23/628	14/104	0·24<br>(0·12–0·49)	no
Tabor 1990	CKC–LC	28/201	25/224	1·28<br>(0·73–2·27)	no
Berget 1991	LA–CT	12/98	10/99	1·25<br>(0·52–3·01)	no (RCT)
Goodman 1991	LA–CC	15/67	12/65	1·27<br>(0·55–2·95)	no (RCT)
Martel 1992	LC–LA	1/59	4/25	0·09<br>(0·01–0·86)	no
Guijon 1993	LA–CT	13/160	15/276	1·54<br>(0·72–3·30)	no
Oyesanya 1993 (cohort study)	CKC–LLETZ	2/43	3/43	0·65<br>(0·10–4·11)	no
Oyesanya 1993 (RCT)	LC–LLETZ	25/147	17/148	1·58<br>(0·81–3·08)	no (RCT)
Alvarez 1994	LA–LLETZ	4/96	5/180	1·52<br>(0·40–5·77)	no (RCT)
Sideri 1994	CKC–LLETZ	2/50	9/124	0·53<br>(0·11–2·55)	no
Diakomanolis 1995	LC–LA	5/85	14/228	0·95<br>(0·33–2·74)	no
Baldauf 1996	LC–LLETZ	6/255	15/277	0·42<br>(0·16–1·09)	no
Santos 1996	LC–LLETZ	5/145	7/149	0·73<br>(0·22–2·35)	no (RCT)

Urbaniak 1996	LC–LLETZ	28/155	85/323	0·62 (0·39–0·99)	no
Varawalla 1996	LA–CT	70/200	54/191	1·36 (0·89–2·10)	no
Varawalla 1996	LA–LLETZ	70/200	26/200	3·60 (2·16–5·99)	no
Varawalla 1996	CT–LLETZ	54/191	26/200	2·64 (1·58–4·39)	no
Widrich 1996	CKC–LC	6/24	1/3	0·66 (0·05–8·65)	no
Widrich 1996	CKC–LLETZ	6/24	8/18	0·41 (0·11–1·54)	no
Widrich 1996	LC–LLETZ	1/3	8/18	0·63 (0·05–8·15)	no
Wolf 1996	CKC–LC	17/47	0/1	1·72 (0·07–44·42)	no
Wolf 1996	CKC–LLETZ	17/47	3/7	0·74 (0·16–3·35)	no
Wolf 1996	LC–LLETZ	0/1	3/7	0·43 (0·01–14·00)	no
Gonzalez-Bosquet 1997	CKC–LA	1/25	6/40	0·24 (0·03–2·09)	no
Gonzalez-Bosquet 1997	CKC–LLETZ	1/25	8/58	0·26 (0·03–2·20)	no
Gonzalez-Bosquet 1997	LA–LLETZ	6/40	8/58	1·11 (0·35–3·44)	no
Mitchell 1998	LA–CT	21/121	33/139	0·68 (0·37–1·24)	no (RCT)
Mitchell 1998	LA–LLETZ	21/121	21/130	1·09 (0·56–2·13)	no (RCT)
Mitchell 1998	CT–LLETZ	33/139	21/130	1·62 (0·88–2·97)	no (RCT)
Simmons 1998	CKC–LLETZ	0/22	1/24	0·35 (0·01–8·97)	no

Bornstein 1999	CKC–LA	3/22	2/13	0·87<br>(0·12–6·05)	no
Bornstein 1999	CKC–LLETZ	3/22	8/52	0·87<br>(0·21–3·64)	no
Bornstein 1999	LA–LLETZ	2/13	8/52	1·00<br>(0·19–5·40)	no
Duggan 1999	CKC–LLETZ	8/77	14/78	0·53<br>(0·21–1·36)	no (RCT)
Giacalone 1999	CKC–LLETZ	4/38	6/28	0·43<br>(0·11–1·70)	no (RCT)
Ioffe 1999	CKC–LLETZ	8/24	32/76	0·69<br>(0·26–1·80)	no
Takac 1999	CKC–LLETZ	2/120	5/120	0·39<br>(0·07–2·07)	no (RCT)
Vejerslev_A 1999	LC–LLETZ	3/55	1/65	3·71<br>(0·37–36·72)	no (RCT)
Vejerslev_B 1999	LC–LLETZ	1/38	3/48	0·41<br>(0·04–4·11)	no (RCT)
Husseinzadeh 2000	CKC–LLETZ	11/60	18/77	0·73<br>(0·32–1·70)	no
Persad 2001	LA–CT	90/1126	93/1114	0·95<br>(0·71–1·28)	no
Dey 2002	LA–LLETZ	44/133	34/152	1·72<br>(1·01–2·91)	no (RCT)
Mathevet 2003	CKC–LC	4/35	3/36	1·42<br>(0·30–6·81)	no (RCT)
Mathevet 2003	CKC–LLETZ	4/35	4/36	1·03<br>(0·24–4·48)	no (RCT)
Mathevet 2003	LC–LLETZ	3/36	4/36	0·73<br>(0·15–3·48)	no (RCT)
Omnes 2003	CKC–LLETZ	0/5	0/3	0·64<br>(0·01–39·87)	no
Zielinski 2003	CKC–LLETZ	3/23	3/85	4·10<br>(0·77–21·67)	no

Murta 2004	CKC–LLETZ	26/108	24/71	0·62<br>(0·32–1·20)	no
Dalrymple 2008	CKC–LC	7/34	5/41	1·86<br>(0·53–6·52)	no
Park 2008	CKC–LLETZ	5/77	5/159	2·14<br>(0·60–7·64)	no
Ostojic 2010	CKC–LLETZ	6/151	7/110	0·61<br>(0·20–1·85)	no
Kocken 2011	CKC–LLETZ	12/77	64/358	0·85<br>(0·44–1·66)	no (pooled analysis of two RCTs and one cohort study)
Kietpeerakool 2012	CKC–LLETZ	5/23	13/37	0·51<br>(0·15–1·69)	no
Serati 2012	CKC–LLETZ	13/68	51/214	0·76<br>(0·38–1·50)	no
van Hanegem 2012	CKC–LLETZ	3/55	3/54	0·98<br>(0·19–5·09)	no
Zeng 2012	CKC–LLETZ	2/869	3/74	0·05<br>(0·01–0·33)	no
Taylor 2014	CKC–LLETZ	4/30	4/14	0·38<br>(0·08–1·84)	no
Cai 2015	CKC–LLETZ	1/51	2/64	0·62<br>(0·05–7·03)	no
Kiuchi 2016	LC–LLETZ	6/405	20/146	0·09<br>(0·04–0·24)	no
Mariya 2016	LC–LA	9/101	22/137	0·51<br>(0·22–1·17)	no
Hansen 2017	LA–LLETZ	41/113	35/153	1·92<br>(1·13–3·25)	no
Papoutsis 2017	CC–LLETZ	15/178	6/202	4·48<br>(1·21–16·66)	yes (age, parity, smoking, pre-treatment cytology, pre-treatment colposcopy, number of pre-treatment biopsies, maximum depth of pre-treatment biopsies, endocervical crypt involvement on pre-treatment biopsies)
Wyse 2017	CC–LLETZ	27/200	33/200	0·79<br>(0·45–1·36)	no

Byun 2018	CKC–LLETZ	2/90	4/82	0·44<br>(0·08–2·50)	no
Bogani 2020	LC–LLETZ	20/500	81/1000	0·47<br>(0·28–0·79)	no
Sun 2020	CKC–LLETZ	3/22	53/107	0·16<br>(0·04–0·57)	no
Duan 2021	CC–CT	2/69	5/74	0·41<br>(0·08–2·17)	no (RCT)

2.5.2. Preterm Birth

Table 2.5.2.1: Network meta-analysis for risk of preterm birth after CIN treatments (N=29 studies)

**CKC<br>(P-score: 0·09)**	1·28<br>(0·88–1·85)<br>(0·78–2·10)	1·65<br>(1·28–2·13)<br>(1·10–2·49)	1·21<br>(0·77–1·88)<br>(0·69–2·11)	2·16<br>(1·48–3·15)<br>(1·30–3·57)	3·37<br>(0·08–146·96)<br>(0·07–171·80)	2·24<br>(0·77–6·45)<br>(0·71–7·02)	2·27<br>(1·70–3·02)<br>(1·47–3·50)
0·78<br>(0·54–1·13)<br>(0·48–1·28)	**LC<br>(P-score: 0·29)**	1·29<br>(0·97–1·72)<br>(0·84–1·99)	0·94<br>(0·59–1·5)<br>(0·53–1·68)	1·69<br>(1·15–2·46)<br>(1·02–2·79)	2·63<br>(0·06–115·14)<br>(0·05–134·62)	1·75<br>(0·60–5·07)<br>(0·55–5·52)	1·77<br>(1·29–2·43)<br>(1·12–2·79)
0·61<br>(0·47–0·78)<br>(0·40–0·91)	0·77<br>(0·58–1·03)<br>(0·50–1·20)	**LLETZ<br>(P-score: 0·51)**	0·73<br>(0·50–1·08)<br>(0·44–1·22)	1·31<br>(0·96–1·77)<br>(0·83–2·04)	2·04<br>(0·05–88·20)<br>(0·04–103·08)	1·35<br>(0·47–3·86)<br>(0·44–4·20)	1·37<br>(1·16–1·62)<br>(0·96–1·96)
0·83<br>(0·53–1·29)<br>(0·47–1·45)	1·06<br>(0·67–1·69)<br>(0·60–1·89)	1·37<br>(0·93–2·02)<br>(0·82–2·28)	**RD<br>(P-score: 0·24)**	1·79<br>(1·19–2·67)<br>(1·06–3·02)	2·79<br>(0·06–123·21)<br>(0·05–144·10)	1·85<br>(0·61–5·64)<br>(0·56–6·14)	1·88<br>(1·30–2·72)<br>(1·14–3·08)
0·46<br>(0·32–0·68)<br>(0·28–0·77)	0·59<br>(0·41–0·87)<br>(0·36–0·98)	0·77<br>(0·56–1·04)<br>(0·49–1·20)	0·56<br>(0·37–0·84)<br>(0·33–0·94)	**LA<br>(P-score: 0·74)**	1·56<br>(0·04–68·42)<br>(0·03–79·99)	1·04<br>(0·35–3·07)<br>(0·32–3·34)	1·05<br>(0·78–1·41)<br>(0·68–1·63)
0·30<br>(0·01–12·93)<br>(0·01–15·11)	0·38<br>(0·01–16·58)<br>(0·01–19·39)	0·49<br>(0·01–21·18)<br>(0·01–24·75)	0·36<br>(0·01–15·78)<br>(0·01–18·45)	0·64<br>(0·01–27·99)<br>(0·01–32·72)	**CC<br>(P-score: 0·65)**	0·66<br>(0·01–33·07)<br>(0·01–38·84)	0·67<br>(0·02–29·15)<br>(0·01–34·07)
0·45<br>(0·15–1·29)<br>(0·14–1·41)	0·57<br>(0·20–1·66)<br>(0·18–1·81)	0·74<br>(0·26–2·11)<br>(0·24–2·30)	0·54<br>(0·18–1·64)<br>(0·16–1·79)	0·96<br>(0·33–2·86)<br>(0·30–3·11)	1·51<br>(0·03–75·23)<br>(0·03–88·37)	**CT<br>(P-score: 0·68)**	1·01<br>(0·35–2·92)<br>(0·32–3·18)
0·44<br>(0·33–0·59)<br>(0·29–0·68)	0·56<br>(0·41–0·77)<br>(0·36–0·89)	0·73<br>(0·62–0·86)<br>(0·51–1·04)	0·53<br>(0·37–0·77)<br>(0·32–0·87)	0·95<br>(0·71–1·28)<br>(0·61–1·47)	1·49<br>(0·03–64·55)<br>(0·03–75·44)	0·99<br>(0·34–2·84)<br>(0·31–3·10)	**COLPO<br>(P-score: 0·79)**

Heterogeneity: τ^2=0·02; I^2=22% (0–49)

Table 2.5.2.2: Results of individual studies included in the main network meta-analysis for risk of preterm birth after CIN treatments (N=29 studies)

Study	Comparison	n1/N1	n2/N2	OR (95% CI)	Effect estimate adjusted for confounding factors
Wakita 1990	LC–LA	0/4	1/9	0·63 (0·02–18·74)	no
Gunasekera 1992	LLETZ–LA	0/22	2/109	0·59 (0·01–50·87)	no
Loizzi 1992	CKC–CT	4/29	1/7	0·96 (0·09–10·29)	no
Spitzer 1995	LC–LA	2/34	16/129	0·44 (0·10–2·03)	no
Mathevet 2003	CKC–LC	0/9	0/17	1·84 (0·03–100·32)	no (RCT)
Mathevet 2003	CKC–LLETZ	0/9	1/9	0·30 (0·01–8·35)	no (RCT)
Mathevet 2003	LC–LLETZ	0/17	1/9	0·16 (0·01–4·45)	no (RCT)
Sadler 2004	LC–COLPO	20/105	52/426	1·35 (0·74–2·48)	yes (age, parity, smoking, ethnicity, socioeconomic status, history of preterm birth, antepartum haemorrhage, interhospital transfer; variables were manually removed if they were not found to be important confounders)
Sadler 2004	LLETZ–COLPO	44/278	52/426	1·23 (0·77–1·97)	as above
Sadler 2004	LA–COLPO	23/223	52/426	0·78 (0·48–1·27)	as above
Sadler 2004	LC–LLETZ	20/105	44/278	1·09 (0·56–2·13)	as above
Sadler 2004	LC–LA	20/105	23/223	1·75 (0·90–3·41)	as above
Sadler 2004	LLETZ–LA	44/278	23/223	1·58 (0·92–2·74)	as above

Crane 2006	CKC–LLETZ	4/21	10/75	1·52<br>(0·43–5·44)	yes (age, parity, smoking, antepartum haemorrhage, history of spontaneous preterm birth, gestational age at the time of ultrasound scan; only variables with P<0·10 were kept in the final model)
Crane 2006	CKC–CT	4/21	1/36	8·25<br>(0·85–80·13)	as above
Crane 2006	LLETZ–CT	10/75	1/36	5·37<br>(0·66–43·69)	as above
Bruinsma 2007	CKC–LLETZ	11/71	11/69	0·96<br>(0·38–2·46)	yes (age, parity, marital status, maternal medical conditions, country of origin, history of miscarriage, history of preterm birth, illicit drug use; parity and country of origin were not included in the final model because of non-significant contribution)
Bruinsma 2007	CKC–RD	11/71	109/760	0·91<br>(0·45–1·85)	as above
Bruinsma 2007	CKC–LA	11/71	92/1005	1·62<br>(0·80–3·27)	as above
Bruinsma 2007	CKC–COLPO	11/71	309/3484	1·72<br>(0·88–3·34)	as above
Bruinsma 2007	LLETZ–RD	11/69	109/760	0·95<br>(0·47–1·93)	as above
Bruinsma 2007	LLETZ–LA	11/69	92/1005	1·68<br>(0·83–3·41)	as above
Bruinsma 2007	LLETZ–COLPO	11/69	309/3484	1·79<br>(0·90–3·55)	as above
Bruinsma 2007	RD–LA	109/760	92/1005	1·68<br>(1·23–2·30)	as above
Bruinsma 2007	RD–COLPO	109/760	309/3484	1·88<br>(1·46–2·42)	as above
Bruinsma 2007	LA–COLPO	92/1005	309/3484	1·06<br>(0·82–1·37)	as above
Bull-Phelps 2007	CKC–LLETZ	2/31	0/6	1·11<br>(0·05–25·94)	no

Himes 2007	LLETZ–COLPO	11/114	86/962	1·08 (0·56–2·11)	no
Patrelli 2008	CKC–LC	3/32	0/3	0·83 (0·04–19·41)	no
Patrelli 2008	CKC–LLETZ	3/32	5/45	0·87 (0·21–3·64)	no
Patrelli 2008	LC–LLETZ	0/3	5/45	1·05 (23·26–0·05)	no
Michelin 2009	CKC–LLETZ	4/17	1/18	5·21 (0·52–52·60)	no
Noehr 2009 (AJOG)	LLETZ–COLPO	530/8180	1318/31630	1·65 (1·49–1·82)	yes (age, smoking, marital status, year of delivery)
Nam 2010	CKC–LLETZ	9/14	9/51	8·41 (2·26–31·29)	no
Lima 2011	LC–LLETZ	2/11	4/18	0·78 (0·12–5·21)	no
Reilly 2012	LLETZ–COLPO	146/1546	209/2534	1·14 (0·90–1·44)	yes (age, parity, smoking, social deprivation, time to conception, history of adverse pregnancy outcomes)
Simoens 2012	LC–LLETZ	5/24	12/52	0·45 (0·08–2·67)	yes (age, parity, smoking, ethnicity, education, HIV)
Frey 2013	LLETZ–COLPO	111/598	91/552	1·15 (0·84–1·57)	no
Guo 2013	CKC–LLETZ	14/36	10/48	2·41 (0·92–6·30)	no
Guo 2013	CKC–COLPO	14/36	14/68	2·46 (1·02–5·94)	no
Guo 2013	LLETZ–COLPO	10/48	14/68	1·01 (0·41–2·49)	no
Kitson 2014	LLETZ–COLPO	25/278	10/278	2·64 (1·18–5·89)	no
Liu 2014	CKC–LLETZ	13/115	6/121	2·44 (0·90–6·62)	no (RCT)
Cai 2015	CKC–LLETZ	3/43	4/54	0·94 (0·20–4·43)	no

Martyn 2015	LLETZ–COLPO	20/278	6/204	2·41 (0·96–6·06)	no
Bjorge 2016	CKC–LC	NA/61	NA/3587	1·15 (0·50–2·67)*	yes (age, parity, smoking, marital status, education, country of origin, year of delivery, history of cytology screening)
Bjorge 2016	CKC–LLETZ	NA/61	NA/4361	1·84 (0·79–4·28)*	as above
Bjorge 2016	CKC–LA	NA/61	NA/96	1·72 (0·45–6·51)*	as above
Bjorge 2016	CKC–CT	NA/61	NA/24	1·62 (0·32–8·06)*	as above
Bjorge 2016	LC–LLETZ	NA/3587	NA/4361	1·60 (1·32–1·95)*	as above
Bjorge 2016	LC–LA	NA/3587	NA/96	1·49 (0·52–4·30)*	as above
Bjorge 2016	LC–CT	NA/3587	NA/24	1·40 (0·36–5·54)*	as above
Bjorge 2016	LLETZ–LA	NA/4361	NA/96	0·93 (0·32–2·69)*	as above
Bjorge 2016	LLETZ–CT	NA/4361	NA/24	0·87 (0·22–3·43)*	as above
Bjorge 2016	LA–CT	NA/96	NA/24	0·93 (0·17–5·23)*	as above
Jancar 2016	CKC–LLETZ	267/2083	204/2498	1·60 (1·32–1·95)	no
Mariya 2016	LC–LA	4/14	2/15	2·61 (0·40–17·14)	no
Heinonen 2018	LLETZ–COLPO	53/797	116/220	1·90 (0·97–3·69)	yes (age, socioeconomic status, marital status, history of preterm birth)
Papoutsis 2018	LLETZ–CC	4/60	5/56	2·03 (0·05–87·64)	yes (age, parity, smoking, pre-treatment cytology, pre-treatment colposcopy, number of pre-treatment biopsies, maximum depth of pre-treatment biopsies)
Zhang 2020	CKC–LLETZ	1/36	1/37	0·46 (0·03–7·78)	no

*Authors reported hazard ratios (HRs); we assumed that HR=RR before calculating OR

2.5.3. Trade-off between risk of treatment failure and risk of preterm birth

Figure 2.5.3.1: Odds ratios of treatment failure and preterm birth after various CIN treatments compared to LLETZ

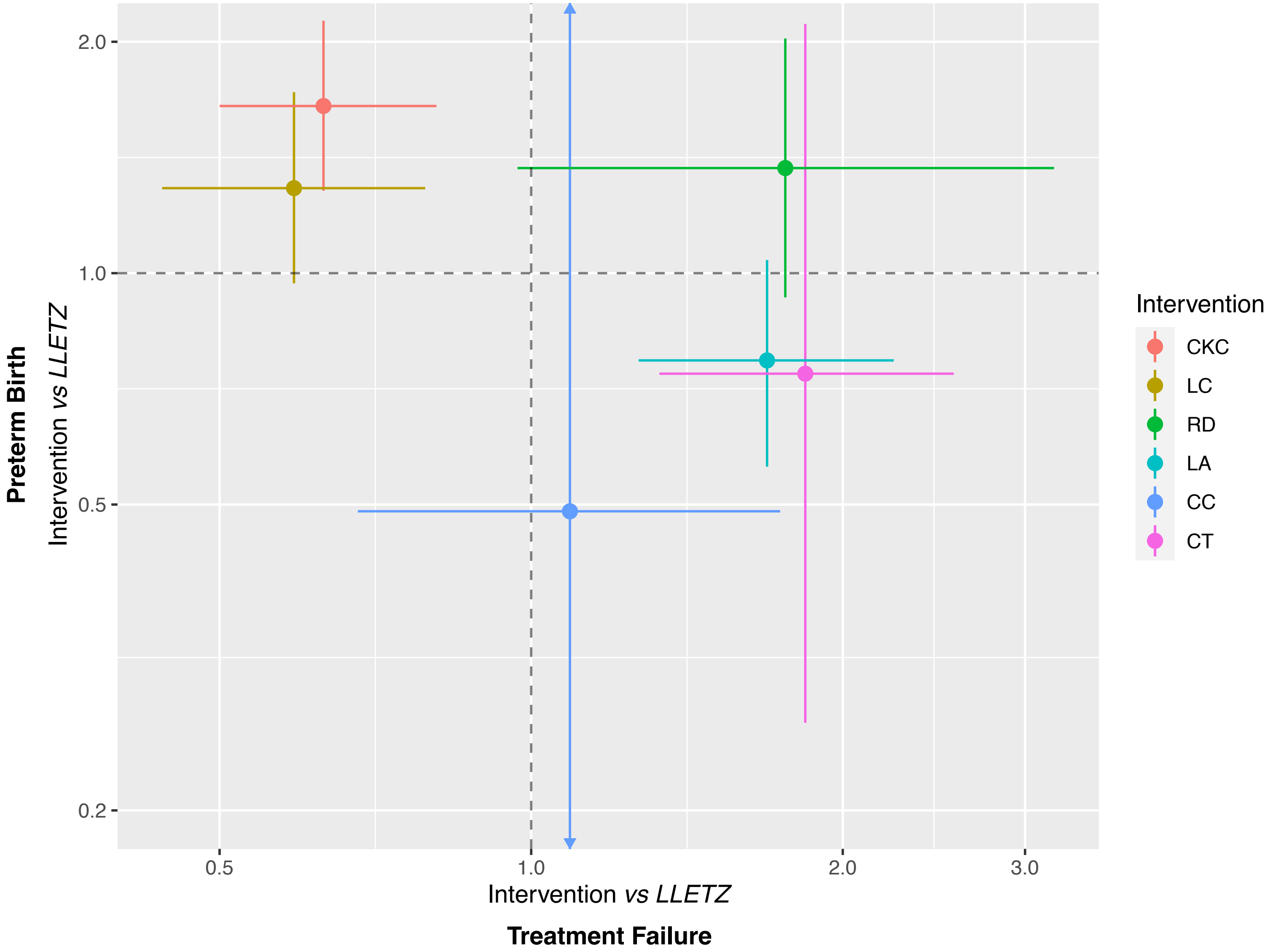

The x and y axis shows the ORs of treatment failure and preterm birth, respectively, after various CIN treatments compared to LLETZ. The width or height of each lines represents the 95% CI. ORs>1 favour LLETZ, whilst ORs<1 favour the intervention.

2.6. Tests for Inconsistency

To assess inconsistency, we used both back-calculation and design-by-treatment interaction model.

2.6.1. Treatment Failure

Table 2.6.1.1: Tests of inconsistency for risk of CIN treatment failure

Comparison	N studies	Proportion of direct evidence	OR from direct evidence (95% CI)	OR from indirect evidence (95% CI)	Ratio of odds ratios* (95% CI)	p-value (back-calculation method)
CKC–LC	6	34%	1·40 (0·78–2·49)	0·93 (0·62–1·42)	0·67 (0·33–1·36)	0·27
CKC–RD	2	76%	0·34 (0·17–0·65)	0·44 (0·14–1·41)	1·31 (0·35–5·00)	0·69
CKC–LA	2	5%	0·49 (0·11–2·20)	0·37 (0·26–0·53)	0·76 (0·16–3·59)	0·73
CKC–CC	1	17%	0·79 (0·23–2·70)	0·55 (0·32–0·95)	0·70 (0·18–2·67)	0·60
CKC–CT	2	19%	0·23 (0·10–0·54)	0·38 (0·25–0·57)	1·63 (0·63–4·21)	0·32
CKC–LLETZ	27	78%	0·62 (0·47–0·81)	0·68 (0·41–1·14)	1·10 (0·61–1·97)	0·75
LC–RD	0	0%	-	0·34 (0·18–0·64)	-	-
LC–LA	5	34%	0·54 (0·29–1·00)	0·28 (0·18–0·43)	1·94 (0·92–4·13)	0·084
LC–CC	0	0%	-	0·54 (0·32–0·93)	-	-
LC–CT	0	0%	-	0·32 (0·21–0·48)	-	-
LC–LLETZ	11	65%	0·56 (0·39–0·81)	0·65 (0·40–1·06)	0·87 (0·47–1·60)	0·65
RD–LA	1	6%	2·46 (0·20–30·89)	0·99 (0·52–1·88)	2·49 (0·18–33·86)	0·49
RD–CC	1	36%	2·50 (0·80–7·81)	1·27 (0·54–2·99)	1·97 (0·47–8·17)	0·35
RD–CT	1	16%	0·29 (0·06–1·38)	1·21 (0·61–2·40)	0·24 (0·04–1·32)	0·11

RD–LLETZ	0	0%	-	1·76 (0·97–3·20)	-	-
LA–CC	1	21%	1·27 (0·45–3·62)	1·64 (0·95–2·84)	0·78 (0·24–2·52)	0·67
LA–CT	13	85%	1·02 (0·77–1·36)	0·50 (0·26–0·97)	2·05 (0·99–4·22)	0·052
LA–LLETZ	8	63%	1·78 (1·24–2·54)	1·55 (0·97–2·47)	1·15 (0·64–2·06)	0·65
CC–CT	2	31%	0·57 (0·24–1·38)	0·60 (0·33–1·08)	0·96 (0·33–2·78)	0·94
CC–LLETZ	2	43%	1·20 (0·59–2·46)	1·01 (0·54–1·88)	1·20 (0·46–3·09)	0·71
CT–LLETZ	2	31%	2·10 (1·17–3·78)	1·74 (1·17–2·58)	1·21 (0·60–2·46)	0·60

*OR from direct data divided by OR from indirect data

P-value for inconsistency from design-by-treatment interaction test: 0·57

2.6.2. Preterm Birth

Table 2.6.2.1: Tests of inconsistency for risk of preterm birth after CIN treatments

Comparison	N studies	Proportion of direct evidence	OR from direct evidence (95% CI)	OR from indirect evidence (95% CI)	Ratio of odds ratios* (95% CI)	p-value (back-calculation method)
CKC–LC	3	19%	1·15 (0·50–2·66)	1·31 (0·87–1·98)	0·87 (0·34–2·23)	0·78
CKC–LLETZ	13	92%	1·69 (1·29–2·19)	1·31 (0·53–3·21)	1·29 (0·51–3·27)	0·60
CKC–RD	1	34%	0·91 (0·42–1·95)	1·39 (0·81–2·41)	0·65 (0·26–1·67)	0·37
CKC–LA	2	32%	1·64 (0·84–3·20)	2·46 (1·55–3·89)	0·67 (0·30–1·50)	0·33
CKC–CC	0	0%	-	3·37 (0·08–147·06)	-	-
CKC–CT	3	84%	2·17 (0·68–6·92)	2·61 (0·19–35·97)	0·83 (0·05–14·66)	0·90
CKC–COLPO	2	24%	1·96 (1·10–3·51)	2·37 (1·71–3·30)	0·83 (0·42–1·62)	0·58
LC–LLETZ	6	87%	1·38 (1·01–1·89)	0·82 (0·37–1·81)	1·70 (0·72–4·00)	0·23
LC–RD	0	0%	-	0·94 (0·59–1·50)	-	-
LC–LA	5	50%	1·44 (0·84–2·45)	1·98 (1·16–3·39)	0·73 (0·34–1·55)	0·41
LC–CC	0	0%	-	2·63 (0·06–115·14)	-	-
LC–CT	1	57%	1·40 (0·34–5·72)	2·35 (0·46–12·00)	0·60 (0·07–5·14)	0·64
LC–COLPO	1	21%	1·36 (0·69–2·68)	1·90 (1·33–2·72)	0·71 (0·33–1·54)	0·39
LLETZ–RD	1	25%	0·95 (0·44–2·05)	0·67 (0·43–1·05)	1·42 (0·58–3·46)	0·45
LLETZ–LA	4	48%	1·47 (0·94–2·29)	1·17 (0·77–1·79)	1·25 (0·68–2·31)	0·47

LLETZ–CC	1	100%	2·04<br>(0·05–88·20)	NA<br>(NA, NA)	-	-
LLETZ–CT	2	80%	1·52<br>(0·47–4·93)	0·84<br>(0·08–8·75)	1·81<br>(0·13–24·91)	0·66
LLETZ–COLPO	10	92%	1·41<br>(1·19–1·68)	0·98<br>(0·54–1·75)	1·45<br>(0·79–2·66)	0·23
RD–LA	1	86%	1·77<br>(1·15–2·74)	1·87<br>(0·65–5·43)	0·95<br>(0·30–2·99)	0·93
RD–CC	0	0%	-	2·79<br>(0·06–123·21)	-	-
RD–CT	0	0%	-	1·85<br>(0·61–5·64)	-	-
RD–COLPO	1	89%	1·88<br>(1·27–2·78)	1·86<br>(0·60–5·76)	1·01<br>(0·31–3·34)	0·99
LA–CC	0	0%	-	1·56<br>(0·04–68·42)	-	-
LA–CT	1	38%	0·94<br>(0·16–5·39)	1·10<br>(0·28–4·41)	0·85<br>(0·09–7·89)	0·88
LA–COLPO	2	82%	0·96<br>(0·69–1·33)	1·57<br>(0·79–3·11)	0·61<br>(0·29–1·30)	0·20
CC–CT	0	0%	-	0·66<br>(0·01–33·07)	-	-
CC–COLPO	0	0%	-	0·67<br>(0·02–29·15)	-	-
CT–COLPO	0	0%	-	1·01<br>(0·35–2·92)	-	-

*OR from direct data divided by OR from indirect data
p-value for inconsistency from design-by-treatment interaction test: 0·54

2.7. Design-Adjusted Network Meta-Analyses

In this section we present the results of the design-adjusted NMAs. We performed four different inflation variance models and in each model we progressively down-weighted NRS as follows:

In inflation variance **model 1**, the weight of NRS at low, moderate and high RoB was decreased by 0, 20% and 40%, respectively. RCTs were not down-weighted.
In inflation variance **model 2**, the weight of NRS at low, moderate and high RoB was decreased by 20%, 40% and 60%, respectively. RCTs were not down-weighted.
In inflation variance **model 3**, the weight of NRS at low, moderate and high RoB was decreased by 40%, 60% and 80%, respectively. RCTs were not down-weighted.
In inflation variance **model 4**, the weight of NRS at low, moderate and high RoB was decreased by 60%, 80% and 100%, respectively. RCTs were not down-weighted.

In the unadjusted (or 'naïve' or 'traditional') NMA we used the original weights of all studies (i.e. we did not down-weight any studies).

Design-adjusted analyses are presented in league tables, where each box represents the comparison of the row-defining treatment vs the column-defining treatment. OR is reported first, followed by 95% CI and 95% PI. ORs>1 favour the column-defining treatment, while ORs<1 favour the row-defining treatment. After league tables we present the results of all design-adjusted analyses in a summary figure.

2.7.1. Treatment Failure

Table 2.7.1.1: Unadjusted NMA for risk of CIN treatment failure (main analysis) (N=71 studies)

**CKC<br>(P-score: 0·89)**	1·07<br>(0·76–1·50)<br>(0·53–2·17)	0·36<br>(0·20–0·64)<br>(0·15–0·84)	0·38<br>(0·27–0·53)<br>(0·18–0·76)	0·58<br>(0·35–0·96)<br>(0·26–1·30)	0·34<br>(0·24–0·50)<br>(0·17–0·71)	0·63<br>(0·50–0·81)<br>(0·33–1·23)
0·93<br>(0·67–1·31)<br>(0·46–1·89)	**LC<br>(P-score: 0·94)**	0·34<br>(0·18–0·64)<br>(0·14–0·82)	0·35<br>(0·25–0·50)<br>(0·17–0·72)	0·54<br>(0·32–0·93)<br>(0·24–1·24)	0·32<br>(0·21–0·48)<br>(0·15–0·67)	0·59<br>(0·44–0·79)<br>(0·30–1·17)
2·79<br>(1·57–4·94)<br>(1·19–6·51)	2·98<br>(1·57–5·67)<br>(1·21–7·33)	**RD<br>(P-score: 0·19)**	1·04<br>(0·56–1·95)<br>(0·43–2·53)	1·62<br>(0·82–3·22)<br>(0·64–4·12)	0·96<br>(0·51–1·80)<br>(0·39–2·33)	1·76<br>(0·97–3·20)<br>(0·74–4·19)
2·67<br>(1·89–3·75)<br>(1·31–5·42)	2·86<br>(2·00–4·08)<br>(1·39–5·85)	0·96<br>(0·51–1·78)<br>(0·40–2·32)	**LA<br>(P-score: 0·22)**	1·55<br>(0·96–2·52)<br>(0·71–3·43)	0·92<br>(0·71–1·19)<br>(0·47–1·80)	1·69<br>(1·27–2·24)<br>(0·85–3·34)
1·72<br>(1·04–2·83)<br>(0·77–3·82)	1·84<br>(1·08–3·13)<br>(0·81–4·19)	0·62<br>(0·31–1·22)<br>(0·24–1·56)	0·64<br>(0·40–1·04)<br>(0·29–1·42)	**CC<br>(P-score: 0·54)**	0·59<br>(0·36–0·96)<br>(0·27–1·31)	1·09<br>(0·68–1·74)<br>(0·50–2·37)
2·91<br>(2·01–4·21)<br>(1·41–6·00)	3·12<br>(2·08–4·66)<br>(1·48–6·54)	1·04<br>(0·56–1·96)<br>(0·43–2·54)	1·09<br>(0·84–1·42)<br>(0·56–2·14)	1·70<br>(1·04–2·78)<br>(0·77–3·76)	**CT<br>(P-score: 0·12)**	1·84<br>(1·33–2·56)<br>(0·91–3·72)
1·58<br>(1·24–2·01)<br>(0·81–3·07)	1·69<br>(1·26–2·26)<br>(0·85–3·36)	0·57<br>(0·31–1·03)<br>(0·24–1·35)	0·59<br>(0·45–0·79)<br>(0·30–1·17)	0·92<br>(0·58–1·47)<br>(0·42–2·01)	0·54<br>(0·39–0·75)<br>(0·27–1·10)	**LLETZ<br>(P-score: 0·60)**

Heterogeneity: τ^2=0·10; I^2=30% (6–48)

Table 2.7.1.2: Inflation variance model 1 for risk of CIN treatment failure (N=71 studies)

**CKC (P-score: 0·90)**	1·04 (0·71–1·50) (0·50–2·14)	0·38 (0·20–0·72) (0·15–0·93)	0·37 (0·25–0·54) (0·18–0·77)	0·59 (0·34–1·03) (0·25–1·36)	0·35 (0·23–0·52) (0·17–0·73)	0·64 (0·48–0·84) (0·32–1·26)
0·97 (0·67–1·40) (0·47–1·99)	**LC (P-score: 0·92)**	0·36 (0·18–0·75) (0·14–0·95)	0·36 (0·24–0·53) (0·17–0·75)	0·57 (0·32–1·02) (0·24–1·35)	0·34 (0·22–0·52) (0·16–0·72)	0·62 (0·45–0·85) (0·31–1·24)
2·66 (1·39–5·09) (1·08–6·57)	2·75 (1·34–5·65) (1·06–7·17)	**RD (P-score: 0·22)**	0·99 (0·49–1·98) (0·39–2·52)	1·57 (0·72–3·39) (0·58–4·26)	0·92 (0·46–1·86) (0·36–2·37)	1·70 (0·87–3·32) (0·67–4·27)
2·69 (1·84–3·94) (1·30–5·58)	2·79 (1·88–4·13) (1·33–5·82)	1·01 (0·51–2·03) (0·40–2·59)	**LA (P-score: 0·20)**	1·59 (0·94–2·67) (0·70–3·58)	0·94 (0·71–1·23) (0·48–1·84)	1·72 (1·27–2·33) (0·86–3·43)
1·70 (0·97–2·96) (0·73–3·92)	1·76 (0·98–3·16) (0·74–4·15)	0·64 (0·29–1·38) (0·23–1·73)	0·63 (0·37–1·06) (0·28–1·42)	**CC (P-score: 0·54)**	0·59 (0·35–1·00) (0·26–1·33)	1·08 (0·65–1·82) (0·48–2·44)
2·88 (1·92–4·32) (1·37–6·06)	2·98 (1·93–4·61) (1·39–6·38)	1·08 (0·54–2·18) (0·42–2·78)	1·07 (0·82–1·40) (0·54–2·10)	1·70 (1·00–2·87) (0·75–3·84)	**CT (P-score: 0·13)**	1·84 (1·29–2·61) (0·90–3·75)
1·57 (1·19–2·06) (0·79–3·09)	1·62 (1·18–2·23) (0·81–3·26)	0·59 (0·30–1·15) (0·23–1·48)	0·58 (0·43–0·79) (0·29–1·16)	0·92 (0·55–1·55) (0·41–2·08)	0·54 (0·38–0·77) (0·27–1·11)	**LLETZ (P-score: 0·59)**

Heterogeneity: τ^2=0·10; I^2=9% (0–32)

Table 2.7.1.3: Inflation variance model 2 for risk of CIN treatment failure (N=71 studies)

**CKC (P-score: 0·91)**	1·00 (0·67–1·50) (0·48–2·10)	0·39 (0·19–0·81) (0·15–1·03)	0·37 (0·24–0·56) (0·18–0·78)	0·59 (0·32–1·09) (0·25–1·42)	0·35 (0·23–0·55) (0·16–0·76)	0·64 (0·47–0·87) (0·32–1·28)
1·00 (0·67–1·50) (0·48–2·10)	**LC (P-score: 0·90)**	0·40 (0·18–0·87) (0·14–1·09)	0·37 (0·24–0·57) (0·17–0·79)	0·59 (0·31–1·12) (0·24–1·45)	0·35 (0·22–0·57) (0·16–0·77)	0·64 (0·46–0·90) (0·32–1·30)
2·53 (1·23–5·22) (0·97–6·63)	2·53 (1·14–5·60) (0·92–7·00)	**RD (P-score: 0·24)**	0·94 (0·44–2·01) (0·35–2·53)	1·50 (0·64–3·52) (0·52–4·36)	0·90 (0·42–1·93) (0·33–2·42)	1·62 (0·77–3·42) (0·61–4·32)
2·70 (1·78–4·09) (1·28–5·71)	2·70 (1·76–4·13) (1·27–5·74)	1·07 (0·50–2·28) (0·40–2·87)	**LA (P-score: 0·18)**	1·60 (0·92–2·78) (0·70–3·69)	0·96 (0·72–1·27) (0·48–1·89)	1·73 (1·25–2·39) (0·86–3·49)
1·69 (0·92–3·10) (0·70–4·04)	1·68 (0·89–3·18) (0·69–4·12)	0·67 (0·28–1·56) (0·23–1·93)	0·62 (0·36–1·08) (0·27–1·44)	**CC (P-score: 0·54)**	0·60 (0·34–1·04) (0·26–1·38)	1·08 (0·62–1·89) (0·47–2·50)
2·82 (1·81–4·40) (1·31–6·07)	2·82 (1·76–4·51) (1·29–6·16)	1·11 (0·52–2·39) (0·41–3·01)	1·05 (0·79–1·39) (0·53–2·07)	1·67 (0·96–2·91) (0·73–3·86)	**CT (P-score: 0·13)**	1·81 (1·25–2·62) (0·88–3·73)
1·56 (1·15–2·12) (0·78–3·12)	1·56 (1·11–2·19) (0·77–3·17)	0·62 (0·29–1·30) (0·23–1·64)	0·58 (0·42–0·80) (0·29–1·17)	0·93 (0·53–1·62) (0·40–2·15)	0·55 (0·38–0·80) (0·27–1·14)	**LLETZ (P-score: 0·59)**

Heterogeneity: τ^2=0·10; I^2=0% (0–28)

Table 2.7.1.4: Inflation variance model 3 for risk of CIN treatment failure (N=71 studies)

**CKC (P-score: 0·91)**	0·94 (0·59–1·50) (0·43–2·06)	0·44 (0·18–1·06) (0·15–1·31)	0·37 (0·23–0·60) (0·17–0·82)	0·60 (0·30–1·21) (0·23–1·54)	0·37 (0·22–0·61) (0·16–0·83)	0·65 (0·45–0·94) (0·31–1·33)
1·06 (0·67–1·69) (0·49–2·31)	**LC (P-score: 0·87)**	0·47 (0·18–1·20) (0·15–1·46)	0·40 (0·24–0·64) (0·18–0·87)	0·63 (0·31–1·30) (0·24–1·65)	0·39 (0·23–0·66) (0·17–0·88)	0·69 (0·47–1·01) (0·33–1·42)
2·27 (0·94–5·46) (0·77–6·72)	2·14 (0·84–5·48) (0·69–6·68)	**RD (P-score: 0·31)**	0·85 (0·35–2·07) (0·28–2·54)	1·36 (0·50–3·70) (0·41–4·47)	0·83 (0·34–2·03) (0·28–2·50)	1·47 (0·60–3·57) (0·49–4·38)
2·68 (1·66–4·34) (1·22–5·90)	2·53 (1·56–4·09) (1·15–5·57)	1·18 (0·48–2·88) (0·39–3·54)	**LA (P-score: 0·16)**	1·60 (0·88–2·92) (0·67–3·82)	0·99 (0·73–1·33) (0·49–1·97)	1·73 (1·21–2·48) (0·85–3·55)
1·67 (0·83–3·37) (0·65–4·30)	1·58 (0·77–3·23) (0·61–4·10)	0·74 (0·27–2·01) (0·22–2·43)	0·62 (0·34–1·14) (0·26–1·49)	**CC (P-score: 0·53)**	0·61 (0·34–1·12) (0·26–1·46)	1·08 (0·58–2·03) (0·44–2·64)
2·72 (1·63–4·53) (1·21–6·10)	2·56 (1·52–4·34) (1·13–5·81)	1·20 (0·49–2·92) (0·40–3·59)	1·01 (0·75–1·37) (0·51–2·02)	1·63 (0·90–2·95) (0·68–3·87)	**CT (P-score: 0·14)**	1·76 (1·17–2·65) (0·83–3·70)
1·55 (1·07–2·23) (0·75–3·18)	1·46 (0·99–2·14) (0·70–3·03)	0·68 (0·28–1·66) (0·23–2·04)	0·58 (0·40–0·83) (0·28–1·18)	0·92 (0·49–1·74) (0·38–2·26)	0·57 (0·38–0·86) (0·27–1·20)	**LLETZ (P-score: 0·57)**

Heterogeneity: τ^2=0·10; I^2=0% (0–28)

Table 2.7.1.5: Inflation variance model 4 for risk of CIN treatment failure (N=37 studies)

**CKC (P-score: 0·82)**	0·87 (0·47–1·60) (0·36–2·13)	0·85 (0·19–3·79) (0·16–4·54)	0·43 (0·22–0·85) (0·17–1·11)	0·67 (0·26–1·77) (0·21–2·20)	0·45 (0·22–0·93) (0·17–1·21)	0·71 (0·41–1·22) (0·30–1·65)
1·15 (0·62–2·12) (0·47–2·81)	**LC (P-score: 0·72)**	0·97 (0·22–4·23) (0·19–5·07)	0·49 (0·27–0·91) (0·20–1·21)	0·77 (0·31–1·94) (0·25–2·43)	0·52 (0·26–1·01) (0·20–1·32)	0·82 (0·51–1·31) (0·37–1·82)
1·18 (0·26–5·30) (0·22–6·35)	1·03 (0·24–4·48) (0·20–5·37)	**RD (P-score: 0·63)**	0·51 (0·13–1·95) (0·11–2·35)	0·80 (0·18–3·52) (0·15–4·22)	0·53 (0·14–2·00) (0·12–2·42)	0·84 (0·21–3·42) (0·17–4·11)
2·33 (1·17–4·64) (0·90–6·05)	2·03 (1·10–3·76) (0·83–4·99)	1·97 (0·51–7·60) (0·42–9·16)	**LA (P-score: 0·12)**	1·57 (0·78–3·17) (0·60–4·12)	1·05 (0·74–1·48) (0·51–2·16)	1·66 (1·07–2·56) (0·76–3·60)
1·48 (0·56–3·90) (0·45–4·86)	1·29 (0·52–3·24) (0·41–4·06)	1·26 (0·28–5·56) (0·24–6·66)	0·64 (0·32–1·29) (0·24–1·67)	**CC (P-score: 0·52)**	0·67 (0·33–1·33) (0·26–1·73)	1·05 (0·47–2·36) (0·37–3·01)
2·23 (1·07–4·64) (0·83–5·99)	1·94 (0·99–3·79) (0·76–4·97)	1·89 (0·50–7·12) (0·41–8·60)	0·96 (0·68–1·35) (0·46–1·98)	1·50 (0·75–2·99) (0·58–3·90)	**CT (P-score: 0·16)**	1·58 (0·96–2·62) (0·70–3·60)
1·41 (0·82–2·42) (0·60–3·28)	1·23 (0·76–1·97) (0·55–2·73)	1·19 (0·29–4·85) (0·24–5·83)	0·60 (0·39–0·93) (0·28–1·31)	0·95 (0·42–2·13) (0·33–2·71)	0·63 (0·38–1·05) (0·28–1·43)	**LLETZ (P-score: 0·54)**

Heterogeneity: τ^2=0·10; I^2=0% (0–38)

Summary Figure
Figure 2.7.1.1: Inflation variance models (design-adjusted analyses) for risk of CIN treatment failure

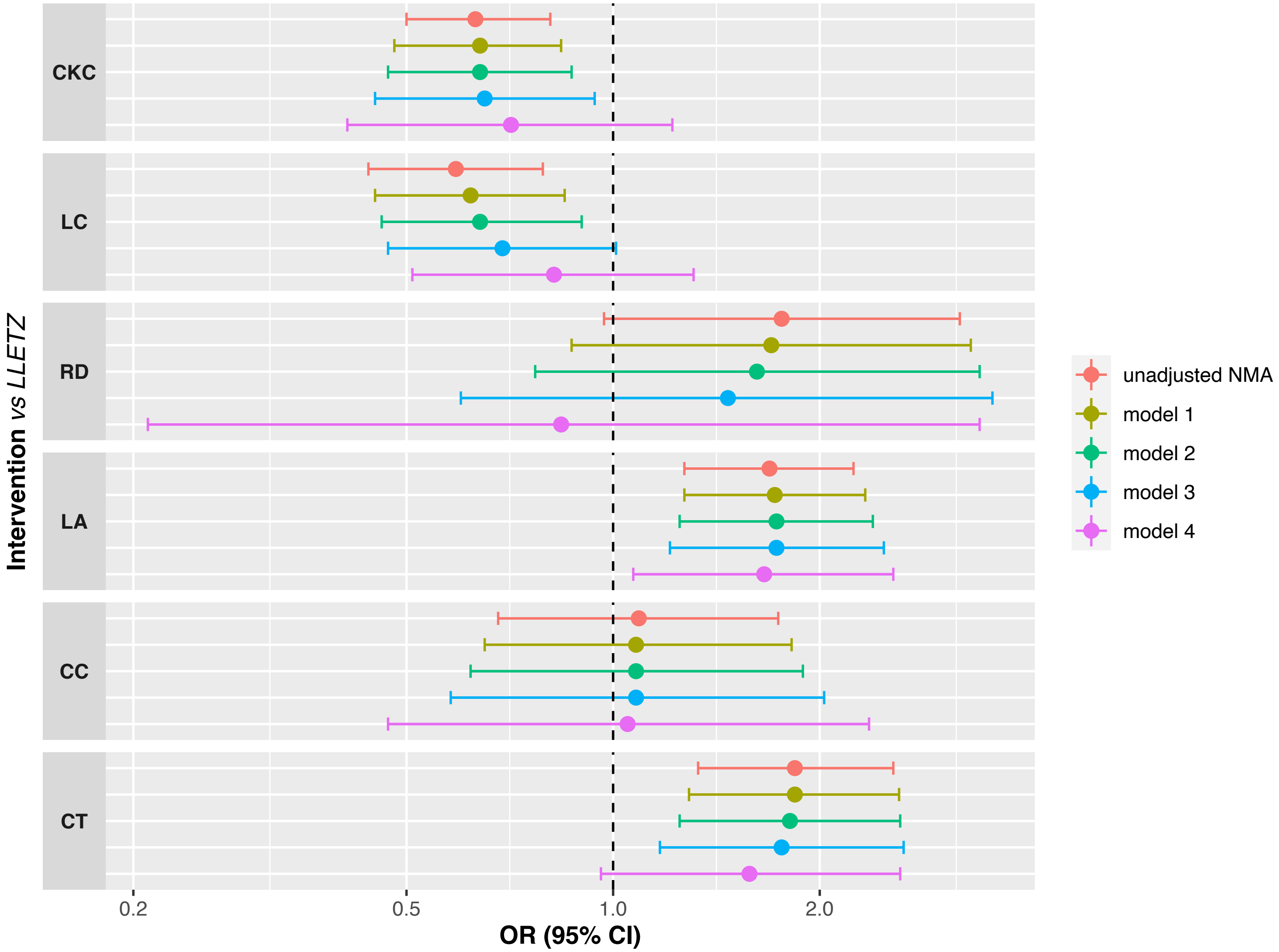

2.7.2. Preterm Birth

Table 2.7.2.1: Unadjusted NMA for risk of preterm birth after CIN treatments (main analysis) (N=29 studies)

**CKC<br>(P-score: 0·09)**	1·28<br>(0·88–1·85)<br>(0·78–2·10)	1·65<br>(1·28–2·13)<br>(1·10–2·49)	1·21<br>(0·77–1·88)<br>(0·69–2·11)	2·16<br>(1·48–3·15)<br>(1·30–3·57)	3·37<br>(0·08–146·96)<br>(0·07–171·80)	2·24<br>(0·77–6·45)<br>(0·71–7·02)	2·27<br>(1·70–3·02)<br>(1·47–3·50)
0·78<br>(0·54–1·13)<br>(0·48–1·28)	**LC<br>(P-score: 0·29)**	1·29<br>(0·97–1·72)<br>(0·84–1·99)	0·94<br>(0·59–1·5)<br>(0·53–1·68)	1·69<br>(1·15–2·46)<br>(1·02–2·79)	2·63<br>(0·06–115·14)<br>(0·05–134·62)	1·75<br>(0·60–5·07)<br>(0·55–5·52)	1·77<br>(1·29–2·43)<br>(1·12–2·79)
0·61<br>(0·47–0·78)<br>(0·40–0·91)	0·77<br>(0·58–1·03)<br>(0·50–1·20)	**LLETZ<br>(P-score: 0·51)**	0·73<br>(0·50–1·08)<br>(0·44–1·22)	1·31<br>(0·96–1·77)<br>(0·83–2·04)	2·04<br>(0·05–88·20)<br>(0·04–103·08)	1·35<br>(0·47–3·86)<br>(0·44–4·20)	1·37<br>(1·16–1·62)<br>(0·96–1·96)
0·83<br>(0·53–1·29)<br>(0·47–1·45)	1·06<br>(0·67–1·69)<br>(0·60–1·89)	1·37<br>(0·93–2·02)<br>(0·82–2·28)	**RD<br>(P-score: 0·24)**	1·79<br>(1·19–2·67)<br>(1·06–3·02)	2·79<br>(0·06–123·21)<br>(0·05–144·10)	1·85<br>(0·61–5·64)<br>(0·56–6·14)	1·88<br>(1·30–2·72)<br>(1·14–3·08)
0·46<br>(0·32–0·68)<br>(0·28–0·77)	0·59<br>(0·41–0·87)<br>(0·36–0·98)	0·77<br>(0·56–1·04)<br>(0·49–1·20)	0·56<br>(0·37–0·84)<br>(0·33–0·94)	**LA<br>(P-score: 0·74)**	1·56<br>(0·04–68·42)<br>(0·03–79·99)	1·04<br>(0·35–3·07)<br>(0·32–3·34)	1·05<br>(0·78–1·41)<br>(0·68–1·63)
0·30<br>(0·01–12·93)<br>(0·01–15·11)	0·38<br>(0·01–16·58)<br>(0·01–19·39)	0·49<br>(0·01–21·18)<br>(0·01–24·75)	0·36<br>(0·01–15·78)<br>(0·01–18·45)	0·64<br>(0·01–27·99)<br>(0·01–32·72)	**CC<br>(P-score: 0·65)**	0·66<br>(0·01–33·07)<br>(0·01–38·84)	0·67<br>(0·02–29·15)<br>(0·01–34·07)
0·45<br>(0·15–1·29)<br>(0·14–1·41)	0·57<br>(0·20–1·66)<br>(0·18–1·81)	0·74<br>(0·26–2·11)<br>(0·24–2·30)	0·54<br>(0·18–1·64)<br>(0·16–1·79)	0·96<br>(0·33–2·86)<br>(0·30–3·11)	1·51<br>(0·03–75·23)<br>(0·03–88·37)	**CT<br>(P-score: 0·68)**	1·01<br>(0·35–2·92)<br>(0·32–3·18)
0·44<br>(0·33–0·59)<br>(0·29–0·68)	0·56<br>(0·41–0·77)<br>(0·36–0·89)	0·73<br>(0·62–0·86)<br>(0·51–1·04)	0·53<br>(0·37–0·77)<br>(0·32–0·87)	0·95<br>(0·71–1·28)<br>(0·61–1·47)	1·49<br>(0·03–64·55)<br>(0·03–75·44)	0·99<br>(0·34–2·84)<br>(0·31–3·10)	**COLPO<br>(P-score: 0·79)**

Heterogeneity: τ^2=0·02; I^2=22% (0–49)

Table 2.7.2.2: Inflation variance model 1 for risk of preterm birth after CIN treatments (N=29 studies)

**CKC (P-score: 0·11)**	1·26 (0·85–1·86) (0·75–2·10)	1·65 (1·26–2·17) (1·08–2·52)	1·22 (0·76–1·95) (0·68–2·18)	2·21 (1·48–3·30) (1·31–3·72)	3·38 (0·03–437·23) (0·02–531·27)	2·12 (0·61–7·40) (0·56–8·06)	2·27 (1·68–3·08) (1·46–3·55)
0·80 (0·54–1·18) (0·48–1·33)	**LC (P-score: 0·29)**	1·32 (0·97–1·78) (0·84–2·05)	0·97 (0·59–1·58) (0·53–1·76)	1·75 (1·17–2·62) (1·04–2·96)	2·68 (0·02–348·27) (0·02–423·19)	1·68 (0·48–5·88) (0·44–6·40)	1·81 (1·30–2·51) (1·14–2·88)
0·60 (0·46–0·79) (0·40–0·92)	0·76 (0·56–1·03) (0·49–1·19)	**LLETZ (P-score: 0·52)**	0·74 (0·49–1·11) (0·43–1·25)	1·33 (0·97–1·84) (0·84–2·11)	2·04 (0·02–262·25) (0·01–318·56)	1·28 (0·37–4·41) (0·34–4·80)	1·37 (1·16–1·63) (0·96–1·97)
0·82 (0·51–1·32) (0·46–1·47)	1·03 (0·63–1·69) (0·57–1·88)	1·36 (0·90–2·05) (0·80–2·31)	**RD (P-score: 0·27)**	1·81 (1·18–2·78) (1·05–3·12)	2·77 (0·02–362·37) (0·02–440·46)	1·74 (0·47–6·37) (0·43–6·94)	1·87 (1·26–2·76) (1·12–3·12)
0·45 (0·30–0·68) (0·27–0·76)	0·57 (0·38–0·85) (0·34–0·96)	0·75 (0·54–1·03) (0·47–1·19)	0·55 (0·36–0·85) (0·32–0·95)	**LA (P-score: 0·76)**	1·53 (0·01–198·72) (0·01–241·48)	0·96 (0·27–3·42) (0·25–3·73)	1·03 (0·76–1·40) (0·66–1·61)
0·30 (0·00–38·34) (0·00–46·59)	0·37 (0·00–48·31) (0·00–58·70)	0·49 (0·00–62·97) (0·00–76·49)	0·36 (0·00–47·17) (0·00–57·33)	0·65 (0·01–84·87) (0·00–103·14)	**CC (P-score: 0·62)**	0·63 (0·00–94·09) (0·00–114·93)	0·67 (0·01–86·79) (0·00–105·44)
0·47 (0·14–1·65) (0·12–1·80)	0·59 (0·17–2·07) (0·16–2·26)	0·78 (0·23–2·70) (0·21–2·93)	0·58 (0·16–2·11) (0·14–2·30)	1·04 (0·29–3·72) (0·27–4·05)	1·60 (0·01–239·42) (0·01–292·46)	**CT (P-score: 0·64)**	1·07 (0·31–3·74) (0·28–4·07)
0·44 (0·32–0·60) (0·28–0·69)	0·55 (0·40–0·77) (0·35–0·88)	0·73 (0·61–0·86) (0·51–1·04)	0·54 (0·36–0·79) (0·32–0·89)	0·97 (0·71–1·32) (0·62–1·52)	1·48 (0·01–191·37) (0·01–232·49)	0·93 (0·27–3·24) (0·25–3·53)	**LLETZ (P-score: 0·79)**

Heterogeneity: τ^2=0·02; I^2=3% (0–29)

Table 2.7.2.3: Inflation variance model 2 for risk of preterm birth after CIN treatments (N=29 studies)

**CKC (P-score: 0·13)**	1·24<br>(0·81–1·90)<br>(0·72–2·13)	1·66<br>(1·24–2·23)<br>(1·07–2·57)	1·23<br>(0·73–2·05)<br>(0·66–2·28)	2·23<br>(1·44–3·47)<br>(1·28–3·89)	3·39<br>(0·01–1300·17)<br>(0·01–1643·54)	2·07<br>(0·48–8·99)<br>(0·44–9·82)	2·29<br>(1·64–3·18)<br>(1·44–3·64)
0·81<br>(0·53–1·23)<br>(0·47–1·39)	**LC (P-score: 0·29)**	1·34<br>(0·97–1·85)<br>(0·84–2·12)	0·99<br>(0·58–1·69)<br>(0·52–1·87)	1·80<br>(1·16–2·79)<br>(1·03–3·13)	2·73<br>(0·01–1049·87)<br>(0·01–1327·22)	1·67<br>(0·38–7·22)<br>(0·35–7·88)	1·84<br>(1·29–2·63)<br>(1·14–2·99)
0·60<br>(0·45–0·81)<br>(0·39–0·93)	0·75<br>(0·54–1·03)<br>(0·47–1·19)	**LLETZ (P-score: 0·53)**	0·74<br>(0·47–1·15)<br>(0·42–1·29)	1·34<br>(0·95–1·91)<br>(0·83–2·18)	2·04<br>(0·01–778·03)<br>(0·00–983·24)	1·25<br>(0·29–5·34)<br>(0·27–5·83)	1·38<br>(1·15–1·65)<br>(0·96–1·99)
0·82<br>(0·49–1·36)<br>(0·44–1·52)	1·01<br>(0·59–1·72)<br>(0·54–1·91)	1·35<br>(0·87–2·11)<br>(0·77–2·36)	**RD (P-score: 0·28)**	1·82<br>(1·14–2·90)<br>(1·02–3·24)	2·76<br>(0·01–1070·07)<br>(0·01–1353·13)	1·69<br>(0·37–7·69)<br>(0·34–8·40)	1·86<br>(1·22–2·84)<br>(1·09–3·20)
0·45<br>(0·29–0·70)<br>(0·26–0·78)	0·56<br>(0·36–0·86)<br>(0·32–0·97)	0·74<br>(0·52–1·06)<br>(0·46–1·20)	0·55<br>(0·34–0·88)<br>(0·31–0·98)	**LA (P-score: 0·77)**	1·52<br>(0·00–584·61)<br>(0·00–739·09)	0·93<br>(0·21–4·11)<br>(0·19–4·49)	1·03<br>(0·73–1·43)<br>(0·64–1·64)
0·30<br>(0·00–113·43)<br>(0·00–143·39)	0·37<br>(0·00–140·91)<br>(0·00–178·13)	0·49<br>(0·00–186·80)<br>(0·00–236·08)	0·36<br>(0·00–140·40)<br>(0·00–177·54)	0·66<br>(0·00–253·80)<br>(0·00–320·87)	**CC (P-score: 0·60)**	0·61<br>(0·00–277·44)<br>(0·00–352·88)	0·68<br>(0·00–258·18)<br>(0·00–326·31)
0·48<br>(0·11–2·10)<br>(0·10–2·30)	0·60<br>(0·14–2·60)<br>(0·13–2·84)	0·80<br>(0·19–3·44)<br>(0·17–3·75)	0·59<br>(0·13–2·70)<br>(0·12–2·96)	1·08<br>(0·24–4·79)<br>(0·22–5·23)	1·64<br>(0·00–744·05)<br>(0·00–946·38)	**CT (P-score: 0·61)**	1·11<br>(0·26–4·78)<br>(0·23–5·22)
0·44<br>(0·31–0·61)<br>(0·27–0·70)	0·54<br>(0·38–0·77)<br>(0·33–0·88)	0·73<br>(0·61–0·87)<br>(0·50–1·05)	0·54<br>(0·35–0·82)<br>(0·31–0·92)	0·98<br>(0·70–1·36)<br>(0·61–1·56)	1·48<br>(0·00–566·00)<br>(0·00–715·36)	0·90<br>(0·21–3·91)<br>(0·19–4·27)	**COLPO (P-score: 0·79)**

Heterogeneity: τ^2=0·02; I^2=0% (0–38)

Table 2.7.2.4: Inflation variance model 3 for risk of preterm birth after CIN treatments (N=29 studies)

**CKC (P-score: 0·15)**	1·23 (0·76–1·98) (0·68–2·21)	1·67 (1·20–2·32) (1·04–2·66)	1·24 (0·69–2·23) (0·63–2·46)	2·28 (1·38–3·76) (1·24–4·19)	3·40 (0·00–15220·72) (0·00–21074·92)	1·97 (0·30–12·84) (0·27–14·14)	2·31 (1·59–3·35) (1·41–3·80)
0·82 (0·51–1·32) (0·45–1·47)	**LC (P-score: 0·29)**	1·36 (0·95–1·95) (0·83–2·22)	1·01 (0·55–1·85) (0·50–2·04)	1·86 (1·12–3·07) (1·01–3·42)	2·78 (0·00–12438·93) (0·00–17224·00)	1·61 (0·25–10·38) (0·23–11·43)	1·89 (1·27–2·80) (1·13–3·17)
0·60 (0·43–0·84) (0·38–0·96)	0·74 (0·51–1·06) (0·45–1·20)	**LLETZ (P-score: 0·54)**	0·75 (0·45–1·23) (0·40–1·37)	1·37 (0·92–2·04) (0·81–2·30)	2·04 (0·00–9073·69) (0·00–12560·54)	1·18 (0·18–7·57) (0·17–8·33)	1·39 (1·14–1·69) (0·95–2·02)
0·81 (0·45–1·45) (0·41–1·60)	0·99 (0·54–1·80) (0·49–1·99)	1·34 (0·81–2·22) (0·73–2·47)	**RD (P-score: 0·31)**	1·83 (1·07–3·13) (0·97–3·47)	2·74 (0·00–12360·52) (0·00–17120·07)	1·59 (0·23–10·83) (0·21–11·94)	1·86 (1·16–3·00) (1·04–3·35)
0·44 (0·27–0·73) (0·24–0·81)	0·54 (0·33–0·89) (0·29–0·99)	0·73 (0·49–1·09) (0·44–1·23)	0·55 (0·32–0·93) (0·29–1·03)	**LA (P-score: 0·77)**	1·49 (0·00–6705·44) (0·00–9285·50)	0·87 (0·13–5·75) (0·12–6·34)	1·02 (0·70–1·48) (0·61–1·68)
0·29 (0·00–1315·92) (0·00–1822·05)	0·36 (0·00–1614·19) (0·00–2235·15)	0·49 (0·00–2178·59) (0·00–3015·79)	0·37 (0·00–1648·21) (0·00–2282·87)	0·67 (0·00–3004·46) (0·00–4160·50)	**CC (P-score: 0·57)**	0·58 (0·00–3154·13) (0·00–4399·42)	0·68 (0·00–3029·19) (0·00–4193·61)
0·51 (0·08–3·31) (0·07–3·64)	0·62 (0·10–4·02) (0·09–4·42)	0·85 (0·13–5·42) (0·12–5·96)	0·63 (0·09–4·30) (0·08–4·74)	1·16 (0·17–7·68) (0·16–8·46)	1·73 (0·00–9401·94) (0·00–13113·95)	**CT (P-score: 0·58)**	1·17 (0·18–7·58) (0·17–8·34)
0·43 (0·30–0·63) (0·26–0·71)	0·53 (0·36–0·79) (0·32–0·89)	0·72 (0·59–0·88) (0·50–1·05)	0·54 (0·33–0·87) (0·30–0·97)	0·98 (0·67–1·44) (0·59–1·63)	1·47 (0·00–6555·56) (0·00–9075·50)	0·85 (0·13–5·50) (0·12–6·06)	**COLPO (P-score: 0·79)**

Heterogeneity: τ^2=0·02; I^2=0% (0–38)

Table 2.7.2.5: Inflation variance model 4 for risk of preterm birth after CIN treatments (N=15 studies)

**CKC (P-score: 0·15)**	1·22 (0·67–2·23) (0·60–2·51)	1·69 (1·11–2·55) (0·97–2·92)	1·28 (0·61–2·71) (0·54–3·04)	2·40 (1·27–4·53) (1·13–5·10)	1·61 (0·07–36·11) (0·06–45·89)	2·38 (1·50–3·75) (1·32–4·28)
0·82 (0·45–1·49) (0·40–1·68)	**LC (P-score: 0·29)**	1·38 (0·88–2·16) (0·77–2·46)	1·05 (0·49–2·26) (0·43–2·54)	1·96 (1·04–3·70) (0·92–4·17)	1·31 (0·06–28·68) (0·05–36·38)	1·94 (1·19–3·15) (1·05–3·58)
0·59 (0·39–0·90) (0·34–1·03)	0·73 (0·46–1·14) (0·41–1·30)	**LLETZ (P-score: 0·55)**	0·76 (0·40–1·44) (0·35–1·63)	1·42 (0·86–2·35) (0·76–2·66)	0·95 (0·04–20·87) (0·03–26·48)	1·41 (1·13–1·76) (0·94–2·11)
0·78 (0·37–1·65) (0·33–1·85)	0·96 (0·44–2·06) (0·39–2·32)	1·32 (0·69–2·50) (0·61–2·82)	**RD (P-score: 0·33)**	1·87 (0·93–3·75) (0·83–4·22)	1·25 (0·05–29·29) (0·04–37·32)	1·85 (1·00–3·42) (0·89–3·86)
0·42 (0·22–0·79) (0·20–0·89)	0·51 (0·27–0·97) (0·24–1·09)	0·70 (0·43–1·16) (0·38–1·32)	0·53 (0·27–1·07) (0·24–1·21)	**LA (P-score: 0·83)**	0·67 (0·03–15·21) (0·02–19·35)	0·99 (0·61–1·60) (0·54–1·82)
0·62 (0·03–13·98) (0·02–17·76)	0·76 (0·03–16·65) (0·03–21·13)	1·05 (0·05–22·99) (0·04–29·16)	0·80 (0·03–18·61) (0·03–23·71)	1·49 (0·07–33·85) (0·05–43·04)	**CT (P-score: 0·51)**	1·48 (0·07–32·58) (0·05–41·34)
0·42 (0·27–0·66) (0·23–0·76)	0·52 (0·32–0·84) (0·28–0·95)	0·71 (0·57–0·89) (0·47–1·06)	0·54 (0·29–1·00) (0·26–1·12)	1·01 (0·63–1·63) (0·55–1·85)	0·68 (0·03–14·92) (0·02–18·94)	**COLPO (P-score: 0·84)**

Heterogeneity: τ^2=0·02; I^2=0% (0–48)

Summary Figure

Figure 2.7.2.1: Inflation variance models (design-adjusted analyses) for risk of preterm birth after CIN treatments

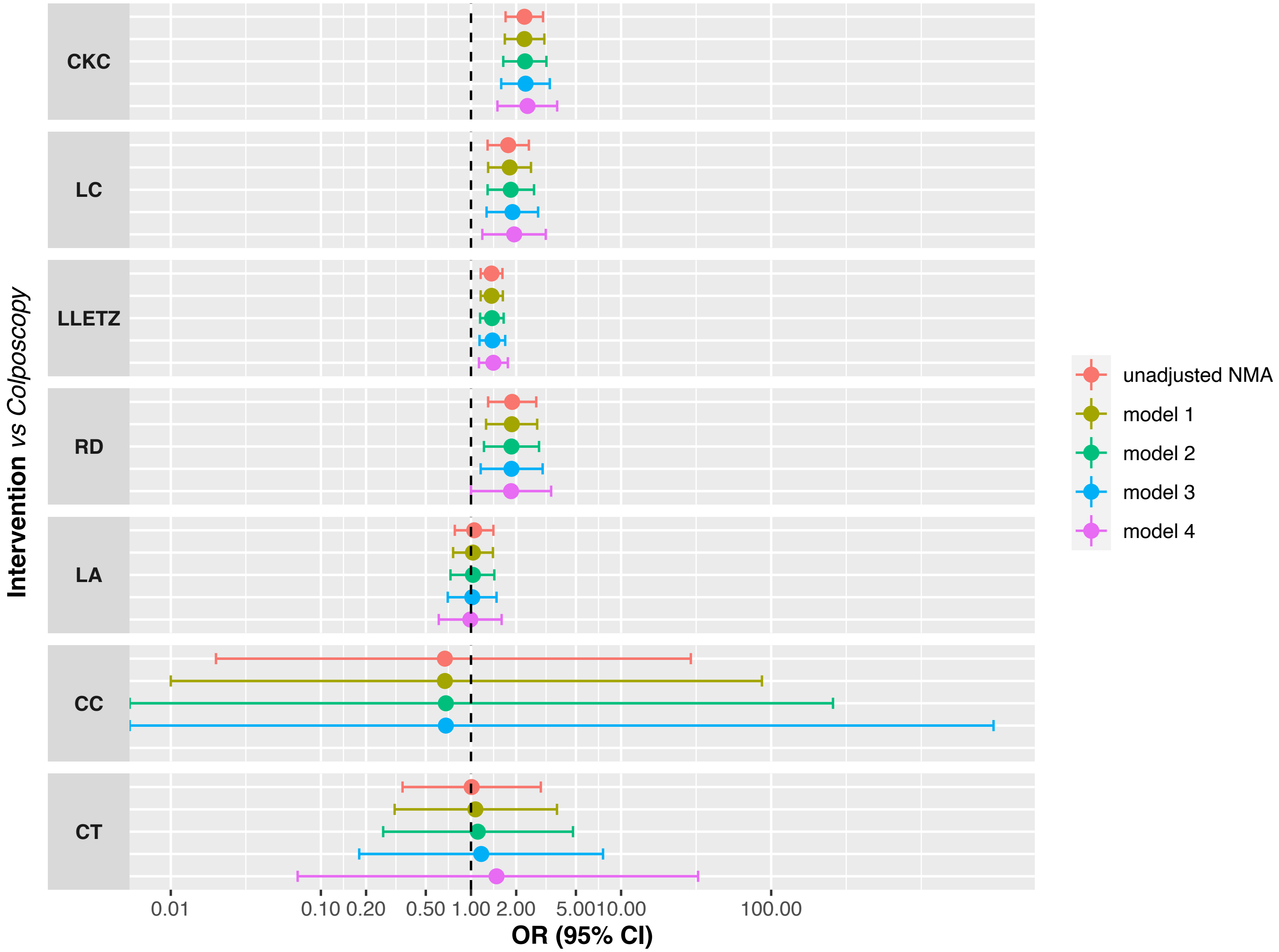

2.8. Subgroup and Sensitivity Analyses

In this section we present the figures of the distribution of all effect modifiers (publication year, age, parity [only for preterm birth], smoking, method of ascertainment of exposure or outcome, level of income of country, and percentage of women treated for CIN2+, CIN3+, AIS or cervical cancer) across treatment comparisons. After each figure we show the subgroup analyses for this effect modifier. For treatment failure we also present subgroup analyses based on the treatment indication (i.e. treatment for biopsy-proven CIN2+ or persistent CIN1; treatment only for CIN3; treatment only for AIS; treatment only for stage IA1 cervical cancer), the location of the lesion (ectocervical vs endocervical) and/or visibility of TZ (satisfactory vs unsatisfactory colposcopy), and LLETZ technique (top-hat vs standard LLETZ). Finally, we performed sensitivity analyses where we excluded all NRS and NRS at high RoB, respectively. For preterm birth we also performed a sensitivity analysis where we included only studies reporting both iatrogenic and spontaneous preterm birth.

Subgroup/sensitivity analyses are presented in league tables, where each box represents the comparison of the row-defining treatment vs the column-defining treatment. OR is reported first, followed by 95% CI and 95% PI. ORs>1 favour the column-defining treatment, while ORs<1 favour the row-defining treatment. After league tables we present the results of all subgroup/sensitivity analyses in a summary table.

2.8.1. Treatment Failure

Figure 2.8.1.1: Distribution of publication year across treatment comparisons

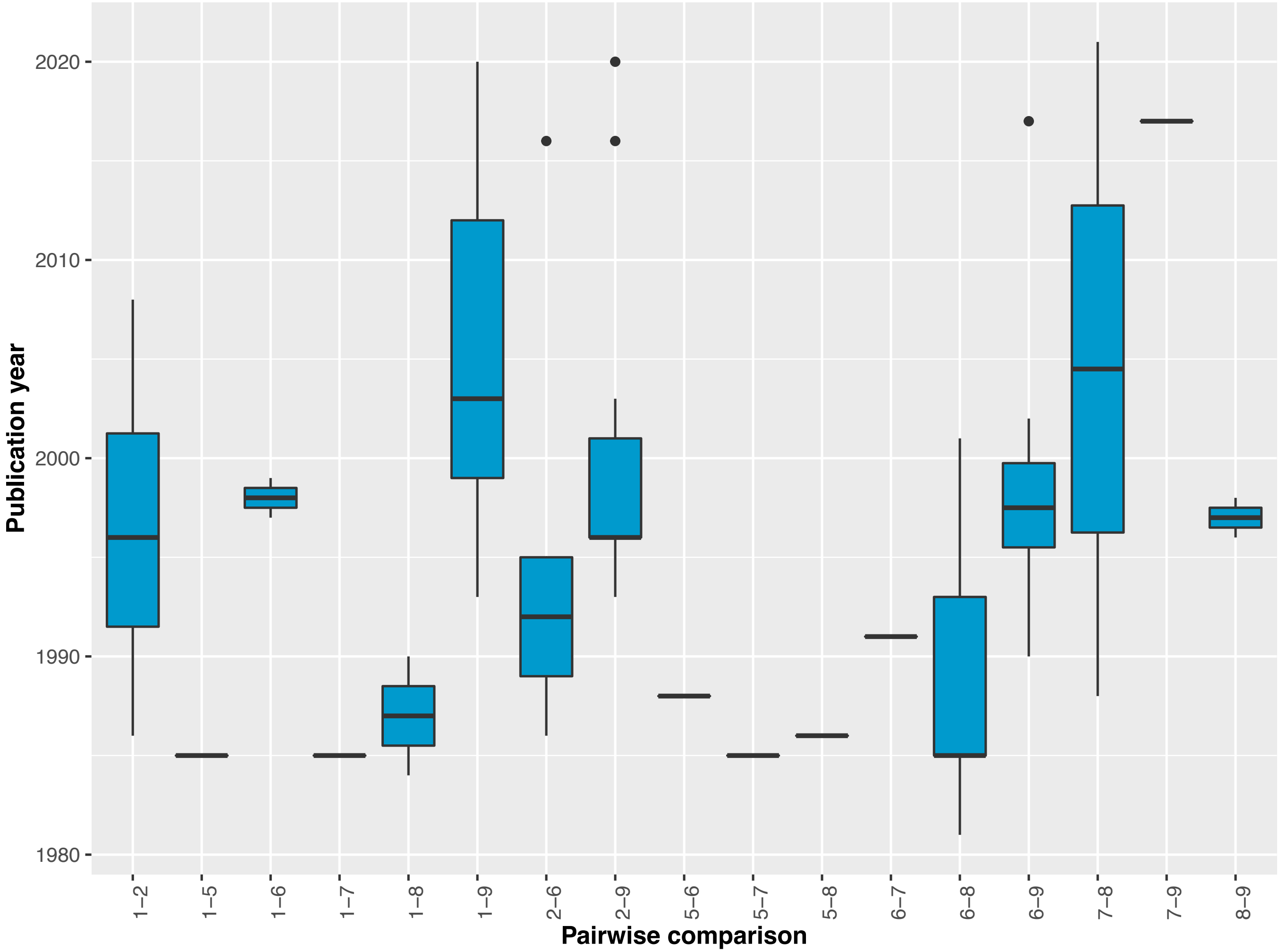

Median of the publication year across studies: 1997 (IQR=1990–2008)

1: CKC; 2: LC; 5: RD; 6: LA; 7: CC; 8: CT; 9: LLETZ

Table 2.8.1.1: Risk of treatment failure in studies published in or after 1997 (N=36 studies)

**CKC (P-score: 0·80)**	1·54 (0·91–2·60) (0·66–3·56)	0·45 (0·28–0·73) (0·20–1·02)	0·57 (0·27–1·17) (0·21–1·52)	0·38 (0·20–0·70) (0·15–0·94)	0·63 (0·48–0·84) (0·31–1·28)
0·65 (0·38–1·10) (0·28–1·51)	**LC (P-score: 0·99)**	0·30 (0·17–0·52) (0·12–0·70)	0·37 (0·16–0·83) (0·13–1·07)	0·24 (0·12–0·49) (0·09–0·65)	0·41 (0·26–0·66) (0·18–0·92)
2·20 (1·36–3·55) (0·98–4·94)	3·38 (1·93–5·94) (1·43–8·03)	**LA (P-score: 0·22)**	1·25 (0·58–2·66) (0·45–3·44)	0·83 (0·50–1·36) (0·36–1·88)	1·39 (0·94–2·07) (0·65–2·98)
1·76 (0·85–3·65) (0·66–4·74)	2·71 (1·20–6·15) (0·94–7·84)	0·80 (0·38–1·71) (0·29–2·21)	**CC (P-score: 0·40)**	0·66 (0·29–1·51) (0·23–1·93)	1·12 (0·57–2·19) (0·43–2·87)
2·66 (1·42–4·97) (1·07–6·61)	4·09 (2·02–8·26) (1·55–10·78)	1·21 (0·73–1·99) (0·53–2·75)	1·51 (0·66–3·44) (0·52–4·38)	**CT (P-score: 0·09)**	1·68 (0·96–2·96) (0·71–4·00)
1·58 (1·19–2·10) (0·78–3·19)	2·43 (1·51–3·90) (1·09–5·44)	0·72 (0·48–1·07) (0·34–1·54)	0·90 (0·46–1·75) (0·35–2·30)	0·59 (0·34–1·05) (0·25–1·41)	**LLETZ (P-score: 0·51)**

Heterogeneity: τ^2=0·10; I^2=28% (0–52)

Table 2.8.1.2: Risk of treatment failure in studies published before 1997 (N=35 studies)

**CKC (P-score: 0·92)**	0·92 (0·56–1·50) (0·41–2·08)	0·35 (0·20–0·63) (0·15–0·85)	0·33 (0·20–0·55) (0·14–0·76)	0·56 (0·28–1·13) (0·21–1·48)	0·32 (0·19–0·53) (0·14–0·73)	0·71 (0·43–1·17) (0·31–1·61)
1·09 (0·67–1·78) (0·48–2·46)	**LC (P-score: 0·86)**	0·38 (0·19–0·78) (0·14–1·02)	0·36 (0·22–0·58) (0·16–0·80)	0·61 (0·29–1·28) (0·22–1·66)	0·35 (0·21–0·58) (0·15–0·80)	0·77 (0·52–1·15) (0·36–1·65)
2·85 (1·58–5·13) (1·18–6·88)	2·62 (1·28–5·35) (0·98–6·96)	**RD (P-score: 0·22)**	0·94 (0·47–1·87) (0·36–2·44)	1·60 (0·73–3·49) (0·57–4·48)	0·91 (0·46–1·80) (0·35–2·36)	2·02 (0·99–4·12) (0·76–5·36)
3·03 (1·81–5·07) (1·32–6·96)	2·79 (1·73–4·49) (1·24–6·24)	1·07 (0·54–2·12) (0·41–2·77)	**LA (P-score: 0·18)**	1·70 (0·89–3·23) (0·68–4·28)	0·97 (0·71–1·33) (0·48–1·98)	2·15 (1·37–3·37) (0·98–4·74)
1·78 (0·88–3·61) (0·68–4·70)	1·64 (0·78–3·44) (0·60–4·45)	0·63 (0·29–1·37) (0·22–1·76)	0·59 (0·31–1·12) (0·23–1·48)	**CC (P-score: 0·53)**	0·57 (0·30–1·08) (0·23–1·43)	1·27 (0·61–2·62) (0·47–3·40)
3·12 (1·88–5·18) (1·37–7·12)	2·86 (1·72–4·78) (1·25–6·56)	1·10 (0·56–2·15) (0·42–2·83)	1·03 (0·75–1·40) (0·50–2·10)	1·75 (0·92–3·31) (0·70–4·38)	**CT (P-score: 0·15)**	2·21 (1·37–3·57) (0·99–4·96)
1·41 (0·86–2·31) (0·62–3·19)	1·29 (0·87–1·93) (0·61–2·76)	0·49 (0·24–1·01) (0·19–1·31)	0·46 (0·30–0·73) (0·21–1·02)	0·79 (0·38–1·64) (0·29–2·12)	0·45 (0·28–0·73) (0·20–1·01)	**LLETZ (P-score: 0·65)**

Heterogeneity: τ^2=0·10; I^2=28% (0–53)

Figure 2.8.1.2: Distribution of age across treatment comparisons

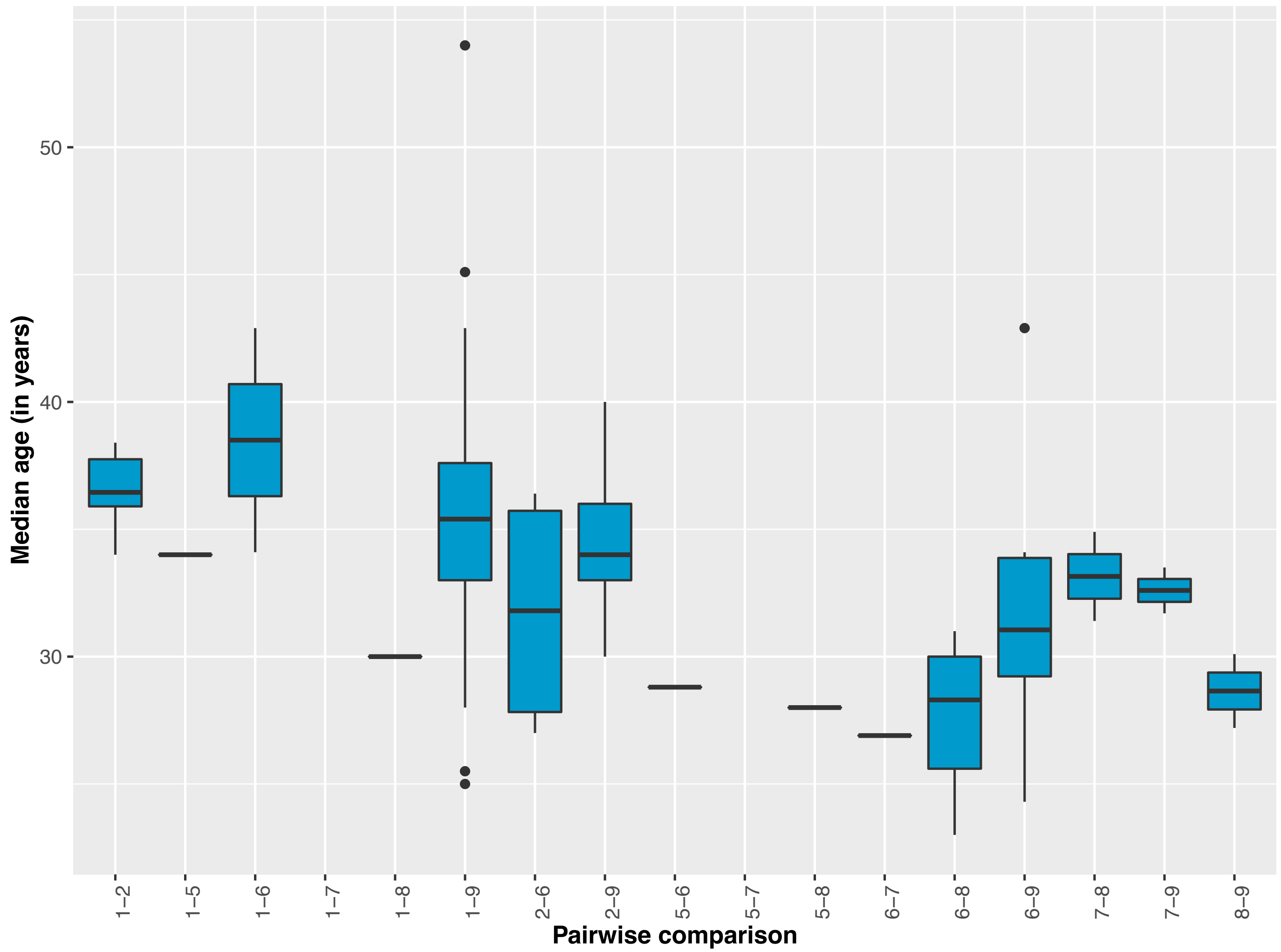

Median of the median age across studies: 33y (IQR=30–36); mean was used if median not reported; neither median nor mean reported in 11 studies

1: CKC; 2: LC; 5: RD; 6: LA; 7: CC; 8: CT; 9: LLETZ

Table 2.8.1.3: Risk of treatment failure in studies with median age ≥33 years (N=33 studies)

**CKC (P-score: 0·76)**	1·50 (1·00–2·27) (0·71–3·19)	0·35 (0·15–0·78) (0·12–0·99)	0·52 (0·27–1·00) (0·20–1·31)	0·77 (0·32–1·81) (0·26–2·28)	0·49 (0·13–1·84) (0·11–2·21)	0·61 (0·45–0·81) (0·30–1·21)
0·66 (0·44–1·00) (0·31–1·41)	**LC (P-score: 0·98)**	0·23 (0·09–0·57) (0·07–0·72)	0·34 (0·18–0·65) (0·14–0·85)	0·51 (0·21–1·25) (0·17–1·56)	0·33 (0·08–1·25) (0·07–1·50)	0·40 (0·27–0·59) (0·19–0·84)
2·87 (1·28–6·46) (1·01–8·17)	4·32 (1·74–10·72) (1·40–13·37)	**RD (P-score: 0·13)**	1·49 (0·52–4·24) (0·42–5·20)	2·20 (0·67–7·18) (0·56–8·73)	1·41 (0·30–6·63) (0·25–7·92)	1·74 (0·73–4·12) (0·59–5·17)
1·93 (1·00–3·75) (0·77–4·88)	2·91 (1·53–5·53) (1·17–7·23)	0·67 (0·24–1·92) (0·19–2·35)	**LA (P-score: 0·31)**	1·48 (0·53–4·13) (0·43–5·08)	0·95 (0·23–3·97) (0·19–4·76)	1·17 (0·62–2·19) (0·48–2·88)
1·31 (0·55–3·09) (0·44–3·88)	1·96 (0·80–4·83) (0·64–6·03)	0·45 (0·14–1·48) (0·11–1·80)	0·68 (0·24–1·88) (0·20–2·32)	**CC (P-score: 0·59)**	0·64 (0·23–1·74) (0·19–2·15)	0·79 (0·35–1·78) (0·28–2·25)
2·04 (0·54–7·68) (0·45–9·24)	3·08 (0·80–11·83) (0·66–14·23)	0·71 (0·15–3·36) (0·13–4·01)	1·06 (0·25–4·44) (0·21–5·32)	1·57 (0·57–4·27) (0·47–5·27)	**CT (P-score: 0·32)**	1·24 (0·34–4·49) (0·28–5·42)
1·65 (1·23–2·22) (0·83–3·30)	2·49 (1·68–3·67) (1·19–5·21)	0·58 (0·24–1·36) (0·19–1·71)	0·86 (0·46–1·60) (0·35–2·10)	1·27 (0·56–2·85) (0·45–3·60)	0·81 (0·22–2·94) (0·18–3·54)	**LLETZ (P-score: 0·42)**

Heterogeneity: τ^2=0·09; I^2=21% (0–49)

Table 2.8.1.4: Risk of treatment failure in studies with median age <33 years (N=27 studies)

**CKC**<br>**(P-score: 0·94)**	0·41<br>(0·19–0·89)<br>(0·18–0·93)	0·62<br>(0·16–2·49)<br>(0·14–2·70)	0·30<br>(0·16–0·57)<br>(0·16–0·59)	0·33<br>(0·14–0·82)<br>(0·13–0·86)	0·31<br>(0·17–0·60)<br>(0·16–0·62)	0·63<br>(0·35–1·13)<br>(0·34–1·17)
2·46<br>(1·13–5·34)<br>(1·08–5·59)	**LC**<br>**(P-score: 0·45)**	1·53<br>(0·40–5·90)<br>(0·37–6·38)	0·75<br>(0·44–1·27)<br>(0·43–1·31)	0·82<br>(0·36–1·89)<br>(0·34–1·99)	0·77<br>(0·44–1·35)<br>(0·43–1·39)	1·55<br>(0·93–2·59)<br>(0·90–2·67)
1·61<br>(0·40–6·44)<br>(0·37–6·98)	0·65<br>(0·17–2·52)<br>(0·16–2·73)	**RD**<br>**(P-score: 0·67)**	0·49<br>(0·14–1·70)<br>(0·13–1·82)	0·54<br>(0·13–2·19)<br>(0·12–2·38)	0·51<br>(0·15–1·74)<br>(0·14–1·87)	1·01<br>(0·29–3·59)<br>(0·27–3·86)
3·29<br>(1·76–6·17)<br>(1·69–6·40)	1·34<br>(0·79–2·28)<br>(0·76–2·35)	2·05<br>(0·59–7·12)<br>(0·55–7·65)	**LA**<br>**(P-score: 0·17)**	1·10<br>(0·57–2·13)<br>(0·55–2·21)	1·04<br>(0·85–1·26)<br>(0·84–1·27)	2·08<br>(1·62–2·66)<br>(1·60–2·70)
2·99<br>(1·22–7·32)<br>(1·16–7·71)	1·22<br>(0·53–2·81)<br>(0·50–2·95)	1·86<br>(0·46–7·60)<br>(0·42–8·24)	0·91<br>(0·47–1·76)<br>(0·45–1·83)	**CC**<br>**(P-score: 0·29)**	0·94<br>(0·48–1·85)<br>(0·46–1·93)	1·89<br>(0·95–3·73)<br>(0·92–3·88)
3·18<br>(1·68–6·03)<br>(1·62–6·25)	1·29<br>(0·74–2·25)<br>(0·72–2·33)	1·98<br>(0·57–6·82)<br>(0·53–7·33)	0·97<br>(0·80–1·17)<br>(0·79–1·19)	1·06<br>(0·54–2·09)<br>(0·52–2·18)	**CT**<br>**(P-score: 0·23)**	2·00<br>(1·51–2·66)<br>(1·49–2·70)
1·59<br>(0·88–2·84)<br>(0·86–2·94)	0·65<br>(0·39–1·08)<br>(0·37–1·11)	0·99<br>(0·28–3·49)<br>(0·26–3·76)	0·48<br>(0·38–0·62)<br>(0·37–0·63)	0·53<br>(0·27–1·05)<br>(0·26–1·09)	0·50<br>(0·38–0·66)<br>(0·37–0·67)	**LLETZ**<br>**(P-score: 0·75)**

Heterogeneity: τ^2=0·00; I^2=0% (0–45)

Figure 2.8.1.3: Distribution of smoking across treatment comparisons

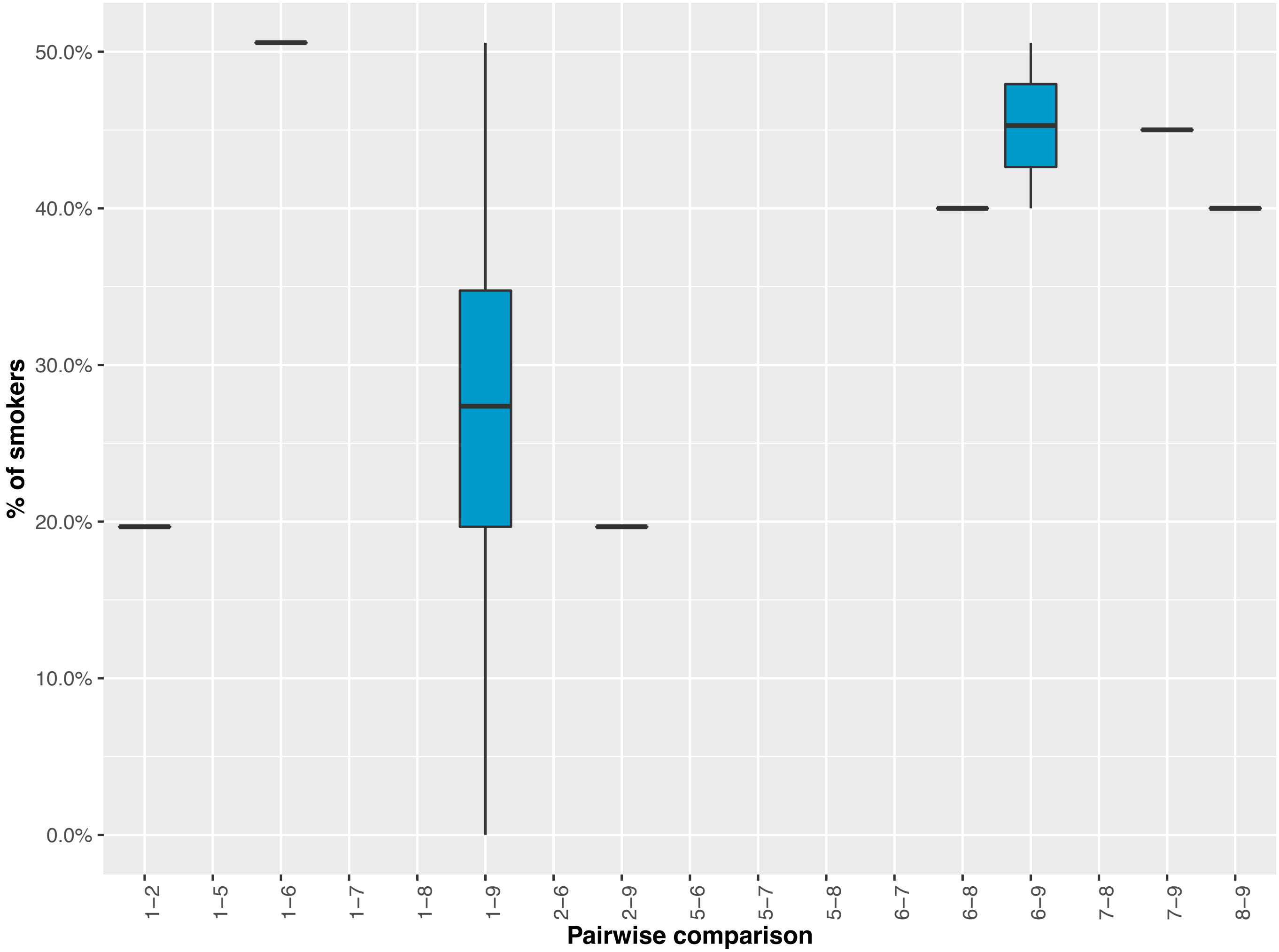

Median of the percentage of smokers across studies: 35% (IQR=24–43); percentage of smokers not reported in 64 studies

1: CKC; 2: LC; 5: RD; 6: LA; 7: CC; 8: CT; 9: LLETZ

Table 2.8.1.5: Risk of treatment failure in studies where ≥35% of women smoked (N=3 studies)

**CKC**<br>**(P-score: 0·73)**	0·82<br>(0·19–3·58)<br>(NA, NA)	0·20<br>(0·03–1·34)<br>(NA, NA)	0·55<br>(0·12–2·44)<br>(NA, NA)	0·88<br>(0·22–3·56)<br>(NA, NA)
1·22<br>(0·28–5·35)<br>(NA, NA)	**LA**<br>**(P-score: 0·67)**	0·24<br>(0·06–1·03)<br>(NA, NA)	0·67<br>(0·37–1·22)<br>(NA, NA)	1·08<br>(0·58–2·00)<br>(NA, NA)
5·11<br>(0·75–34·90)<br>(NA, NA)	4·18<br>(0·97–17·94)<br>(NA, NA)	**CC**<br>**(P-score: 0·04)**	2·80<br>(0·66–11·93)<br>(NA, NA)	4·50<br>(1·20–16·85)<br>(NA, NA)
1·82<br>(0·41–8·12)<br>(NA, NA)	1·49<br>(0·82–2·71)<br>(NA, NA)	0·36<br>(0·08–1·52)<br>(NA, NA)	**CT**<br>**(P-score: 0·32)**	1·61<br>(0·88–2·92)<br>(NA, NA)
1·13<br>(0·28–4·59)<br>(NA, NA)	0·93<br>(0·50–1·72)<br>(NA, NA)	0·22<br>(0·06–0·83)<br>(NA, NA)	0·62<br>(0·34–1·13)<br>(NA, NA)	**LLETZ**<br>**(P-score: 0·74)**

Heterogeneity: τ^2=0·00; I^2=0% (95% CI NA)

Table 2.8.1.6: Risk of treatment failure in studies where <35% smoked (N=4 studies)

**CKC (P-score: 0·68)**	1·54 (0·05–45·01) (0·00–3424·93)	0·48 (0·21–1·09) (0·03–7·82)
0·65 (0·02–18·86) (0·00–1435·22)	**LC (P-score: 0·67)**	0·31 (0·01–9·52) (0·00–764·37)
2·08 (0·92–4·72) (0·13–33·86)	3·22 (0·11–98·37) (0·00–7902·32)	**LLETZ (P-score: 0·15)**

Heterogeneity: τ^2=0·25; I^2=35% (0–77)

Figure 2.8.1.4: Ascertainment of exposure across treatment comparisons

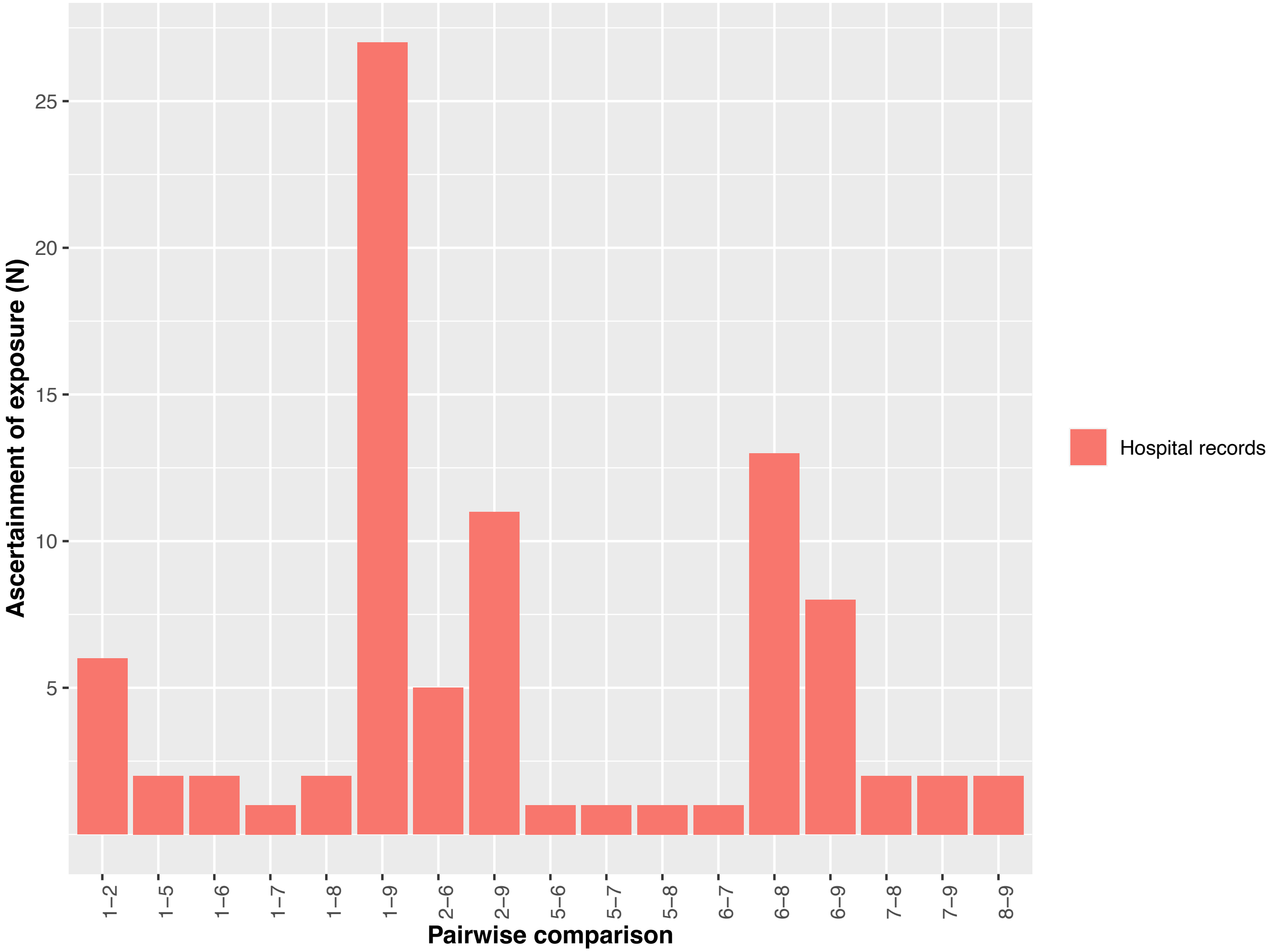

1: CKC; 2: LC; 5: RD; 6: LA; 7: CC; 8: CT; 9: LLETZ

Figure 2.8.1.5: Ascertainment of outcome across treatment comparisons

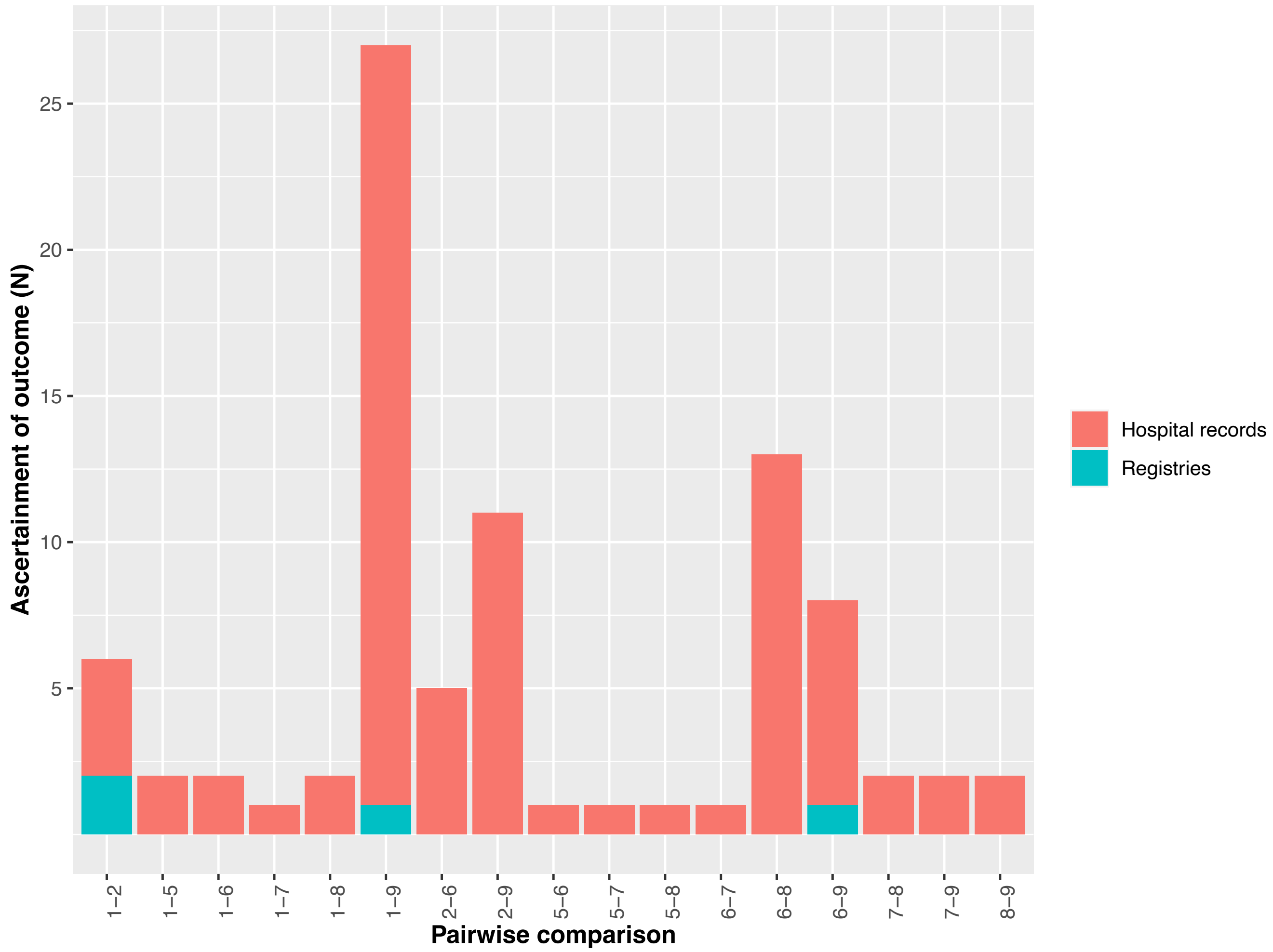

1: CKC; 2: LC; 5: RD; 6: LA; 7: CC; 8: CT; 9: LLETZ

Table 2.8.1.7: Risk of treatment failure in studies where ascertainment of outcome was through hospital records (N=67 studies)

**CKC (P-score: 0·93)**	0·97 (0·65–1·44) (0·44–2·12)	0·35 (0·20–0·64) (0·14–0·87)	0·36 (0·24–0·52) (0·16–0·77)	0·55 (0·33–0·93) (0·23–1·30)	0·33 (0·22–0·49) (0·15–0·72)	0·60 (0·46–0·78) (0·29–1·23)
1·03 (0·69–1·54) (0·47–2·27)	**LC (P-score: 0·90)**	0·37 (0·18–0·72) (0·14–0·96)	0·37 (0·25–0·54) (0·17–0·80)	0·57 (0·33–1·01) (0·24–1·39)	0·34 (0·22–0·52) (0·15–0·75)	0·62 (0·45–0·85) (0·29–1·31)
2·83 (1·56–5·12) (1·15–6·99)	2·74 (1·38–5·41) (1·04–7·19)	**RD (P-score: 0·21)**	1·01 (0·53–1·93) (0·39–2·59)	1·57 (0·77–3·18) (0·58–4·20)	0·92 (0·48–1·78) (0·36–2·38)	1·69 (0·91–3·14) (0·67–4·25)
2·81 (1·92–4·10) (1·29–6·10)	2·72 (1·84–4·01) (1·24–5·93)	0·99 (0·52–1·90) (0·39–2·55)	**LA (P-score: 0·21)**	1·56 (0·94–2·58) (0·67–3·63)	0·92 (0·70–1·20) (0·44–1·90)	1·68 (1·23–2·30) (0·80–3·54)
1·81 (1·07–3·05) (0·77–4·26)	1·75 (0·99–3·07) (0·72–4·23)	0·64 (0·31–1·30) (0·24–1·71)	0·64 (0·39–1·06) (0·28–1·50)	**CC (P-score: 0·54)**	0·59 (0·35–0·98) (0·25–1·38)	1·08 (0·66–1·76) (0·47–2·49)
3·06 (2·06–4·56) (1·40–6·72)	2·96 (1·92–4·57) (1·33–6·62)	1·08 (0·56–2·08) (0·42–2·79)	1·09 (0·83–1·43) (0·53–2·26)	1·70 (1·02–2·82) (0·73–3·97)	**CT (P-score: 0·12)**	1·83 (1·29–2·60) (0·86–3·92)
1·67 (1·28–2·18) (0·81–3·45)	1·62 (1·17–2·24) (0·76–3·42)	0·59 (0·32–1·10) (0·24–1·49)	0·60 (0·44–0·81) (0·28–1·25)	0·93 (0·57–1·51) (0·40–2·14)	0·55 (0·38–0·78) (0·26–1·17)	**LLETZ (P-score: 0·60)**

Heterogeneity: τ^2=0·11; I^2=32% (9–50)

Table 2.8.1.8: Risk of treatment failure in studies where ascertainment of outcome was through population-based registries (N=4 studies)

**CKC (P-score: 0·58)**	1·37 (0·81–2·32) (NA, NA)	0·49 (0·21–1·16) (NA, NA)	0·85 (0·43–1·66) (NA, NA)
0·73 (0·43–1·23) (NA, NA)	**LC (P-score: 0·91)**	0·36 (0·13–0·98) (NA, NA)	0·62 (0·26–1·45) (NA, NA)
2·02 (0·86–4·75) (NA, NA)	2·78 (1·02–7·57) (NA, NA)	**LA (P-score: 0·03)**	1·72 (1·01–2·90) (NA, NA)
1·18 (0·60–2·31) (NA, NA)	1·62 (0·69–3·80) (NA, NA)	0·58 (0·34–0·99) (NA, NA)	**LLETZ (P-score: 0·48)**

Heterogeneity: τ^2=0·00; I^2=0% (95% CI NA)

Figure 2.8.1.6: Level of income of country across treatment comparisons

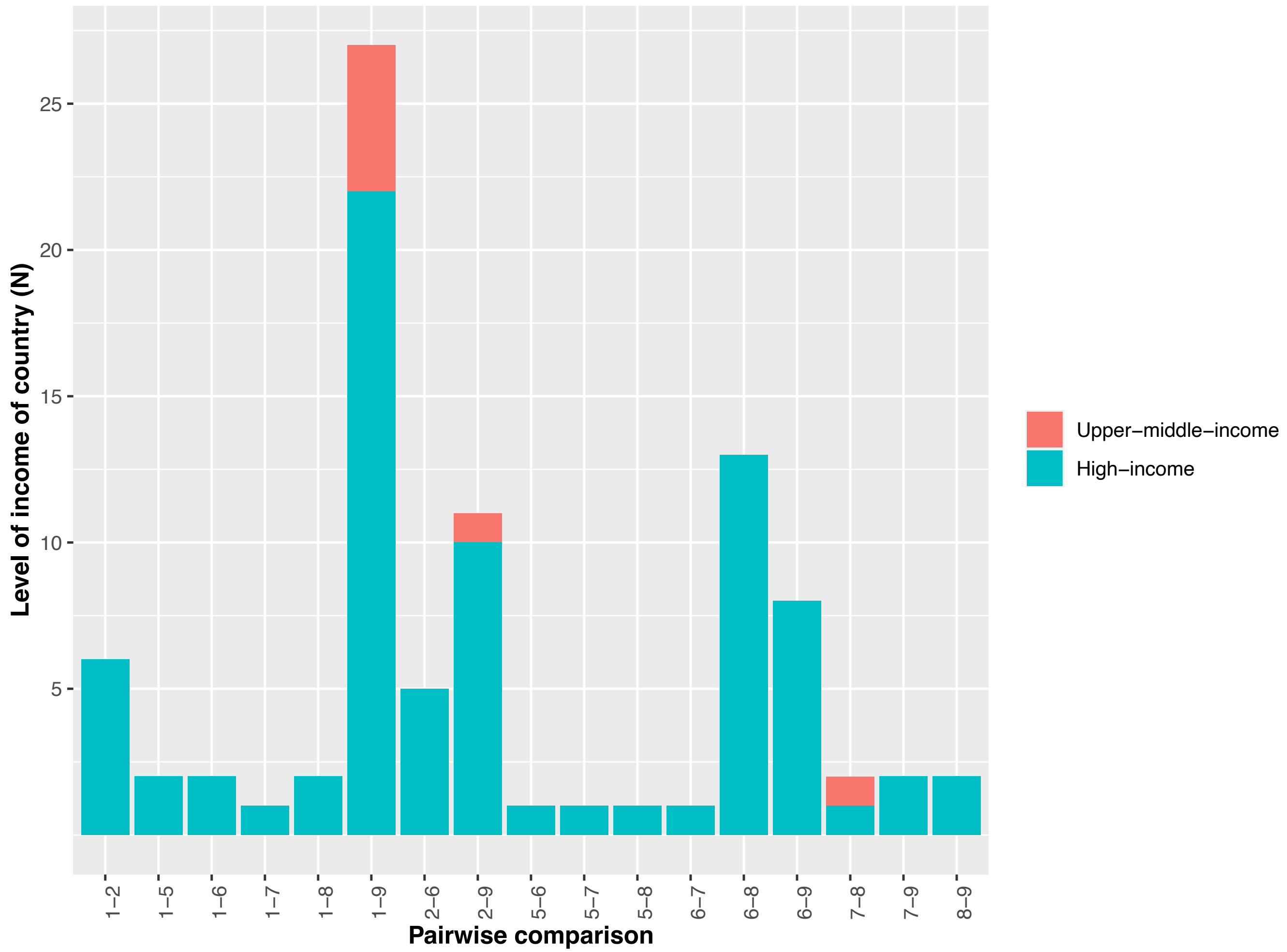

1: CKC; 2: LC; 5: RD; 6: LA; 7: CC; 8: CT; 9: LLETZ

Table 2.8.1.9: Risk of treatment failure in studies conducted in middle-income countries (N=7 studies)

**CKC**<br>**(P-score: 0·90)**	0·43<br>(0·06–3·14)<br>(0·01–21·14)	0·31<br>(0·13–0·74)<br>(0·02–4·19)
2·30<br>(0·32–16·65)<br>(0·05–112·07)	**LC**<br>**(P-score: 0·42)**	0·72<br>(0·12–4·31)<br>(0·02–27·30)
3·18<br>(1·35–7·47)<br>(0·24–42·31)	1·38<br>(0·23–8·22)<br>(0·04–52·01)	**LLETZ**<br>**(P-score: 0·18)**

Heterogeneity: τ^2=0·47; I^2=54% (0–83)

Table 2.8.1.10: Risk of treatment failure in studies conducted in high-income countries (N=64 studies)

**CKC (P-score: 0·86)**	1·17<br>(0·83–1·64)<br>(0·59–2·29)	0·37<br>(0·21–0·64)<br>(0·16–0·83)	0·41<br>(0·29–0·58)<br>(0·21–0·80)	0·62<br>(0·37–1·03)<br>(0·29–1·34)	0·37<br>(0·26–0·54)<br>(0·19–0·75)	0·70<br>(0·54–0·91)<br>(0·37–1·32)
0·86<br>(0·61–1·21)<br>(0·44–1·69)	**LC (P-score: 0·97)**	0·32<br>(0·17–0·59)<br>(0·13–0·75)	0·35<br>(0·25–0·50)<br>(0·18–0·69)	0·53<br>(0·31–0·91)<br>(0·24–1·18)	0·32<br>(0·22–0·48)<br>(0·16–0·65)	0·60<br>(0·45–0·81)<br>(0·31–1·15)
2·72<br>(1·56–4·76)<br>(1·21–6·13)	3·17<br>(1·69–5·96)<br>(1·34–7·53)	**RD (P-score: 0·15)**	1·11<br>(0·60–2·05)<br>(0·48–2·60)	1·69<br>(0·86–3·32)<br>(0·69–4·15)	1·02<br>(0·55–1·89)<br>(0·43–2·40)	1·91<br>(1·06–3·43)<br>(0·83–4·39)
2·45<br>(1·74–3·46)<br>(1·25–4·82)	2·85<br>(2·01–4·06)<br>(1·45–5·64)	0·90<br>(0·49–1·66)<br>(0·38–2·10)	**LA (P-score: 0·24)**	1·52<br>(0·93–2·47)<br>(0·71–3·25)	0·92<br>(0·71–1·18)<br>(0·49–1·73)	1·72<br>(1·30–2·27)<br>(0·90–3·27)
1·61<br>(0·98–2·67)<br>(0·74–3·50)	1·88<br>(1·10–3·21)<br>(0·85–4·16)	0·59<br>(0·30–1·17)<br>(0·24–1·46)	0·66<br>(0·41–1·07)<br>(0·31–1·41)	**CC (P-score: 0·53)**	0·60<br>(0·37–1·00)<br>(0·28–1·31)	1·13<br>(0·71–1·81)<br>(0·53–2·40)
2·67<br>(1·84–3·87)<br>(1·34–5·33)	3·11<br>(2·09–4·63)<br>(1·54–6·30)	0·98<br>(0·53–1·82)<br>(0·42–2·30)	1·09<br>(0·85–1·40)<br>(0·58–2·05)	1·65<br>(1·00–2·73)<br>(0·77–3·57)	**CT (P-score: 0·13)**	1·87<br>(1·36–2·59)<br>(0·96–3·64)
1·43<br>(1·10–1·84)<br>(0·75–2·69)	1·66<br>(1·24–2·23)<br>(0·87–3·18)	0·52<br>(0·29–0·94)<br>(0·23–1·20)	0·58<br>(0·44–0·77)<br>(0·31–1·11)	0·88<br>(0·55–1·41)<br>(0·42–1·87)	0·53<br>(0·39–0·74)<br>(0·27–1·04)	**LLETZ (P-score: 0·61)**

Heterogeneity: τ^2=0·08; I^2=28% (2–47)

Figure 2.8.1.7: Distribution of CIN2+ across treatment comparisons

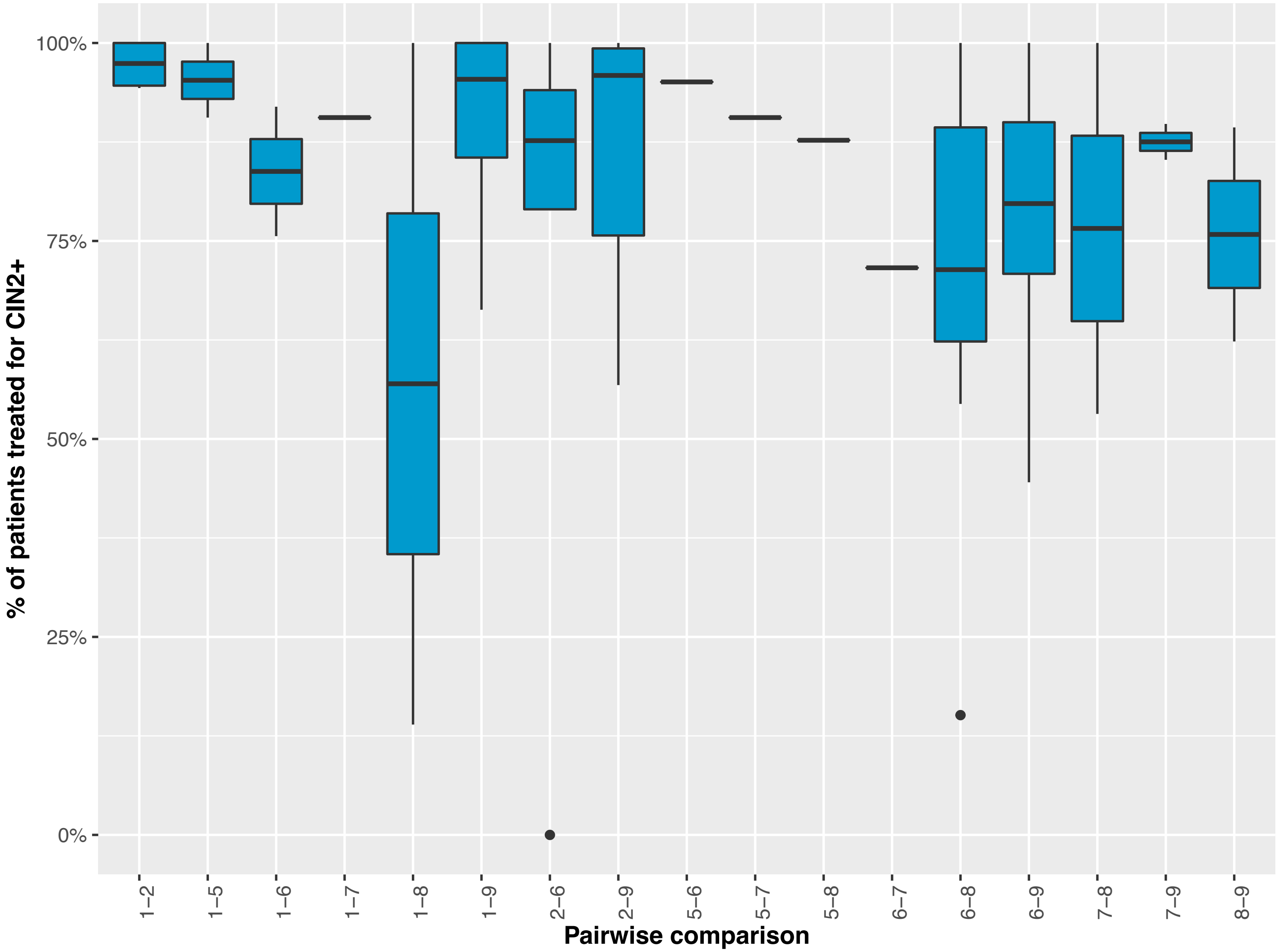

Median of the percentage of women treated for CIN2+ across studies: 89% (IQR=72–100); percentage of women treated for CIN2+ not reported in 4 studies

1: CKC; 2: LC; 5: RD; 6: LA; 7: CC; 8: CT; 9: LLETZ

Table 2.8.1.11: Risk of treatment failure in studies where ≥89% of women had been treated for CIN2+ (N=35 studies)

**CKC (P-score: 0·83)**	1·43 (0·94–2·19) (0·68–3·02)	0·29 (0·16–0·53) (0·12–0·69)	0·32 (0·18–0·55) (0·14–0·72)	0·48 (0·21–1·07) (0·17–1·34)	0·32 (0·18–0·59) (0·13–0·77)	0·64 (0·46–0·89) (0·32–1·29)
0·70 (0·46–1·06) (0·33–1·47)	**LC (P-score: 0·99)**	0·20 (0·10–0·42) (0·08–0·53)	0·22 (0·13–0·38) (0·10–0·51)	0·33 (0·14–0·80) (0·11–0·99)	0·23 (0·12–0·42) (0·09–0·55)	0·45 (0·30–0·67) (0·22–0·94)
3·48 (1·89–6·40) (1·45–8·33)	4·98 (2·41–10·31) (1·90–13·09)	**RD (P-score: 0·16)**	1·10 (0·50–2·40) (0·40–3·02)	1·66 (0·69–3·98) (0·56–4·93)	1·12 (0·49–2·56) (0·39–3·20)	2·24 (1·14–4·38) (0·89–5·62)
3·16 (1·83–5·48) (1·38–7·25)	4·54 (2·60–7·90) (1·97–10·44)	0·91 (0·42–1·99) (0·33–2·50)	**LA (P-score: 0·21)**	1·51 (0·62–3·69) (0·50–4·56)	1·02 (0·66–1·60) (0·48–2·18)	2·04 (1·25–3·33) (0·92–4·48)
2·10 (0·94–4·70) (0·75–5·88)	3·01 (1·26–7·19) (1·01–8·91)	0·60 (0·25–1·45) (0·20–1·79)	0·66 (0·27–1·62) (0·22–2·00)	**CC (P-score: 0·46)**	0·68 (0·27–1·67) (0·22–2·07)	1·35 (0·60–3·04) (0·48–3·80)
3·10 (1·68–5·69) (1·29–7·42)	4·44 (2·36–8·35) (1·82–10·82)	0·89 (0·39–2·03) (0·31–2·53)	0·98 (0·63–1·53) (0·46–2·09)	1·48 (0·60–3·65) (0·48–4·50)	**CT (P-score: 0·23)**	1·99 (1·14–3·48) (0·86–4·60)
1·55 (1·12–2·16) (0·78–3·11)	2·23 (1·49–3·34) (1·07–4·65)	0·45 (0·23–0·88) (0·18–1·12)	0·49 (0·30–0·80) (0·22–1·08)	0·74 (0·33–1·67) (0·26–2·09)	0·50 (0·29–0·88) (0·22–1·16)	**LLETZ (P-score: 0·63)**

Heterogeneity: τ^2=0·09; I^2=22% (0–48)

Table 2.8.1.12: Risk of treatment failure in studies where <89% of women had been treated for CIN2+ (N=32 studies)

**CKC (P-score: 0·85)**	0·76 (0·43–1·34) (0·35–1·65)	1·31 (0·27–6·41) (0·23–7·43)	0·42 (0·26–0·68) (0·20–0·85)	0·67 (0·35–1·27) (0·29–1·55)	0·38 (0·23–0·63) (0·18–0·79)	0·64 (0·42–0·97) (0·33–1·24)
1·31 (0·75–2·30) (0·61–2·85)	**LC (P-score: 0·65)**	1·71 (0·35–8·43) (0·30–9·78)	0·55 (0·35–0·86) (0·27–1·09)	0·87 (0·46–1·65) (0·38–2·02)	0·50 (0·30–0·83) (0·24–1·04)	0·84 (0·57–1·24) (0·44–1·60)
0·77 (0·16–3·76) (0·13–4·36)	0·58 (0·12–2·87) (0·10–3·33)	**RD (P-score: 0·81)**	0·32 (0·07–1·49) (0·06–1·73)	0·51 (0·10–2·54) (0·09–2·95)	0·29 (0·06–1·32) (0·06–1·53)	0·49 (0·10–2·32) (0·09–2·69)
2·40 (1·48–3·89) (1·17–4·90)	1·83 (1·16–2·87) (0·92–3·64)	3·13 (0·67–14·58) (0·58–16·91)	**LA (P-score: 0·14)**	1·60 (0·94–2·71) (0·76–3·38)	0·91 (0·68–1·22) (0·51–1·64)	1·53 (1·11–2·11) (0·84–2·80)
1·50 (0·78–2·87) (0·64–3·49)	1·14 (0·60–2·16) (0·50–2·63)	1·96 (0·39–9·76) (0·34–11·33)	0·63 (0·37–1·06) (0·30–1·32)	**CC (P-score: 0·53)**	0·57 (0·33–0·99) (0·27–1·22)	0·96 (0·57–1·62) (0·46–2·02)
2·63 (1·60–4·32) (1·27–5·42)	2·00 (1·21–3·32) (0·96–4·15)	3·43 (0·76–15·52) (0·65–18·01)	1·10 (0·82–1·47) (0·61–1·97)	1·75 (1·01–3·02) (0·82–3·75)	**CT (P-score: 0·06)**	1·68 (1·15–2·45) (0·89–3·18)
1·57 (1·03–2·37) (0·80–3·04)	1·19 (0·81–1·76) (0·62–2·28)	2·04 (0·43–9·69) (0·37–11·25)	0·65 (0·47–0·90) (0·36–1·19)	1·04 (0·62–1·76) (0·50–2·20)	0·60 (0·41–0·87) (0·31–1·13)	**LLETZ (P-score: 0·47)**

Heterogeneity: τ^2=0·06; I^2=25% (0–53)

Figure 2.8.1.8: Distribution of CIN3+ across treatment comparisons

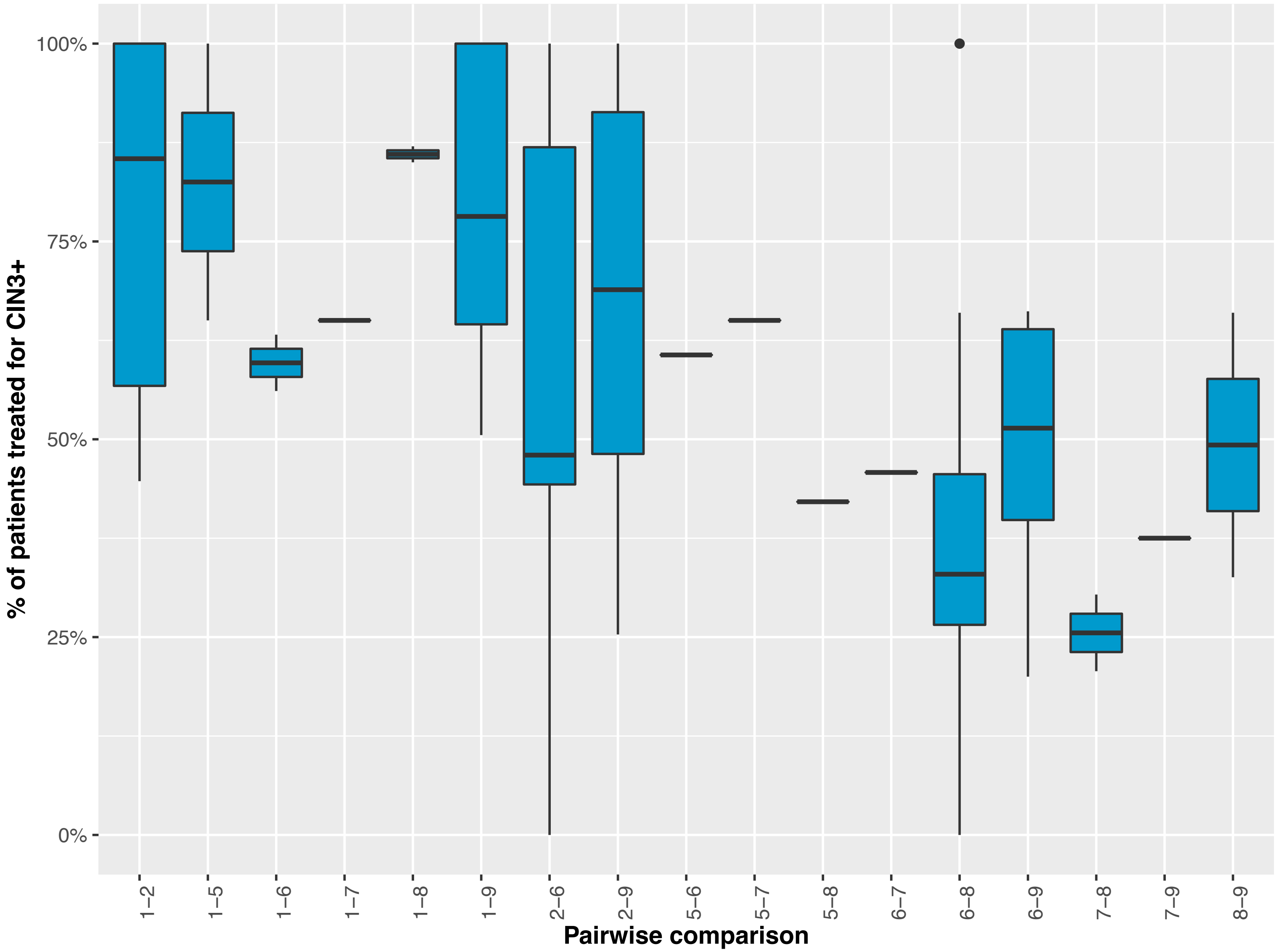

Median of the percentage of women treated for CIN3+ across studies: 58% (IQR=41–87); percentage of women treated for CIN3+ not reported in 11 studies

1: CKC; 2: LC; 5: RD; 6: LA; 7: CC; 8: CT; 9: LLETZ

Table 2.8.1.13: Risk of treatment failure in studies where ≥58% of women had been treated for CIN3+ (N=30 studies)

**CKC (P-score: 0·79)**	1·46 (0·82–2·59) (0·55–3·89)	0·31 (0·16–0·63) (0·11–0·91)	0·33 (0·19–0·59) (0·12–0·89)	0·79 (0·24–2·56) (0·18–3·37)	0·27 (0·15–0·51) (0·10–0·76)	0·63 (0·42–0·93) (0·26–1·51)
0·68 (0·39–1·21) (0·26–1·82)	**LC (P-score: 0·95)**	0·21 (0·09–0·52) (0·06–0·72)	0·23 (0·12–0·42) (0·08–0·62)	0·54 (0·15–1·99) (0·11–2·58)	0·19 (0·09–0·39) (0·06–0·56)	0·43 (0·26–0·70) (0·17–1·09)
3·19 (1·58–6·44) (1·10–9·26)	4·66 (1·91–11·40) (1·39–15·67)	**RD (P-score: 0·19)**	1·06 (0·44–2·56) (0·32–3·53)	2·51 (0·80–7·91) (0·60–10·45)	0·88 (0·35–2·21) (0·25–3·02)	2·00 (0·90–4·42) (0·64–6·21)
3·02 (1·69–5·40) (1·13–8·07)	4·41 (2·39–8·12) (1·62–12·02)	0·95 (0·39–2·29) (0·28–3·15)	**LA (P-score: 0·23)**	2·37 (0·64–8·74) (0·50–11·36)	0·83 (0·45–1·53) (0·30–2·26)	1·89 (1·14–3·13) (0·74–4·82)
1·27 (0·39–4·14) (0·30–5·45)	1·86 (0·50–6·87) (0·39–8·92)	0·40 (0·13–1·26) (0·10–1·66)	0·42 (0·11–1·56) (0·09–2·02)	**CC (P-score: 0·66)**	0·35 (0·09–1·32) (0·07–1·71)	0·80 (0·23–2·76) (0·18–3·60)
3·64 (1·95–6·79) (1·32–10·01)	5·32 (2·56–11·05) (1·79–15·78)	1·14 (0·45–2·87) (0·33–3·93)	1·21 (0·65–2·23) (0·44–3·30)	2·86 (0·76–10·81) (0·58–14·01)	**CT (P-score: 0·12)**	2·28 (1·24–4·18) (0·84–6·20)
1·60 (1·07–2·37) (0·66–3·84)	2·33 (1·42–3·84) (0·92–5·93)	0·50 (0·23–1·11) (0·16–1·56)	0·53 (0·32–0·88) (0·21–1·35)	1·26 (0·36–4·34) (0·28–5·67)	0·44 (0·24–0·80) (0·16–1·19)	**LLETZ (P-score: 0·55)**

Heterogeneity: τ^2=0·14; I^2=29% (0–54)

Table 2.8.1.14: Risk of treatment failure in studies where <58% of women had been treated for CIN3+ (N=30 studies)

**CKC (P-score: 0·83)**	0·82 (0·43–1·56) (0·30–2·21)	1·55 (0·26–9·27) (0·21–11·63)	0·45 (0·21–0·97) (0·15–1·34)	0·72 (0·29–1·80) (0·22–2·41)	0·45 (0·20–1·02) (0·15–1·39)	0·61 (0·30–1·24) (0·22–1·73)
1·23 (0·64–2·35) (0·45–3·33)	**LC (P-score: 0·78)**	1·90 (0·35–10·42) (0·28–13·11)	0·55 (0·33–0·94) (0·22–1·39)	0·89 (0·43–1·84) (0·31–2·55)	0·55 (0·30–1·02) (0·21–1·46)	0·75 (0·47–1·19) (0·31–1·80)
0·65 (0·11–3·86) (0·09–4·85)	0·53 (0·10–2·89) (0·08–3·64)	**RD (P-score: 0·66)**	0·29 (0·06–1·49) (0·05–1·88)	0·47 (0·09–2·57) (0·07–3·23)	0·29 (0·06–1·43) (0·05–1·81)	0·39 (0·07–2·09) (0·06–2·63)
2·21 (1·03–4·75) (0·75–6·54)	1·80 (1·06–3·06) (0·72–4·51)	3·42 (0·67–17·46) (0·53–22·00)	**LA (P-score: 0·57)**	1·60 (0·88–2·90) (0·61–4·18)	1·00 (0·70–1·42) (0·44–2·26)	1·35 (0·90–2·03) (0·58–3·15)
1·38 (0·56–3·42) (0·42–4·58)	1·13 (0·54–2·33) (0·39–3·24)	2·14 (0·39–11·73) (0·31–14·75)	0·62 (0·35–1·13) (0·24–1·63)	**CC (P-score: 0·41)**	0·62 (0·34–1·14) (0·24–1·64)	0·84 (0·46–1·55) (0·32–2·23)
2·21 (0·98–5·02) (0·72–6·83)	1·81 (0·98–3·31) (0·69–4·75)	3·43 (0·70–16·83) (0·55–21·24)	1·00 (0·71–1·42) (0·44–2·27)	1·60 (0·88–2·94) (0·61–4·22)	**CT (P-score: 0·13)**	1·35 (0·83–2·21) (0·55–3·30)
1·64 (0·81–3·31) (0·58–4·62)	1·33 (0·84–2·12) (0·56–3·20)	2·53 (0·48–13·38) (0·38–16·84)	0·74 (0·49–1·11) (0·32–1·72)	1·18 (0·64–2·18) (0·45–3·13)	0·74 (0·45–1·20) (0·30–1·80)	**LLETZ (P-score: 0·12)**

Heterogeneity: τ^2=0·13; I^2=35% (0–59)

Figure 2.8.1.9: Distribution of AIS across treatment comparisons

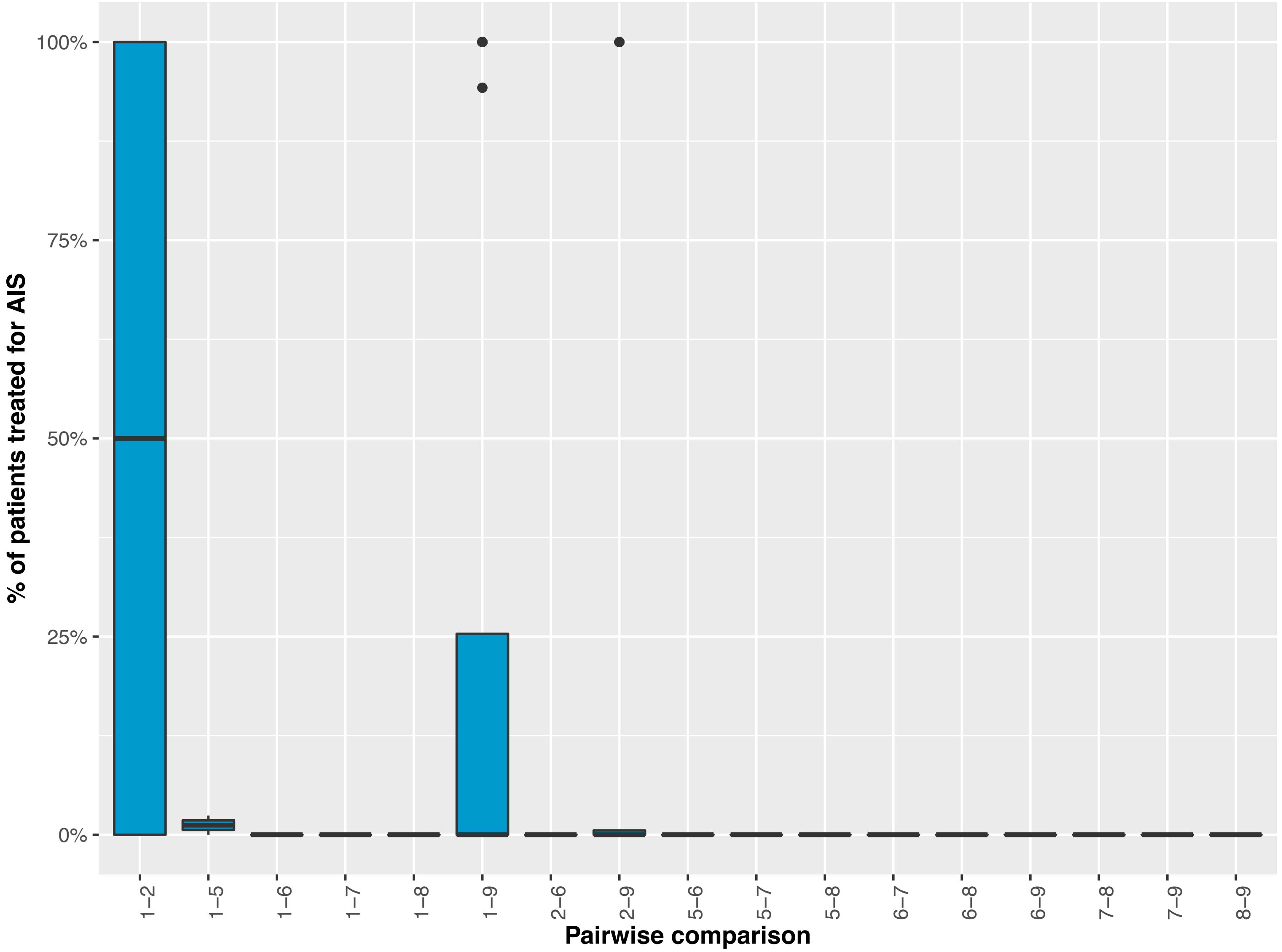

Median of the percentage of women treated for AIS across studies: 0% (IQR=0–0); percentage of women treated for AIS not reported in 4 studies

1: CKC; 2: LC; 5: RD; 6: LA; 7: CC; 8: CT; 9: LLETZ

Table 2.8.1.15: Risk of treatment failure in studies where >0% of women had been treated for AIS (N=14 studies)

**CKC**<br>**(P-score: 0·69)**	1·53<br>(0·87–2·70)<br>(0·74–3·17)	0·35<br>(0·18–0·66)<br>(0·16–0·77)	0·58<br>(0·39–0·87)<br>(0·33–1·04)
0·65<br>(0·37–1·15)<br>(0·32–1·36)	**LC**<br>**(P-score: 0·98)**	0·23<br>(0·10–0·54)<br>(0·08–0·63)	0·38<br>(0·24–0·60)<br>(0·20–0·71)
2·87<br>(1·51–5·45)<br>(1·29–6·39)	4·39<br>(1·87–10·34)<br>(1·59–12·16)	**RD**<br>**(P-score: 0·03)**	1·67<br>(0·78–3·57)<br>(0·67–4·18)
1·72<br>(1·15–2·58)<br>(0·96–3·06)	2·63<br>(1·66–4·15)<br>(1·41–4·91)	0·60<br>(0·28–1·28)<br>(0·24–1·50)	**LLETZ**<br>**(P-score: 0·30)**

Heterogeneity: τ^2=0·03; I^2=6% (0–58)

Table 2.8.1.16: Risk of treatment failure in studies where 0% of women had been treated for AIS (N=53 studies)

**CKC**<br>**(P-score: 0·93)**	0·92<br>(0·59–1·43)<br>(0·39–2·14)	0·38<br>(0·16–0·88)<br>(0·12–1·16)	0·38<br>(0·25–0·58)<br>(0·16–0·88)	0·60<br>(0·34–1·06)<br>(0·24–1·51)	0·35<br>(0·22–0·55)<br>(0·15–0·82)	0·66<br>(0·46–0·95)<br>(0·30–1·49)
1·09<br>(0·70–1·69)<br>(0·47–2·54)	**LC**<br>**(P-score: 0·87)**	0·41<br>(0·17–0·99)<br>(0·13–1·30)	0·41<br>(0·28–0·62)<br>(0·18–0·95)	0·65<br>(0·36–1·17)<br>(0·25–1·66)	0·38<br>(0·24–0·60)<br>(0·16–0·90)	0·72<br>(0·50–1·04)<br>(0·32–1·62)
2·65<br>(1·14–6·17)<br>(0·86–8·16)	2·44<br>(1·01–5·92)<br>(0·77–7·76)	**RD**<br>**(P-score: 0·22)**	1·01<br>(0·44–2·34)<br>(0·33–3·10)	1·58<br>(0·67–3·76)<br>(0·51–4·95)	0·93<br>(0·40–2·16)<br>(0·30–2·86)	1·76<br>(0·76–4·08)<br>(0·57–5·40)
2·62<br>(1·72–4·01)<br>(1·13–6·07)	2·41<br>(1·61–3·62)<br>(1·05–5·52)	0·99<br>(0·43–2·29)<br>(0·32–3·03)	**LA**<br>**(P-score: 0·21)**	1·56<br>(0·93–2·62)<br>(0·64–3·81)	0·92<br>(0·70–1·22)<br>(0·43–2·00)	1·74<br>(1·28–2·37)<br>(0·79–3·81)
1·68<br>(0·95–2·98)<br>(0·66–4·24)	1·54<br>(0·85–2·79)<br>(0·60–3·95)	0·63<br>(0·27–1·50)<br>(0·20–1·98)	0·64<br>(0·38–1·07)<br>(0·26–1·56)	**CC**<br>**(P-score: 0·54)**	0·59<br>(0·35–0·99)<br>(0·24–1·44)	1·11<br>(0·67–1·85)<br>(0·46–2·70)
2·84<br>(1·81–4·46)<br>(1·21–6·67)	2·61<br>(1·66–4·13)<br>(1·11–6·15)	1·07<br>(0·46–2·48)<br>(0·35–3·28)	1·08<br>(0·82–1·43)<br>(0·50–2·35)	1·70<br>(1·01–2·86)<br>(0·69–4·15)	**CT**<br>**(P-score: 0·12)**	1·89<br>(1·31–2·71)<br>(0·84–4·23)
1·51<br>(1·05–2·16)<br>(0·67–3·38)	1·39<br>(0·96–2·00)<br>(0·62–3·11)	0·57<br>(0·24–1·32)<br>(0·19–1·74)	0·57<br>(0·42–0·78)<br>(0·26–1·26)	0·90<br>(0·54–1·49)<br>(0·37–2·18)	0·53<br>(0·37–0·76)<br>(0·24–1·19)	**LLETZ**<br>**(P-score: 0·60)**

Heterogeneity: τ^2=0·13; I^2=38% (13–55)

Figure 2.8.1.10: Distribution of cervical cancer across treatment comparisons

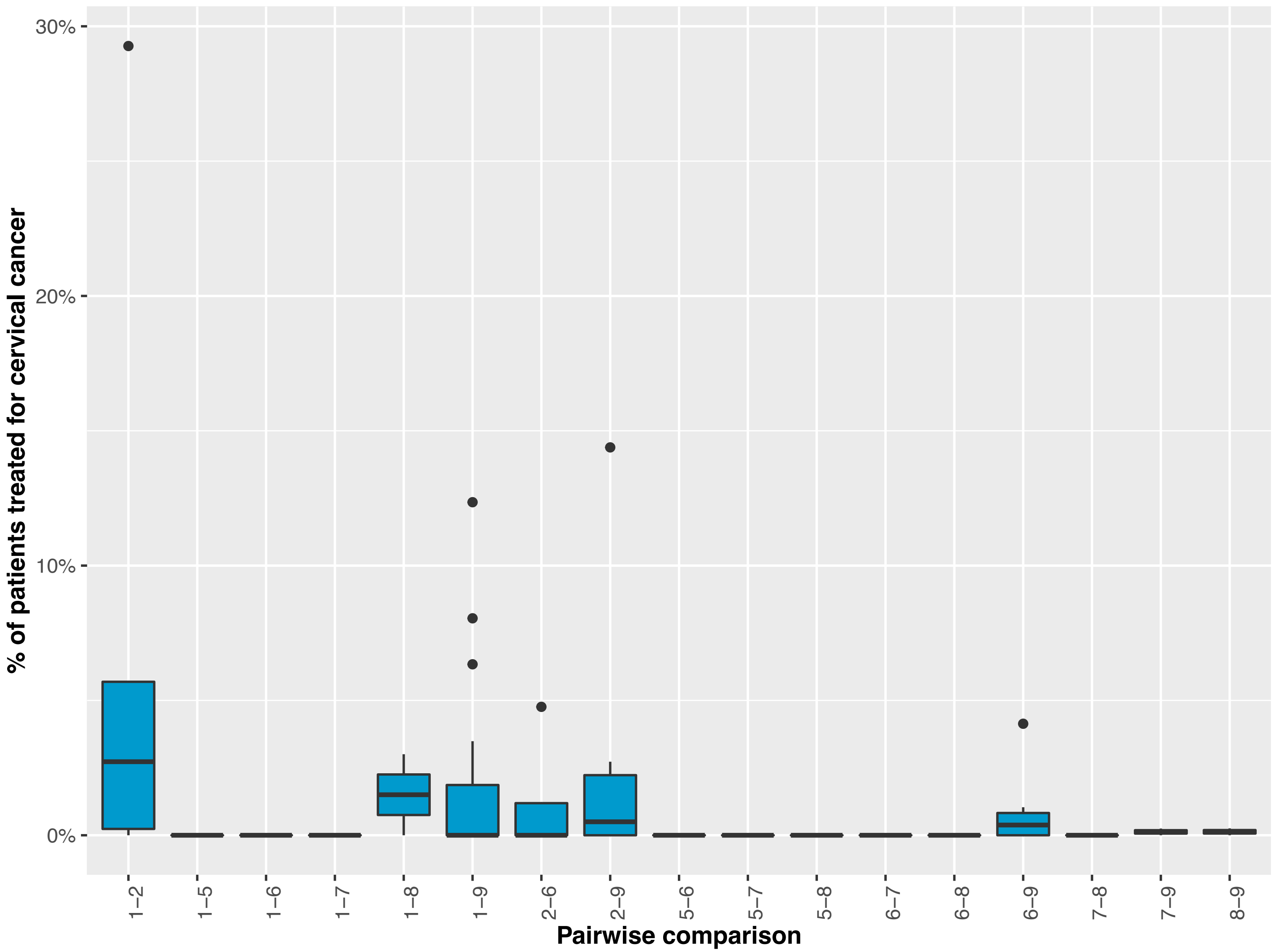

Median of the percentage of women treated for cancer across studies: 0% (IQR=0–1); percentage of women treated for cancer not reported in 5 studies

1: CKC; 2: LC; 5: RD; 6: LA; 7: CC; 8: CT; 9: LLETZ

Table 2.8.1.17: Risk of treatment failure in studies where >0% of women had been treated for cervical cancer (N=22 studies)

**CKC (P-score: 0·77)**	1·31 (0·81–2·11) (0·54–3·14)	0·37 (0·21–0·66) (0·14–0·95)	0·77 (0·29–2·01) (0·22–2·69)	0·28 (0·14–0·54) (0·10–0·77)	0·61 (0·40–0·92) (0·26–1·41)
0·76 (0·47–1·23) (0·32–1·83)	**LC (P-score: 0·94)**	0·28 (0·15–0·51) (0·11–0·73)	0·59 (0·22–1·55) (0·17–2·07)	0·21 (0·10–0·45) (0·07–0·62)	0·46 (0·30–0·72) (0·20–1·09)
2·72 (1·52–4·88) (1·06–7·03)	3·57 (1·95–6·52) (1·36–9·34)	**LA (P-score: 0·17)**	2·10 (0·80–5·52) (0·60–7·38)	0·76 (0·38–1·52) (0·27–2·13)	1·66 (1·07–2·56) (0·71–3·89)
1·30 (0·50–3·39) (0·37–4·54)	1·70 (0·64–4·49) (0·48–6·00)	0·48 (0·18–1·26) (0·14–1·68)	**CC (P-score: 0·61)**	0·36 (0·12–1·07) (0·09–1·41)	0·79 (0·33–1·87) (0·25–2·54)
3·57 (1·84–6·94) (1·30–9·78)	4·67 (2·24–9·77) (1·61–13·54)	1·31 (0·66–2·61) (0·47–3·66)	2·75 (0·94–8·05) (0·71–10·65)	**CT (P-score: 0·05)**	2·17 (1·14–4·12) (0·81–5·84)
1·65 (1·09–2·50) (0·71–3·82)	2·15 (1·39–3·35) (0·92–5·07)	0·60 (0·39–0·94) (0·26–1·42)	1·27 (0·53–3·00) (0·39–4·07)	0·46 (0·24–0·87) (0·17–1·24)	**LLETZ (P-score: 0·46)**

Heterogeneity: τ^2=0·12; I^2=34% (0–62)

Table 2.8.1.18: Risk of treatment failure in studies where 0% of women had been treated for cervical cancer (N=44 studies)

**CKC (P-score: 0·95)**	0·82 (0·45–1·50) (0·31–2·16)	0·33 (0·18–0·63) (0·12–0·89)	0·30 (0·17–0·53) (0·12–0·77)	0·45 (0·23–0·88) (0·16–1·24)	0·29 (0·16–0·52) (0·11–0·75)	0·60 (0·41–0·89) (0·26–1·39)
1·21 (0·67–2·21) (0·46–3·18)	**LC (P-score: 0·85)**	0·40 (0·18–0·90) (0·13–1·23)	0·37 (0·21–0·64) (0·14–0·93)	0·54 (0·26–1·15) (0·19–1·58)	0·35 (0·19–0·64) (0·13–0·92)	0·73 (0·45–1·18) (0·30–1·78)
3·00 (1·60–5·65) (1·12–8·05)	2·48 (1·11–5·53) (0·82–7·52)	**RD (P-score: 0·26)**	0·91 (0·44–1·88) (0·32–2·60)	1·34 (0·61–2·95) (0·45–4·02)	0·87 (0·42–1·81) (0·30–2·50)	1·80 (0·91–3·59) (0·65–5·02)
3·30 (1·90–5·73) (1·30–8·39)	2·72 (1·57–4·72) (1·07–6·92)	1·10 (0·53–2·27) (0·38–3·14)	**LA (P-score: 0·19)**	1·47 (0·80–2·71) (0·56–3·88)	0·96 (0·70–1·30) (0·43–2·14)	1·98 (1·23–3·18) (0·82–4·80)
2·24 (1·13–4·44) (0·81–6·22)	1·85 (0·87–3·91) (0·63–5·38)	0·75 (0·34–1·64) (0·25–2·24)	0·68 (0·37–1·25) (0·26–1·79)	**CC (P-score: 0·47)**	0·65 (0·35–1·19) (0·25–1·71)	1·34 (0·70–2·58) (0·49–3·65)
3·44 (1·94–6·11) (1·34–8·87)	2·84 (1·56–5·16) (1·08–7·43)	1·15 (0·55–2·38) (0·40–3·29)	1·04 (0·77–1·42) (0·47–2·33)	1·54 (0·84–2·82) (0·58–4·05)	**CT (P-score: 0·14)**	2·07 (1·25–3·42) (0·84–5·10)
1·67 (1·13–2·46) (0·72–3·87)	1·37 (0·85–2·23) (0·56–3·36)	0·56 (0·28–1·10) (0·20–1·55)	0·50 (0·31–0·81) (0·21–1·22)	0·74 (0·39–1·43) (0·27–2·02)	0·48 (0·29–0·80) (0·20–1·20)	**LLETZ (P-score: 0·64)**

Heterogeneity: τ^2=0·13; I^2=35% (6–55)

Table 2.8.1.19: Risk of treatment failure in women treated for biopsy-proven CIN2+ or persistent CIN1 (N=18 studies)

**CKC (P-score: 0·93)**	0·64 (0·16–2·54) (0·10–4·36)	0·35 (0·11–1·10) (0·06–1·94)	0·26 (0·07–0·90) (0·04–1·56)	0·44 (0·15–1·28) (0·09–2·29)	0·22 (0·05–0·92) (0·03–1·56)	0·58 (0·32–1·05) (0·16–2·17)
1·55 (0·39–6·11) (0·23–10·48)	**LC (P-score: 0·70)**	0·54 (0·09–3·23) (0·05–5·41)	0·40 (0·14–1·10) (0·08–1·99)	0·68 (0·16–2·87) (0·10–4·89)	0·34 (0·09–1·31) (0·05–2·26)	0·91 (0·26–3·13) (0·15–5·44)
2·87 (0·91–9·06) (0·52–15·99)	1·85 (0·31–11·08) (0·18–18·58)	**RD (P-score: 0·37)**	0·74 (0·14–4·03) (0·08–6·77)	1·27 (0·27–6·06) (0·16–10·26)	0·64 (0·10–3·95) (0·06–6·63)	1·68 (0·46–6·10) (0·27–10·56)
3·89 (1·12–13·56) (0·64–23·59)	2·51 (0·91–6·95) (0·50–12·55)	1·35 (0·25–7·39) (0·15–12·42)	**LA (P-score: 0·22)**	1·72 (0·48–6·13) (0·28–10·63)	0·87 (0·34–2·21) (0·18–4·05)	2·27 (0·76–6·84) (0·42–12·16)
2·27 (0·78–6·55) (0·44–11·73)	1·46 (0·35–6·12) (0·20–10·44)	0·79 (0·16–3·77) (0·10–6·38)	0·58 (0·16–2·08) (0·09–3·60)	**CC (P-score: 0·48)**	0·50 (0·13–1·94) (0·08–3·33)	1·32 (0·55–3·21) (0·29–5·97)
4·50 (1·09–18·52) (0·64–31·66)	2·90 (0·76–11·04) (0·44–19·01)	1·57 (0·25–9·69) (0·15–16·24)	1·16 (0·45–2·95) (0·25–5·42)	1·99 (0·52–7·63) (0·30–13·13)	**CT (P-score: 0·17)**	2·63 (0·73–9·52) (0·42–16·49)
1·71 (0·95–3·08) (0·46–6·35)	1·10 (0·32–3·81) (0·18–6·63)	0·60 (0·16–2·16) (0·09–3·75)	0·44 (0·15–1·32) (0·08–2·35)	0·76 (0·31–1·83) (0·17–3·41)	0·38 (0·11–1·38) (0·06–2·39)	**COLPO (P-score: 0·64)**

Heterogeneity: τ^2=0·26; I^2=42% (0–70)

Table 2.8.1.20: Risk of treatment failure in women treated for CIN3 (N=22 studies)

**CKC (P-score: 0·93)**	0·62 (0·14–2·73) (0·08–4·53)	0·32 (0·13–0·77) (0·07–1·46)	0·21 (0·08–0·61) (0·04–1·10)	0·37 (0·10–1·33) (0·06–2·28)	0·16 (0·06–0·47) (0·03–0·85)	0·51 (0·21–1·22) (0·11–2·33)
1·62 (0·37–7·13) (0·22–11·83)	**LC (P-score: 0·73)**	0·51 (0·11–2·45) (0·07–4·02)	0·34 (0·12–0·99) (0·07–1·79)	0·60 (0·11–3·31) (0·07–5·34)	0·26 (0·08–0·88) (0·05–1·53)	0·82 (0·21–3·15) (0·13–5·34)
3·16 (1·30–7·67) (0·68–14·55)	1·95 (0·41–9·35) (0·25–15·34)	**RD (P-score: 0·41)**	0·67 (0·21–2·14) (0·12–3·77)	1·17 (0·32–4·23) (0·19–7·26)	0·52 (0·16–1·62) (0·09–2·86)	1·60 (0·52–4·90) (0·29–8·71)
4·69 (1·65–13·31) (0·91–24·11)	2·90 (1·01–8·35) (0·56–15·08)	1·49 (0·47–4·72) (0·27–8·31)	**LA (P-score: 0·22)**	1·74 (0·46–6·65) (0·27–11·30)	0·77 (0·43–1·37) (0·20–2·95)	2·38 (1·03–5·48) (0·53–10·60)
2·69 (0·75–9·62) (0·44–16·53)	1·67 (0·30–9·18) (0·19–14·82)	0·85 (0·24–3·08) (0·14–5·28)	0·57 (0·15–2·19) (0·09–3·72)	**CC (P-score: 0·49)**	0·44 (0·12–1·59) (0·07–2·73)	1·37 (0·35–5·33) (0·21–9·02)
6·12 (2·14–17·55) (1·18–31·72)	3·79 (1·14–12·65) (0·65–22·05)	1·94 (0·62–6·09) (0·35–10·76)	1·31 (0·73–2·33) (0·34–5·03)	2·27 (0·63–8·25) (0·37–14·14)	**CT (P-score: 0·07)**	3·11 (1·29–7·49) (0·68–14·25)
1·97 (0·82–4·75) (0·43–9·04)	1·22 (0·32–4·69) (0·19–7·95)	0·62 (0·20–1·91) (0·11–3·39)	0·42 (0·18–0·97) (0·09–1·87)	0·73 (0·19–2·86) (0·11–4·83)	0·32 (0·13–0·78) (0·07–1·48)	**LLETZ (P-score: 0·65)**

Heterogeneity: τ^2=0·32; I^2=41% (0–65)

Table 2.8.1.21: Risk of treatment failure in women treated for AIS (N=7 studies)

**CKC**<br>**(P-score: 0·58)**	1·57<br>(0·54–4·53)<br>(0·42–5·90)	0·56<br>(0·30–1·05)<br>(0·26–1·22)
0·64<br>(0·22–1·84)<br>(0·17–2·39)	**LC**<br>**(P-score: 0·88)**	0·36<br>(0·11–1·18)<br>(0·08–1·59)
1·78<br>(0·96–3·32)<br>(0·82–3·88)	2·80<br>(0·85–9·25)<br>(0·63–12·45)	**LLETZ**<br>**(P-score: 0·03)**

Heterogeneity: τ^2=0·00; I^2=0% (0–68)

Table 2.8.1.22: Risk of treatment failure in studies where some women might have been treated for less clinically significant disease (e.g. non-persistent CIN1) (N=36 studies)

**CKC (P-score: 0·58)**	0·89 (0·46–1·73) (0·28–2·88)	0·36 (0·14–0·94) (0·09–1·43)	0·34 (0·19–0·61) (0·11–1·05)	0·57 (0·24–1·33) (0·16–2·07)	0·31 (0·17–0·56) (0·10–0·96)	0·56 (0·32–0·95) (0·18–1·67)
1·12 (0·58–2·16) (0·35–3·60)	**LC (P-score: 0·58)**	0·40 (0·14–1·14) (0·10–1·70)	0·38 (0·22–0·65) (0·13–1·14)	0·64 (0·26–1·56) (0·17–2·40)	0·35 (0·19–0·63) (0·11–1·08)	0·62 (0·39–0·99) (0·21–1·80)
2·77 (1·06–7·26) (0·70–10·98)	2·48 (0·87–7·04) (0·59–10·46)	**RD (P-score: 0·58)**	0·94 (0·37–2·43) (0·24–3·69)	1·58 (0·57–4·36) (0·38–6·52)	0·87 (0·34–2·21) (0·22–3·37)	1·54 (0·58–4·08) (0·38–6·16)
2·94 (1·64–5·27) (0·96–9·06)	2·63 (1·54–4·50) (0·88–7·90)	1·06 (0·41–2·74) (0·27–4·16)	**LA (P-score: 0·58)**	1·68 (0·78–3·58) (0·49–5·75)	0·92 (0·65–1·30) (0·33–2·53)	1·63 (1·09–2·44) (0·58–4·60)
1·76 (0·75–4·09) (0·48–6·39)	1·57 (0·64–3·84) (0·42–5·92)	0·63 (0·23–1·75) (0·15–2·62)	0·60 (0·28–1·28) (0·17–2·05)	**CC (P-score: 0·58)**	0·55 (0·26–1·17) (0·16–1·88)	0·98 (0·43–2·20) (0·27–3·47)
3·21 (1·78–5·76) (1·04–9·88)	2·87 (1·58–5·19) (0·93–8·88)	1·16 (0·45–2·95) (0·30–4·50)	1·09 (0·77–1·54) (0·40–3·00)	1·82 (0·86–3·89) (0·53–6·25)	**CT (P-score: 0·58)**	1·78 (1·12–2·82) (0·62–5·15)
1·80 (1·05–3·09) (0·60–5·42)	1·61 (1·01–2·56) (0·56–4·66)	0·65 (0·24–1·72) (0·16–2·60)	0·61 (0·41–0·91) (0·22–1·72)	1·02 (0·45–2·31) (0·29–3·65)	0·56 (0·35–0·89) (0·19–1·62)	**LLETZ (P-score: 0·58)**

Heterogeneity: τ^2=0·22; I^2=55% (34–69)

Table 2.8.1.23: Risk of treatment failure in women with ectocervical lesions and/or satisfactory colposcopy (N=26 studies)

**CKC (P-score: 0·87)**	0·41<br>(0·04–3·91)<br>(0·03–5·12)	0·33<br>(0·02–4·45)<br>(0·02–5·86)	0·19<br>(0·02–1·69)<br>(0·02–2·21)	0·21<br>(0·02–2·11)<br>(0·02–2·76)	0·17<br>(0·02–1·59)<br>(0·01–2·08)	0·31<br>(0·04–2·80)<br>(0·03–3·67)
2·47<br>(0·26–23·76)<br>(0·20–31·13)	**LC (P-score: 0·75)**	0·82<br>(0·17–3·86)<br>(0·13–5·12)	0·46<br>(0·23–0·94)<br>(0·15–1·39)	0·53<br>(0·21–1·35)<br>(0·15–1·90)	0·43<br>(0·20–0·91)<br>(0·14–1·33)	0·77<br>(0·42–1·42)<br>(0·27–2·17)
3·01<br>(0·22–40·41)<br>(0·17–53·17)	1·22<br>(0·26–5·76)<br>(0·20–7·64)	**RD (P-score: 0·58)**	0·57<br>(0·14–2·27)<br>(0·11–3·04)	0·64<br>(0·14–2·96)<br>(0·11–3·93)	0·52<br>(0·13–2·07)<br>(0·10–2·77)	0·94<br>(0·22–4·05)<br>(0·17–5·39)
5·32<br>(0·59–47·85)<br>(0·45–62·68)	2·16<br>(1·07–4·36)<br>(0·72–6·48)	1·77<br>(0·44–7·10)<br>(0·33–9·50)	**LA (P-score: 0·22)**	1·14<br>(0·57–2·26)<br>(0·38–3·37)	0·92<br>(0·67–1·28)<br>(0·38–2·22)	1·67<br>(1·05–2·65)<br>(0·65–4·29)
4·68<br>(0·47–46·11)<br>(0·36–60·43)	1·90<br>(0·74–4·86)<br>(0·53–6·85)	1·55<br>(0·34–7·13)<br>(0·25–9·47)	0·88<br>(0·44–1·74)<br>(0·30–2·60)	**CC (P-score: 0·33)**	0·81<br>(0·41–1·61)<br>(0·27–2·40)	1·47<br>(0·68–3·15)<br>(0·47–4·60)
5·76<br>(0·63–52·71)<br>(0·48–69·05)	2·33<br>(1·10–4·96)<br>(0·75–7·27)	1·91<br>(0·48–7·55)<br>(0·36–10·12)	1·08<br>(0·78–1·49)<br>(0·45–2·60)	1·23<br>(0·62–2·43)<br>(0·42–3·64)	**CT (P-score: 0·14)**	1·80<br>(1·07–3·05)<br>(0·68–4·81)
3·19<br>(0·36–28·53)<br>(0·27–37·38)	1·29<br>(0·70–2·38)<br>(0·46–3·64)	1·06<br>(0·25–4·54)<br>(0·19–6·05)	0·60<br>(0·38–0·95)<br>(0·23–1·54)	0·68<br>(0·32–1·46)<br>(0·22–2·14)	0·55<br>(0·33–0·94)<br>(0·21–1·48)	**LLETZ (P-score: 0·61)**

Heterogeneity: τ^2=0·15; I^2=45% (10–66)

Table 2.8.1.24: Risk of treatment failure in women with ectocervical lesions and/or satisfactory colposcopy; only studies with median age ≥33 years included (N=4 studies)

**CKC (P-score: 0·81)**	0·67 (0·08–5·81) (0·00–787758·40)	0·19 (0·02–1·63) (0·00–187132·80)	0·30 (0·04–2·46) (0·00–252474·40)
1·49 (0·17–12·82) (0·00–1737283·00)	**LC (P-score: 0·79)**	0·29 (0·12–0·70) (0·00–86·19)	0·45 (0·27–0·73) (0·02–10·76)
5·14 (0·61–43·03) (0·00–4941310·00)	3·46 (1·44–8·33) (0·01–1032·00)	**LA (P-score: 0·07)**	1·54 (0·72–3·32) (0·01–221·84)
3·33 (0·41–27·30) (0·00–2797044·00)	2·24 (1·37–3·66) (0·09–54·06)	0·65 (0·30–1·39) (0·00–93·07)	**LLETZ (P-score: 0·33)**

Heterogeneity: τ^2=0·00; I^2=0% (0–90)

Table 2.8.1.25: Risk of treatment failure in women with ectocervical lesions and/or satisfactory colposcopy; only studies with median age <33 years included (N=17 studies)

**LC (P-score: 0·43)**	1·76 (0·42–7·31) (0·36–8·66)	0·89 (0·45–1·75) (0·41–1·93)	0·92 (0·36–2·33) (0·32–2·63)	0·89 (0·44–1·79) (0·39–1·98)	1·51 (0·81–2·79) (0·74–3·07)
0·57 (0·14–2·36) (0·12–2·80)	**RD (P-score: 0·78)**	0·50 (0·14–1·77) (0·12–2·06)	0·52 (0·13–2·16) (0·11–2·56)	0·50 (0·14–1·75) (0·12–2·03)	0·86 (0·23–3·14) (0·20–3·67)
1·13 (0·57–2·23) (0·52–2·46)	1·99 (0·57–6·98) (0·49–8·13)	**LA (P-score: 0·29)**	1·04 (0·53–2·04) (0·48–2·25)	1·00 (0·79–1·27) (0·72–1·38)	1·70 (1·18–2·45) (1·09–2·65)
1·09 (0·43–2·75) (0·38–3·10)	1·91 (0·46–7·91) (0·39–9·37)	0·96 (0·49–1·89) (0·44–2·09)	**CC (P-score: 0·36)**	0·96 (0·48–1·93) (0·43–2·14)	1·63 (0·80–3·36) (0·72–3·72)
1·13 (0·56–2·29) (0·50–2·53)	1·99 (0·57–6·91) (0·49–8·04)	1·00 (0·79–1·27) (0·72–1·39)	1·04 (0·52–2·09) (0·47–2·31)	**CT (P-score: 0·29)**	1·70 (1·14–2·55) (1·05–2·77)
0·66 (0·36–1·23) (0·33–1·35)	1·17 (0·32–4·29) (0·27–5·03)	0·59 (0·41–0·85) (0·38–0·92)	0·61 (0·30–1·26) (0·27–1·39)	0·59 (0·39–0·88) (0·36–0·96)	**LLETZ (P-score: 0·84)**

Heterogeneity: τ^2=0·01; I^2=4% (0–57)

Table 2.8.1.26: Risk of treatment failure in women with endocervical lesions and/or unsatisfactory colposcopy (N=12 studies)

**CKC (P-score: 0·56)**	1·35 (0·81–2·24) (0·76–2·38)	0·59 (0·39–0·91) (0·37–0·95)
0·74 (0·45–1·23) (0·42–1·31)	**LC (P-score: 0·94)**	0·44 (0·23–0·84) (0·21–0·91)
1·69 (1·10–2·58) (1·05–2·72)	2·28 (1·18–4·38) (1·09–4·75)	**LLETZ (P-score: 0·01)**

Heterogeneity: τ^2=0·00; I^2=0% (0–57)

Table 2.8.1.27: Risk of treatment failure in women with endocervical lesions and/or unsatisfactory colposcopy; only studies with median age ≥33 years included (N=9 studies)

**CKC**<br>**(P-score: 0·55)**	1·35<br>(0·81–2·23)<br>(0·74–2·44)	0·52<br>(0·28–0·96)<br>(0·25–1·07)
0·74<br>(0·45–1·23)<br>(0·41–1·35)	**LC**<br>**(P-score: 0·93)**	0·38<br>(0·17–0·84)<br>(0·15–0·97)
1·94<br>(1·05–3·61)<br>(0·94–4·02)	2·61<br>(1·19–5·75)<br>(1·03–6·60)	**LLETZ**<br>**(P-score: 0·01)**

Heterogeneity: τ^2=0·00; I^2=0% (0–62)

Table 2.8.1.28: Risk of treatment failure in women with endocervical lesions and/or unsatisfactory colposcopy; only studies with median age <33 years included (N=2 studies)

**CKC (P-score: 0·74)**	0·78 (0·37–1·65) (NA, NA)
1·28 (0·61–2·71) (NA, NA)	**LLETZ (P-score: 0·26)**

Heterogeneity: τ^2=0·00; I^2=0% (95% CI NA)

Table 2.8.1.29: Risk of treatment failure in women with endocervical lesions and/or unsatisfactory colposcopy; women treated with LLETZ underwent the top-hat technique (N=6 studies)

**CKC**<br>**(P-score: 0·80)**	0·89<br>(0·37–2·10)<br>(0·26–3·02)	0·47<br>(0·28–0·79)<br>(0·23–0·98)
1·13<br>(0·48–2·68)<br>(0·33–3·85)	**LC**<br>**(P-score: 0·67)**	0·53<br>(0·26–1·09)<br>(0·19–1·47)
2·11<br>(1·27–3·53)<br>(1·02–4·37)	1·87<br>(0·92–3·82)<br>(0·68–5·14)	**Top-hat LLETZ**<br>**(P-score: 0·02)**

Heterogeneity: τ^2=0·00; I^2=0% (0–75); 6 studies

Table 2.8.1.30: Risk of treatment failure in women with endocervical lesions and/or unsatisfactory colposcopy; women treated with LLETZ underwent the standard technique (N=4 studies)

**CKC**<br>**(P-score: 0·61)**	1·18<br>(0·28–5·00)<br>(0·05–28·13)	0·71<br>(0·33–1·55)<br>(0·13–3·92)
0·85<br>(0·20–3·60)<br>(0·04–20·29)	**LC**<br>**(P-score: 0·67)**	0·60<br>(0·14–2·56)<br>(0·03–14·41)
1·41<br>(0·65–3·06)<br>(0·26–7·75)	1·66<br>(0·39–7·03)<br>(0·07–39·53)	**Standard LLETZ**<br>**(P-score: 0·22)**

Heterogeneity: τ^2=0·00; I^2=0% (0–85)

Table 2.8.1.31: Sensitivity analysis for risk of treatment failure: excluding non-randomised studies (N=25 studies)

**CKC (P-score: 0·84)**	0·67 (0·35–1·29) (0·33–1·34)	0·95 (0·23–3·91) (0·21–4·28)	0·45 (0·23–0·87) (0·22–0·90)	0·71 (0·30–1·69) (0·28–1·78)	0·49 (0·24–0·99) (0·23–1·03)	0·70 (0·40–1·24) (0·38–1·29)
1·50 (0·78–2·88) (0·75–3·00)	**LC (P-score: 0·51)**	1·42 (0·36–5·57) (0·33–6·08)	0·67 (0·38–1·16) (0·37–1·20)	1·06 (0·48–2·33) (0·46–2·45)	0·73 (0·40–1·33) (0·38–1·39)	1·05 (0·66–1·66) (0·64–1·71)
1·05 (0·26–4·31) (0·23–4·72)	0·70 (0·18–2·75) (0·16–3·00)	**RD (P-score: 0·71)**	0·47 (0·13–1·65) (0·12–1·79)	0·74 (0·19–2·88) (0·18–3·15)	0·51 (0·15–1·77) (0·14–1·92)	0·74 (0·20–2·69) (0·19–2·92)
2·24 (1·15–4·35) (1·11–4·54)	1·50 (0·86–2·60) (0·83–2·70)	2·13 (0·61–7·51) (0·56–8·14)	**LA (P-score: 0·09)**	1·58 (0·89–2·84) (0·85–2·94)	1·09 (0·80–1·49) (0·78–1·52)	1·57 (1·11–2·23) (1·09–2·28)
1·41 (0·59–3·37) (0·56–3·56)	0·95 (0·43–2·08) (0·41–2·19)	1·35 (0·35–5·22) (0·32–5·70)	0·63 (0·35–1·13) (0·34–1·17)	**CC (P-score: 0·58)**	0·69 (0·39–1·22) (0·38–1·26)	0·99 (0·51–1·92) (0·49–2·00)
2·05 (1·01–4·14) (0·97–4·34)	1·37 (0·75–2·51) (0·72–2·61)	1·95 (0·56–6·74) (0·52–7·30)	0·91 (0·67–1·25) (0·66–1·28)	1·45 (0·82–2·56) (0·79–2·66)	**CT (P-score: 0·20)**	1·44 (0·95–2·19) (0·92–2·25)
1·42 (0·80–2·52) (0·78–2·62)	0·95 (0·60–1·51) (0·58–1·55)	1·36 (0·37–4·94) (0·34–5·37)	0·64 (0·45–0·90) (0·44–0·92)	1·01 (0·52–1·95) (0·50–2·03)	0·69 (0·46–1·06) (0·45–1·08)	**LLETZ (P-score: 0·58)**

Heterogeneity: τ^2=0·00; I^2=0% (0–46)

Table 2.8.1.32: Sensitivity analysis for risk of treatment failure: excluding non-randomised studies at high risk of bias (N=37 studies)

**CKC (P-score: 0·83)**	1·01 (0·61–1·68) (0·40–2·54)	0·71 (0·16–3·17) (0·13–4·01)	0·39 (0·21–0·71) (0·14–1·04)	0·59 (0·23–1·51) (0·17–2·02)	0·37 (0·20–0·72) (0·14–1·03)	0·69 (0·45–1·08) (0·29–1·68)
0·99 (0·60–1·65) (0·39–2·50)	**LC (P-score: 0·84)**	0·70 (0·16–3·11) (0·13–3·93)	0·38 (0·22–0·69) (0·15–1·02)	0·58 (0·23–1·47) (0·17–1·98)	0·37 (0·20–0·69) (0·14–1·01)	0·69 (0·45–1·05) (0·29–1·65)
1·41 (0·32–6·29) (0·25–7·94)	1·42 (0·32–6·27) (0·25–7·92)	**RD (P-score: 0·56)**	0·55 (0·14–2·17) (0·11–2·77)	0·82 (0·18–3·85) (0·14–4·84)	0·53 (0·13–2·07) (0·11–2·64)	0·98 (0·23–4·09) (0·18–5·19)
2·58 (1·40–4·74) (0·96–6·93)	2·60 (1·45–4·64) (0·99–6·85)	1·83 (0·46–7·29) (0·36–9·28)	**LA (P-score: 0·15)**	1·51 (0·72–3·17) (0·51–4·46)	0·97 (0·70–1·33) (0·42–2·21)	1·79 (1·16–2·75) (0·74–4·32)
1·71 (0·66–4·39) (0·49–5·91)	1·72 (0·68–4·35) (0·51–5·87)	1·21 (0·26–5·67) (0·21–7·13)	0·66 (0·32–1·39) (0·22–1·96)	**CC (P-score: 0·46)**	0·64 (0·31–1·33) (0·22–1·87)	1·18 (0·51–2·74) (0·37–3·77)
2·67 (1·40–5·10) (0·97–7·36)	2·69 (1·44–5·00) (0·99–7·29)	1·89 (0·48–7·42) (0·38–9·46)	1·03 (0·75–1·42) (0·45–2·37)	1·56 (0·75–3·24) (0·53–4·57)	**CT (P-score: 0·12)**	1·85 (1·14–2·99) (0·74–4·59)
1·44 (0·93–2·24) (0·59–3·50)	1·45 (0·96–2·21) (0·61–3·49)	1·02 (0·24–4·30) (0·19–5·45)	0·56 (0·36–0·86) (0·23–1·35)	0·84 (0·36–1·96) (0·27–2·69)	0·54 (0·33–0·88) (0·22–1·34)	**LLETZ (P-score: 0·54)**

Heterogeneity: τ^2=0·14; I^2=39% (8–60)

Summary Table

Table 2.8.1.33: Subgroup and sensitivity analyses for risk of CIN treatment failure

Analysis / Comparison	N studies	CKC vs LLETZ	LC vs LLETZ	RD vs LLETZ	LA vs LLETZ	CC vs LLETZ	CT vs LLETZ
Main analysis	71	0·63 (0·50–0·81)	0·59 (0·44–0·79)	1·76 (0·97–3·20)	1·69 (1·27–2·24)	1·09 (0·68–1·74)	1·84 (1·33–2·56)
Studies published in or after 1997	36	0·63 (0·48–0·84)	0·41 (0·26–0·66)	–	1·39 (0·94–2·07)	1·12 (0·57–2·19)	1·68 (0·96–2·96)
Studies published before 1997	35	0·71 (0·43–1·17)	0·77 (0·52–1·15)	2·02 (0·99–4·12)	2·15 (1·37–3·37)	1·27 (0·61–2·62)	2·21 (1·37–3·57)
Studies with median age ≥33 years	33	0·61 (0·45–0·81)	0·40 (0·27–0·59)	1·74 (0·73–4·12)	1·17 (0·62–2·19)	0·79 (0·35–1·78)	1·24 (0·34–4·49)
Studies with median age <33 years	27	0·63 (0·35–1·13)	1·55 (0·93–2·59)	1·01 (0·29–3·59)	2·08 (1·62–2·66)	1·89 (0·95–3·73)	2·00 (1·51–2·66)
Studies with percentage of smokers ≥35%	3	0·88 (0·22–3·56)	–	–	1·08 (0·58–2·00)	4·50 (1·20–16·85)	1·61 (0·88–2·92)
Studies with percentage of smokers <35%	4	0·48 (0·21–1·09)	0·31 (0·01–9·52)	–	–	–	–
Studies where ascertainment of exposure was through hospital records	71	0·63 (0·50–0·81)	0·59 (0·44–0·79)	1·76 (0·97–3·20)	1·69 (1·27–2·24)	1·09 (0·68–1·74)	1·84 (1·33–2·56)
Studies where ascertainment of exposure was through registries	0	–	–	–	–	–	–
Studies where ascertainment of outcome was through hospital records	67	0·60 (0·46–0·78)	0·62 (0·45–0·85)	1·69 (0·91–3·14)	1·68 (1·23–2·30)	1·08 (0·66–1·76)	1·83 (1·29–2·60)
Studies where ascertainment of outcome was through registries	4	0·85 (0·43–1·66)	0·62 (0·26–1·45)	–	1·72 (1·01–2·90)	–	–
Studies conducted in middle-income countries	7	0·31 (0·13–0·74)	0·72 (0·12–4·31)	–	–	–	–
Studies conducted in high-income countries	64	0·70 (0·54–0·91)	0·60 (0·45–0·81)	1·91 (1·06–3·43)	1·72 (1·30–2·27)	1·13 (0·71–1·81)	1·87 (1·36–2·59)
Studies with percentage of women treated for high-grade disease (CIN2+) ≥89%	35	0·64 (0·46–0·89)	0·45 (0·30–0·67)	2·24 (1·14–4·38)	2·04 (1·25–3·33)	1·35 (0·60–3·04)	1·99 (1·14–3·48)

Studies with percentage of women treated for high-grade disease (CIN2+) <89%	32	0·64 (0·42–0·97)	0·84 (0·57–1·24)	0·49 (0·10–2·32)	1·53 (1·11–2·11)	0·96 (0·57–1·62)	1·68 (1·15–2·45)
Studies with percentage of women treated for high-grade disease (CIN3+) ≥58%	30	0·63 (0·42–0·93)	0·43 (0·26–0·70)	2·00 (0·90–4·42)	1·89 (1·14–3·13)	0·80 (0·23–2·76)	2·28 (1·24–4·18)
Studies with percentage of women treated for high-grade disease (CIN3+) <58%	30	0·61 (0·30–1·24)	0·75 (0·47–1·19)	0·39 (0·07–2·09)	1·35 (0·90–2·03)	0·84 (0·46–1·55)	1·35 (0·83–2·21)
Studies with percentage of women treated for AIS >0%	14	0·58 (0·39–0·87)	0·38 (0·24–0·60)	1·67 (0·78–3·57)	–	–	–
Studies with percentage of women treated for AIS =0%	53	0·66 (0·46–0·95)	0·72 (0·50–1·04)	1·76 (0·76–4·08)	1·74 (1·28–2·37)	1·11 (0·67–1·85)	1·89 (1·31–2·71)
Studies with percentage of women treated for cancer >0%	22	0·61 (0·40–0·92)	0·46 (0·30–0·72)	–	1·66 (1·07–2·56)	0·79 (0·33–1·87)	2·17 (1·14–4·12)
Studies with percentage of women treated for cancer =0%	44	0·60 (0·41–0·89)	0·73 (0·45–1·18)	1·80 (0·91–3·59)	1·98 (1·23–3·18)	1·34 (0·70–2·58)	2·07 (1·25–3·42)
Treatment only for biopsy-proven CIN2+ or persistent CIN1	18	0·58 (0·32–1·05)	0·91 (0·26–3·13)	1·68 (0·46–6·10)	2·27 (0·76–6·84)	1·32 (0·55–3·21)	2·63 (0·73–9·52)
Treatment only for CIN3	22	0·51 (0·21–1·22)	0·82 (0·21–3·15)	1·60 (0·52–4·90)	2·38 (1·03–5·48)	1·37 (0·35–5·33)	3·11 (1·29–7·49)
Treatment only for AIS	7	0·56 (0·30–1·05)	0·36 (0·11–1·18)	–	–	–	–
Treatment only for stage IA1 cervical cancer	Analysis not possible due to small number of cases						
Studies where some women might have been treated for clinically insignificant disease (e.g. non-persistent CIN1)	36	0·56 (0·32–0·95)	0·62 (0·39–0·99)	1·54 (0·58–4·08)	1·63 (1·09–2·44)	0·98 (0·43–2·20)	1·78 (1·12–2·82)
Women with ectocervical lesions and/or satisfactory colposcopy	26	0·31 (0·04–2·80)	0·77 (0·42–1·42)	0·94 (0·22–4·05)	1·67 (1·05–2·65)	1·47 (0·68–3·15)	1·80 (1·07–3·05)
Women with ectocervical lesions and/or satisfactory colposcopy; only studies with median age ≥33 years included	4	0·30 (0·04–2·46)	0·45 (0·27–0·73)	–	1·54 (0·72–3·32)	–	–
Women with ectocervical lesions and/or satisfactory colposcopy; only studies with median age <33 years included	17	–	1·51 (0·81–2·79)	0·86 (0·23–3·14)	1·70 (1·18–2·45)	1·63 (0·80–3·36)	1·70 (1·14–2·55)
Women with endocervical lesions and/or unsatisfactory colposcopy	12	0·59 (0·39–0·91)	0·44 (0·23–0·84)	–	–	–	–
Women with endocervical lesions and/or unsatisfactory colposcopy; only studies with median age ≥33 years included	9	0·52 (0·28–0·96)	0·38 (0·17–0·84)	–	–	–	–

Women with endocervical lesions and/or unsatisfactory colposcopy; only studies with median age <33 years included	2	0·78 (0·37–1·65)	–	–	–	–	–
Women with endocervical lesions and/or unsatisfactory colposcopy; top-hat LLETZ technique	6	0·47 (0·28–0·79)	0·53 (0·26–1·09)	–	–	–	–
Women with endocervical lesions and/or unsatisfactory colposcopy; standard LLETZ technique	4	0·71 (0·33–1·55)	0·60 (0·14–2·56)	–	–	–	–
Sensitivity analysis: excluding non-randomised studies	25	0·70 (0·40–1·24)	1·05 (0·66–1·66)	0·74 (0·20–2·69)	1·57 (1·11–2·23)	0·99 (0·51–1·92)	1·44 (0·95–2·19)
Sensitivity analysis: excluding non-randomised studies at high risk of bias	37	0·69 (0·45–1·08)	0·69 (0·45–1·05)	0·98 (0·23–4·09)	1·79 (1·16–2·75)	1·18 (0·51–2·74)	1·85 (1·14–2·99)

2.8.2. Preterm Birth

Figure 2.8.2.1: Distribution of publication year across treatment comparisons

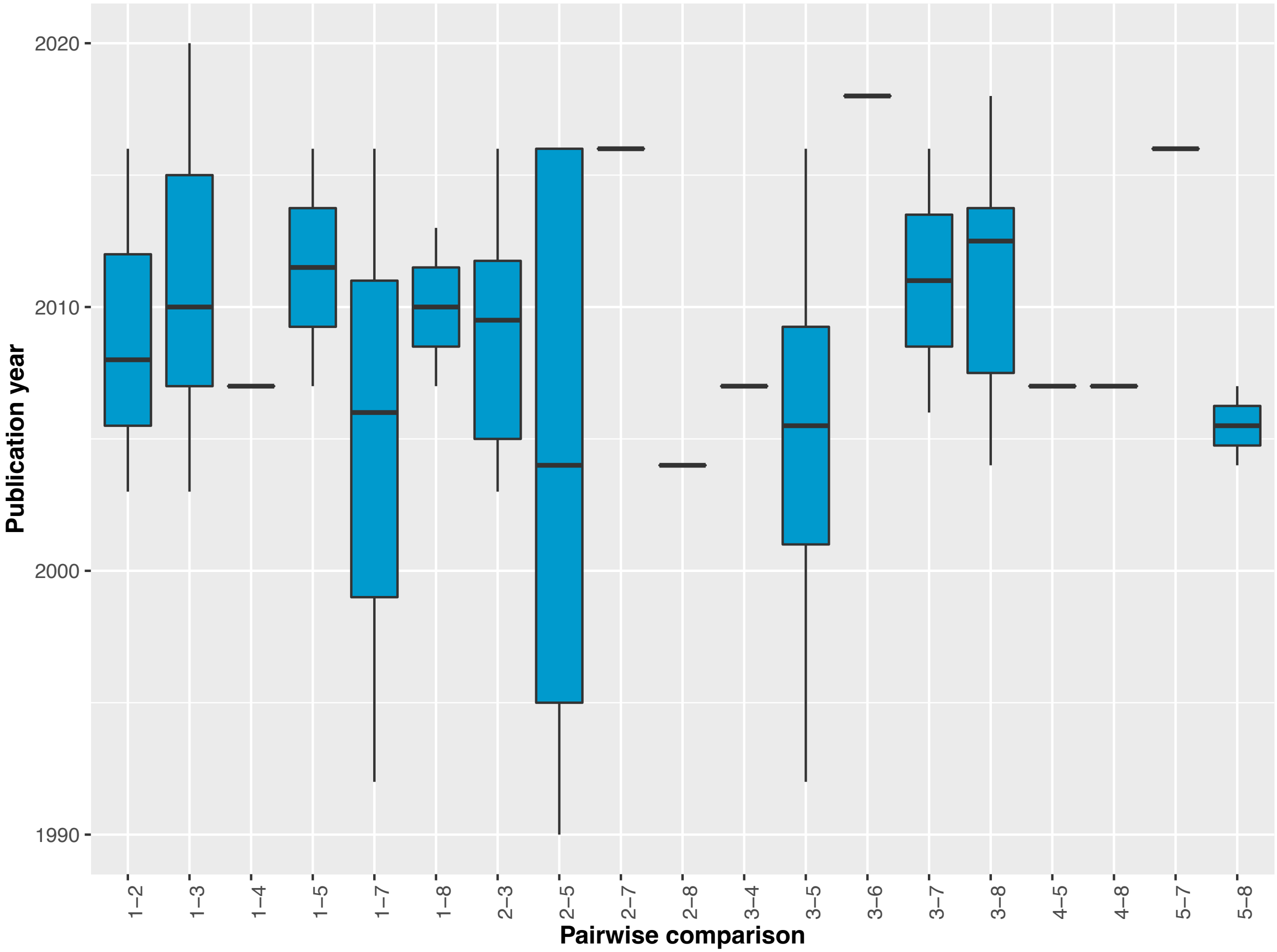

Median of the publication year across studies: 2011 (IQR=2007–2015)

1: CKC; 2: LC; 3: LLETZ; 4: RD; 5: LA; 6: CC; 7: CT; 8: COLPO

Table 2.8.2.1: Risk of preterm birth in studies published in or after 2011 (N=15 studies)

**CKC**<br>**(P-score: 0·18)**	1·04<br>(0·81–1·35)<br>(0·78–1·39)	1·64<br>(1·37–1·96)<br>(1·34–2·00)	1·77<br>(0·69–4·56)<br>(0·62–5·07)	3·35<br>(0·08–143·49)<br>(0·05–218·31)	1·45<br>(0·36–5·81)<br>(0·31–6·79)	2·06<br>(1·62–2·62)<br>(1·58–2·69)
0·96<br>(0·74–1·24)<br>(0·72–1·27)	**LC**<br>**(P-score: 0·23)**	1·57<br>(1·30–1·90)<br>(1·27–1·94)	1·70<br>(0·67–4·27)<br>(0·61–4·73)	3·20<br>(0·07–137·42)<br>(0·05–209·09)	1·39<br>(0·35–5·49)<br>(0·30–6·41)	1·97<br>(1·53–2·53)<br>(1·49–2·60)
0·61<br>(0·51–0·73)<br>(0·50–0·74)	0·64<br>(0·53–0·77)<br>(0·52–0·79)	**LLETZ**<br>**(P-score: 0·56)**	1·08<br>(0·43–2·74)<br>(0·38–3·03)	2·04<br>(0·05–87·13)<br>(0·03–132·50)	0·89<br>(0·22–3·51)<br>(0·19–4·09)	1·26<br>(1·07–1·48)<br>(1·05–1·51)
0·56<br>(0·22–1·45)<br>(0·20–1·61)	0·59<br>(0·23–1·48)<br>(0·21–1·64)	0·93<br>(0·37–2·34)<br>(0·33–2·60)	**LA**<br>**(P-score: 0·61)**	1·89<br>(0·04–90·29)<br>(0·03–139·06)	0·82<br>(0·16–4·26)<br>(0·13–5·12)	1·16<br>(0·45–2·98)<br>(0·41–3·32)
0·30<br>(0·01–12·81)<br>(0·00–19·49)	0·31<br>(0·01–13·39)<br>(0·00–20·37)	0·49<br>(0·01–20·92)<br>(0·01–31·81)	0·53<br>(0·01–25·32)<br>(0·01–38·99)	**CC**<br>**(P-score: 0·67)**	0·43<br>(0·01–23·66)<br>(0·01–36·97)	0·62<br>(0·01–26·36)<br>(0·01–40·10)
0·69<br>(0·17–2·75)<br>(0·15–3·21)	0·72<br>(0·18–2·84)<br>(0·16–3·31)	1·13<br>(0·29–4·47)<br>(0·24–5·21)	1·22<br>(0·23–6·33)<br>(0·20–7·61)	2·30<br>(0·04–125·52)<br>(0·03–196·15)	**CT**<br>**(P-score: 0·48)**	1·42<br>(0·35–5·66)<br>(0·30–6·61)
0·49<br>(0·38–0·62)<br>(0·37–0·63)	0·51<br>(0·40–0·65)<br>(0·38–0·67)	0·80<br>(0·68–0·94)<br>(0·66–0·96)	0·86<br>(0·34–2·21)<br>(0·30–2·46)	1·63<br>(0·04–69·65)<br>(0·02–105·96)	0·71<br>(0·18–2·82)<br>(0·15–3·29)	**COLPO**<br>**(P-score: 0·78)**

Heterogeneity: τ^2=0·00; I^2=0% (0–55)

Table 2.8.2.2: Risk of preterm birth in studies published before 2011 (N=14 studies)

**CKC (P-score: 0·08)**	1·82 (0·86–3·83) (0·75–4·38)	1·54 (0·92–2·58) (0·80–2·97)	1·21 (0·66–2·20) (0·58–2·52)	2·21 (1·25–3·91) (1·09–4·48)	3·25 (0·67–15·86) (0·56–18·84)	2·25 (1·33–3·80) (1·15–4·37)
0·55 (0·26–1·16) (0·23–1·33)	**LC (P-score: 0·55)**	0·85 (0·47–1·52) (0·41–1·75)	0·66 (0·34–1·29) (0·30–1·47)	1·22 (0·68–2·19) (0·59–2·51)	1·79 (0·33–9·76) (0·27–11·68)	1·24 (0·70–2·20) (0·61–2·52)
0·65 (0·39–1·09) (0·34–1·25)	1·18 (0·66–2·12) (0·57–2·43)	**LLETZ (P-score: 0·39)**	0·78 (0·51–1·20) (0·44–1·39)	1·44 (1·00–2·06) (0·86–2·41)	2·11 (0·43–10·48) (0·36–12·45)	1·46 (1·15–1·86) (0·95–2·25)
0·83 (0·45–1·51) (0·40–1·73)	1·51 (0·78–2·92) (0·68–3·34)	1·28 (0·83–1·95) (0·72–2·26)	**RD (P-score: 0·18)**	1·83 (1·21–2·78) (1·04–3·23)	2·70 (0·52–13·91) (0·44–16·59)	1·86 (1·27–2·72) (1·09–3·18)
0·45 (0·26–0·80) (0·22–0·92)	0·82 (0·46–1·48) (0·40–1·69)	0·70 (0·49–1·00) (0·41–1·17)	0·55 (0·36–0·82) (0·31–0·96)	**LA (P-score: 0·75)**	1·47 (0·29–7·48) (0·24–8·91)	1·02 (0·74–1·39) (0·63–1·65)
0·31 (0·06–1·50) (0·05–1·78)	0·56 (0·10–3·04) (0·09–3·64)	0·47 (0·10–2·34) (0·08–2·78)	0·37 (0·07–1·91) (0·06–2·28)	0·68 (0·13–3·46) (0·11–4·12)	**CT (P-score: 0·79)**	0·69 (0·14–3·45) (0·12–4·11)
0·45 (0·26–0·75) (0·23–0·87)	0·81 (0·45–1·44) (0·40–1·65)	0·68 (0·54–0·87) (0·45–1·05)	0·54 (0·37–0·78) (0·31–0·92)	0·98 (0·72–1·35) (0·61–1·60)	1·45 (0·29–7·24) (0·24–8·61)	**COLPO (P-score: 0·77)**

Heterogeneity: τ^2=0·03; I^2=15% (0–51)

Figure 2.8.2.2: Distribution of age at pregnancy across treatment comparisons

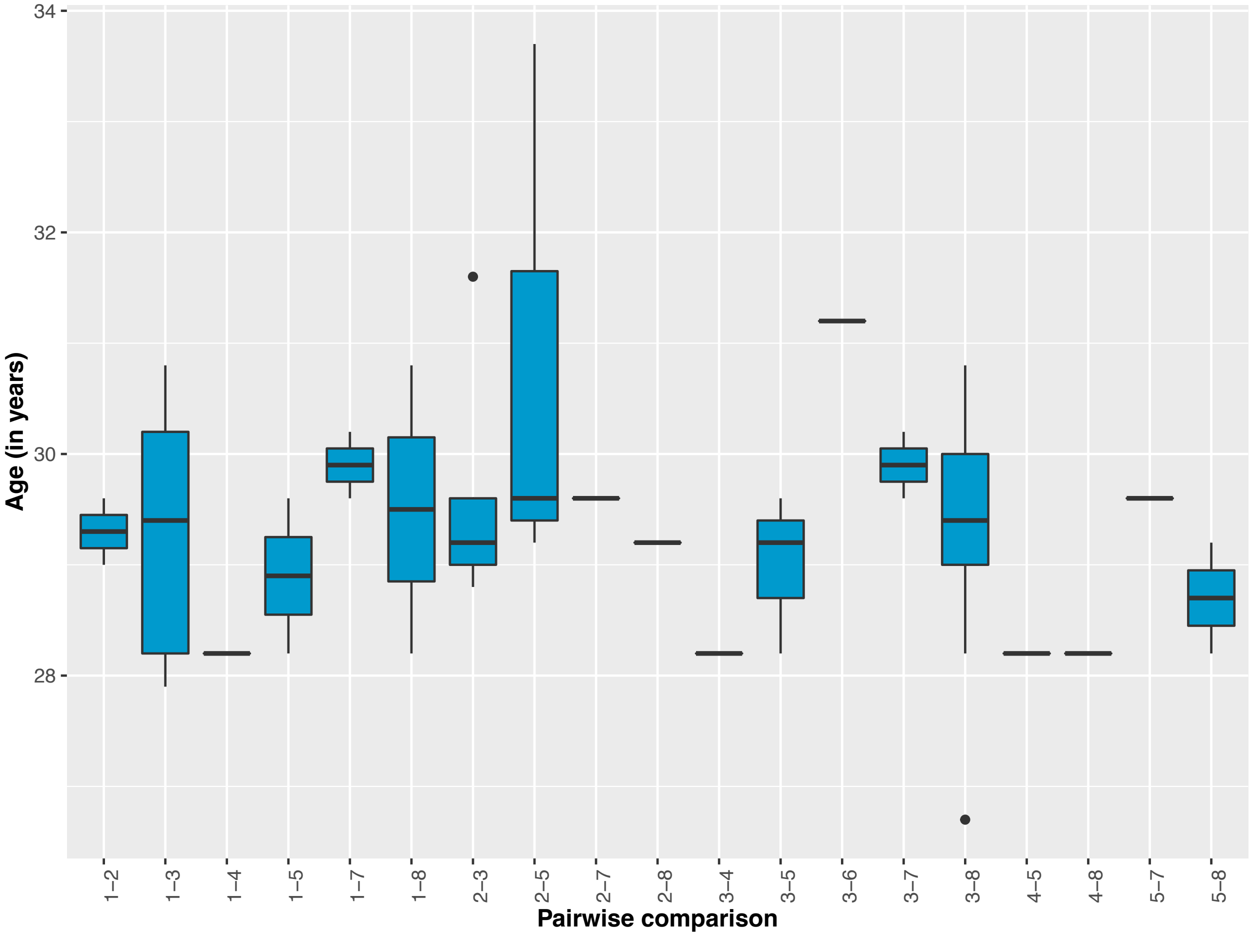

Median of the median age across studies: 30y (IQR=29–30); mean was used if median not reported; neither median nor mean reported in 9 studies

1: CKC; 2: LC; 3: LLETZ; 4: RD; 5: LA; 6: CC; 7: CT; 8: COLPO

Table 2.8.2.3: Risk of preterm birth in studies with median age ≥30 years (N=9 studies)

**CKC (P-score: 0·14)**	3·59 (0·53–24·26) (0·24–53·75)	1·61 (0·83–3·12) (0·63–4·12)	2·26 (0·05–111·55) (0·01–565·93)	3·28 (0·07–148·49) (0·01–726·82)	8·54 (1·02–71·37) (0·42–172·84)	2·67 (1·37–5·19) (1·04–6·85)
0·28 (0·04–1·88) (0·02–4·17)	**LC (P-score: 0·62)**	0·45 (0·07–2·68) (0·04–5·66)	0·63 (0·02–18·84) (0·01–77·60)	0·91 (0·01–58·49) (0·00–330·80)	2·38 (0·15–36·86) (0·05–115·48)	0·74 (0·12–4·46) (0·06–9·42)
0·62 (0·32–1·21) (0·24–1·59)	2·23 (0·37–13·39) (0·18–28·24)	**LLETZ (P-score: 0·33)**	1·41 (0·03–65·54) (0·01–324·70)	2·04 (0·05–87·13) (0·01–416·24)	5·31 (0·67–42·32) (0·28–100·45)	1·66 (1·50–1·84) (1·43–1·92)
0·44 (0·01–21·81) (0·00–110·65)	1·59 (0·05–47·52) (0·01–195·74)	0·71 (0·02–33·12) (0·00–164·10)	**LA (P-score: 0·47)**	1·45 (0·01–312·09) (0·00–2924·27)	3·78 (0·05–297·33) (0·01–1832·96)	1·18 (0·03–55·00) (0·01–272·63)
0·30 (0·01–13·79) (0·00–67·51)	1·09 (0·02–70·09) (0·00–396·43)	0·49 (0·01–20·92) (0·00–99·94)	0·69 (0·00–148·27) (0·00–1389·30)	**CC (P-score: 0·55)**	2·60 (0·04–189·82) (0·01–1133·38)	0·81 (0·02–34·74) (0·00–166·05)
0·12 (0·01–0·98) (0·01–2·37)	0·42 (0·03–6·52) (0·01–20·43)	0·19 (0·02–1·50) (0·01–3·56)	0·26 (0·00–20·85) (0·00–128·54)	0·38 (0·01–28·02) (0·00–167·29)	**CT (P-score: 0·82)**	0·31 (0·04–2·49) (0·02–5·92)
0·38 (0·19–0·73) (0·15–0·96)	1·35 (0·22–8·10) (0·11–17·10)	0·60 (0·54–0·67) (0·52–0·70)	0·85 (0·02–39·58) (0·00–196·19)	1·23 (0·03–52·62) (0·01–251·52)	3·20 (0·40–25·57) (0·17–60·75)	**COLPO (P-score: 0·58)**

Heterogeneity: τ^2=0·00; I^2=0% (0–75)

Table 2.8.2.4: Risk of preterm birth in studies with median age <30 years (N=11 studies)

**CKC (P-score: 0·19)**	1·04 (0·81–1·33) (0·79–1·36)	1·59 (1·33–1·90) (1·31–1·94)	1·03 (0·74–1·43) (0·72–1·48)	1·91 (1·42–2·57) (1·37–2·65)	1·43 (0·36–5·71) (0·31–6·58)	1·90 (1·51–2·39) (1·48–2·44)
0·97 (0·75–1·24) (0·73–1·27)	**LC (P-score: 0·24)**	1·54 (1·29–1·84) (1·26–1·87)	0·99 (0·72–1·38) (0·69–1·43)	1·84 (1·37–2·47) (1·33–2·55)	1·38 (0·35–5·45) (0·30–6·27)	1·83 (1·46–2·30) (1·43–2·36)
0·63 (0·53–0·75) (0·52–0·76)	0·65 (0·54–0·78) (0·53–0·79)	**LLETZ (P-score: 0·61)**	0·65 (0·49–0·86) (0·47–0·88)	1·20 (0·93–1·54) (0·91–1·58)	0·90 (0·23–3·55) (0·20–4·08)	1·19 (1·02–1·39) (1·01–1·41)
0·97 (0·70–1·34) (0·68–1·39)	1·01 (0·72–1·40) (0·70–1·44)	1·55 (1·17–2·05) (1·13–2·11)	**RD (P-score: 0·23)**	1·85 (1·38–2·49) (1·34–2·56)	1·39 (0·34–5·64) (0·30–6·51)	1·84 (1·44–2·35) (1·41–2·41)
0·52 (0·39–0·71) (0·38–0·73)	0·54 (0·40–0·73) (0·39–0·75)	0·83 (0·65–1·07) (0·63–1·10)	0·54 (0·40–0·73) (0·39–0·75)	**LA (P-score: 0·85)**	0·75 (0·19–3·02) (0·16–3·49)	1·00 (0·80–1·24) (0·78–1·26)
0·70 (0·17–2·80) (0·15–3·22)	0·72 (0·18–2·86) (0·16–3·29)	1·11 (0·28–4·41) (0·24–5·07)	0·72 (0·18–2·93) (0·15–3·38)	1·34 (0·33–5·39) (0·29–6·22)	**CT (P-score: 0·53)**	1·33 (0·33–5·30) (0·29–6·10)
0·53 (0·42–0·66) (0·41–0·68)	0·55 (0·43–0·69) (0·42–0·70)	0·84 (0·72–0·98) (0·71–0·99)	0·54 (0·42–0·69) (0·41–0·71)	1·00 (0·81–1·25) (0·79–1·28)	0·75 (0·19–3·00) (0·16–3·45)	**COLPO (P-score: 0·85)**

Heterogeneity: τ^2=0·00; I^2=0% (0–54)

Figure 2.8.2.3: Distribution of nulliparity across treatment comparisons

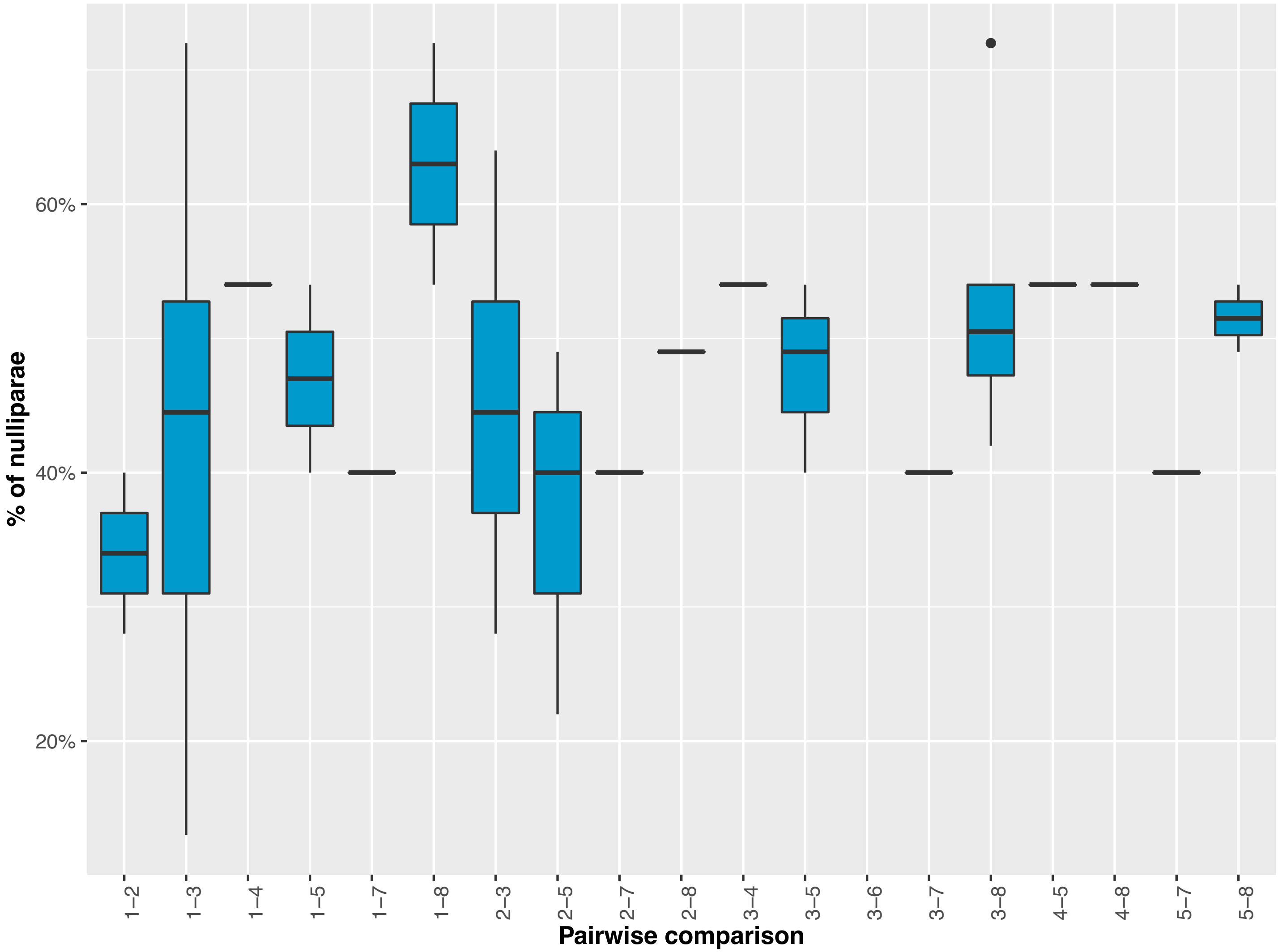

Median of the percentage of nulliparae across studies: 49% (IQR=41–54); percentage of nulliparae not reported in 15 studies

1: CKC; 2: LC; 3: LLETZ; 4: RD; 5: LA; 6: CC; 7: CT; 8: COLPO

Table 2.8.2.5: Risk of preterm birth in studies where ≥49% of women were nulliparous (N=7 studies)

**CKC (P-score: 0·11)**	1·44 (0·80–2·59) (0·71–2·92)	1·61 (1·34–1·93) (1·29–2·00)	1·09 (0·78–1·53) (0·73–1·64)	2·05 (1·50–2·81) (1·41–3·00)	2·02 (1·58–2·57) (1·50–2·70)
0·69 (0·39–1·25) (0·34–1·41)	**LC (P-score: 0·11)**	1·12 (0·64–1·96) (0·57–2·20)	0·76 (0·42–1·39) (0·37–1·57)	1·43 (0·80–2·53) (0·71–2·85)	1·40 (0·80–2·44) (0·72–2·73)
0·62 (0·52–0·75) (0·50–0·78)	0·90 (0·51–1·57) (0·45–1·77)	**LLETZ (P-score: 0·11)**	0·68 (0·51–0·91) (0·48–0·97)	1·28 (0·98–1·67) (0·92–1·77)	1·25 (1·05–1·50) (1·01–1·55)
0·91 (0·65–1·28) (0·61–1·37)	1·32 (0·72–2·40) (0·64–2·72)	1·47 (1·09–1·97) (1·03–2·10)	**RD (P-score: 0·11)**	1·88 (1·39–2·53) (1·31–2·69)	1·84 (1·44–2·35) (1·37–2·48)
0·49 (0·36–0·67) (0·33–0·71)	0·70 (0·39–1·25) (0·35–1·40)	0·78 (0·60–1·02) (0·57–1·08)	0·53 (0·40–0·72) (0·37–0·76)	**LA (P-score: 0·11)**	0·98 (0·79–1·22) (0·75–1·28)
0·50 (0·39–0·63) (0·37–0·66)	0·71 (0·41–1·24) (0·37–1·39)	0·80 (0·67–0·95) (0·64–0·99)	0·54 (0·42–0·69) (0·40–0·73)	1·02 (0·82–1·27) (0·78–1·33)	**COLPO (P-score: 0·11)**

Heterogeneity: τ^2=0·00; I^2=0% (0–65)

Table 2.8.2.6: Risk of preterm birth in studies where <49% of women were nulliparous (N=7 studies)

**CKC (P-score: 0·18)**	1·12 (0·61–2·07) (0·49–2·56)	1·79 (0·99–3·24) (0·80–3·98)	1·55 (0·48–4·99) (0·33–7·26)	1·56 (0·35–6·97) (0·22–11·21)	2·76 (1·50–5·09) (1·21–6·30)
0·89 (0·48–1·65) (0·39–2·04)	**LC (P-score: 0·24)**	1·60 (1·26–2·03) (1·11–2·30)	1·39 (0·50–3·83) (0·36–5·33)	1·40 (0·35–5·56) (0·23–8·64)	2·47 (1·86–3·27) (1·63–3·74)
0·56 (0·31–1·01) (0·25–1·24)	0·63 (0·49–0·79) (0·43–0·90)	**LLETZ (P-score: 0·63)**	0·87 (0·31–2·41) (0·22–3·35)	0·87 (0·22–3·48) (0·14–5·41)	1·54 (1·32–1·80) (1·17–2·03)
0·65 (0·20–2·08) (0·14–3·02)	0·72 (0·26–2·00) (0·19–2·78)	1·16 (0·42–3·21) (0·30–4·48)	**LA (P-score: 0·51)**	1·01 (0·18–5·54) (0·11–9·48)	1·78 (0·63–5·01) (0·45–7·00)
0·64 (0·14–2·85) (0·09–4·58)	0·72 (0·18–2·85) (0·12–4·42)	1·14 (0·29–4·56) (0·18–7·09)	0·99 (0·18–5·43) (0·11–9·31)	**CT (P-score: 0·51)**	1·76 (0·44–7·09) (0·28–11·05)
0·36 (0·20–0·67) (0·16–0·83)	0·41 (0·31–0·54) (0·27–0·61)	0·65 (0·56–0·76) (0·49–0·85)	0·56 (0·20–1·58) (0·14–2·21)	0·57 (0·14–2·28) (0·09–3·55)	**COLPO (P-score: 0·93)**

Heterogeneity: τ^2=0·01; I^2=8% (0–73)

Figure 2.8.2.4: Distribution of smoking across treatment comparisons

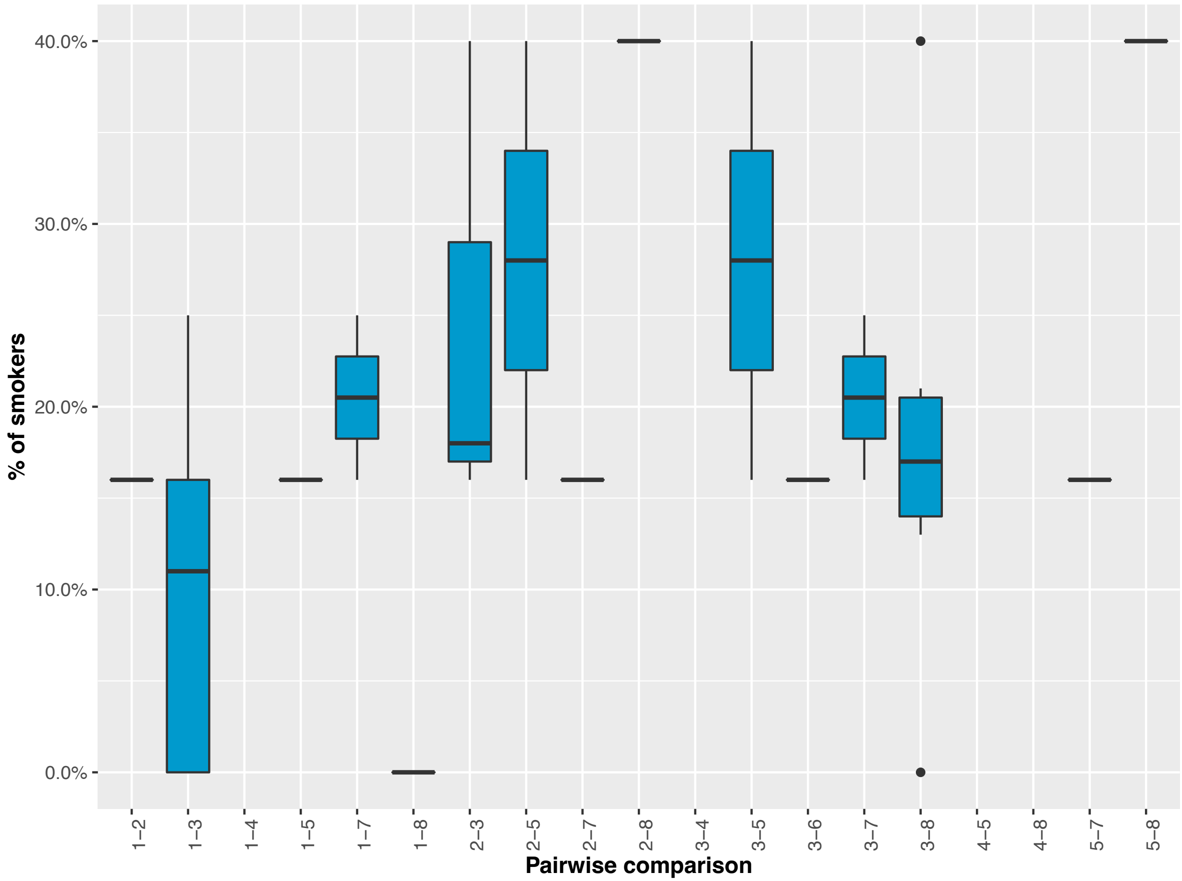

Median of the percentage of smokers across studies: 16% (IQR=13–20); percentage of smokers not reported in 16 studies

1: CKC; 2: LC; 3: LLETZ; 4: RD; 5: LA; 6: CC; 7: CT; 8: COLPO

Table 2.8.2.7: Risk of preterm birth in studies where ≥16% of women smoked (N=8 studies)

**CKC (P-score: 0·13)**	1·29 (0·58–2·87) (0·42–3·92)	1·64 (0·76–3·56) (0·56–4·85)	2·45 (0·98–6·10) (0·71–8·38)	3·35 (0·07–159·18) (0·03–364·69)	2·76 (0·72–10·64) (0·50–15·38)	2·31 (1·02–5·22) (0·75–7·12)
0·78 (0·35–1·73) (0·26–2·36)	**LC (P-score: 0·21)**	1·28 (0·86–1·89) (0·62–2·63)	1·90 (1·05–3·43) (0·77–4·67)	2·60 (0·06–116·64) (0·03–264·18)	2·14 (0·64–7·20) (0·45–10·20)	1·79 (1·14–2·82) (0·83–3·89)
0·61 (0·28–1·32) (0·21–1·79)	0·78 (0·53–1·16) (0·38–1·62)	**LLETZ (P-score: 0·40)**	1·49 (0·87–2·55) (0·64–3·48)	2·04 (0·05–89·55) (0·02–202·00)	1·68 (0·51–5·54) (0·36–7·85)	1·40 (1·06–1·86) (0·74–2·68)
0·41 (0·16–1·02) (0·12–1·40)	0·53 (0·29–0·95) (0·21–1·30)	0·67 (0·39–1·15) (0·29–1·57)	**LA (P-score: 0·72)**	1·37 (0·03–62·56) (0·01–142·19)	1·13 (0·31–4·10) (0·22–5·88)	0·94 (0·54–1·64) (0·40–2·24)
0·30 (0·01–14·14) (0·00–32·40)	0·38 (0·01–17·19) (0·00–38·94)	0·49 (0·01–21·5) (0·00–48·5)	0·73 (0·02–33·23) (0·01–75·53)	**CC (P-score: 0·62)**	0·82 (0·02–43·42) (0·01–101·60)	0·69 (0·02–30·52) (0·01–68·99)
0·36 (0·09–1·39) (0·07–2·02)	0·47 (0·14–1·56) (0·10–2·22)	0·60 (0·18–1·96) (0·13–2·78)	0·89 (0·24–3·21) (0·17–4·60)	1·21 (0·02–64·05) (0·01–149·86)	**CT (P-score: 0·71)**	0·84 (0·25–2·84) (0·17–4·03)
0·43 (0·19–0·98) (0·14–1·34)	0·56 (0·35–0·88) (0·26–1·21)	0·71 (0·54–0·94) (0·37–1·36)	1·06 (0·61–1·84) (0·45–2·51)	1·45 (0·03–64·43) (0·01–145·64)	1·20 (0·35–4·06) (0·25–5·76)	**COLPO (P-score: 0·70)**

Heterogeneity: τ^2=0·05; I^2=56% (8–79)

Table 2.8.2.8: Risk of preterm birth in studies where <16% of women smoked (N=5 studies)

**CKC**<br>**(P-score: 0·00)**	1·65<br>(1·37–1·99)<br>(1·22–2·23)	2·26<br>(1·47–3·48)<br>(1·12–4·55)
0·61<br>(0·50–0·73)<br>(0·45–0·82)	**LLETZ**<br>**(P-score: 0·53)**	1·37<br>(0·92–2·05)<br>(0·71–2·63)
0·44<br>(0·29–0·68)<br>(0·22–0·89)	0·73<br>(0·49–1·09)<br>(0·38–1·40)	**COLPO**<br>**(P-score: 0·97)**

Heterogeneity: τ^2=0·00; I^2=0% (0–79)

Figure 2.8.2.5: Method of ascertainment of exposure across treatment comparisons

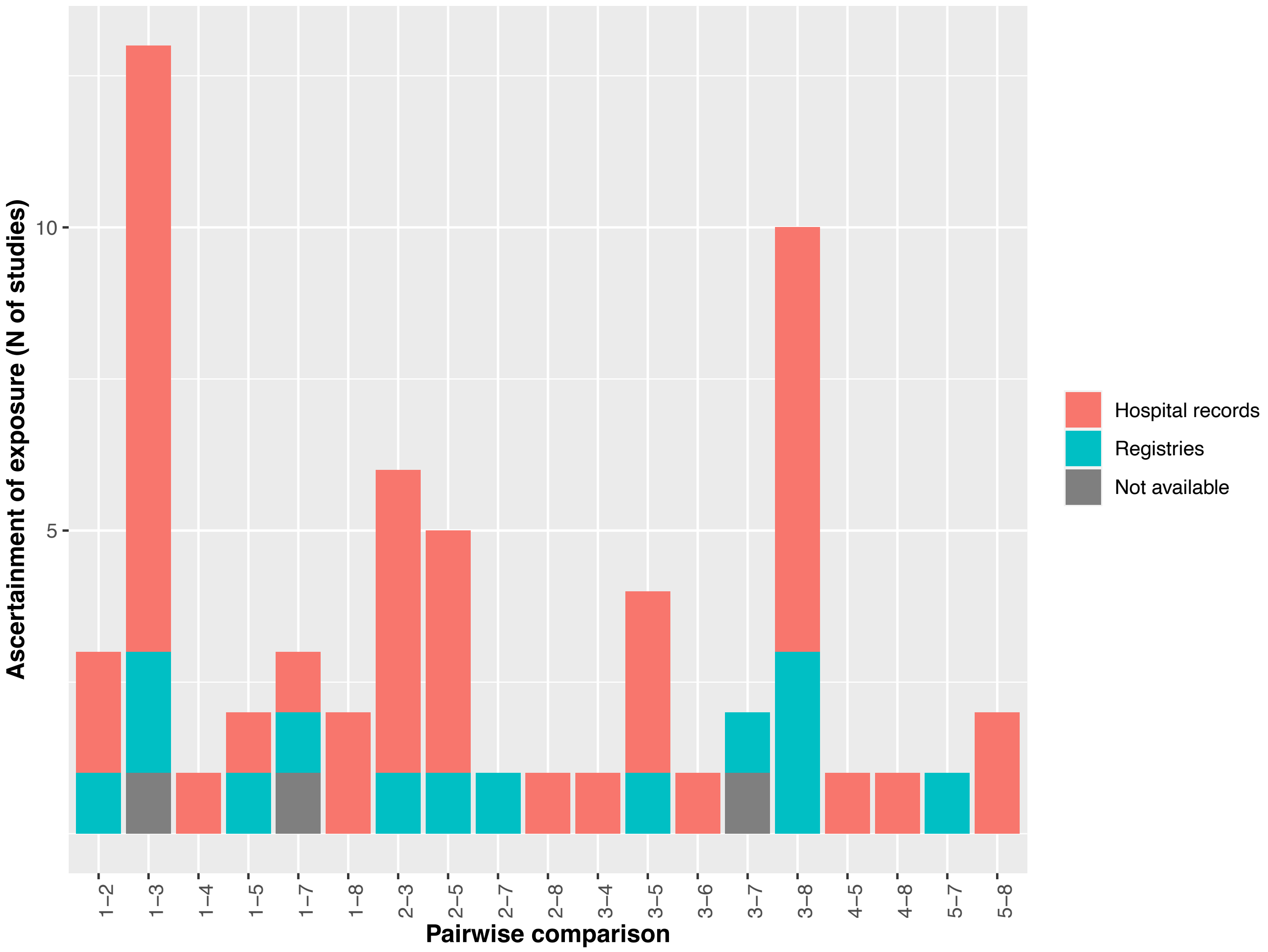

1: CKC; 2: LC; 3: LLETZ; 4: RD; 5: LA; 6: CC; 7: CT; 8: COLPO

Table 2.8.2.9: Risk of preterm birth in studies where ascertainment of exposure was through hospital records (N= 23 studies)

**CKC (P-score: 0·14)**	1·93 (1·06–3·53) (1·02–3·65)	1·77 (1·19–2·62) (1·16–2·68)	1·25 (0·79–1·97) (0·77–2·02)	2·32 (1·49–3·62) (1·46–3·71)	3·61 (0·08–157·15) (0·07–193·75)	0·96 (0·09–10·22) (0·08–11·66)	2·32 (1·56–3·46) (1·52–3·54)
0·52 (0·28–0·95) (0·27–0·98)	**LC (P-score: 0·59)**	0·91 (0·56–1·49) (0·55–1·53)	0·65 (0·38–1·10) (0·37–1·13)	1·20 (0·74–1·96) (0·72–2·01)	1·87 (0·04–82·20) (0·03–101·41)	0·50 (0·04–5·71) (0·04–6·54)	1·20 (0·75–1·93) (0·73–1·99)
0·57 (0·38–0·84) (0·37–0·86)	1·09 (0·67–1·78) (0·66–1·83)	**LLETZ (P-score: 0·49)**	0·71 (0·52–0·96) (0·51–0·98)	1·32 (1·00–1·74) (0·98–1·76)	2·04 (0·05–87·13) (0·04–107·30)	0·54 (0·05–5·98) (0·04–6·83)	1·31 (1·08–1·60) (1·07–1·62)
0·80 (0·51–1·26) (0·49–1·30)	1·55 (0·91–2·62) (0·89–2·70)	1·41 (1·04–1·92) (1·02–1·96)	**RD (P-score: 0·25)**	1·86 (1·38–2·50) (1·36–2·54)	2·89 (0·07–124·78) (0·05–153·77)	0·77 (0·07–8·55) (0·06–9·77)	1·86 (1·45–2·37) (1·43–2·41)
0·43 (0·28–0·67) (0·27–0·69)	0·83 (0·51–1·35) (0·50–1·39)	0·76 (0·58–1·00) (0·57–1·02)	0·54 (0·40–0·72) (0·39–0·73)	**LA (P-score: 0·78)**	1·55 (0·04–66·90) (0·03–82·44)	0·41 (0·04–4·58) (0·03–5·24)	1·00 (0·80–1·24) (0·79–1·26)
0·28 (0·01–12·09) (0·01–14·91)	0·54 (0·01–23·60) (0·01–29·12)	0·49 (0·01–20·92) (0·01–25·76)	0·35 (0·01–14·98) (0·01–18·46)	0·64 (0·01–27·80) (0·01–34·26)	**CC (P-score: 0·66)**	0·27 (0·00–22·91) (0·00–29·33)	0·64 (0·01–27·62) (0·01–34·02)
1·04 (0·10–11·09) (0·09–12·65)	2·01 (0·18–23·11) (0·15–26·47)	1·84 (0·17–20·25) (0·15–23·13)	1·30 (0·12–14·48) (0·10–16·55)	2·42 (0·22–26·86) (0·19–30·70)	3·76 (0·04–323·11) (0·03–413·65)	**CT (P-score: 0·32)**	2·42 (0·22–26·62) (0·19–30·41)
0·43 (0·29–0·64) (0·28–0·66)	0·83 (0·52–1·34) (0·50–1·38)	0·76 (0·62–0·93) (0·62–0·94)	0·54 (0·42–0·69) (0·42–0·70)	1·00 (0·80–1·25) (0·79–1·26)	1·55 (0·04–66·68) (0·03–82·14)	0·41 (0·04–4·56) (0·03–5·21)	**COLPO (P-score: 0·78)**

Heterogeneity: τ^2=0·00; I^2=0% (0–44)

Table 2.8.2.10: Risk of preterm birth in studies where ascertainment of exposure was through population-based registries (N=5 studies)

**CKC**<br>**(P-score: 0·20)**	1·04<br>(0·60–1·80)<br>(0·24–4·54)	1·65<br>(1·11–2·44)<br>(0·49–5·56)	1·56<br>(0·48–5·05)<br>(0·10–23·68)	1·46<br>(0·34–6·32)<br>(0·05–40·85)	2·39<br>(1·48–3·88)<br>(0·61–9·36)
0·96<br>(0·56–1·67)<br>(0·22–4·21)	**LC**<br>**(P-score: 0·23)**	1·59<br>(1·04–2·42)<br>(0·45–5·61)	1·50<br>(0·49–4·62)<br>(0·11–20·52)	1·40<br>(0·34–5·84)<br>(0·05–36·12)	2·31<br>(1·39–3·83)<br>(0·57–9·41)
0·61<br>(0·41–0·90)<br>(0·18–2·05)	0·63<br>(0·41–0·96)<br>(0·18–2·23)	**LLETZ**<br>**(P-score: 0·62)**	0·94<br>(0·31–2·91)<br>(0·07–12·90)	0·88<br>(0·21–3·68)<br>(0·03–22·72)	1·45<br>(1·10–1·93)<br>(0·51–4·17)
0·64<br>(0·20–2·09)<br>(0·04–9·79)	0·67<br>(0·22–2·06)<br>(0·05–9·15)	1·06<br>(0·34–3·26)<br>(0·08–14·46)	**LA**<br>**(P-score: 0·55)**	0·94<br>(0·16–5·48)<br>(0·02–49·80)	1·54<br>(0·48–4·91)<br>(0·10–22·59)
0·69<br>(0·16–2·98)<br>(0·02–19·29)	0·71<br>(0·17–2·98)<br>(0·03–18·40)	1·13<br>(0·27–4·71)<br>(0·04–29·08)	1·07<br>(0·18–6·26)<br>(0·02–56·87)	**CT**<br>**(P-score: 0·50)**	1·65<br>(0·38–7·04)<br>(0·06–44·82)
0·42<br>(0·26–0·68)<br>(0·11–1·63)	0·43<br>(0·26–0·72)<br>(0·11–1·77)	0·69<br>(0·52–0·91)<br>(0·24–1·97)	0·65<br>(0·20–2·07)<br>(0·04–9·53)	0·61<br>(0·14–2·60)<br>(0·02–16·55)	**COLPO**<br>**(P-score: 0·90)**

Heterogeneity: τ^2=0·04; I^2=66% (1–88)

Figure 2.8.2.6: Method of ascertainment of outcome across treatment comparisons

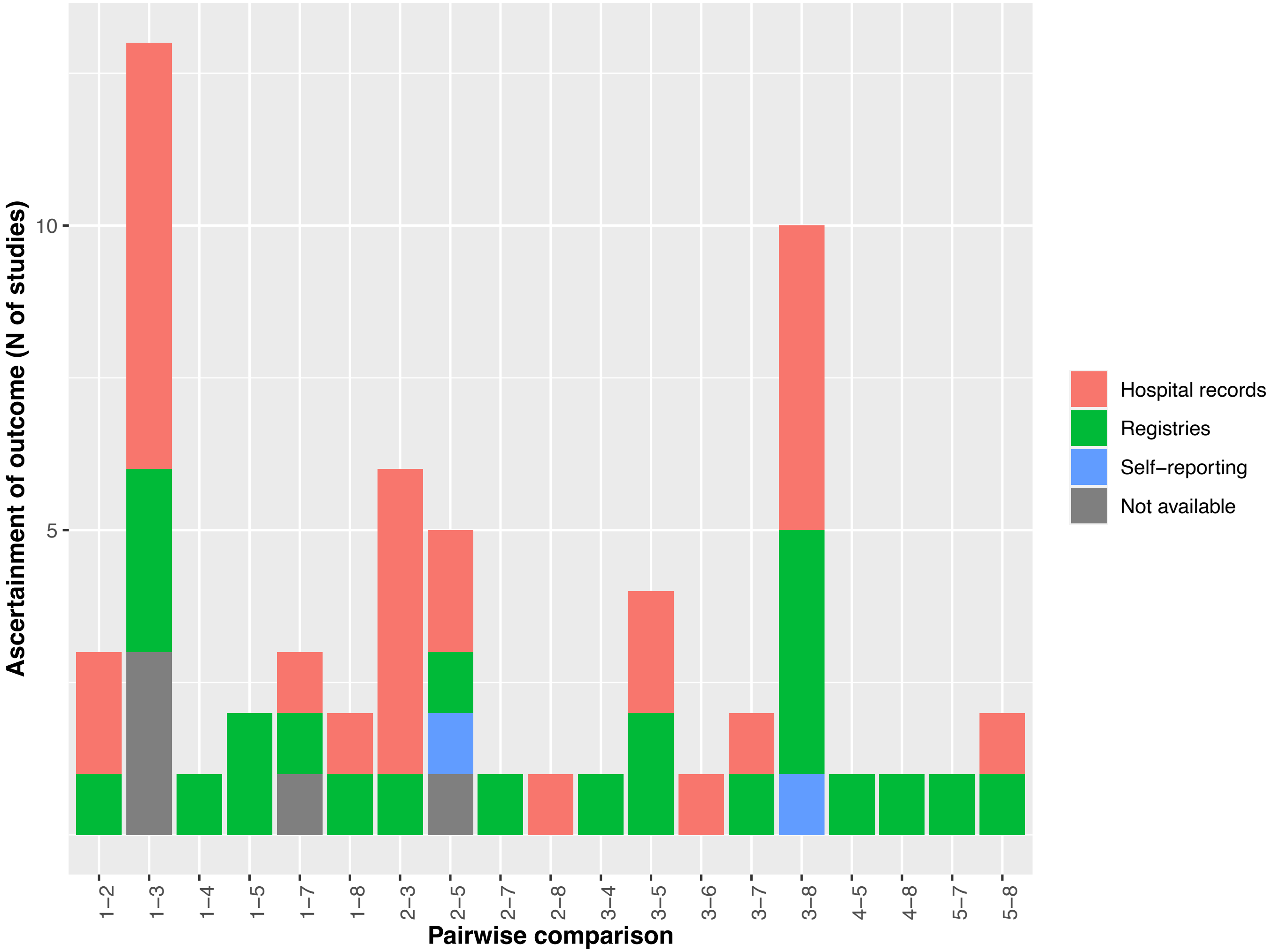

1: CKC; 2: LC; 3: LLETZ; 4: RD; 5: LA; 6: CC; 7: CT; 8: COLPO

Table 2.8.2.11: Risk of preterm birth in studies where ascertainment of outcome was through hospital records (N=16 studies)

**CKC (P-score: 0·04)**	2·18 (1·09–4·35) (1·03–4·63)	2·17 (1·35–3·49) (1·29–3·64)	3·56 (1·86–6·84) (1·75–7·24)	4·43 (0·10–194·73) (0·07–271·16)	10·62 (1·31–86·39) (1·09–103·78)	2·70 (1·63–4·47) (1·56–4·67)
0·46 (0·23–0·91) (0·22–0·97)	**LC (P-score: 0·36)**	0·99 (0·59–1·67) (0·57–1·74)	1·63 (0·90–2·95) (0·86–3·10)	2·03 (0·05–89·71) (0·03–124·98)	4·87 (0·58–41·11) (0·48–49·55)	1·24 (0·74–2·07) (0·70–2·17)
0·46 (0·29–0·74) (0·27–0·77)	1·01 (0·60–1·69) (0·57–1·77)	**LLETZ (P-score: 0·33)**	1·64 (1·04–2·60) (1·00–2·71)	2·04 (0·05–87·13) (0·03–121·01)	4·90 (0·62–38·86) (0·51–46·58)	1·24 (1·00–1·55) (0·98–1·58)
0·28 (0·15–0·54) (0·14–0·57)	0·61 (0·34–1·11) (0·32–1·16)	0·61 (0·38–0·96) (0·37–1·00)	**LA (P-score: 0·74)**	1·24 (0·03–54·55) (0·02–75·94)	2·98 (0·36–24·86) (0·30–29·93)	0·76 (0·48–1·19) (0·46–1·23)
0·23 (0·01–9·94) (0·00–13·85)	0·49 (0·01–21·81) (0·01–30·39)	0·49 (0·01–20·92) (0·01–29·05)	0·80 (0·02–35·35) (0·01–49·21)	**CC (P-score: 0·59)**	2·40 (0·03–174·66) (0·02–254·17)	0·61 (0·01–26·19) (0·01–36·39)
0·09 (0·01–0·77) (0·01–0·92)	0·21 (0·02–1·74) (0·02–2·09)	0·20 (0·03–1·62) (0·02–1·94)	0·34 (0·04–2·80) (0·03–3·37)	0·42 (0·01–30·34) (0·00–44·15)	**CT (P-score: 0·87)**	0·25 (0·03–2·04) (0·03–2·44)
0·37 (0·22–0·61) (0·21–0·64)	0·81 (0·48–1·36) (0·46–1·42)	0·80 (0·65–1·00) (0·63–1·02)	1·32 (0·84–2·07) (0·81–2·15)	1·64 (0·04–70·51) (0·03–97·97)	3·94 (0·49–31·57) (0·41–37·87)	**COLPO (P-score: 0·56)**

Heterogeneity: τ^2=0·00; I^2=0% (0–51)

Table 2.8.2.12: Risk of preterm birth in studies where ascertainment of outcome was through population-based registries (N=6 studies)

**CKC (P-score: 0·19)**	0·99<br>(0·63–1·56)<br>(0·48–2·04)	1·54<br>(1·13–2·11)<br>(0·87–2·75)	1·16<br>(0·71–1·89)<br>(0·54–2·47)	2·00<br>(1·24–3·21)<br>(0·95–4·21)	1·39<br>(0·33–5·84)<br>(0·20–9·55)	2·21<br>(1·54–3·17)<br>(1·18–4·13)
1·01<br>(0·64–1·59)<br>(0·49–2·07)	**LC (P-score: 0·18)**	1·56<br>(1·09–2·22)<br>(0·84–2·89)	1·16<br>(0·68–1·99)<br>(0·51–2·63)	2·01<br>(1·20–3·37)<br>(0·91–4·43)	1·40<br>(0·34–5·73)<br>(0·21–9·30)	2·23<br>(1·48–3·36)<br>(1·13–4·38)
0·65<br>(0·47–0·88)<br>(0·36–1·15)	0·64<br>(0·45–0·92)<br>(0·35–1·19)	**LLETZ (P-score: 0·59)**	0·75<br>(0·49–1·14)<br>(0·38–1·48)	1·29<br>(0·87–1·93)<br>(0·67–2·51)	0·90<br>(0·22–3·68)<br>(0·14–5·97)	1·43<br>(1·15–1·78)<br>(0·87–2·36)
0·87<br>(0·53–1·41)<br>(0·40–1·85)	0·86<br>(0·50–1·47)<br>(0·38–1·94)	1·34<br>(0·88–2·03)<br>(0·67–2·65)	**RD (P-score: 0·32)**	1·73<br>(1·12–2·67)<br>(0·86–3·48)	1·20<br>(0·28–5·21)<br>(0·17–8·58)	1·91<br>(1·30–2·81)<br>(1·00–3·66)
0·50<br>(0·31–0·80)<br>(0·24–1·05)	0·50<br>(0·30–0·83)<br>(0·23–1·09)	0·77<br>(0·52–1·15)<br>(0·40–1·50)	0·58<br>(0·37–0·89)<br>(0·29–1·17)	**LA (P-score: 0·81)**	0·70<br>(0·16–2·99)<br>(0·10–4·91)	1·11<br>(0·76–1·60)<br>(0·59–2·09)
0·72<br>(0·17–3·02)<br>(0·10–4·94)	0·71<br>(0·17–2·92)<br>(0·11–4·74)	1·11<br>(0·27–4·54)<br>(0·17–7·36)	0·83<br>(0·19–3·59)<br>(0·12–5·92)	1·44<br>(0·33–6·16)<br>(0·20–10·13)	**CT (P-score: 0·49)**	1·59<br>(0·38–6·59)<br>(0·24–10·73)
0·45<br>(0·32–0·65)<br>(0·24–0·84)	0·45<br>(0·30–0·68)<br>(0·23–0·88)	0·70<br>(0·56–0·87)<br>(0·42–1·15)	0·52<br>(0·36–0·77)<br>(0·27–1·00)	0·90<br>(0·62–1·31)<br>(0·48–1·71)	0·63<br>(0·15–2·61)<br>(0·09–4·25)	**COLPO (P-score: 0·91)**

Heterogeneity: τ^2=0·03; I^2=44% (0–77)

Figure 2.8.2.7: Level of income of country across treatment comparisons

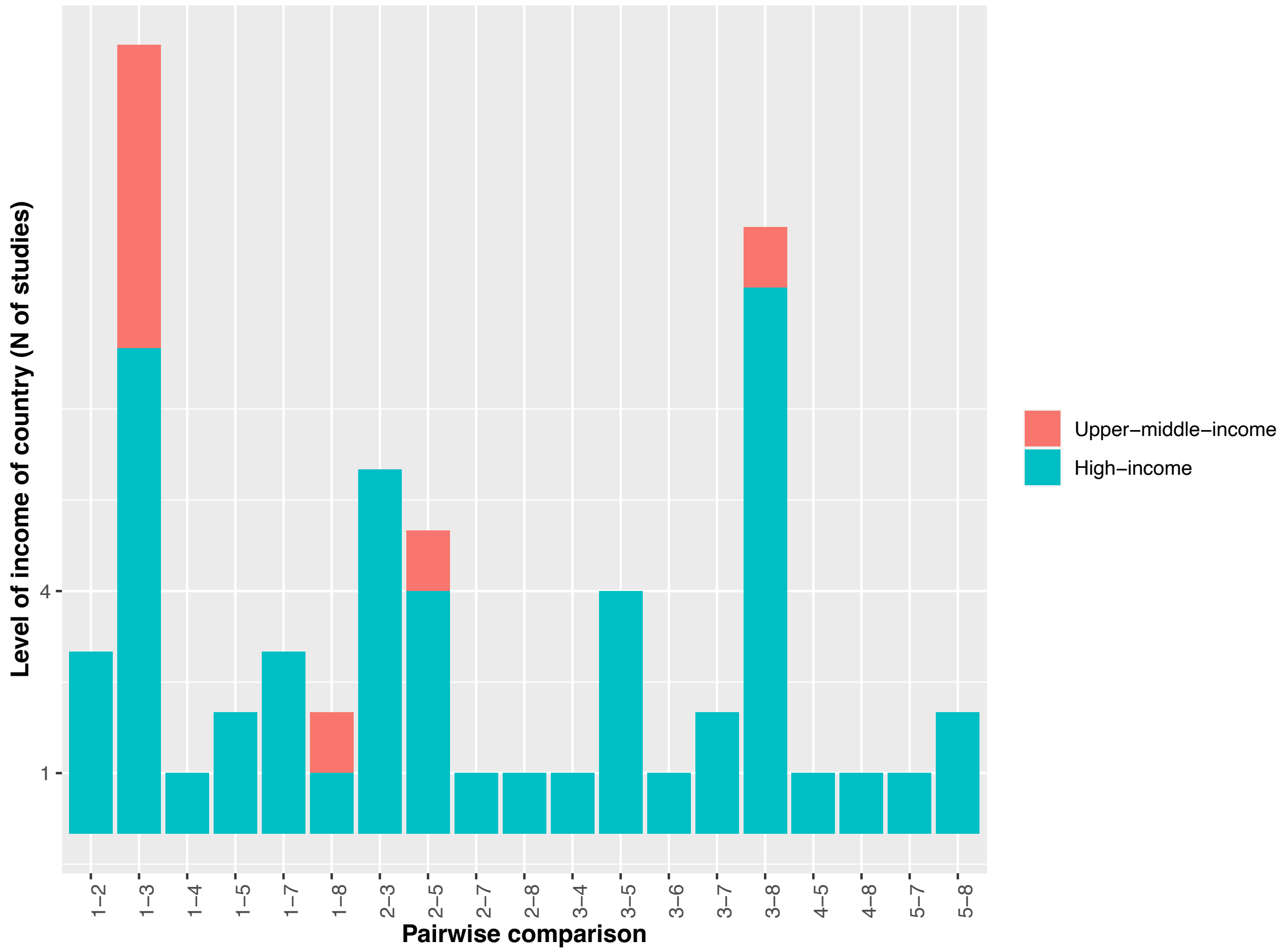

1: CKC; 2: LC; 3: LLETZ; 4: RD; 5: LA; 6: CC; 7: CT; 8: COLPO

Table 2.8.2.13: Risk of preterm birth in studies conducted in middle-income countries (N=5 studies)

**CKC (P-score: 0·02)**	2·06 (1·13–3·75) (0·78–5·45)	2·27 (1·01–5·13) (0·61–8·52)
0·48 (0·27–0·88) (0·18–1·28)	**LLETZ (P-score: 0·70)**	1·10 (0·48–2·51) (0·29–4·19)
0·44 (0·20–0·99) (0·12–1·65)	0·91 (0·40–2·06) (0·24–3·44)	**COLPO (P-score: 0·78)**

Heterogeneity: τ^2=0·00; I^2=0% (0–85)

Table 2.8.2.14: Risk of preterm birth in studies conducted in high-income countries (N=32 studies)

**CKC (P-score: 0·11)**	1·19 (0·80–1·75) (0·71–1·99)	1·59 (1·20–2·10) (1·03–2·45)	1·19 (0·75–1·89) (0·67–2·12)	2·19 (1·47–3·26) (1·29–3·70)	3·24 (0·07–141·65) (0·06–172·47)	2·15 (0·74–6·24) (0·68–6·87)	2·21 (1·61–3·03) (1·40–3·49)
0·84 (0·57–1·24) (0·50–1·41)	**LC (P-score: 0·25)**	1·34 (1·00–1·79) (0·86–2·08)	1·00 (0·63–1·61) (0·56–1·81)	1·84 (1·24–2·72) (1·10–3·10)	2·73 (0·06–119·38) (0·05–145·36)	1·81 (0·62–5·27) (0·57–5·80)	1·86 (1·35–2·57) (1·17–2·96)
0·63 (0·48–0·83) (0·41–0·97)	0·75 (0·56–1·00) (0·48–1·16)	**LLETZ (P-score: 0·51)**	0·75 (0·51–1·11) (0·45–1·26)	1·38 (1·01–1·88) (0·87–2·17)	2·04 (0·05–88·20) (0·04–107·34)	1·36 (0·47–3·87) (0·43–4·26)	1·39 (1·18–1·64) (0·97–2·00)
0·84 (0·53–1·33) (0·47–1·49)	1·00 (0·62–1·59) (0·55–1·79)	1·33 (0·90–1·97) (0·79–2·24)	**RD (P-score: 0·26)**	1·83 (1·22–2·75) (1·08–3·11)	2·72 (0·06–119·91) (0·05–146·06)	1·81 (0·59–5·51) (0·54–6·07)	1·85 (1·28–2·68) (1·12–3·06)
0·46 (0·31–0·68) (0·27–0·77)	0·54 (0·37–0·80) (0·32–0·91)	0·73 (0·53–0·99) (0·46–1·15)	0·55 (0·36–0·82) (0·32–0·93)	**LA (P-score: 0·77)**	1·48 (0·03–64·94) (0·03–79·08)	0·99 (0·33–2·92) (0·30–3·22)	1·01 (0·75–1·36) (0·65–1·58)
0·31 (0·01–13·46) (0·01–16·39)	0·37 (0·01–16·00) (0·01–19·49)	0·49 (0·01–21·18) (0·01–25·77)	0·37 (0·01–16·21) (0·01–19·75)	0·67 (0·02–29·51) (0·01–35·93)	**CC (P-score: 0·64)**	0·66 (0·01–33·14) (0·01–40·60)	0·68 (0·02–29·55) (0·01–35·96)
0·46 (0·16–1·34) (0·15–1·48)	0·55 (0·19–1·60) (0·17–1·76)	0·74 (0·26–2·11) (0·23–2·32)	0·55 (0·18–1·69) (0·16–1·86)	1·01 (0·34–3·01) (0·31–3·32)	1·51 (0·03–75·09) (0·02–91·99)	**CT (P-score: 0·68)**	1·03 (0·36–2·96) (0·32–3·26)
0·45 (0·33–0·62) (0·29–0·72)	0·54 (0·39–0·74) (0·34–0·85)	0·72 (0·61–0·85) (0·50–1·03)	0·54 (0·37–0·78) (0·33–0·89)	0·99 (0·74–1·33) (0·63–1·54)	1·47 (0·03–63·68) (0·03–77·52)	0·98 (0·34–2·82) (0·31–3·10)	**COLPO (P-score: 0·78)**

Heterogeneity: τ^2=0·02; I^2=25% (0–53)

Figure 2.8.2.8: Distribution of CIN2+ across treatment comparisons

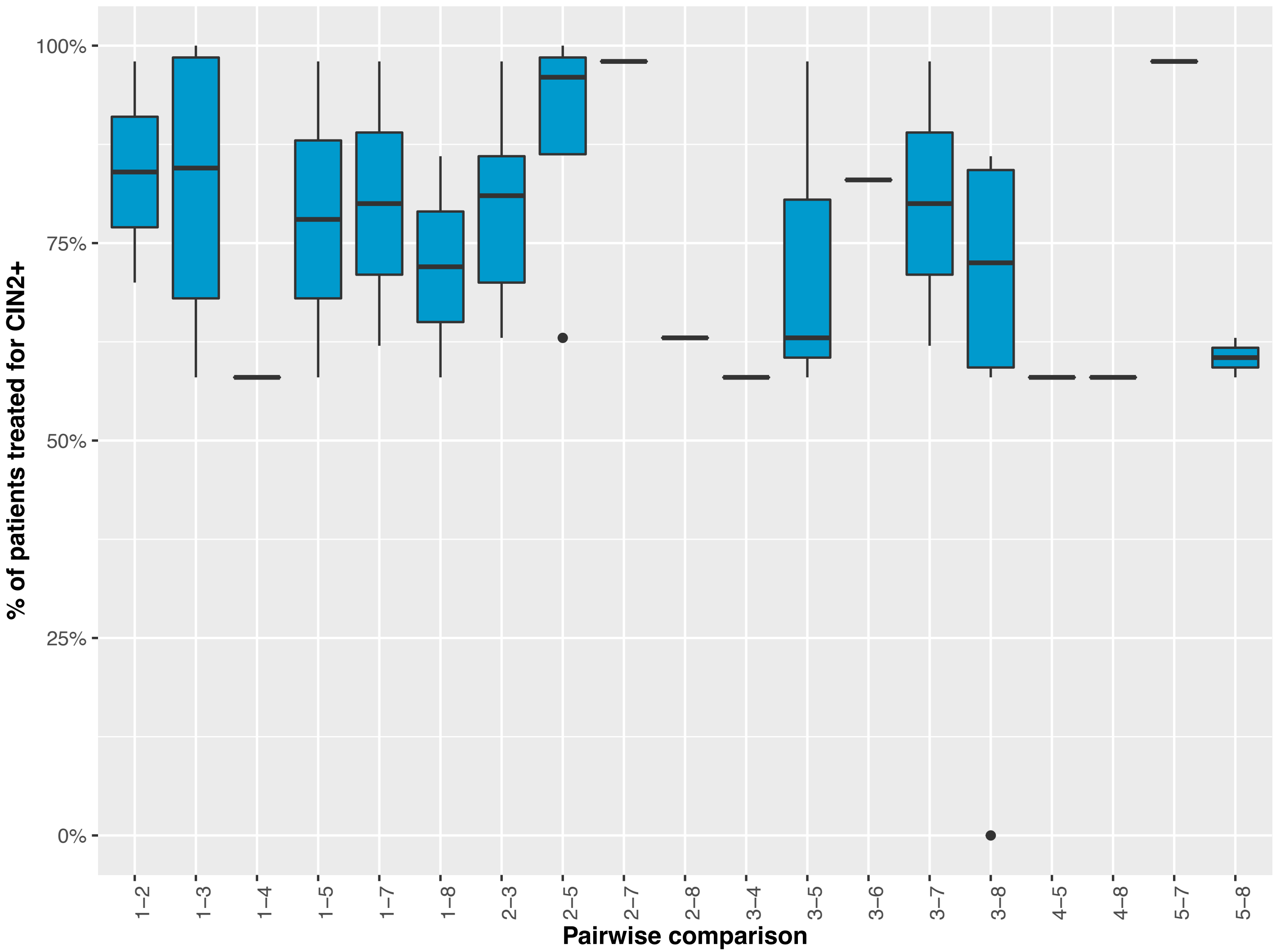

Median of the percentage of women treated for CIN2+ across studies: 83% (IQR=70–94); percentage of women treated for CIN2+ not reported in 12 studies

1: CKC; 2: LC; 3: LLETZ; 4: RD; 5: LA; 6: CC; 7: CT; 8: COLPO

Table 2.8.2.15: Risk of preterm birth in studies where ≥83% of women had been treated for CIN2+ (N=10 studies)

**CKC (P-score: 0·16)**	1·14 (0·69–1·87) (0·62–2·08)	1·82 (1·13–2·92) (1·02–3·22)	1·81 (0·66–4·97) (0·54–6·12)	3·71 (0·08–163·04) (0·04–356·10)	1·59 (0·37–6·79) (0·28–9·16)	2·98 (1·84–4·81) (1·67–5·32)
0·88 (0·53–1·44) (0·48–1·60)	**LC (P-score: 0·24)**	1·60 (1·32–1·93) (1·27–2·01)	1·59 (0·65–3·88) (0·54–4·66)	3·26 (0·08–139·66) (0·03–303·48)	1·40 (0·35–5·52) (0·27–7·33)	2·61 (2·10–3·24) (2·01–3·39)
0·55 (0·34–0·88) (0·31–0·98)	0·63 (0·52–0·76) (0·50–0·79)	**LLETZ (P-score: 0·57)**	1·00 (0·41–2·45) (0·34–2·95)	2·04 (0·05–87·13) (0·02–189·14)	0·88 (0·22–3·47) (0·17–4·61)	1·64 (1·48–1·82) (1·44–1·86)
0·55 (0·20–1·51) (0·16–1·86)	0·63 (0·26–1·53) (0·21–1·84)	1·00 (0·41–2·46) (0·34–2·96)	**LA (P-score: 0·55)**	2·05 (0·04–97·08) (0·02–215·39)	0·88 (0·17–4·48) (0·12–6·28)	1·64 (0·66–4·05) (0·55–4·89)
0·27 (0·01–11·87) (0·00–25·92)	0·31 (0·01–13·18) (0·00–28·63)	0·49 (0·01–20·92) (0·01–45·41)	0·49 (0·01–23·21) (0·00–51·49)	**CC (P-score: 0·66)**	0·43 (0·01–23·40) (0·00–53·43)	0·80 (0·02–34·32) (0·01–74·52)
0·63 (0·15–2·68) (0·11–3·61)	0·72 (0·18–2·83) (0·14–3·75)	1·14 (0·29–4·52) (0·22–6·00)	1·14 (0·22–5·81) (0·16–8·14)	2·33 (0·04–126·90) (0·02–289·71)	**CT (P-score: 0·47)**	1·87 (0·47–7·43) (0·35–9·87)
0·34 (0·21–0·54) (0·19–0·60)	0·38 (0·31–0·48) (0·29–0·50)	0·61 (0·55–0·68) (0·54–0·69)	0·61 (0·25–1·50) (0·20–1·81)	1·25 (0·03–53·27) (0·01–115·67)	0·54 (0·13–2·13) (0·10–2·83)	**COLPO (P-score: 0·85)**

Heterogeneity: τ^2=0·00; I^2=0% (0–65)

Table 2.8.2.16: Risk of preterm birth in studies where <83% of women had been treated for CIN2+ (N=7 studies)

**CKC**<br>**(P-score: 0·23)**	1·20<br>(0·56–2·57)<br>(0·48–3·01)	1·06<br>(0·59–1·90)<br>(0·53–2·14)	0·96<br>(0·53–1·74)<br>(0·47–1·96)	1·77<br>(0·98–3·17)<br>(0·87–3·57)	6·31<br>(0·77–52·06)<br>(0·49–80·48)	1·78<br>(1·02–3·12)<br>(0·91–3·50)
0·83<br>(0·39–1·79)<br>(0·33–2·09)	**LC**<br>**(P-score: 0·39)**	0·88<br>(0·50–1·56)<br>(0·44–1·76)	0·80<br>(0·44–1·45)<br>(0·39–1·64)	1·47<br>(0·83–2·60)<br>(0·74–2·92)	5·26<br>(0·62–44·92)<br>(0·40–69·96)	1·49<br>(0·86–2·58)<br>(0·76–2·89)
0·94<br>(0·53–1·68)<br>(0·47–1·90)	1·13<br>(0·64–2·00)<br>(0·57–2·25)	**LLETZ**<br>**(P-score: 0·28)**	0·90<br>(0·62–1·31)<br>(0·58–1·41)	1·66<br>(1·18–2·34)<br>(1·10–2·51)	5·95<br>(0·75–47·29)<br>(0·49–72·56)	1·68<br>(1·25–2·26)<br>(1·18–2·40)
1·04<br>(0·58–1·89)<br>(0·51–2·14)	1·25<br>(0·69–2·27)<br>(0·61–2·57)	1·11<br>(0·76–1·60)<br>(0·71–1·73)	**RD**<br>**(P-score: 0·17)**	1·84<br>(1·37–2·48)<br>(1·29–2·64)	6·59<br>(0·81–53·70)<br>(0·52–82·82)	1·86<br>(1·46–2·38)<br>(1·38–2·50)
0·57<br>(0·32–1·02)<br>(0·28–1·15)	0·68<br>(0·38–1·20)<br>(0·34–1·35)	0·60<br>(0·43–0·84)<br>(0·40–0·91)	0·54<br>(0·40–0·73)<br>(0·38–0·78)	**LA**<br>**(P-score: 0·74)**	3·57<br>(0·44–29·00)<br>(0·29–44·69)	1·01<br>(0·81–1·26)<br>(0·77–1·32)
0·16<br>(0·02–1·31)<br>(0·01–2·02)	0·19<br>(0·02–1·62)<br>(0·01–2·53)	0·17<br>(0·02–1·34)<br>(0·01–2·05)	0·15<br>(0·02–1·24)<br>(0·01–1·91)	0·28<br>(0·03–2·27)<br>(0·02–3·50)	**CT**<br>**(P-score: 0·93)**	0·28<br>(0·04–2·28)<br>(0·02–3·50)
0·56<br>(0·32–0·98)<br>(0·29–1·10)	0·67<br>(0·39–1·17)<br>(0·35–1·31)	0·60<br>(0·44–0·80)<br>(0·42–0·85)	0·54<br>(0·42–0·69)<br>(0·40–0·72)	0·99<br>(0·79–1·24)<br>(0·76–1·30)	3·54<br>(0·44–28·53)<br>(0·29–43·90)	**COLPO**<br>**(P-score: 0·76)**

Heterogeneity: τ^2=0·00; I^2=0% (0–65)

Figure 2.8.2.9: Distribution of CIN3+ across treatment comparisons

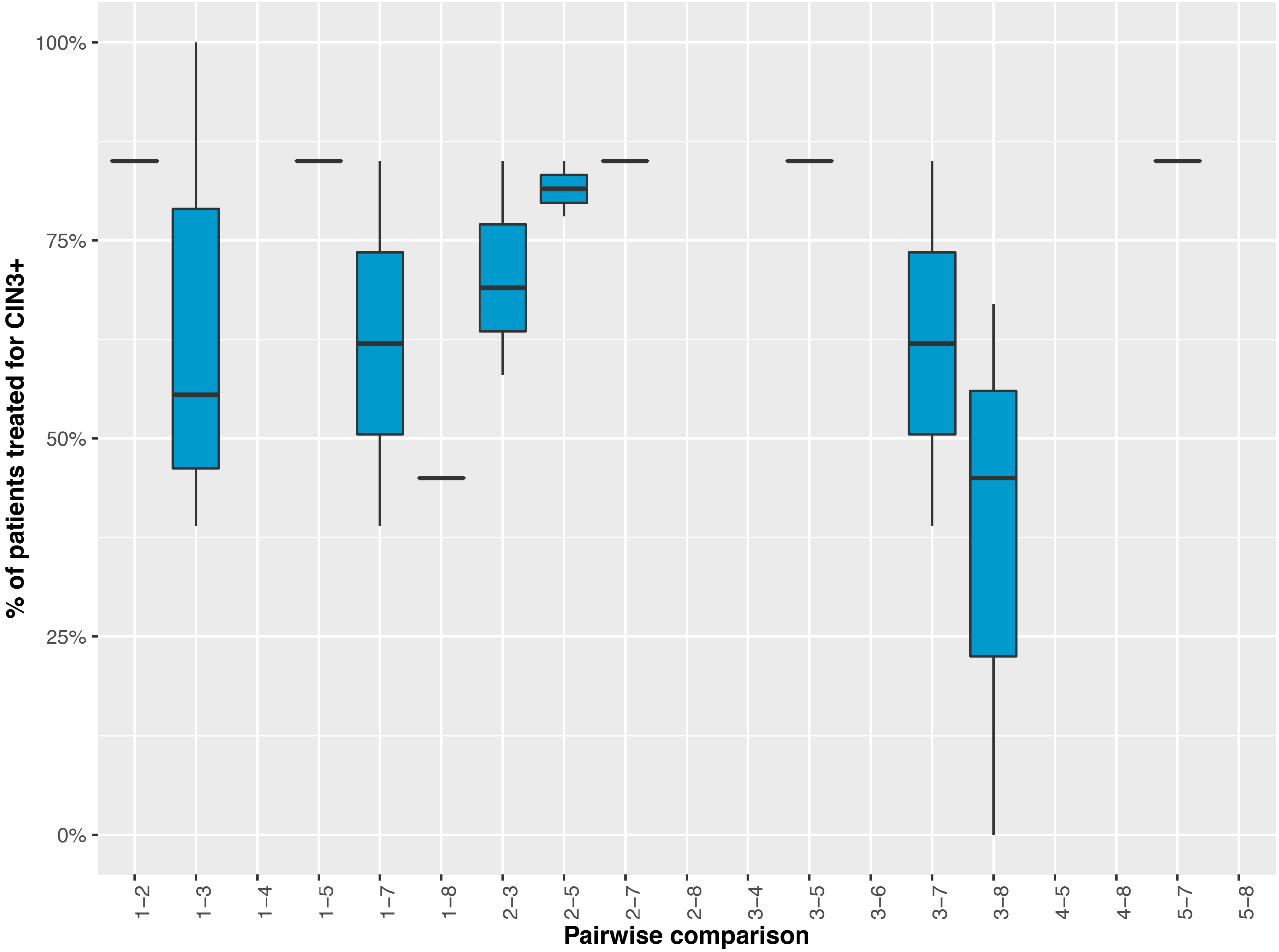

Median of the percentage of women treated for CIN3+ across studies: 61% (IQR=48–74); percentage of women treated for CIN3+ not reported in 18 studies

1: CKC; 2: LC; 3: LLETZ; 4: RD; 5: LA; 6: CC; 7: CT; 8: COLPO

Table 2.8.2.17: Risk of preterm birth in studies where ≥61% of women had been treated for CIN3+ (N=6 studies)

**CKC (P-score: 0·26)**	0·98 (0·47–2·02) (0·30–3·17)	1·55 (0·75–3·19) (0·48–5·00)	1·35 (0·39–4·63) (0·18–9·99)	1·36 (0·29–6·39) (0·11–16·72)	2·55 (1·23–5·29) (0·78–8·34)
1·02 (0·50–2·11) (0·32–3·32)	**LC (P-score: 0·21)**	1·58 (1·31–1·92) (1·16–2·16)	1·38 (0·50–3·80) (0·27–7·13)	1·40 (0·35–5·51) (0·15–12·99)	2·61 (2·10–3·25) (1·83–3·72)
0·65 (0·31–1·33) (0·20–2·08)	0·63 (0·52–0·76) (0·46–0·86)	**LLETZ (P-score: 0·61)**	0·87 (0·32–2·40) (0·17–4·53)	0·88 (0·22–3·48) (0·09–8·22)	1·65 (1·48–1·83) (1·39–1·96)
0·74 (0·22–2·53) (0·10–5·46)	0·72 (0·26–1·98) (0·14–3·73)	1·15 (0·42–3·16) (0·22–5·94)	**LA (P-score: 0·48)**	1·01 (0·18–5·50) (0·06–15·85)	1·89 (0·68–5·23) (0·36–9·88)
0·73 (0·16–3·43) (0·06–8·98)	0·72 (0·18–2·83) (0·08–6·67)	1·14 (0·29–4·49) (0·12–10·60)	0·99 (0·18–5·41) (0·06–15·58)	**CT (P-score: 0·49)**	1·87 (0·47–7·44) (0·20–17·59)
0·39 (0·19–0·81) (0·12–1·28)	0·38 (0·31–0·48) (0·27–0·55)	0·61 (0·55–0·67) (0·51–0·72)	0·53 (0·19–1·47) (0·10–2·77)	0·53 (0·13–2·12) (0·06–5·02)	**COLPO (P-score: 0·94)**

Heterogeneity: τ^2=0·00; I^2=0% (0–79)

Table 2.8.2.18: Risk of preterm birth in studies where <61% of women had been treated for CIN3+ (N=5 studies)

**CKC (P-score: 0·05)**	3·67 (0·54–25·05) (0·05–248·92)	1·64 (0·82–3·29) (0·36–7·55)	8·67 (1·03–72·88) (0·08–927·71)	2·58 (1·24–5·39) (0·51–12·99)
0·27 (0·04–1·86) (0·00–18·48)	**LC (P-score: 0·66)**	0·45 (0·07–2·68) (0·01–22·82)	2·36 (0·15–36·67) (0·01–971·59)	0·70 (0·11–4·55) (0·01–42·39)
0·61 (0·30–1·22) (0·13–2·80)	2·23 (0·37–13·39) (0·04–113·88)	**LLETZ (P-score: 0·30)**	5·28 (0·66–42·10) (0·06–503·42)	1·57 (0·93–2·67) (0·49–5·02)
0·12 (0·01–0·97) (0·00–12·33)	0·42 (0·03–6·56) (0·00–173·88)	0·19 (0·02–1·51) (0·00–18·05)	**CT (P-score: 0·88)**	0·30 (0·04–2·50) (0·00–31·80)
0·39 (0·19–0·81) (0·08–1·95)	1·42 (0·22–9·21) (0·02–85·78)	0·64 (0·38–1·08) (0·20–2·03)	3·36 (0·40–28·25) (0·03–359·55)	**COLPO (P-score: 0·61)**

Heterogeneity: τ^2=0·00; I^2=0% (0–85)

Figure 2.8.2.10: Distribution of AIS across treatment comparisons

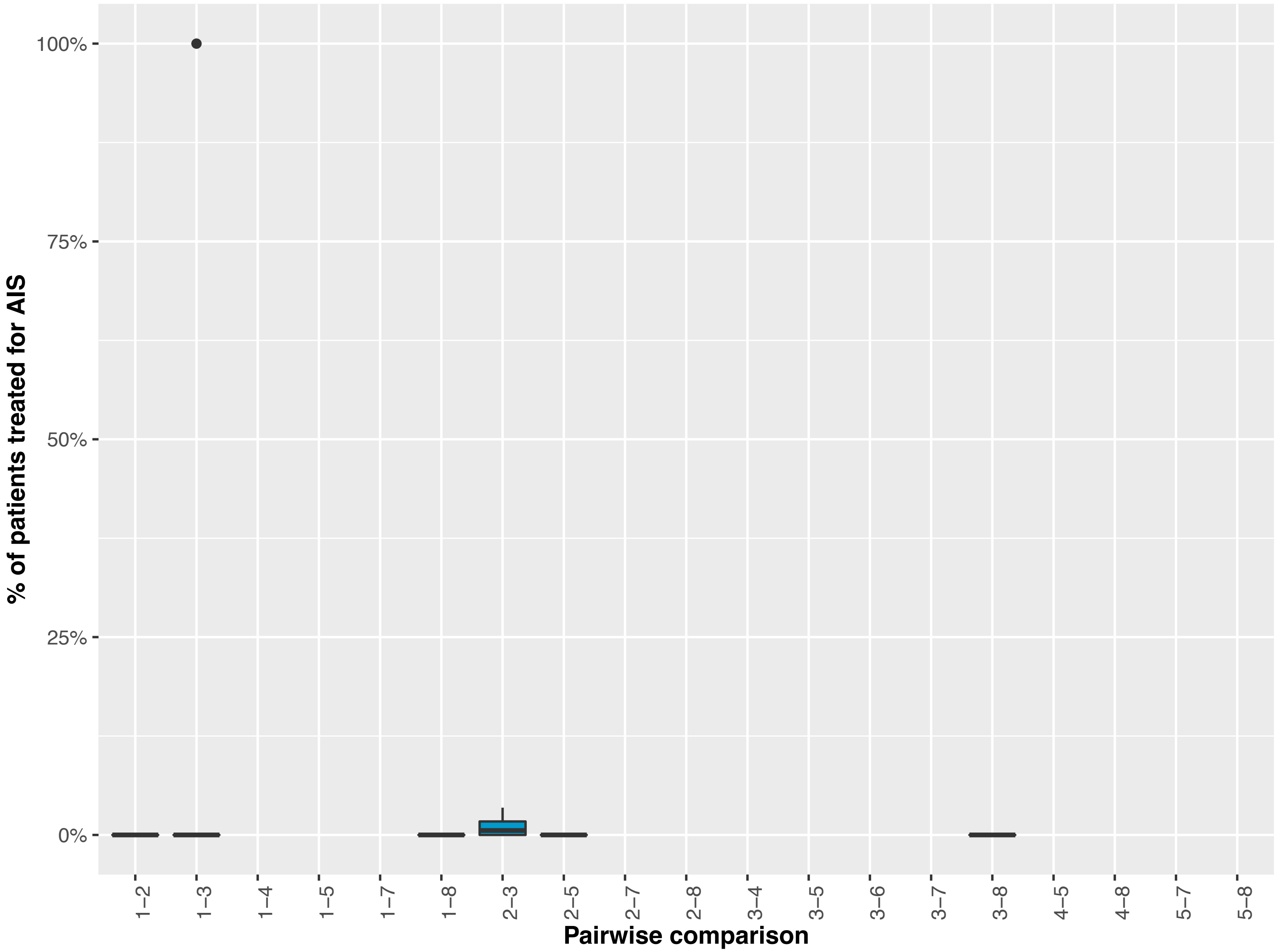

Median of the percentage of women treated for AIS across studies: 0% (IQR=0–0); percentage of women treated for AIS not reported in 17 studies

1: CKC; 2: LC; 3: LLETZ; 4: RD; 5: LA; 6: CC; 7: CT; 8: COLPO

Table 2.8.2.19: Risk of preterm birth in studies where >0% of women had been treated for AIS (N=3 studies)

**CKC<br>(P-score: 0·42)**	1·90<br>(0·06–57·42)<br>(NA, NA)	1·10<br>(0·05–25·78)<br>(NA, NA)
0·53<br>(0·02–15·97)<br>(NA, NA)	**LC<br>(P-score: 0·72)**	0·58<br>(0·16–2·13)<br>(NA, NA)
0·91<br>(0·04–21·24)<br>(NA, NA)	1·72<br>(0·47–6·32)<br>(NA, NA)	**LLETZ<br>(P-score: 0·37)**

Heterogeneity: τ^2=0·00; I^2=0% (95% CI NA)

Table 2.8.2.20: Risk of preterm birth in studies where 0% of women had been treated for AIS (N=9 studies)

**CKC (P-score: 0·08)**	2·70 (0·29–25·02) (0·18–39·62)	1·65 (0·97–2·81) (0·87–3·13)	7·02 (0·38–130·00) (0·21–237·46)	2·85 (1·53–5·32) (1·35–6·05)
0·37 (0·04–3·43) (0·03–5·43)	**LC (P-score: 0·53)**	0·61 (0·07–5·55) (0·04–8·74)	2·60 (0·39–17·16) (0·27–25·33)	1·06 (0·11–9·96) (0·07–15·83)
0·61 (0·36–1·03) (0·32–1·15)	1·64 (0·18–14·84) (0·11–23·39)	**LLETZ (P-score: 0·37)**	4·25 (0·23–77·47) (0·13–141·04)	1·73 (1·10–2·72) (1·00–2·99)
0·14 (0·01–2·64) (0·00–4·81)	0·38 (0·06–2·54) (0·04–3·75)	0·24 (0·01–4·28) (0·01–7·80)	**LA (P-score: 0·83)**	0·41 (0·02–7·62) (0·01–13·97)
0·35 (0·19–0·65) (0·17–0·74)	0·95 (0·10–8·92) (0·06–14·18)	0·58 (0·37–0·91) (0·33–1·00)	2·46 (0·13–46·17) (0·07–84·57)	**COLPO (P-score: 0·70)**

Heterogeneity: τ^2=0·00; I^2=0% (0–65)

Figure 2.8.2.11: Distribution of cervical cancer across treatment comparisons

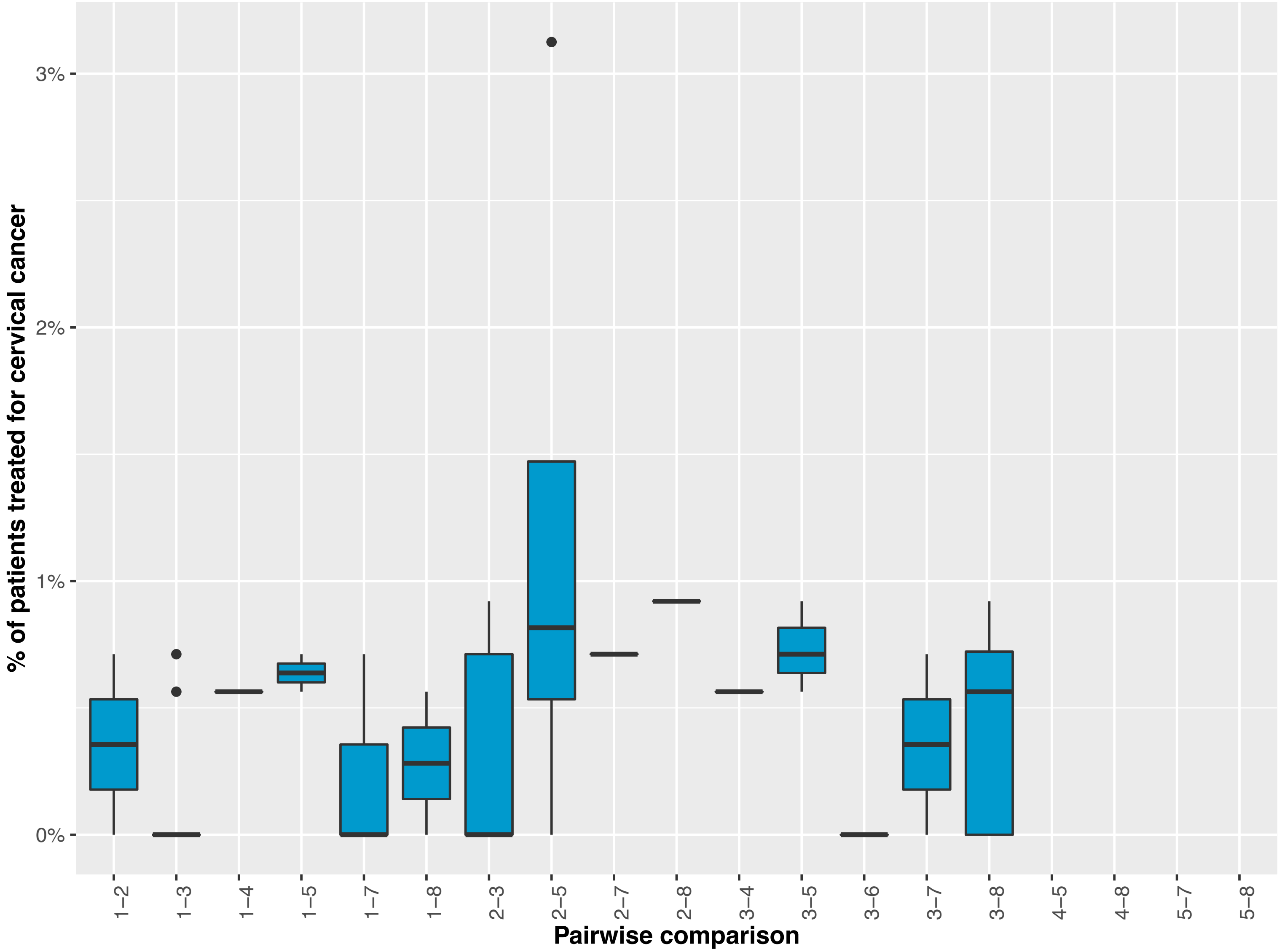

Median of the percentage of women treated for cancer across studies: 0% (IQR=0–0); percentage of women treated for cancer not reported in 10 studies

1: CKC; 2: LC; 3: LLETZ; 4: RD; 5: LA; 6: CC; 7: CT; 8: COLPO

Table 2.8.2.21: Risk of preterm birth in studies where >0% of women had been treated for cervical cancer (N=5 studies)

**CKC<br>(P-score: 0·25)**	0·94<br>(0·53–1·67)<br>(0·45–1·96)	1·41<br>(0·80–2·47)<br>(0·68–2·89)	1·05<br>(0·58–1·89)<br>(0·50–2·23)	1·91<br>(1·08–3·38)<br>(0·92–3·96)	1·29<br>(0·29–5·66)<br>(0·20–8·24)	1·95<br>(1·13–3·38)<br>(0·97–3·95)
1·06<br>(0·60–1·88)<br>(0·51–2·21)	**LC<br>(P-score: 0·18)**	1·49<br>(1·20–1·85)<br>(1·09–2·04)	1·12<br>(0·73–1·70)<br>(0·65–1·93)	2·03<br>(1·40–2·93)<br>(1·25–3·30)	1·37<br>(0·34–5·43)<br>(0·24–7·70)	2·07<br>(1·47–2·92)<br>(1·31–3·28)
0·71<br>(0·40–1·25)<br>(0·35–1·46)	0·67<br>(0·54–0·83)<br>(0·49–0·92)	**LLETZ<br>(P-score: 0·57)**	0·75<br>(0·51–1·11)<br>(0·45–1·25)	1·36<br>(0·97–1·91)<br>(0·86–2·14)	0·92<br>(0·23–3·64)<br>(0·16–5·17)	1·39<br>(1·02–1·89)<br>(0·91–2·11)
0·95<br>(0·53–1·71)<br>(0·45–2·02)	0·90<br>(0·59–1·36)<br>(0·52–1·55)	1·34<br>(0·90–1·98)<br>(0·80–2·23)	**RD<br>(P-score: 0·29)**	1·82<br>(1·32–2·50)<br>(1·18–2·79)	1·22<br>(0·29–5·11)<br>(0·20–7·34)	1·86<br>(1·41–2·44)<br>(1·27–2·71)
0·52<br>(0·30–0·93)<br>(0·25–1·09)	0·49<br>(0·34–0·71)<br>(0·30–0·80)	0·74<br>(0·52–1·03)<br>(0·47–1·16)	0·55<br>(0·40–0·76)<br>(0·36–0·85)	**LA<br>(P-score: 0·85)**	0·67<br>(0·16–2·78)<br>(0·11–3·97)	1·02<br>(0·80–1·30)<br>(0·73–1·44)
0·78<br>(0·18–3·41)<br>(0·12–4·97)	0·73<br>(0·18–2·91)<br>(0·13–4·13)	1·09<br>(0·27–4·34)<br>(0·19–6·17)	0·82<br>(0·20–3·41)<br>(0·14–4·90)	1·48<br>(0·36–6·11)<br>(0·25–8·74)	**CT<br>(P-score: 0·49)**	1·52<br>(0·37–6·21)<br>(0·26–8·87)
0·51<br>(0·30–0·89)<br>(0·25–1·04)	0·48<br>(0·34–0·68)<br>(0·31–0·76)	0·72<br>(0·53–0·98)<br>(0·47–1·10)	0·54<br>(0·41–0·71)<br>(0·37–0·79)	0·98<br>(0·77–1·25)<br>(0·69–1·38)	0·66<br>(0·16–2·70)<br>(0·11–3·86)	**COLPO<br>(P-score: 0·88)**

Heterogeneity: τ^2=0·00; I^2=4% (0–69)

Table 2.8.2.22: Risk of preterm birth in studies where 0% of women had been treated for cervical cancer (N=14 studies)

**CKC (P-score: 0·25)**	2.39<br>(0.67–8.60)<br>(0.56–10.24)	1.58<br>(0.98–2.56)<br>(0.92–2.73)	6.23<br>(0.64–60.83)<br>(0.47–83.09)	3.23<br>(0.07–142.03)<br>(0.04–238.39)	3.29<br>(0.69–15.81)<br>(0.55–19.60)	2.50<br>(1.34–4.68)<br>(1.23–5.10)
0.42<br>(0.12–1.50)<br>(0.10–1.79)	**LC (P-score: 0·25)**	0.66<br>(0.20–2.18)<br>(0.17–2.57)	2.60<br>(0.39–17.16)<br>(0.30–22.21)	1.35<br>(0.03–69.29)<br>(0.02–118.79)	1.37<br>(0.19–9.95)<br>(0.14–13.04)	1.05<br>(0.28–3.84)<br>(0.24–4.59)
0.63<br>(0.39–1.02)<br>(0.37–1.09)	1.51<br>(0.46–5.01)<br>(0.39–5.90)	**LLETZ (P-score: 0·25)**	3.94<br>(0.42–36.78)<br>(0.31–49.94)	2.04<br>(0.05–87.13)<br>(0.03–145.63)	2.08<br>(0.43–10.13)<br>(0.34–12.58)	1.58<br>(0.95–2.66)<br>(0.88–2.85)
0.16<br>(0.02–1.57)<br>(0.01–2.14)	0.38<br>(0.06–2.54)<br>(0.05–3.29)	0.25<br>(0.03–2.37)<br>(0.02–3.22)	**LA (P-score: 0·25)**	0.52<br>(0.01–40.91)<br>(0.00–74.37)	0.53<br>(0.03–8.14)<br>(0.02–11.84)	0.40<br>(0.04–3.98)<br>(0.03–5.45)
0.31<br>(0.01–13.64)<br>(0.00–22.90)	0.74<br>(0.01–38.17)<br>(0.01–65.44)	0.49<br>(0.01–20.92)<br>(0.01–34.97)	1.93<br>(0.02–152.33)<br>(0.01–276.94)	**CC (P-score: 0·25)**	1.02<br>(0.02–59.97)<br>(0.01–104.71)	0.78<br>(0.02–34.34)<br>(0.01–57.67)
0.30<br>(0.06–1.46)<br>(0.05–1.81)	0.73<br>(0.10–5.27)<br>(0.08–6.90)	0.48<br>(0.10–2.34)<br>(0.08–2.90)	1.89<br>(0.12–29.14)<br>(0.08–42.36)	0.98<br>(0.02–57.61)<br>(0.01–100.60)	**CT (P-score: 0·25)**	0.76<br>(0.15–3.94)<br>(0.12–4.94)
0.40<br>(0.21–0.75)<br>(0.20–0.81)	0.96<br>(0.26–3.51)<br>(0.22–4.19)	0.63<br>(0.38–1.06)<br>(0.35–1.14)	2.49<br>(0.25–24.59)<br>(0.18–33.65)	1.29<br>(0.03–56.98)<br>(0.02–95.69)	1.31<br>(0.25–6.81)<br>(0.20–8.53)	**COLPO (P-score: 0·25)**

Heterogeneity: τ^2=0·00; I^2=0% (0–58)

Table 2.8.2.23: Sensitivity analysis for risk of preterm birth: excluding non-randomised studies (N=2 studies)

**CKC**<br>**(P-score: 0·23)**	5·83<br>(0·15–224·59)<br>(NA, NA)	1·59<br>(0·30–8·37)<br>(NA, NA)
0·17<br>(0·00–6·61)<br>(NA, NA)	**LC**<br>**(P-score: 0·80)**	0·27<br>(0·01–9·12)<br>(NA, NA)
0·63<br>(0·12–3·29)<br>(NA, NA)	3·66<br>(0·11–122·00)<br>(NA, NA)	**LLETZ**<br>**(P-score: 0·47)**

Heterogeneity: τ^2=0·64; I^2=29% (95% CI NA)

Table 2.8.2.24: Sensitivity analysis for risk of preterm birth: excluding non-randomised studies at high risk of bias (N=15 studies)

**CKC**<br>**(P-score: 0·12)**	1·17<br>(0·80–1·70)<br>(0·71–1·91)	1·62<br>(1·25–2·11)<br>(1·08–2·43)	1·19<br>(0·77–1·84)<br>(0·69–2·07)	2·19<br>(1·49–3·21)<br>(1·32–3·62)	1·54<br>(0·37–6·36)<br>(0·33–7·24)	2·21<br>(1·65–2·96)<br>(1·44–3·39)
0·86<br>(0·59–1·25)<br>(0·52–1·41)	**LC**<br>**(P-score: 0·27)**	1·39<br>(1·05–1·85)<br>(0·91–2·12)	1·02<br>(0·65–1·61)<br>(0·58–1·81)	1·88<br>(1·27–2·77)<br>(1·13–3·12)	1·32<br>(0·33–5·34)<br>(0·29–6·08)	1·90<br>(1·39–2·59)<br>(1·22–2·95)
0·62<br>(0·47–0·80)<br>(0·41–0·92)	0·72<br>(0·54–0·95)<br>(0·47–1·10)	**LLETZ**<br>**(P-score: 0·58)**	0·74<br>(0·51–1·07)<br>(0·45–1·21)	1·35<br>(1·00–1·83)<br>(0·87–2·09)	0·95<br>(0·23–3·84)<br>(0·21–4·37)	1·36<br>(1·16–1·60)<br>(0·97–1·91)
0·84<br>(0·54–1·29)<br>(0·48–1·45)	0·98<br>(0·62–1·54)<br>(0·55–1·72)	1·36<br>(0·94–1·97)<br>(0·83–2·22)	**RD**<br>**(P-score: 0·29)**	1·83<br>(1·25–2·70)<br>(1·10–3·04)	1·29<br>(0·30–5·45)<br>(0·27–6·22)	1·85<br>(1·30–2·63)<br>(1·15–2·98)
0·46<br>(0·31–0·67)<br>(0·28–0·76)	0·53<br>(0·36–0·79)<br>(0·32–0·89)	0·74<br>(0·55–1·00)<br>(0·48–1·15)	0·55<br>(0·37–0·80)<br>(0·33–0·91)	**LA**<br>**(P-score: 0·86)**	0·70<br>(0·17–2·92)<br>(0·15–3·33)	1·01<br>(0·76–1·34)<br>(0·66–1·54)
0·65<br>(0·16–2·69)<br>(0·14–3·06)	0·76<br>(0·19–3·07)<br>(0·16–3·49)	1·05<br>(0·26–4·27)<br>(0·23–4·85)	0·78<br>(0·18–3·29)<br>(0·16–3·75)	1·42<br>(0·34–5·92)<br>(0·30–6·74)	**CT**<br>**(P-score: 0·52)**	1·44<br>(0·35–5·85)<br>(0·31–6·66)
0·45<br>(0·34–0·61)<br>(0·29–0·69)	0·53<br>(0·39–0·72)<br>(0·34–0·82)	0·73<br>(0·63–0·86)<br>(0·52–1·03)	0·54<br>(0·38–0·77)<br>(0·34–0·87)	0·99<br>(0·74–1·32)<br>(0·65–1·51)	0·70<br>(0·17–2·84)<br>(0·15–3·23)	**COLPO**<br>**(P-score: 0·87)**

Heterogeneity: τ^2=0·02; I^2=27% (0–58)

Table 2.8.2.25: Sensitivity analysis for risk of preterm birth: including only studies reporting both spontaneous and iatrogenic preterm birth (N=23 studies)

**CKC**<br>**(P-score: 0·09)**	1·26<br>(0·85–1·85)<br>(0·83–1·89)	1·88<br>(1·31–2·68)<br>(1·29–2·74)	1·23<br>(0·80–1·89)<br>(0·78–1·93)	2·23<br>(1·48–3·35)<br>(1·45–3·43)	1·48<br>(0·44–4·98)<br>(0·41–5·29)	2·29<br>(1·59–3·30)<br>(1·56–3·37)
0·80<br>(0·54–1·17)<br>(0·53–1·20)	**LC**<br>**(P-score: 0·30)**	1·49<br>(1·25–1·79)<br>(1·23–1·82)	0·98<br>(0·71–1·36)<br>(0·69–1·38)	1·77<br>(1·33–2·36)<br>(1·31–2·40)	1·18<br>(0·36–3·88)<br>(0·34–4·12)	1·83<br>(1·46–2·28)<br>(1·44–2·32)
0·53<br>(0·37–0·76)<br>(0·37–0·78)	0·67<br>(0·56–0·80)<br>(0·55–0·81)	**LLETZ**<br>**(P-score: 0·62)**	0·66<br>(0·49–0·87)<br>(0·49–0·88)	1·19<br>(0·93–1·51)<br>(0·92–1·54)	0·79<br>(0·24–2·60)<br>(0·22–2·75)	1·22<br>(1·06–1·41)<br>(1·04–1·43)
0·81<br>(0·53–1·25)<br>(0·52–1·28)	1·02<br>(0·74–1·41)<br>(0·72–1·44)	1·53<br>(1·15–2·02)<br>(1·13–2·06)	**RD**<br>**(P-score: 0·28)**	1·81<br>(1·35–2·44)<br>(1·32–2·48)	1·20<br>(0·35–4·08)<br>(0·33–4·33)	1·87<br>(1·46–2·39)<br>(1·43–2·43)
0·45<br>(0·30–0·68)<br>(0·29–0·69)	0·56<br>(0·42–0·75)<br>(0·42–0·76)	0·84<br>(0·66–1·07)<br>(0·65–1·09)	0·55<br>(0·41–0·74)<br>(0·40–0·76)	**LA**<br>**(P-score: 0·84)**	0·66<br>(0·20–2·23)<br>(0·19–2·37)	1·03<br>(0·83–1·28)<br>(0·82–1·30)
0·68<br>(0·20–2·29)<br>(0·19–2·43)	0·85<br>(0·26–2·81)<br>(0·24–2·98)	1·27<br>(0·39–4·19)<br>(0·36–4·44)	0·83<br>(0·25–2·83)<br>(0·23–3·01)	1·51<br>(0·45–5·07)<br>(0·42–5·39)	**CT**<br>**(P-score: 0·47)**	1·55<br>(0·47–5·16)<br>(0·44–5·47)
0·44<br>(0·30–0·63)<br>(0·30–0·64)	0·55<br>(0·44–0·68)<br>(0·43–0·69)	0·82<br>(0·71–0·94)<br>(0·70–0·96)	0·54<br>(0·42–0·69)<br>(0·41–0·70)	0·97<br>(0·78–1·20)<br>(0·77–1·22)	0·64<br>(0·19–2·14)<br>(0·18–2·27)	**COLPO**<br>**(P-score: 0·89)**

Heterogeneity: τ^2=0·00; I^2=0% (0–42)

Summary Table

Table 2.8.2.26: Subgroup and sensitivity analyses for risk of preterm birth after CIN treatments

Analysis / Comparison	N studies	CKC vs COLPO	LC vs COLPO	LLETZ vs COLPO	RD vs COLPO	LA vs COLPO	CC vs COLPO	CT vs COLPO
Main analysis	29	2·27 (1·70–3·02)	1·77 (1·29–2·43)	1·37 (1·16–1·62)	1·88 (1·30–2·72)	1·05 (0·78–1·41)	0·67 (0·02–29·15)	1·01 (0·35–2·92)
Studies published in or after 2011	15	2·06 (1·62–2·62)	1·97 (1·53–2·53)	1·26 (1·07–1·48)	–	1·16 (0·45–2·98)	0·62 (0·01–26·36)	1·42 (0·35–5·66)
Studies published before 2011	14	2·25 (1·33–3·80)	1·24 (0·70–2·20)	1·46 (1·15–1·86)	1·86 (1·27–2·72)	1·02 (0·74–1·39)	–	0·69 (0·14–3·45)
Studies with median age ≥30 years	9	2·67 (1·37–5·19)	0·74 (0·12–4·46)	1·66 (1·50–1·84)	–	1·18 (0·03–55·00)	0·81 (0·02–34·74)	0·31 (0·04–2·49)
Studies with median age <30 years	11	1·90 (1·51–2·39)	1·83 (1·46–2·30)	1·19 (1·02–1·39)	1·84 (1·44–2·35)	1·00 (0·80–1·24)	–	1·33 (0·33–5·30)
Studies with percentage of nulliparae ≥49%	7	2·02 (1·58–2·57)	1·40 (0·80–2·44)	1·25 (1·05–1·50)	1·84 (1·44–2·35)	0·98 (0·79–1·22)	–	–
Studies with percentage of nulliparae <49%	7	2·76 (1·50–5·09)	2·47 (1·86–3·27)	1·54 (1·32–1·80)	–	1·78 (0·63–5·01)	–	1·76 (0·44–7·09)
Studies with percentage of smokers ≥16%	8	2·31 (1·02–5·22)	1·79 (1·14–2·82)	1·40 (1·06–1·86)	–	0·94 (0·54–1·64)	0·69 (0·02–30·52)	0·84 (0·25–2·84)
Studies with percentage of smokers <16%	5	2·26 (1·47–3·48)	–	1·37 (0·92–2·05)	–	–	–	–
Studies where ascertainment of exposure was through hospital records	23	2·32 (1·56–3·46)	1·20 (0·75–1·93)	1·31 (1·08–1·60)	1·86 (1·45–2·37)	1·00 (0·80–1·24)	0·64 (0·01–27·62)	2·42 (0·22–26·62)
Studies where ascertainment of exposure was through registries	5	2·39 (1·48–3·88)	2·31 (1·39–3·83)	1·45 (1·10–1·93)	–	1·54 (0·48–4·91)	–	1·65 (0·38–7·04)
Studies where ascertainment of outcome was through hospital records	16	2·70 (1·63–4·47)	1·24 (0·74–2·07)	1·24 (1·00–1·55)	–	0·76 (0·48–1·19)	0·61 (0·01–26·19)	0·25 (0·03–2·04)
Studies where ascertainment of outcome was through registries	6	2·21 (1·54–3·17)	2·23 (1·48–3·36)	1·43 (1·15–1·78)	1·91 (1·30–2·81)	1·11 (0·76–1·60)	–	1·59 (0·38–6·59)
Studies conducted in middle-income countries	5	2·27 (1·01–5·13)	–	1·10 (0·48–2·51)	–	–	–	–

Studies conducted in high-income countries	23	2·21 (1·61–3·03)	1·86 (1·35–2·57)	1·39 (1·18–1·64)	1·85 (1·28–2·68)	1·01 (0·75–1·36)	0·68 (0·02–29·55)	1·03 (0·36–2·96)
Studies with percentage of women treated for high-grade disease (CIN2+) ≥83%	10	2·98 (1·84–4·81)	2·61 (2·10–3·24)	1·64 (1·48–1·82)	–	1·64 (0·66–4·05)	0·80 (0·02–34·32)	1·87 (0·47–7·43)
Studies with percentage of women treated for high-grade disease (CIN2+) <83%	7	1·78 (1·02–3·12)	1·49 (0·86–2·58)	1·68 (1·25–2·26)	1·86 (1·46–2·38)	1·01 (0·81–1·26)	–	0·28 (0·04–2·28)
Studies with percentage of women treated for high-grade disease (CIN3+) ≥61%	6	2·55 (1·23–5·29)	2·61 (2·10–3·25)	1·65 (1·48–1·83)	–	1·89 (0·68–5·23)	–	1·87 (0·47–7·44)
Studies with percentage of women treated for high-grade disease (CIN3+) <61%	5	2·58 (1·24–5·39)	0·70 (0·11–4·55)	1·57 (0·93–2·67)	–	–	–	0·30 (0·04–2·50)
Studies with percentage of women treated for AIS >0%	3	–	–	–	–	–	–	–
Studies with percentage of women treated for AIS =0%	9	2·85 (1·53–5·32)	1·06 (0·11–9·96)	1·73 (1·10–2·72)	–	0·41 (0·02–7·62)	–	–
Studies with percentage of women treated for cervical cancer >0%	5	1·95 (1·13–3·38)	2·07 (1·47–2·92)	1·39 (1·02–1·89)	1·86 (1·41–2·44)	1·02 (0·80–1·30)	–	1·52 (0·37–6·21)
Studies with percentage of women treated for cervical cancer =0%	14	2.50 (1.34–4.68)	1.05 (0.28–3.84)	1.58 (0.95–2.66)	–	0.40 (0.04–3.98)	0.78 (0.02–34.34)	0.76 (0.15–3.94)
Sensitivity analysis: excluding non-randomised studies	2	1·59 (0·30–8·37)	0·27 (0·01–9·12)	–	–	–	–	–
Sensitivity analysis: excluding non-randomised studies at high risk of bias	15	2·21 (1·65–2·96)	1·90 (1·39–2·59)	1·36 (1·16–1·60)	1·85 (1·30–2·63)	1·01 (0·76–1·34)	–	1·44 (0·35–5·85)
Sensitivity analysis: including only studies reporting both spontaneous and iatrogenic preterm birth	23	2·29 (1·59–3·30)	1·83 (1·46–2·28)	1·22 (1·06–1·41)	1·87 (1·46–2·39)	1·03 (0·83–1·28)	–	1·55 (0·47–5·16)

2.9. Effect of Follow-up Duration

2.9.1. Treatment Failure

In the main analysis we considered the treatment failure rates throughout the study period regardless of the f-u duration of each study. In this section we present subgroup analyses according to the f-u duration, where the analysis of the treatment failure rates throughout the study period was restricted to studies with median (or mean if median not reported) f-u duration of at least 12, 24, 36, 48 and 60m, respectively. We additionally performed an analysis of the treatment failure rates up to 6m. We chose 6m since this is usually the time point when the first f-u visit after treatment takes place. Abnormal cytology or histology at this point is more likely to represent true residual disease or recurrence, rather than acquisition of a new HPV infection. If visit at 6m was not reported, we included the visit at 3–9m (whichever visit closest to 6m was reported).

Analyses are presented in league tables, where each box represents the comparison of the row-defining treatment vs the column-defining treatment. OR is reported first, followed by 95% CI and 95% PI. ORs>1 favour the column-defining treatment, while ORs<1 favour the row-defining treatment. After league tables we present the results of all analyses in a summary figure.

Table 2.9.1.1: Risk of treatment failure throughout the study period regardless of follow-up duration (main analysis) (N=71 studies)

**CKC (P-score: 0·89)**	1·07 (0·76–1·50) (0·53–2·17)	0·36 (0·20–0·64) (0·15–0·84)	0·38 (0·27–0·53) (0·18–0·76)	0·58 (0·35–0·96) (0·26–1·30)	0·34 (0·24–0·50) (0·17–0·71)	0·63 (0·50–0·81) (0·33–1·23)
0·93 (0·67–1·31) (0·46–1·89)	**LC (P-score: 0·94)**	0·34 (0·18–0·64) (0·14–0·82)	0·35 (0·25–0·50) (0·17–0·72)	0·54 (0·32–0·93) (0·24–1·24)	0·32 (0·21–0·48) (0·15–0·67)	0·59 (0·44–0·79) (0·30–1·17)
2·79 (1·57–4·94) (1·19–6·51)	2·98 (1·57–5·67) (1·21–7·33)	**RD (P-score: 0·19)**	1·04 (0·56–1·95) (0·43–2·53)	1·62 (0·82–3·22) (0·64–4·12)	0·96 (0·51–1·80) (0·39–2·33)	1·76 (0·97–3·20) (0·74–4·19)
2·67 (1·89–3·75) (1·31–5·42)	2·86 (2·00–4·08) (1·39–5·85)	0·96 (0·51–1·78) (0·40–2·32)	**LA (P-score: 0·22)**	1·55 (0·96–2·52) (0·71–3·43)	0·92 (0·71–1·19) (0·47–1·80)	1·69 (1·27–2·24) (0·85–3·34)
1·72 (1·04–2·83) (0·77–3·82)	1·84 (1·08–3·13) (0·81–4·19)	0·62 (0·31–1·22) (0·24–1·56)	0·64 (0·40–1·04) (0·29–1·42)	**CC (P-score: 0·54)**	0·59 (0·36–0·96) (0·27–1·31)	1·09 (0·68–1·74) (0·50–2·37)
2·91 (2·01–4·21) (1·41–6·00)	3·12 (2·08–4·66) (1·48–6·54)	1·04 (0·56–1·96) (0·43–2·54)	1·09 (0·84–1·42) (0·56–2·14)	1·70 (1·04–2·78) (0·77–3·76)	**CT (P-score: 0·12)**	1·84 (1·33–2·56) (0·91–3·72)
1·58 (1·24–2·01) (0·81–3·07)	1·69 (1·26–2·26) (0·85–3·36)	0·57 (0·31–1·03) (0·24–1·35)	0·59 (0·45–0·79) (0·30–1·17)	0·92 (0·58–1·47) (0·42–2·01)	0·54 (0·39–0·75) (0·27–1·10)	**LLETZ (P-score: 0·60)**

Heterogeneity: τ^2=0·10; I^2=30% (6–48)

Table 2.9.1.2: Risk of treatment failure up to 6 months (N=23 studies)

**CKC<br>(P-score: 0·89)**	0·97<br>(0·52–1·83)<br>(0·37–2·53)	0·28<br>(0·10–0·77)<br>(0·08–1·00)	0·41<br>(0·21–0·79)<br>(0·15–1·09)	0·62<br>(0·31–1·24)<br>(0·23–1·69)	0·34<br>(0·16–0·71)<br>(0·12–0·96)	0·72<br>(0·41–1·25)<br>(0·29–1·76)
1·03<br>(0·55–1·93)<br>(0·40–2·68)	**LC<br>(P-score: 0·87)**	0·29<br>(0·10–0·88)<br>(0·07–1·14)	0·42<br>(0·23–0·78)<br>(0·16–1·09)	0·64<br>(0·31–1·32)<br>(0·23–1·78)	0·35<br>(0·17–0·71)<br>(0·13–0·97)	0·74<br>(0·43–1·26)<br>(0·30–1·79)
3·55<br>(1·30–9·68)<br>(1·00–12·62)	3·46<br>(1·14–10·52)<br>(0·88–13·58)	**RD<br>(P-score: 0·12)**	1·46<br>(0·49–4·32)<br>(0·38–5·58)	2·20<br>(0·80–6·08)<br>(0·61–7·91)	1·20<br>(0·39–3·75)<br>(0·30–4·83)	2·54<br>(0·90–7·19)<br>(0·69–9·34)
2·44<br>(1·26–4·70)<br>(0·92–6·46)	2·37<br>(1·28–4·39)<br>(0·92–6·10)	0·69<br>(0·23–2·03)<br>(0·18–2·62)	**LA<br>(P-score: 0·27)**	1·51<br>(0·83–2·75)<br>(0·59–3·84)	0·83<br>(0·51–1·33)<br>(0·35–1·92)	1·74<br>(1·14–2·66)<br>(0·77–3·94)
1·62<br>(0·81–3·23)<br>(0·59–4·41)	1·57<br>(0·76–3·25)<br>(0·56–4·40)	0·45<br>(0·16–1·26)<br>(0·13–1·63)	0·66<br>(0·36–1·21)<br>(0·26–1·69)	**CC<br>(P-score: 0·55)**	0·55<br>(0·28–1·09)<br>(0·20–1·49)	1·16<br>(0·67–1·99)<br>(0·47–2·82)
2·95<br>(1·41–6·16)<br>(1·05–8·33)	2·87<br>(1·40–5·89)<br>(1·03–7·99)	0·83<br>(0·27–2·58)<br>(0·21–3·33)	1·21<br>(0·75–1·94)<br>(0·52–2·82)	1·83<br>(0·92–3·63)<br>(0·67–4·96)	**CT<br>(P-score: 0·15)**	2·11<br>(1·23–3·63)<br>(0·87–5·15)
1·40<br>(0·80–2·43)<br>(0·57–3·44)	1·36<br>(0·79–2·34)<br>(0·56–3·31)	0·39<br>(0·14–1·11)<br>(0·11–1·45)	0·57<br>(0·38–0·88)<br>(0·25–1·30)	0·87<br>(0·50–1·49)<br>(0·35–2·12)	0·47<br>(0·28–0·82)<br>(0·19–1·16)	**LLETZ<br>(P-score: 0·65)**

Heterogeneity: τ^2=0·10; I^2=29% (0–58)

Table 2.9.1.3: Risk of treatment failure throughout the study period in studies with median follow-up at least 12 months (N=44 studies)

**CKC (P-score: 0·77)**	1·26 (0·82–1·93) (0·51–3·10)	0·63 (0·15–2·65) (0·12–3·36)	0·38 (0·24–0·60) (0·15–0·95)	0·32 (0·20–0·52) (0·13–0·81)	0·66 (0·48–0·91) (0·28–1·55)
0·79 (0·52–1·21) (0·32–1·95)	**LC (P-score: 0·94)**	0·50 (0·12–2·14) (0·09–2·70)	0·30 (0·19–0·49) (0·12–0·77)	0·25 (0·15–0·43) (0·10–0·66)	0·52 (0·36–0·77) (0·22–1·27)
1·60 (0·38–6·76) (0·30–8·55)	2·01 (0·47–8·67) (0·37–10·95)	**RD (P-score: 0·50)**	0·61 (0·15–2·45) (0·12–3·11)	0·51 (0·13–2·01) (0·10–2·56)	1·06 (0·25–4·40) (0·20–5·57)
2·62 (1·67–4·12) (1·05–6·52)	3·31 (2·03–5·39) (1·30–8·39)	1·64 (0·41–6·61) (0·32–8·40)	**LA (P-score: 0·22)**	0·84 (0·60–1·16) (0·36–1·96)	1·74 (1·19–2·53) (0·72–4·16)
3·14 (1·94–5·07) (1·24–7·93)	3·96 (2·32–6·75) (1·52–10·31)	1·97 (0·50–7·78) (0·39–9·90)	1·20 (0·86–1·66) (0·51–2·81)	**CT (P-score: 0·06)**	2·08 (1·36–3·18) (0·85–5·10)
1·51 (1·10–2·06) (0·65–3·52)	1·90 (1·29–2·81) (0·79–4·59)	0·95 (0·23–3·93) (0·18–4·98)	0·58 (0·39–0·84) (0·24–1·38)	0·48 (0·31–0·74) (0·20–1·18)	**LLETZ (P-score: 0·51)**

Heterogeneity: τ^2=0·15; I^2=43% (19–60)

Table 2.9.1.4: Risk of treatment failure throughout the study period in studies with median follow-up at least 24 months (N=30 studies)

**CKC (P-score: 0·71)**	1·24 (0·87–1·77) (0·79–1·94)	1·09 (0·24–4·91) (0·22–5·36)	0·30 (0·21–0·43) (0·19–0·47)	0·32 (0·22–0·47) (0·20–0·51)	0·66 (0·50–0·86) (0·45–0·96)
0·81 (0·57–1·15) (0·52–1·26)	**LC (P-score: 0·89)**	0·88 (0·19–4·02) (0·18–4·39)	0·24 (0·16–0·37) (0·15–0·40)	0·26 (0·16–0·40) (0·15–0·44)	0·53 (0·38–0·75) (0·34–0·82)
0·92 (0·20–4·11) (0·19–4·49)	1·14 (0·25–5·19) (0·23–5·67)	**RD (P-score: 0·73)**	0·28 (0·06–1·20) (0·06–1·31)	0·29 (0·07–1·25) (0·06–1·36)	0·60 (0·14–2·67) (0·12–2·92)
3·33 (2·31–4·80) (2·11–5·26)	4·13 (2·72–6·27) (2·50–6·83)	3·64 (0·83–15·88) (0·76–17·32)	**LA (P-score: 0·07)**	1·06 (0·82–1·37) (0·73–1·53)	2·19 (1·63–2·94) (1·47–3·25)
3·14 (2·13–4·63) (1·95–5·05)	3·90 (2·48–6·11) (2·29–6·63)	3·43 (0·80–14·64) (0·74–15·96)	0·94 (0·73–1·22) (0·65–1·36)	**CT (P-score: 0·14)**	2·06 (1·47–2·90) (1·34–3·19)
1·52 (1·16–1·99) (1·04–2·21)	1·89 (1·34–2·66) (1·22–2·92)	1·66 (0·37–7·38) (0·34–8·05)	0·46 (0·34–0·61) (0·31–0·68)	0·48 (0·35–0·68) (0·31–0·75)	**LLETZ (P-score: 0·45)**

Heterogeneity: τ^2=0·01; I^2=7% (0–38)

Table 2.9.1.5: Risk of treatment failure throughout the study period in studies with median follow-up at least 36 months (N=16 studies)

**CKC**<br>**(P-score: 0·70)**	1·26<br>(0·87–1·82)<br>(0·84–1·89)	1·12<br>(0·25–5·00)<br>(0·22–5·76)	0·32<br>(0·21–0·50)<br>(0·20–0·52)	0·33<br>(0·21–0·50)<br>(0·20–0·52)	0·73<br>(0·50–1·05)<br>(0·49–1·09)
0·80<br>(0·55–1·15)<br>(0·53–1·19)	**LC**<br>**(P-score: 0·89)**	0·89<br>(0·20–4·00)<br>(0·17–4·61)	0·26<br>(0·17–0·40)<br>(0·16–0·42)	0·26<br>(0·17–0·41)<br>(0·16–0·43)	0·58<br>(0·40–0·83)<br>(0·39–0·86)
0·89<br>(0·20–3·98)<br>(0·17–4·58)	1·12<br>(0·25–5·04)<br>(0·22–5·80)	**RD**<br>**(P-score: 0·73)**	0·29<br>(0·07–1·23)<br>(0·06–1·41)	0·29<br>(0·07–1·22)<br>(0·06–1·40)	0·65<br>(0·15–2·83)<br>(0·13–3·25)
3·08<br>(2·01–4·73)<br>(1·93–4·92)	3·88<br>(2·50–6·01)<br>(2·40–6·27)	3·46<br>(0·81–14·74)<br>(0·71–16·90)	**LA**<br>**(P-score: 0·10)**	1·01<br>(0·80–1·27)<br>(0·78–1·30)	2·24<br>(1·63–3·09)<br>(1·58–3·18)
3·06<br>(1·99–4·70)<br>(1·91–4·89)	3·84<br>(2·44–6·05)<br>(2·34–6·31)	3·43<br>(0·82–14·35)<br>(0·72–16·43)	0·99<br>(0·79–1·25)<br>(0·77–1·28)	**CT**<br>**(P-score: 0·11)**	2·22<br>(1·57–3·14)<br>(1·52–3·24)
1·38<br>(0·95–1·98)<br>(0·92–2·05)	1·73<br>(1·20–2·49)<br>(1·16–2·58)	1·54<br>(0·35–6·73)<br>(0·31–7·73)	0·45<br>(0·32–0·61)<br>(0·31–0·63)	0·45<br>(0·32–0·64)<br>(0·31–0·66)	**LLETZ**<br>**(P-score: 0·47)**

Heterogeneity: τ^2=0·00; I^2=0% (0–52)

Table 2.9.1.6: Risk of treatment failure throughout the study period in studies with median follow-up at least 48 months (N=12 studies)

**CKC**<br>**(P-score: 0·78)**	1·22<br>(0·83–1·80)<br>(0·79–1·90)	0·42<br>(0·22–0·79)<br>(0·21–0·86)	0·38<br>(0·21–0·70)<br>(0·19–0·76)	0·66<br>(0·44–1·01)<br>(0·41–1·07)
0·82<br>(0·55–1·21)<br>(0·53–1·27)	**LC**<br>**(P-score: 0·96)**	0·35<br>(0·18–0·67)<br>(0·16–0·74)	0·31<br>(0·16–0·60)<br>(0·15–0·66)	0·54<br>(0·37–0·81)<br>(0·35–0·85)
2·37<br>(1·26–4·44)<br>(1·16–4·84)	2·90<br>(1·49–5·64)<br>(1·36–6·18)	**LA**<br>**(P-score: 0·21)**	0·91<br>(0·69–1·20)<br>(0·66–1·25)	1·58<br>(0·77–3·22)<br>(0·70–3·55)
2·61<br>(1·43–4·75)<br>(1·32–5·16)	3·19<br>(1·66–6·11)<br>(1·52–6·68)	1·10<br>(0·83–1·46)<br>(0·80–1·51)	**CT**<br>**(P-score: 0·08)**	1·73<br>(0·86–3·48)<br>(0·79–3·82)
1·50<br>(0·99–2·28)<br>(0·94–2·41)	1·84<br>(1·24–2·73)<br>(1·17–2·89)	0·63<br>(0·31–1·30)<br>(0·28–1·43)	0·58<br>(0·29–1·16)<br>(0·26–1·27)	**LLETZ**<br>**(P-score: 0·47)**

Heterogeneity: τ^2=0·00; I^2=0% (0–58)

Table 2.9.1.7: Risk of treatment failure throughout the study period in studies with median follow-up at least 60 months (N=7 studies)

**CKC (P-score: 0·72)**	1·49 (0·97–2·28) (0·74–2·97)	0·26 (0·12–0·55) (0·07–0·88)	0·24 (0·12–0·49) (0·08–0·76)	0·77 (0·49–1·24) (0·36–1·65)
0·67 (0·44–1·03) (0·34–1·34)	**LC (P-score: 0·99)**	0·17 (0·07–0·41) (0·04–0·71)	0·16 (0·07–0·37) (0·04–0·62)	0·52 (0·34–0·79) (0·26–1·02)
3·90 (1·82–8·37) (1·13–13·47)	5·80 (2·42–13·90) (1·40–23·98)	**LA (P-score: 0·16)**	0·95 (0·70–1·29) (0·58–1·56)	3·02 (1·23–7·39) (0·71–12·91)
4·09 (2·03–8·24) (1·31–12·76)	6·08 (2·68–13·81) (1·61–23·04)	1·05 (0·77–1·42) (0·64–1·71)	**CT (P-score: 0·10)**	3·17 (1·36–7·35) (0·81–12·43)
1·29 (0·81–2·06) (0·60–2·76)	1·92 (1·27–2·91) (0·98–3·78)	0·33 (0·14–0·81) (0·08–1·42)	0·32 (0·14–0·73) (0·08–1·24)	**LLETZ (P-score: 0·53)**

Heterogeneity: τ^2=0·00; I^2=0% (0–79)

Summary Figure

Figure 2.9.1.1: Risk of CIN treatment failure according to follow-up duration

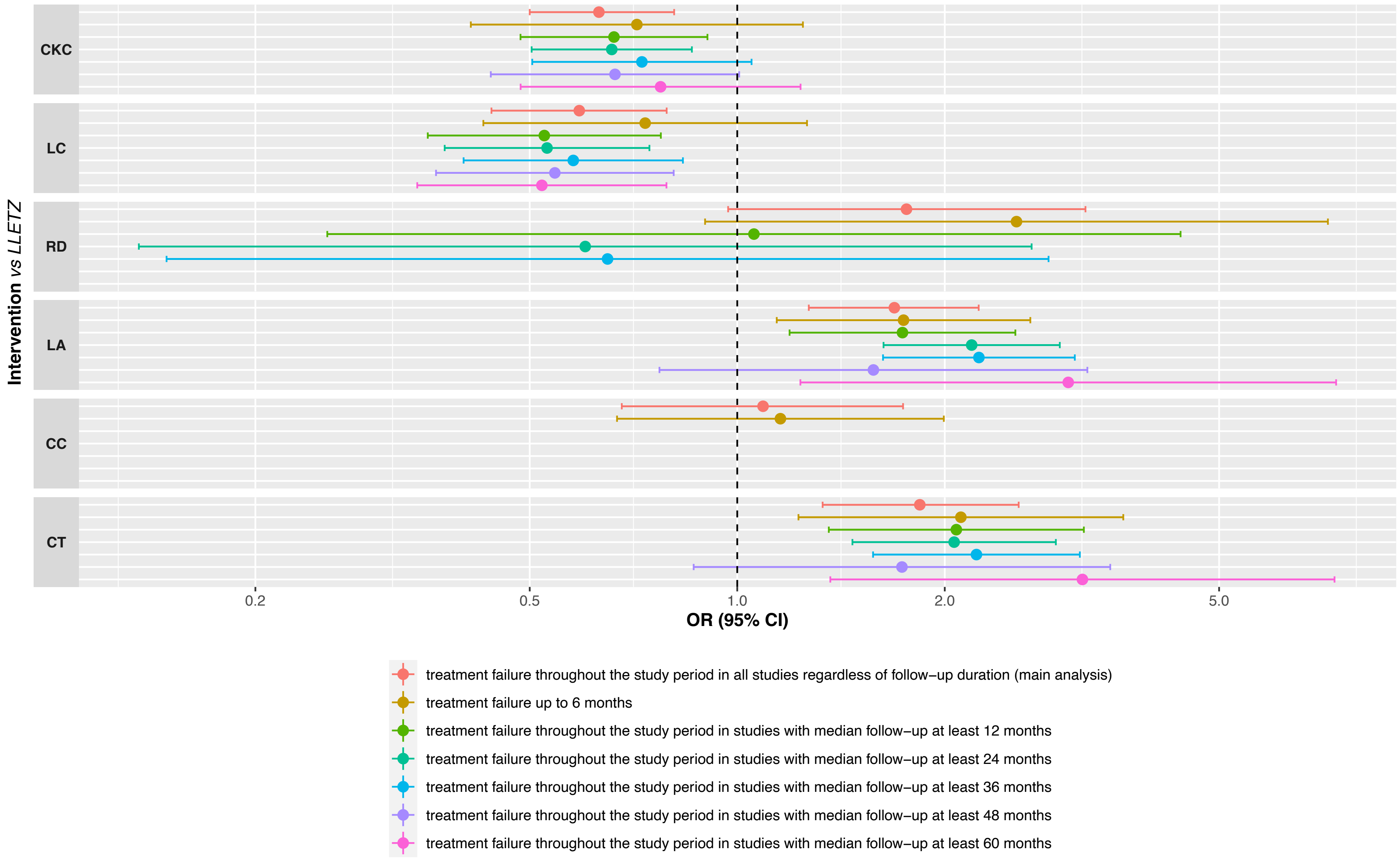

2.9.2. Preterm Birth

In the analysis for preterm birth, some studies recruited women at the time of the initial CIN treatment and followed them up (prospectively or retrospectively) until pregnancy. However, the majority of studies recruited women at the time of their pregnancy or delivery and retrospectively looked into how many of them had received treatment for CIN in the past. In the latter design, f-u duration (i.e. the interval from CIN treatment to pregnancy) cannot be defined, thus we did not perform subgroup analyses according to f-u duration for preterm birth.

2.10. HIV-Infected Women

2.10.1. Treatment Failure

In the main analysis we excluded studies with HIV-infected women in over 20% of their population, since HIV-infected women represent a different population with a different risk of recurrence.[204] In a separate analysis we included studies where >20% or the whole population was infected with HIV. The eligibility criteria remained the same as in the main analysis (e.g. we excluded studies where ablation was performed without prior histological confirmation or where ablation might have been used in women with endocervical lesions and/or unsatisfactory colposcopy).

We found four studies which compared treatment failure rates amongst different CIN treatment techniques and had >20% HIV-infected participants.[67,68,105,106] Of these, three recruited only HIV-infected women, whilst a fourth study recruited 44 HIV-positive and 44 HIV-negative age-matched women and presented results separately for HIV-positive and HIV-negative women. Because results were presented separately for HIV-positive women in all studies, we performed a network meta-analysis exclusively of HIV-infected women. We also performed a subgroup analysis which excluded studies where <90% of women received ART (or this was not reported).

A fifth study compared hrHPV positivity rates after different CIN treatment techniques amongst HIV-infected women.[205] Since this was the only study that reported this outcome amongst HIV-infected women, it was not included in any analysis.

Analyses are presented in league tables, where each box represents the comparison of the row-defining treatment vs the column-defining treatment. OR is reported first, followed by 95% CI and 95% PI. ORs>1 favour the column-defining treatment, while ORs<1 favour the row-defining treatment.

Table 2.10.1.1: Risk of treatment failure in HIV-infected women (N=3 studies*)

**CKC<br>(P-score: 0·93)**	0·23<br>(0·06–0·91)<br>(NA, NA)	0·47<br>(0·13–1·70)<br>(NA, NA)
4·29<br>(1·10–16·74)<br>(NA, NA)	**CT<br>(P-score: 0·01)**	2·03<br>(1·27–3·25)<br>(NA, NA)
2·11<br>(0·59–7·57)<br>(NA, NA)	0·49<br>(0·31–0·79)<br>(NA, NA)	**LLETZ<br>(P-score: 0·56)**

Heterogeneity: τ^2=0·01; I^2=2% (95% CI NA)

*A fourth study of ten participants comparing laser conisation to laser ablation was excluded because it was disconnected from the rest of the network. The treatment failure rate in this study was 3/6 in laser conisation group and 2/4 in laser ablation group.

Table 2.10.1.2: Risk of treatment failure in HIV-infected women in studies where >90% of women received antiretroviral therapy (N=2 studies)

**CT**<br>**(P-score: 0·00)**	2·03<br>(1·27–3·25)<br>(NA, NA)
0·49<br>(0·31–0·79)<br>(NA, NA)	**LLETZ**<br>**(P-score: 1·00)**

Heterogeneity: τ^2=0·01; I^2=2% (95% CI NA)

2.10.2. Preterm Birth

In the analysis of preterm birth, no studies had more than 20% of HIV-infected women.

2.11. Different Definitions of Outcome

2.11.1. Treatment Failure

In the main analysis we defined treatment failure as any abnormal cytology (ASC-US or worse) or histology (CIN1 or worse). This definition was chosen in agreement with our published protocol and the definition used in most studies. In secondary analyses we used different cut-offs to define treatment failure:

- High-grade treatment failure, defined as high-grade cytology (ASC-H or worse) or high-grade histology (CIN2 or worse)
- Histologically confirmed treatment failure, defined as histological CIN1+
- Histologically confirmed high-grade treatment failure, defined as histological CIN2+
- hrHPV positivity rates*
- Cervical cancer diagnosed after treatment

*We compared hrHPV positivity rates at 6m. If visit at 6m was not reported, we considered the visit at 3-9m (whichever visit closest to 6m was reported). All identified studies used hrHPV deoxyribonucleic acid (DNA) as test of cure (ToC); no studies used hrHPV messenger ribonucleic acid (mRNA) as ToC.

For some studies it was possible to extract two or more cut-offs for definition of treatment failure; in this case we used the lowest possible cut-off for the main analysis to include both histologically and cytologically confirmed lesions and both low-grade and high-grade lesions if possible. Studies reporting only high-grade treatment failures, for which it was not possible to extract low-grade treatment failures, were still included in the main analysis. If cytological and histological treatment failures were reported separately but not in combination (i.e. the study did not report how many women had abnormal cytology or abnormal histology or both), then we used histology in preference to cytology in the main analysis.

For the cervical cancer analysis we identified two studies reporting number of cancers per women-years stratified according to the CIN treatment technique.[206,207] A third case-control study was excluded due to not reporting women-years.[208] The first study[206] reported cancer incidence after laser conisation, LLETZ, laser ablation and cold coagulation. The cervical cancer incidence was greater after LLETZ than ablative techniques. However, the authors acknowledged that a meaningful comparison between excisional and ablative treatments was not possible, since excision had been mostly used in older women with more severe lesions. Additionally, the cone length excised with LLETZ was inappropriately shallow to treat endocervical lesions. The second study[207] concluded that the risk of cervical cancer was greater after CKC than LLETZ. However, CKC had been used in the first years of the study (1974–1978) when excision was not colposcopically-guided and f-u scheme differed compared to subsequent years (1979–2001). As such, we did not perform a network meta-analysis on cervical cancer incidence after treatment.

Analyses are presented in league tables, where each box represents the comparison of the row-defining treatment vs the column-defining treatment. OR is reported first, followed by 95% CI and 95% PI. ORs>1 favour the column-defining treatment, while ORs<1 favour the row-defining treatment. After league tables we present the results of all analyses in a summary table.

Table 2.11.1.1: Risk of any treatment failure, i.e. cytological ASC-US+ or histological CIN1+ (main analysis) (N=71 studies*)

**CKC (P-score: 0·89)**	1·07<br>(0·76–1·50)<br>(0·53–2·17)	0·36<br>(0·20–0·64)<br>(0·15–0·84)	0·38<br>(0·27–0·53)<br>(0·18–0·76)	0·58<br>(0·35–0·96)<br>(0·26–1·30)	0·34<br>(0·24–0·50)<br>(0·17–0·71)	0·63<br>(0·50–0·81)<br>(0·33–1·23)
0·93<br>(0·67–1·31)<br>(0·46–1·89)	**LC (P-score: 0·94)**	0·34<br>(0·18–0·64)<br>(0·14–0·82)	0·35<br>(0·25–0·50)<br>(0·17–0·72)	0·54<br>(0·32–0·93)<br>(0·24–1·24)	0·32<br>(0·21–0·48)<br>(0·15–0·67)	0·59<br>(0·44–0·79)<br>(0·30–1·17)
2·79<br>(1·57–4·94)<br>(1·19–6·51)	2·98<br>(1·57–5·67)<br>(1·21–7·33)	**RD (P-score: 0·19)**	1·04<br>(0·56–1·95)<br>(0·43–2·53)	1·62<br>(0·82–3·22)<br>(0·64–4·12)	0·96<br>(0·51–1·80)<br>(0·39–2·33)	1·76<br>(0·97–3·20)<br>(0·74–4·19)
2·67<br>(1·89–3·75)<br>(1·31–5·42)	2·86<br>(2·00–4·08)<br>(1·39–5·85)	0·96<br>(0·51–1·78)<br>(0·40–2·32)	**LA (P-score: 0·22)**	1·55<br>(0·96–2·52)<br>(0·71–3·43)	0·92<br>(0·71–1·19)<br>(0·47–1·80)	1·69<br>(1·27–2·24)<br>(0·85–3·34)
1·72<br>(1·04–2·83)<br>(0·77–3·82)	1·84<br>(1·08–3·13)<br>(0·81–4·19)	0·62<br>(0·31–1·22)<br>(0·24–1·56)	0·64<br>(0·40–1·04)<br>(0·29–1·42)	**CC (P-score: 0·54)**	0·59<br>(0·36–0·96)<br>(0·27–1·31)	1·09<br>(0·68–1·74)<br>(0·50–2·37)
2·91<br>(2·01–4·21)<br>(1·41–6·00)	3·12<br>(2·08–4·66)<br>(1·48–6·54)	1·04<br>(0·56–1·96)<br>(0·43–2·54)	1·09<br>(0·84–1·42)<br>(0·56–2·14)	1·70<br>(1·04–2·78)<br>(0·77–3·76)	**CT (P-score: 0·12)**	1·84<br>(1·33–2·56)<br>(0·91–3·72)
1·58<br>(1·24–2·01)<br>(0·81–3·07)	1·69<br>(1·26–2·26)<br>(0·85–3·36)	0·57<br>(0·31–1·03)<br>(0·24–1·35)	0·59<br>(0·45–0·79)<br>(0·30–1·17)	0·92<br>(0·58–1·47)<br>(0·42–2·01)	0·54<br>(0·39–0·75)<br>(0·27–1·10)	**LLETZ (P-score: 0·60)**

Heterogeneity: τ^2=0·10; I^2=30% (6–48)

* The main analysis included 13 studies reporting only high-grade treatment failures, for which it was not possible to extract low-grade treatment failures

Table 2.11.1.2: Risk of high-grade treatment failure, i.e. cytological ASC-H+ or histological CIN2+ (N=30 studies)

**CKC (P-score: 0·89)**	0·76 (0·33–1·73) (0·17–3·39)	0·29 (0·09–0·97) (0·05–1·70)	0·29 (0·13–0·63) (0·07–1·26)	0·72 (0·20–2·60) (0·12–4·45)	0·34 (0·14–0·84) (0·07–1·61)	0·65 (0·40–1·07) (0·17–2·47)
1·32 (0·58–2·99) (0·29–5·89)	**LC (P-score: 0·71)**	0·38 (0·09–1·61) (0·05–2·68)	0·38 (0·16–0·91) (0·08–1·76)	0·95 (0·24–3·81) (0·14–6·38)	0·45 (0·16–1·30) (0·09–2·36)	0·86 (0·42–1·77) (0·20–3·63)
3·45 (1·03–11·57) (0·59–20·15)	2·62 (0·62–11·05) (0·37–18·34)	**RD (P-score: 0·22)**	1·00 (0·25–3·99) (0·15–6·69)	2·49 (0·44–14·24) (0·28–22·56)	1·18 (0·27–5·16) (0·16–8·51)	2·26 (0·62–8·19) (0·36–14·01)
3·46 (1·59–7·55) (0·79–15·09)	2·63 (1·10–6·28) (0·57–12·12)	1·00 (0·25–4·02) (0·15–6·73)	**LA (P-score: 0·16)**	2·50 (0·67–9·34) (0·39–15·87)	1·19 (0·54–2·58) (0·27–5·17)	2·26 (1·12–4·56) (0·54–9·45)
1·38 (0·39–4·97) (0·22–8·52)	1·05 (0·26–4·20) (0·16–7·04)	0·40 (0·07–2·29) (0·04–3·63)	0·40 (0·11–1·49) (0·06–2·54)	**CC (P-score: 0·66)**	0·47 (0·13–1·76) (0·07–3·00)	0·91 (0·27–3·04) (0·15–5·29)
2·92 (1·19–7·16) (0·62–13·68)	2·21 (0·77–6·36) (0·42–11·54)	0·85 (0·19–3·69) (0·12–6·09)	0·84 (0·39–1·84) (0·19–3·68)	2·11 (0·57–7·84) (0·33–13·34)	**CT (P-score: 0·26)**	1·91 (0·79–4·61) (0·41–8·86)
1·53 (0·94–2·49) (0·40–5·77)	1·16 (0·57–2·38) (0·28–4·89)	0·44 (0·12–1·61) (0·07–2·75)	0·44 (0·22–0·89) (0·11–1·84)	1·10 (0·33–3·70) (0·19–6·45)	0·52 (0·22–1·26) (0·11–2·43)	**LLETZ (P-score: 0·60)**

Heterogeneity: τ^2=0·35; I^2=48% (19–67)

Table 2.11.1.3: Risk of histologically confirmed treatment failure, i.e. histological CIN1+ (N=22 studies)

**CKC<br>(P-score: 0·75)**	2·18<br>(0·96–4·91)<br>(0·56–8·42)	0·36<br>(0·18–0·70)<br>(0·10–1·26)	0·28<br>(0·14–0·55)<br>(0·08–0·99)	0·56<br>(0·33–0·93)<br>(0·17–1·80)
0·46<br>(0·20–1·04)<br>(0·12–1·78)	**LC<br>(P-score: 0·99)**	0·16<br>(0·07–0·41)<br>(0·04–0·68)	0·13<br>(0·05–0·33)<br>(0·03–0·54)	0·26<br>(0·11–0·59)<br>(0·07–1·00)
2·79<br>(1·43–5·45)<br>(0·79–9·85)	6·08<br>(2·43–15·20)<br>(1·46–25·27)	**LA<br>(P-score: 0·23)**	0·78<br>(0·49–1·24)<br>(0·25–2·47)	1·55<br>(0·91–2·65)<br>(0·48–5·08)
3·57<br>(1·82–7·03)<br>(1·01–12·66)	7·78<br>(3·03–19·98)<br>(1·83–32·98)	1·28<br>(0·81–2·03)<br>(0·40–4·04)	**CT<br>(P-score: 0·04)**	1·99<br>(1·12–3·53)<br>(0·60–6·64)
1·80<br>(1·08–3·00)<br>(0·56–5·81)	3·91<br>(1·71–8·95)<br>(1·00–15·27)	0·64<br>(0·38–1·09)<br>(0·20–2·10)	0·50<br>(0·28–0·89)<br>(0·15–1·68)	**LLETZ<br>(P-score: 0·49)**

Heterogeneity: τ^2=0·25; I^2=53% (25–71)

Table 2.11.1.4: Risk of histologically confirmed high-grade treatment failure, i.e. histological CIN2+ (N=18 studies)

**CKC (P-score: 0·77)**	1·16 (0·22–6·16) (0·10–14·20)	0·35 (0·07–1·74) (0·03–4·05)	0·32 (0·10–1·01) (0·04–2·65)	1·07 (0·08–14·57) (0·04–30·64)	0·44 (0·12–1·64) (0·05–4·09)	0·61 (0·32–1·17) (0·10–3·81)
0·86 (0·16–4·55) (0·07–10·49)	**LC (P-score: 0·75)**	0·30 (0·03–3·04) (0·01–6·48)	0·28 (0·06–1·22) (0·03–2·92)	0·92 (0·05–17·27) (0·02–36·04)	0·38 (0·06–2·47) (0·03–5·51)	0·53 (0·10–2·85) (0·04–6·55)
2·87 (0·57–14·37) (0·25–33·47)	3·34 (0·33–33·90) (0·15–72·41)	**RD (P-score: 0·28)**	0·92 (0·13–6·66) (0·06–14·68)	3·08 (0·14–66·08) (0·07–137·73)	1·27 (0·16–10·12) (0·07–22·06)	1·76 (0·31–9·97) (0·14–22·73)
3·12 (0·99–9·83) (0·38–25·74)	3·62 (0·82–16·08) (0·34–38·38)	1·08 (0·15–7·84) (0·07–17·27)	**LA (P-score: 0·19)**	3·34 (0·25–44·71) (0·12–94·07)	1·38 (0·38–4·95) (0·15–12·46)	1·91 (0·62–5·86) (0·24–15·48)
0·93 (0·07–12·70) (0·03–26·70)	1·09 (0·06–20·36) (0·03–42·49)	0·32 (0·02–6·97) (0·01–14·54)	0·30 (0·02–4·01) (0·01–8·44)	**CC (P-score: 0·67)**	0·41 (0·04–3·93) (0·02–8·43)	0·57 (0·04–8·03) (0·02–16·86)
2·27 (0·61–8·42) (0·24–21·00)	2·64 (0·41–17·13) (0·18–38·26)	0·79 (0·10–6·29) (0·05–13·72)	0·73 (0·20–2·62) (0·08–6·59)	2·43 (0·25–23·17) (0·12–49·70)	**CT (P-score: 0·35)**	1·39 (0·35–5·48) (0·14–13·45)
1·63 (0·86–3·10) (0·26–10·13)	1·90 (0·35–10·26) (0·15–23·56)	0·57 (0·10–3·21) (0·04–7·32)	0·52 (0·17–1·60) (0·06–4·24)	1·75 (0·12–24·49) (0·06–51·44)	0·72 (0·18–2·84) (0·07–6·96)	**LLETZ (P-score: 0·49)**

Heterogeneity: τ^2=0·60; I^2=57% (23–76)

Table 2.11.1.5: Risk of positive hrHPV DNA testing at 6 months after treatment (N=8 studies)

**CKC (P-score: 0·68)**	1·18 (0·37–3·84) (0·05–28·35)	0·23 (0·07–0·79) (0·01–6·02)	0·75 (0·30–1·92) (0·05–11·98)	0·74 (0·17–3·11) (0·02–28·75)	0·88 (0·45–1·73) (0·08–9·40)
0·84 (0·26–2·74) (0·04–20·21)	**LC (P-score: 0·77)**	0·20 (0·05–0·80) (0·01–7·09)	0·64 (0·20–2·03) (0·03–14·86)	0·62 (0·13–3·07) (0·01–32·71)	0·74 (0·28–1·95) (0·04–12·31)
4·30 (1·27–14·56) (0·17–111·24)	5·09 (1·26–20·65) (0·14–183·79)	**LA (P-score: 0·03)**	3·24 (0·97–10·83) (0·13–81·85)	3·16 (0·62–16·13) (0·06–177·35)	3·78 (1·37–10·44) (0·21–68·46)
1·33 (0·52–3·39) (0·08–21·08)	1·57 (0·49–5·02) (0·07–36·68)	0·31 (0·09–1·03) (0·01–7·79)	**CC (P-score: 0·46)**	0·98 (0·33–2·92) (0·05–20·28)	1·17 (0·61–2·24) (0·11–12·05)
1·36 (0·32–5·75) (0·03–53·20)	1·61 (0·33–7·96) (0·03–84·91)	0·32 (0·06–1·61) (0·01–17·75)	1·03 (0·34–3·07) (0·05–21·32)	**CT (P-score: 0·48)**	1·20 (0·33–4·28) (0·04–34·22)
1·14 (0·58–2·23) (0·11–12·15)	1·35 (0·51–3·53) (0·08–22·34)	0·26 (0·10–0·73) (0·01–4·79)	0·86 (0·45–1·64) (0·08–8·86)	0·84 (0·23–2·99) (0·03–23·92)	**LLETZ (P-score: 0·58)**

Heterogeneity: τ^2=0·18; I^2=70% (15–90)

Summary Table

Table 2.11.1.6: Risk of treatment failure according to cut-off used to defined failure

Definition / Comparison	N studies	CKC vs LLETZ	LC vs LLETZ	RD vs LLETZ	LA vs LLETZ	CC vs LLETZ	CT vs LLETZ
Any treatment failure, i.e. cytological ASC-US+ or histological CIN1+	71*	0·63 (0·50–0·81)	0·59 (0·44–0·79)	1·76 (0·97–3·20)	1·69 (1·27–2·24)	1·09 (0·68–1·74)	1·84 (1·33–2·56)
High-grade treatment failure, i.e. cytological ASC-H+ or histological CIN2+	30	0·65 (0·40–1·07)	0·86 (0·42–1·77)	2·26 (0·62–8·19)	2·26 (1·12–4·56)	0·91 (0·27–3·04)	1·91 (0·79–4·61)
Histologically confirmed treatment failure, i.e. histological CIN1+	22	0·56 (0·33–0·93)	0·26 (0·11–0·59)	-	1·55 (0·91–2·65)	-	1·99 (1·12–3·53)
Histologically confirmed high-grade treatment failure, i.e. histological CIN2+	18	0·61 (0·32–1·17)	0·53 (0·10–2·85)	1·76 (0·31–9·97)	1·91 (0·62–5·86)	0·57 (0·04–8·03)	1·39 (0·35–5·48)
hrHPV DNA positivity at 6 months after treatment	8	0·88 (0·45–1·73)	0·74 (0·28–1·95)	-	3·78 (1·37–10·44)	1·17 (0·61–2·24)	1·20 (0·33–4·28)

*The main analysis included 13 studies reporting only high-grade treatment failures, for which it was not possible to extract low-grade treatment failures

2.11.2. Preterm Birth

The definition of preterm birth was gestation less than 37w of gestation. We did not show secondary analyses for other definitions.

2.12. Absolute Risks

2.12.1. Treatment Failure

Table 2.12.1.1: Absolute risks of CIN treatment failure according to cut-off used to define failure

Treatment \ Definition	Any treatment failure, i.e. cytological ASC-US+ or histological CIN1+	High-grade treatment failure, i.e. cytological ASC-H+ or histological CIN2+	Histologically confirmed treatment failure, i.e. histological CIN1+	Histologically confirmed high-grade treatment failure, i.e. histological CIN2+	Positive hrHPV testing at 6 months after treatment
CKC	6·6% (5·4–8·5)	3·5% (2·2–5·6)	5·9% (3·6–9·4)	4·7% (2·6–8·6)	15·4% (8·4–26·3)
LC	6·3% (4·8–8·3)	4·6% (2·3–8·9)	2·8% (1·2–6·2)	4·1% (0·8–18·8)	13·3% (5·5–28·7)
RD	16·7% (9·9–26·7)	11·2% (3·3–31·3)	–	12·5% (2·5–44·7)	–
LA	16·2% (12·7–20·3)	11·2% (5·9–20·2)	14·8% (9·3–22·9)	13·4% (4·8–32·2)	43·7% (22·0–68·2)
CC	11·0% (7·2–16·6)	4·8% (1·5–14·4)	–	4·4% (0·3–39·5)	19·3% (11·1–31·6)
CT	17·3% (13·2–22·6)	9·6% (4·2–20·4)	18·3% (11·2–28·4)	10·1% (2·8–30·8)	19·8% (6·5–46·7)
LLETZ	10·2% (7·5–13·7)	5·3% (3·1–8·8)	10·1% (6·4–15·6)	7·5% (3·5–15·2)	17·1% (14·4–20·1)

Figure 2.12.1.1: Absolute risks of treatment failure after various treatments compared to LLETZ (Kilim plot)

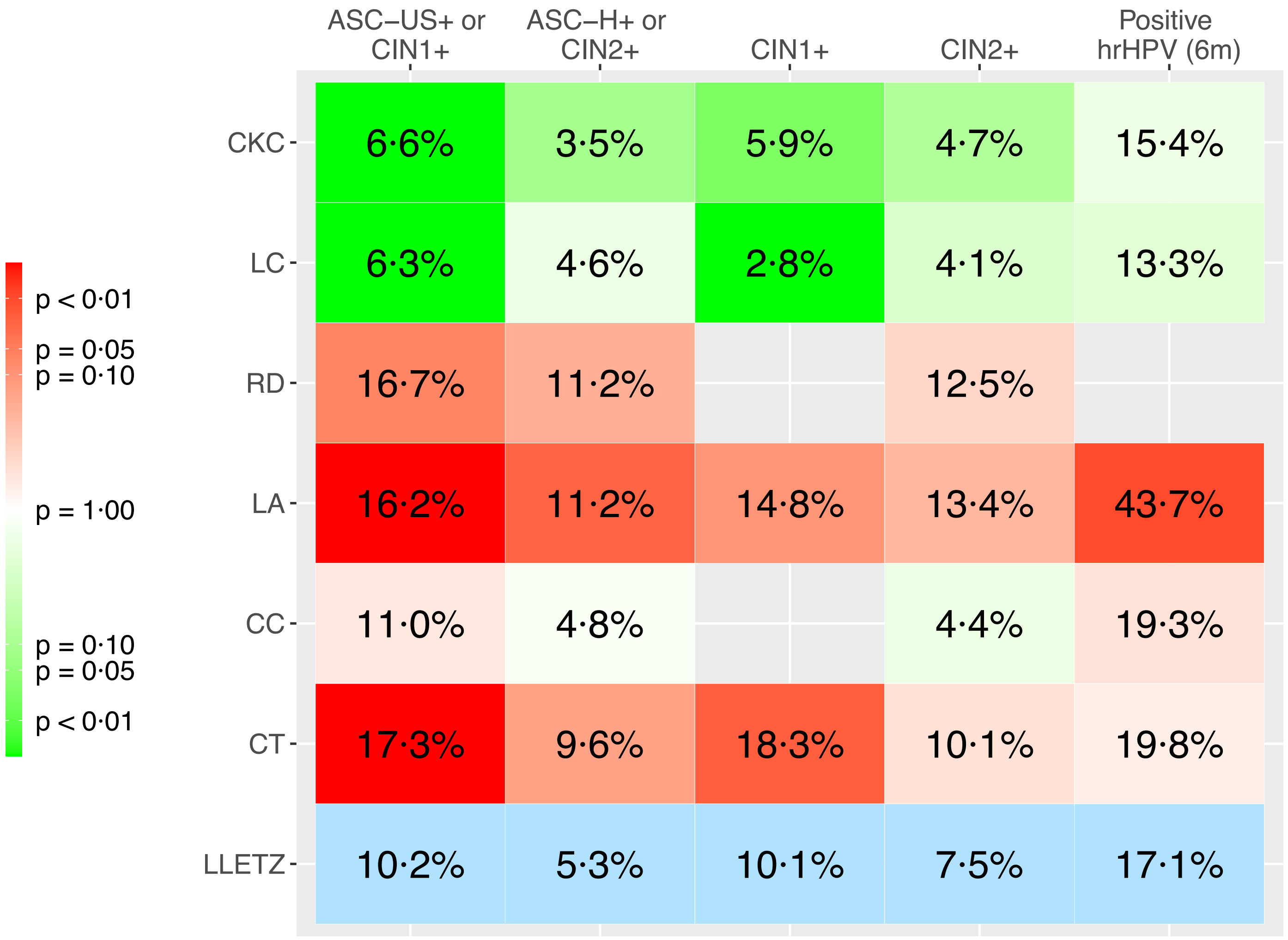

This figure shows the absolute risk of CIN treatment failure according to cut-off used to define failure (any treatment failure, i.e. cytological ASC-US+ or histological CIN1+; high-grade treatment failure, i.e. cytological ASC-H+ or histological CIN2+; histologically confirmed treatment failure, i.e. histological CIN1+; histologically confirmed high-grade treatment failure, i.e. histological CIN2+; positive hrHPV testing at 6m after treatment). The colour is correlated to the strength of the statistical evidence as regards the comparison of each treatment vs LLETZ. A deep rep colour indicates that the treatment performs worse than LLETZ; a deep green colour indicates that the treatment performs better than LLETZ. Colours closer to white indicate that there is lack of evidence regarding whether the treatment performs worse or better than LLETZ. LLETZ (the comparator) is shown in blue.

2.12.2. Preterm Birth

Table 2.12.2.1: Absolute risks of preterm birth in treated and untreated women

**Group**	**Absolute risk (95% CI)**
Colposcopy Group	7·9% (6·3–9·9)
External Group	6·3% (5·7–6·9)
Internal Group	7·4% (6·1–8·8)
**Treatment**	-
Any treatment	10·1% (9·2–11·2)
CKC	16·3% (12·7–20·6)
LC	13·2% (10·0–17·3)
LLETZ	10·5% (9·1–12·2)
RD	13·9% (10·1–18·9)
LA	8·3% (6·3–10·8)
CC	5·5% (0·2–71·5)
CT	8·0% (2·9–20·0)
**Cone length**	-
7mm	9·2% (8·2–10·4)
10mm	10·4% (8·8–12·3)

15mm	17·2%<br>(13·4–22·2)
20mm	32·4%<br>(21·2–49·3)

2.13. Effect of Cone Length

In this section we examine the relationship between length of excised cone and the risk of treatment failure or preterm birth by performing a dose-response meta-analysis. In the following figures the continuous line shows the odds ratios for the different cone lengths compared to reference. Dotted lines show the lower and upper limits of the 95% CI. For preterm birth we used restricted cubic splines, but for treatment failure we used a linear model due to limited data (only three studies).

2.13.1. Treatment Failure

Figure 2.13.1.1: Risk of treatment failure according to length of excised cone (reference: cone length of 5mm) (N=3 studies)

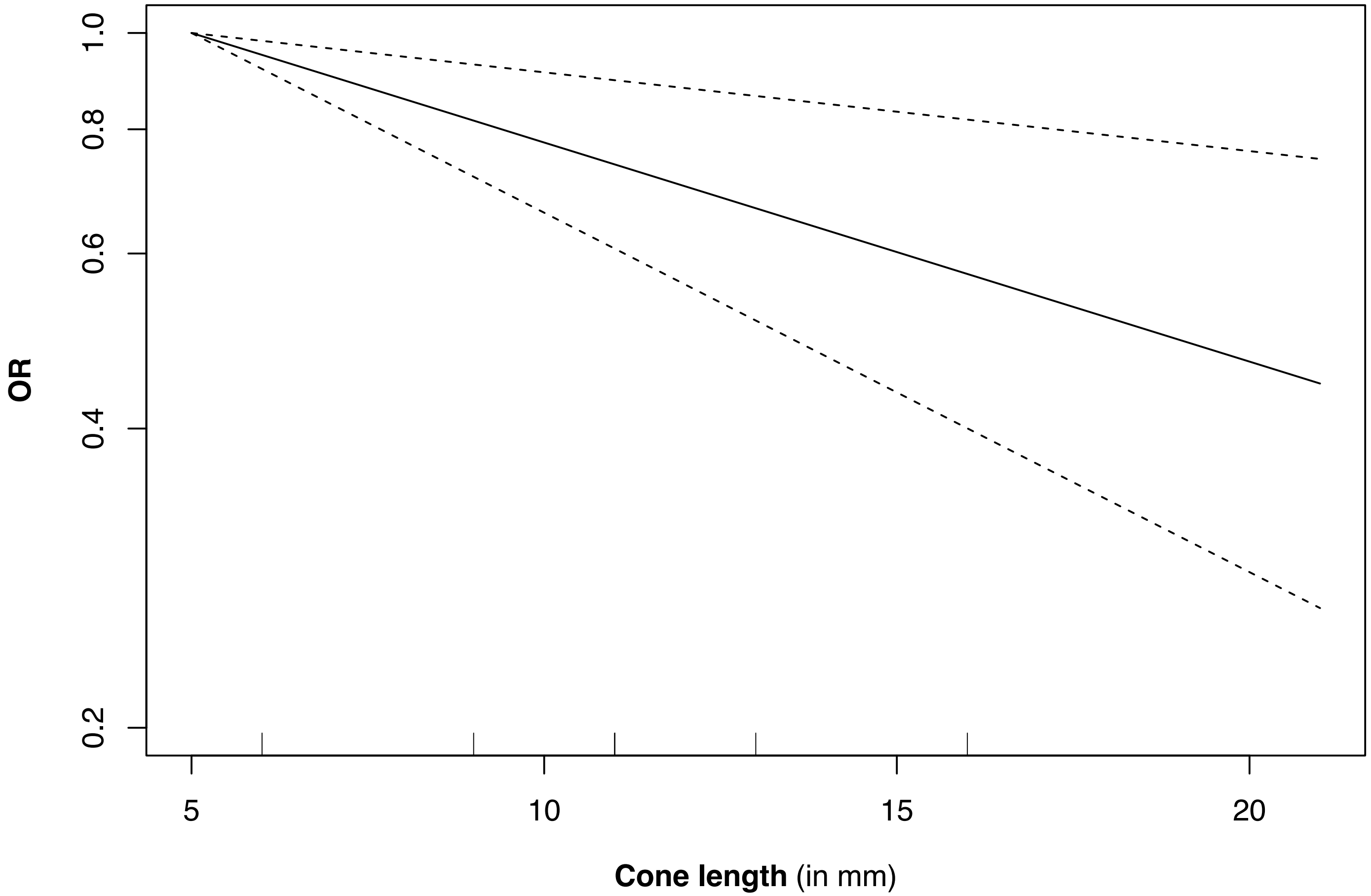

2.13.2. Preterm Birth

Figure 2.13.2.1: Risk of preterm birth according to length of excised cone (reference: women with untreated CIN) (N=20 studies)

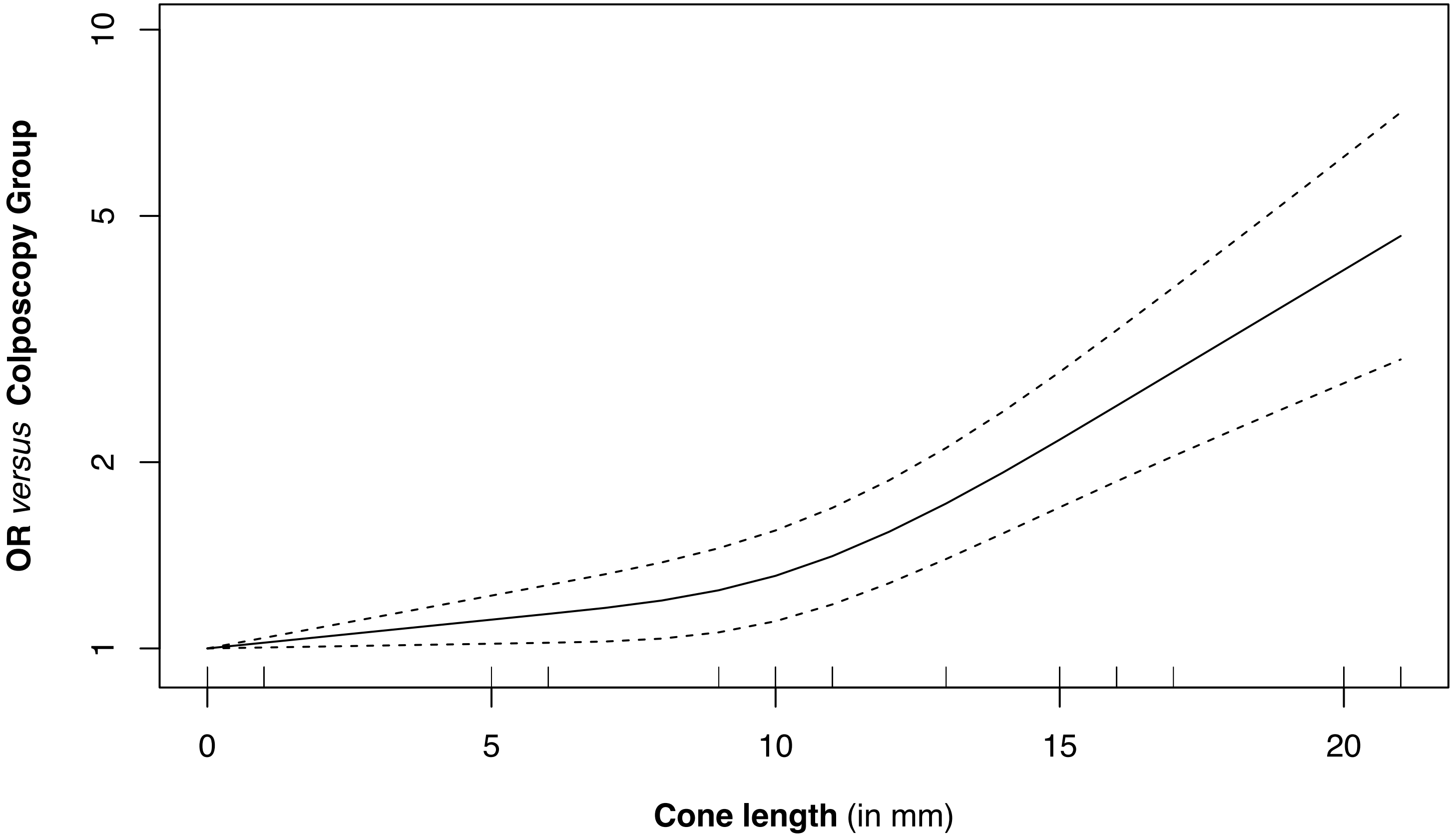

Figure 2.13.2.2: Risk of preterm birth according to length of excised cone (reference: women without CIN) (N=20 studies)

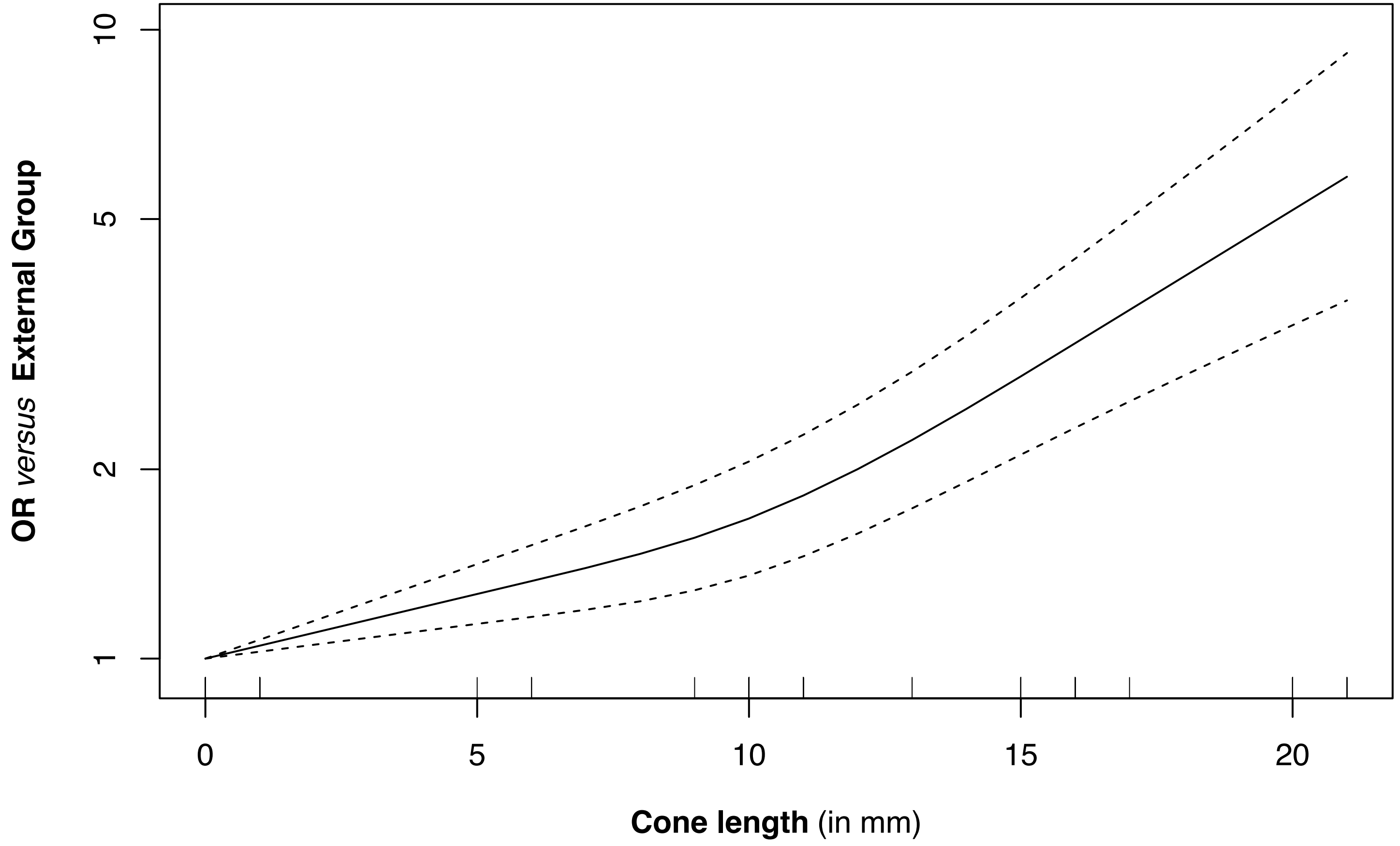

2.14. Assessment of the Credibility of Evidence (CINeMA)

We used the online ROB-MEN (Risk Of Bias due to Missing Evidence in Network meta-analysis) tool to assess for within-study and across-study bias as well as for small-study effects. We used the online CINeMA (Confidence In Network Meta-Analysis) tool to assess the overall credibility of evidence.

Note: the online ROB-MEN tool has been designed to perform a Bayesian network meta-analysis using absolute numbers, while we have performed a frequentist network meta-analysis using adjusted effect estimates (when available). Additionally, one study reporting on preterm birth rates after treatment provided only adjusted effect estimates but no absolute numbers; this study had to be excluded before running the ROB-MEN tool. As a result, numbers provided in Table 2.14.1.1, Table 2.14.1.2, Table 2.14.2.1 and Table 2.14.2.2 might differ from the numbers provided elsewhere, but any (small) possible discrepancies did not affect the overall judgement on the reporting bias.

2.14.1. Treatment Failure

Table 2.14.1.1: Assessing for within-study and across-study reporting bias using the ROB-MEN tool

	**N of studies in each comparison**		**Within-study assessment of bias**	**Across-study assessment of bias**	**Overall bias**
**Pairwise comparison**	Reporting this outcome (sample size)	Total identified in the systematic review (total sample size)	Evaluation of selective reporting within studies using signalling questions	Qualitative and quantitative assessment of publication bias	Overall judgement
**Group A:** **observed for this outcome**					
CKC–LC	6 (763)	7 (802)	No bias detected	No bias detected	No bias detected
CKC–LLETZ	27 (4421)	39 (10490)	No bias detected	No bias detected (Figure 2.14.1.1)	No bias detected
CKC–RD	2 (903)	3 (1734)	No bias detected	No bias detected	No bias detected
CKC–LA	2 (100)	3 (1176)	No bias detected	No bias detected	No bias detected
CKC–CC	1 (154)	1 (154)	No bias detected	No bias detected	No bias detected
CKC–CT	2 (792)	4 (885)	No bias detected	No bias detected	No bias detected
LC–LLETZ	11 (3961)	15 (4501)	No bias detected	No bias detected (Figure 2.14.1.2)	No bias detected
LC–LA	5 (947)	8 (1455)	No bias detected	No bias detected	No bias detected
LLETZ–LA	8 (1840)	11 (3550)	No bias detected	No bias detected	No bias detected
LLETZ–CC	2 (708)	4 (2465)	No bias detected	No bias detected	No bias detected
LLETZ–CT	2 (660)	3 (771)	No bias detected	No bias detected	No bias detected
RD–LA	1 (61)	2 (1826)	No bias detected	No bias detected	No bias detected
RD–CC	1 (134)	1 (134)	No bias detected	No bias detected	No bias detected

RD–CT	1 (57)	1 (57)	No bias detected	No bias detected	No bias detected
LA–CC	1 (132)	1 (132)	No bias detected	No bias detected	No bias detected
LA–CT	13 (4873)	13 (4873)	No bias detected	No bias detected (Figure 2.14.1.3)	No bias detected
CC–CT	2 (300)	2 (300)	No bias detected	No bias detected	No bias detected
**Group B: observed for other outcomes**					
LLETZ–RD	0 (0)	1 (829)	No bias detected	No bias detected	No bias detected
**Group C: Unobserved**					
LC–RD	0 (0)	0 (0)	NA	No bias detected	No bias detected
LC–CC	0 (0)	0 (0)	NA	No bias detected	No bias detected
LC–CT	0 (0)	0 (0)	NA	No bias detected	No bias detected

Figure 2.14.1.1: Contour-enhanced funnel plot for CKC vs LLETZ

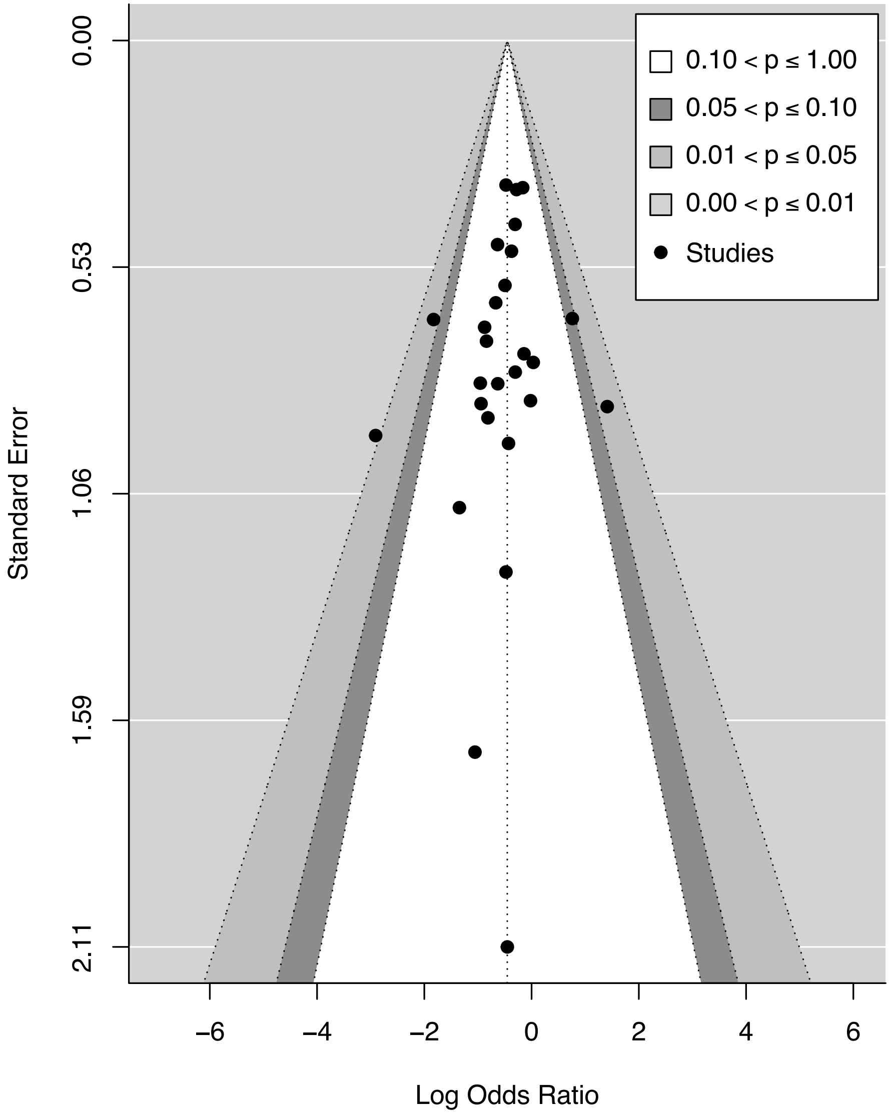

p-value from Egger test: 0·28

Figure 2.14.1.2: Contour-enhanced funnel plot for LC vs LLETZ

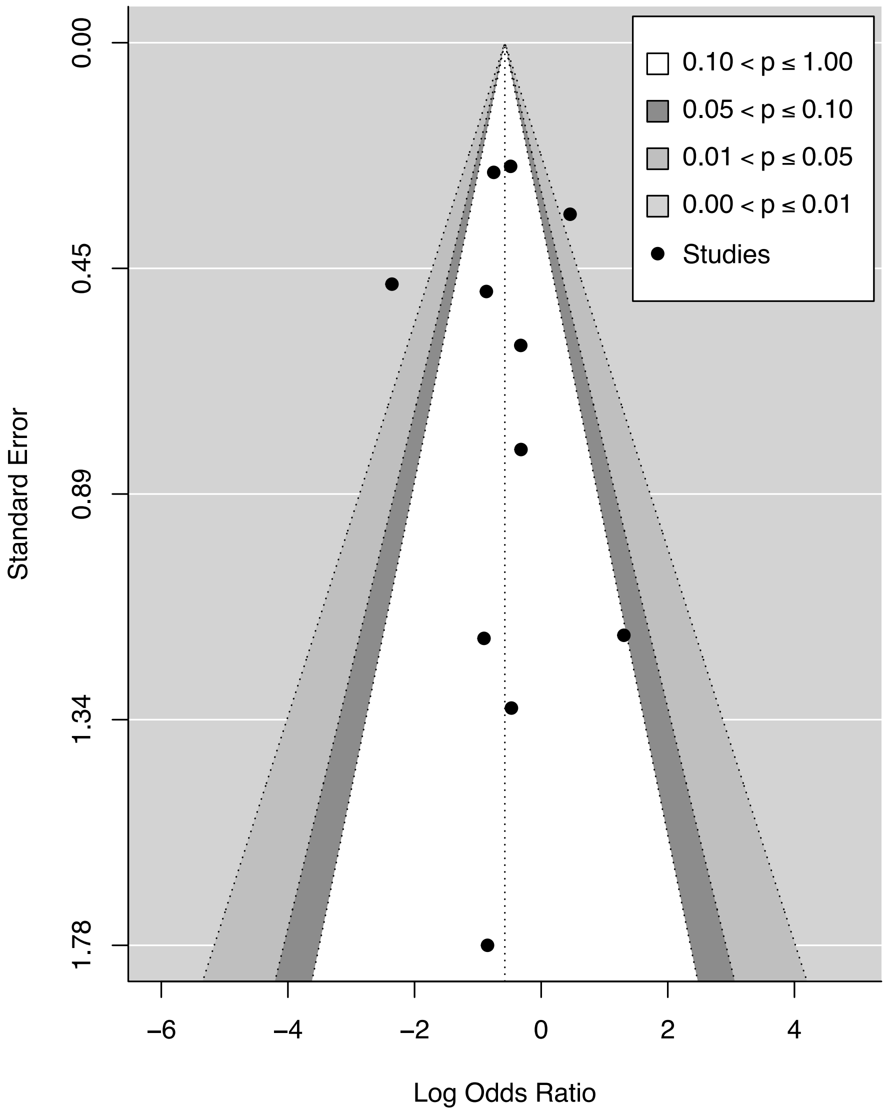

p-value from Egger test: 1·00

Figure 2.14.1.3: Contour-enhanced funnel plot for LA vs CT

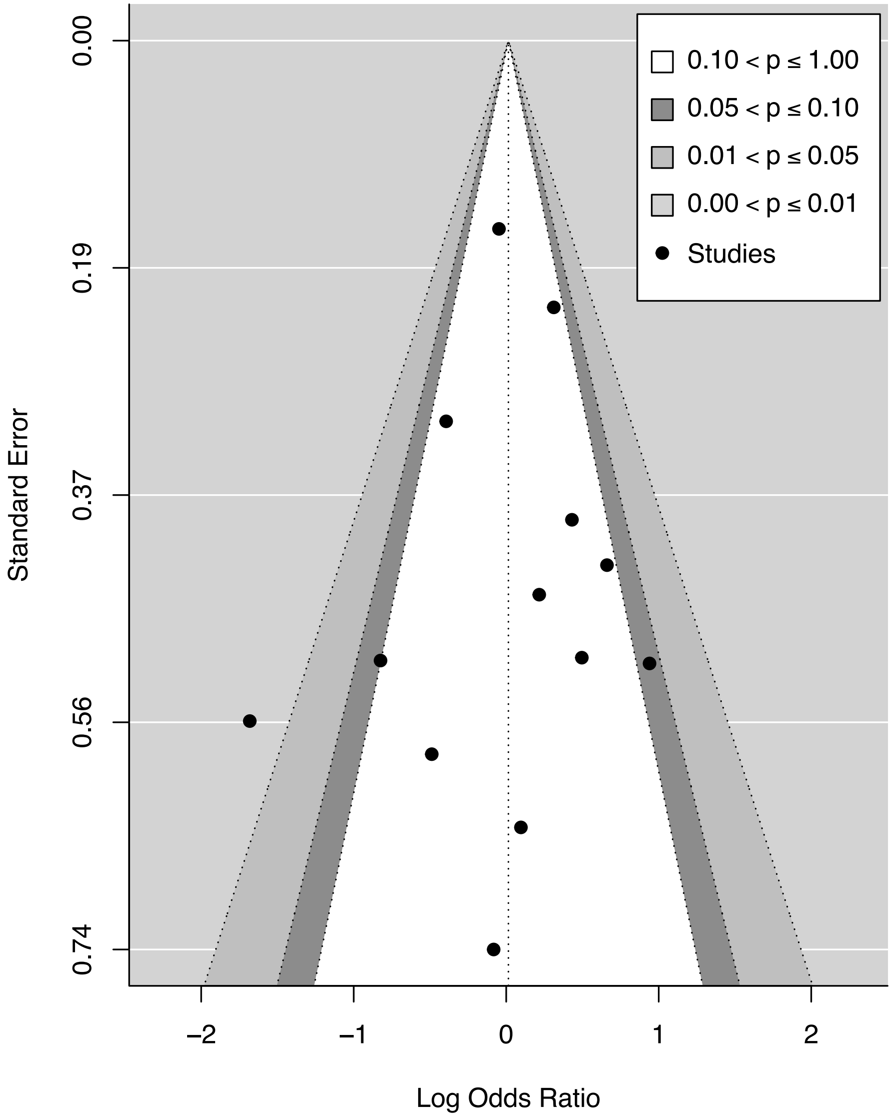

p-value from Egger test: 0·79

Table 2.14.1.2: Assessing for small-study effects using the ROB-MEN tool

NMA estimate	Percentage of contribution of evidence from pairwise comparisons with suspected bias: Favouring first treatment	Percentage of contribution of evidence from pairwise comparisons with suspected bias: Favouring second treatment	Evaluation of contribution from evidence with suspected bias	Bias assessment for indirect evidence	NMA treatment effect	Network meta-regression treatment effect at the smallest observed variance	Evaluation of small-study effects	Overall RoB
Mixed/only direct								
CC–CKC	0%	0%	No substantial contribution from bias	-	1·31 (0·78–2·20)	1·21 (0·62–2·38)	No evidence of small-study effects	Low risk
CC–CT	0%	0%	No substantial contribution from bias	-	0·44 (0·26–0·75)	0·49 (0·25–0·96)	No evidence of small-study effects	Low risk
CC–LA	0%	0%	No substantial contribution from bias	-	0·49 (0·29–0·82)	0·45 (0·24–0·88)	No evidence of small-study effects	Low risk
CC–LLETZ	0%	0%	No substantial contribution from bias	-	0·79 (0·48–1·27)	0·78 (0·43–1·44)	No evidence of small-study effects	Low risk
CC–RD	0%	0%	No substantial contribution from bias	-	0·50 (0·24–1·04)	0·47 (0·19–1·24)	No evidence of small-study effects	Low risk
CKC–CT	0%	0%	No substantial contribution from bias	-	0·34 (0·23–0·50)	0·41 (0·24–0·69)	No evidence of small-study effects	Low risk
CKC–LA	0%	0%	No substantial contribution from bias	-	0·37 (0·26–0·54)	0·38 (0·23–0·62)	No evidence of small-study effects	Low risk
CKC–LC	0%	0%	No substantial contribution from bias	-	1·07 (0·74–1·55)	1·14 (0·69–1·85)	No evidence of small-study effects	Low risk
CKC–LLETZ	0%	0%	No substantial contribution from bias	-	0·60 (0·46–0·78)	0·65 (0·45–0·93)	No evidence of small-study effects	Low risk
CKC–RD	0%	0%	No substantial contribution from bias	-	0·38 (0·21–0·71)	0·39 (0·19–0·85)	No evidence of small-study effects	Low risk
CT–LA	0%	0%	No substantial contribution from bias	-	1·10 (0·83–1·47)	0·93 (0·63–1·37)	No evidence of small-study effects	Low risk
CT–LLETZ	0%	0%	No substantial contribution from bias	-	1·77 (1·24–2·55)	1·59 (1·02–2·49)	No evidence of small-study effects	Low risk
CT–RD	0%	0%	No substantial contribution from bias	-	1·13 (0·57–2·23)	0·96 (0·42–2·30)	No evidence of small-study effects	Low risk
LA–LC	0%	0%	No substantial contribution from bias	-	2·86 (1·95–4·24)	3·01 (1·80–4·97)	No evidence of small-study effects	Low risk

LA–LLETZ	0%	0%	No substantial contribution from bias	-	1·61 (1·17–2·19)	1·72 (1·15–2·50)	No evidence of small-study effects	Low risk
LA–RD	0%	0%	No substantial contribution from bias	-	1·03 (0·52–2·00)	1·04 (0·46–2·39)	No evidence of small-study effects	Low risk
LC–LLETZ	0%	0%	No substantial contribution from bias	-	0·56 (0·41–0·77)	0·57 (0·38–0·85)	No evidence of small-study effects	Low risk
**Only indirect**								
LLETZ–RD	0%	0%	No substantial contribution from bias	No bias detected	0·64 (0·33–1·22)	0·61 (0·28–1·35)	No evidence of small-study effects	Low risk
CC–LC	0%	0%	No substantial contribution from bias	No bias detected	1·40 (0·79–2·46)	1·37 (0·68–2·80)	No evidence of small-study effects	Low risk
CT–LC	0%	0%	No substantial contribution from bias	No bias detected	3·16 (2·04–4·88)	2·80 (1·58–4·92)	No evidence of small-study effects	Low risk
LC–RD	0%	0%	No substantial contribution from bias	No bias detected	0·36 (0·18–0·72)	0·34 (0·15–0·82)	No evidence of small-study effects	Low risk

Figure 2.14.1.4: Risk-of-bias contribution chart (percent stacked bar chart)

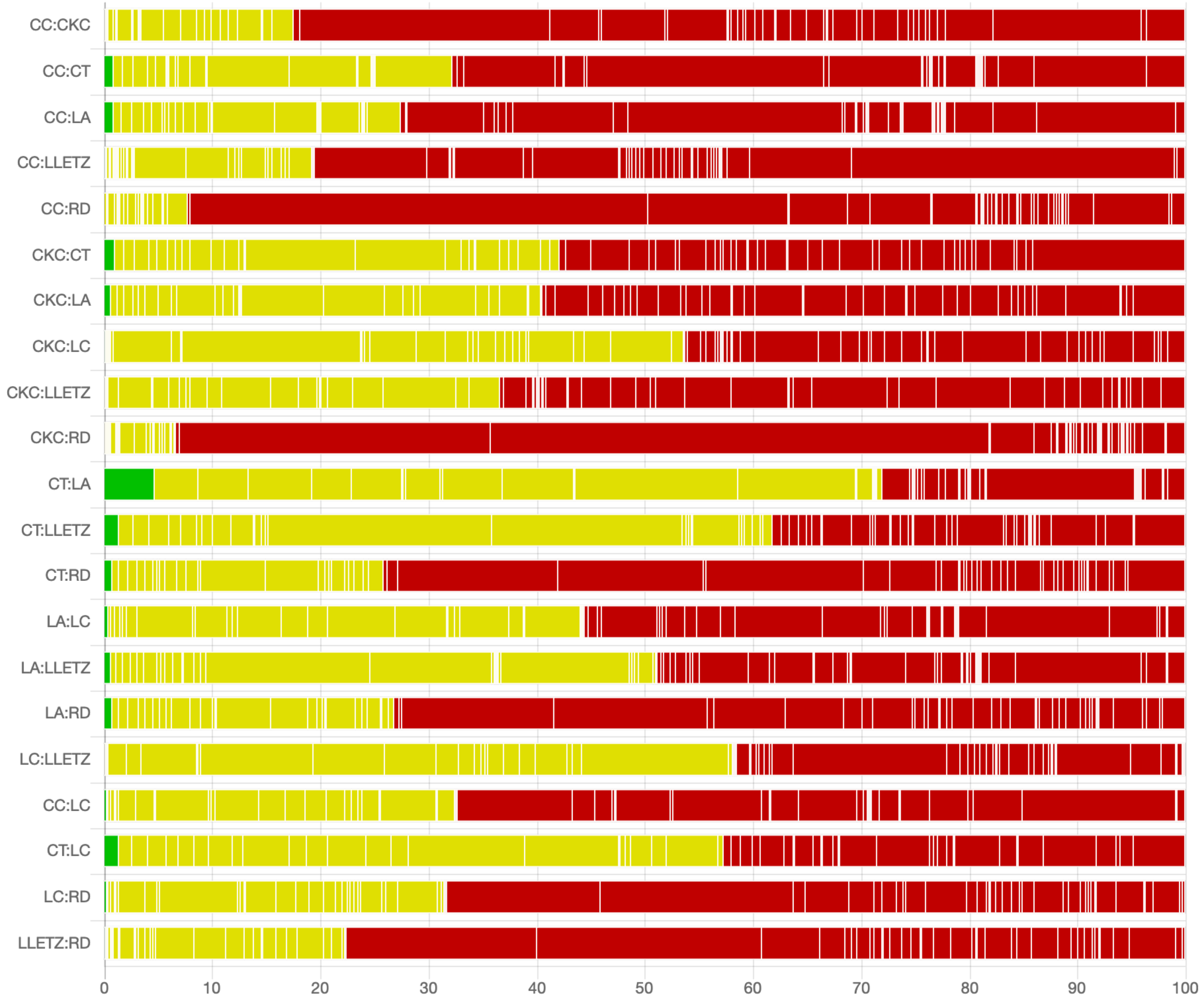

Percentage of studies at low RoB (green colour), moderate RoB (yellow colour), and high RoB (red colour) in each pairwise comparison

Table 2.14.1.3: Assessing the credibility of evidence using the CINeMA tool

Comparison	N studies	Within-study bias (Figure 2.14.1.4)	Reporting bias (Table 2.14.1.1)	Indirectness	Imprecision	Heterogeneity	Incoherence	Confidence rating
CKC–LC	6	Some concerns	Low risk	No concerns	Major concerns	No concerns	No concerns	Very low
CKC–RD	2	Major concerns	Low risk	No concerns	No concerns	No concerns	No concerns	Low
CKC–LA	2	Major concerns	Low risk	No concerns	No concerns	No concerns	No concerns	Low
CKC–CC	1	Major concerns	Low risk	No concerns	No concerns	Major concerns	No concerns	Very low
CKC–CT	2	Major concerns	Low risk	No concerns	No concerns	No concerns	No concerns	Low
CKC–LLETZ	27	Major concerns	Low risk	No concerns	No concerns	Some concerns	No concerns	Very low
LC–RD	0	Major concerns	Low risk	No concerns	No concerns	No concerns	No concerns	Low
LC–LA	5	Major concerns	Low risk	No concerns	No concerns	No concerns	Some concerns	Very low
LC–CC	0	Major concerns	Low risk	No concerns	No concerns	Some concerns	No concerns	Very low
LC–CT	0	Some concerns	Low risk	No concerns	No concerns	No concerns	No concerns	Moderate
LC–LLETZ	11	Some concerns	Low risk	No concerns	No concerns	Some concerns	No concerns	Low
RD–LA	1	Major concerns	Low risk	No concerns	Major concerns	No concerns	No concerns	Very low

RD–CC	1	Major concerns	Low risk	No concerns	Some concerns	Some concerns	No concerns	Very low
RD–CT	1	Major concerns	Low risk	No concerns	Major concerns	No concerns	No concerns	Very low
RD–LLETZ	0	Major concerns	Low risk	No concerns	Some concerns	Some concerns	No concerns	Very low
LA–CC	1	Major concerns	Low risk	No concerns	Some concerns	Some concerns	No concerns	Very low
LA–CT	13	Some concerns	Low risk	No concerns	Some concerns	Some concerns	Some concerns	Low
LA–LLETZ	8	Some concerns	Low risk	No concerns	No concerns	Some concerns	No concerns	Low
CC–CT	2	Major concerns	Low risk	No concerns	No concerns	Major concerns	No concerns	Very low
CC–LLETZ	2	Major concerns	Low risk	No concerns	Major concerns	No concerns	No concerns	Very low
CT–LLETZ	2	Some concerns	Low risk	No concerns	No concerns	Some concerns	No concerns	Low

2.14.2. Preterm Birth

Table 2.14.2.1: Assessing for within-study and across-study reporting bias using the ROB-MEN tool

	**N of studies in each comparison**		**Within-study assessment of bias**	**Across-study assessment of bias**	**Overall bias**
**Pairwise comparison**	Reporting this outcome (sample size)	Total identified in the systematic review (total sample size)	Evaluation of selective reporting within studies using signalling questions	Qualitative and quantitative assessment of publication bias	Overall judgement
**Group A: observed for this outcome**					
CKC–LC	2 (69)	7 (761)	No bias detected	No bias detected	No bias detected
CKC–LLETZ	12 (5531)	39 (10423)	No bias detected	No bias detected (Figure 2.14.2.1)	No bias detected
CKC–RD	1 (831)	3 (1734)	No bias detected	No bias detected	No bias detected
CKC–LA	1 (1076)	3 (1176)	No bias detected	No bias detected	No bias detected
CKC–CT	2 (93)	4 (885)	Suspected bias favouring CKC	No bias detected	Suspected bias favouring CKC
CKC–COLPO	2 (3659)	2 (3659)	No bias detected	No bias detected	No bias detected
LC–LLETZ	5 (570)	15 (4459)	No bias detected	No bias detected	No bias detected
LC–LA	4 (537)	8 (1246)	No bias detected	No bias detected	No bias detected
LC–COLPO	1 (531)	1 (531)	No bias detected	No bias detected	No bias detected
LLETZ–LA	3 (1710)	11 (3550)	No bias detected	No bias detected	No bias detected
LLETZ–CC	1 (116)	4 (2465)	No bias detected	No bias detected	No bias detected
LLETZ–CT	1 (111)	3 (771)	Suspected bias favouring LLETZ	No bias detected	Suspected bias favouring LLETZ
LLETZ–COLPO	10 (52544)	10 (52544)	No bias detected	No bias detected (Figure 2.14.2.2)	No bias detected

RD–LLETZ	1 (829)	1 (829)	No bias detected	No bias detected	No bias detected
RD–LA	1 (1765)	2 (1826)	No bias detected	No bias detected	No bias detected
RD–COLPO	1 (4244)	1 (4244)	No bias detected	No bias detected	No bias detected
LA–COLPO	2 (5138)	2 (5138)	No bias detected	No bias detected	No bias detected
**Group B:<br>observed for other outcomes**					
CKC–CC	0 (0)	1 (154)	No bias detected	No bias detected	No bias detected
RD–CC	0 (0)	1 (134)	No bias detected	No bias detected	No bias detected
RD–CT	0 (0)	1 (57)	No bias detected	No bias detected	No bias detected
LA–CC	0 (0)	1 (132)	No bias detected	No bias detected	No bias detected
LA–CT	0 (0)	13 (4873)	No bias detected	No bias detected	No bias detected
CC–CT	0 (0)	2 (300)	No bias detected	No bias detected	No bias detected
**Group C:<br>Unobserved**					
LC–CC	0 (0)	0 (0)	NA	No bias detected	No bias detected
LC–RD	0 (0)	0 (0)	NA	No bias detected	No bias detected
LC–CT	0 (0)	0 (0)	NA	No bias detected	No bias detected
CC–COLPO	0 (0)	0 (0)	NA	No bias detected	No bias detected
CT–COLPO	0 (0)	0 (0)	NA	No bias detected	No bias detected

Figure 2.14.2.1: Contour-enhanced funnel plot for CKC vs LLETZ

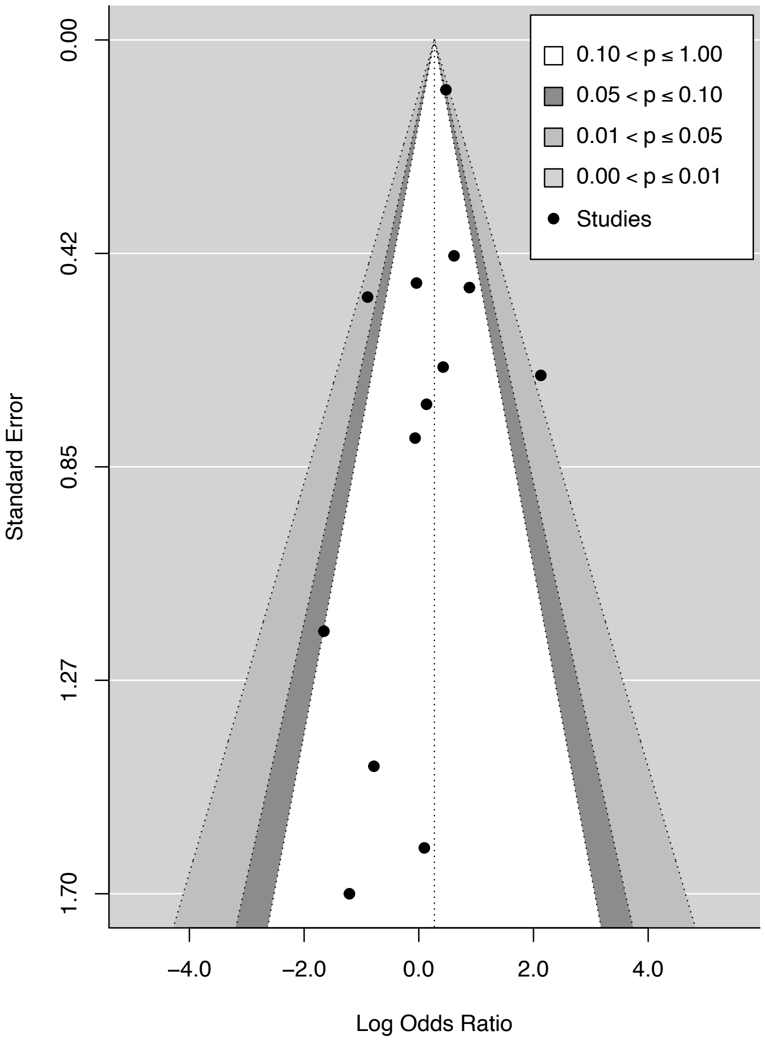

p-value from Egger test: 0·279

Figure 2.14.2.2: Contour-enhanced funnel plot for LLETZ vs COLPO

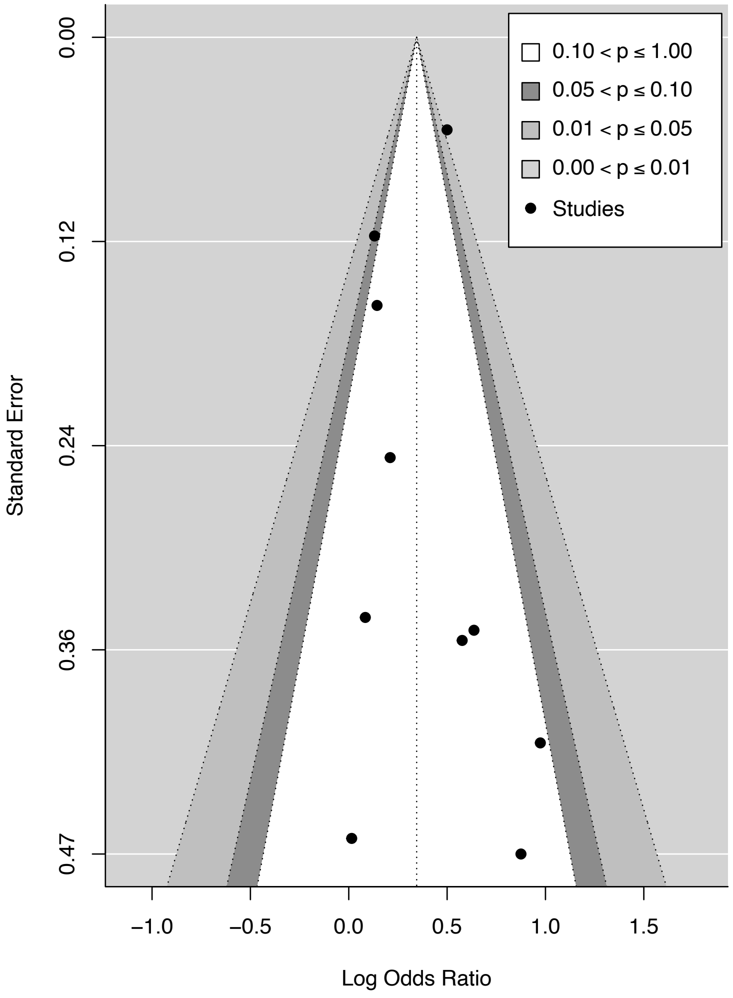

p-value from Egger test: 0·62

Table 2.14.2.2: Assessing for small-study effects using the ROB-MEN tool

NMA estimate	Percentage of contribution of evidence from pairwise comparisons with suspected bias: Favouring first treatment	Percentage of contribution of evidence from pairwise comparisons with suspected bias: Favouring second treatment	Evaluation of contribution from evidence with suspected bias	Bias assessment for indirect evidence	NMA treatment effect	Network meta-regression treatment effect at the smallest observed variance	Evaluation of small-study effects	Overall RoB
**Mixed/only direct**								
CC–LLETZ	0	0	No substantial contribution from bias	-	1·41 (0·16–12·74)	1·76 (0·00–4250·48)	No evidence of small-study effects	Low risk
CKC–COLPO	0	0	No substantial contribution from bias	-	1·85 (0·87–4·01)	2·24 (0·92–5·64)	No evidence of small-study effects	Low risk
CKC–CT	67%	0	Substantial contribution from bias favouring CKC	-	5·01 (0·76–48·44)	20·84 (0·06–184961·14)	No evidence of small-study effects	Some concerns
CKC–LA	0	0	No substantial contribution from bias	-	2·16 (0·77–5·98)	2·65 (0·77–9·01)	No evidence of small-study effects	Low risk
CKC–LC	0	0	No substantial contribution from bias	-	1·79 (0·66–5·13)	1·64 (0·40–6·97)	No evidence of small-study effects	Low risk
CKC–LLETZ	1%	1%	No substantial contribution from bias	-	1·70 (0·91–3·12)	2·27 (1·03–5·14)	No evidence of small-study effects	Low risk
CKC–RD	0	0	No substantial contribution from bias	-	1·27 (0·29–5·43)	1·47 (0·31–7·07)	No evidence of small-study effects	Low risk
COLPO–LA	0	0	No substantial contribution from bias	-	1·16 (0·44–3·00)	1·18 (0·39–3·42)	No evidence of small-study effects	Low risk
COLPO–LC	0	0	No substantial contribution from bias	-	0·97 (0·37–2·54)	0·73 (0·20–2·67)	No evidence of small-study effects	Low risk
COLPO–LLETZ	0	0	No substantial contribution from bias	-	0·92 (0·52–1·58)	1·01 (0·56–1·80)	No evidence of small-study effects	Low risk
COLPO–RD	0	0	No substantial contribution from bias	-	0·69 (0·16–2·85)	0·65 (0·15–2·92)	No evidence of small-study effects	Low risk
CT–LLETZ	0	36%	Substantial contribution from bias favouring LLETZ	-	0·34 (0·03–2·28)	0·11 (0·00–36·94)	No evidence of small-study effects	Some concerns
LA–LC	0	0	No substantial contribution from bias	-	0·83 (0·32–2·18)	0·62 (0·15–2·50)	No evidence of small-study effects	Low risk
LA–LLETZ	0	0	No substantial contribution from bias	-	0·79 (0·32–1·93)	0·85 (0·30–2·53)	No evidence of small-study effects	Low risk

LA–RD	0	0	No substantial contribution from bias	-	0·59 (0·13–2·57)	0·56 (0·12–2·74)	No evidence of small-study effects	Low risk
LC–LLETZ	0	0	No substantial contribution from bias	-	0·94 (0·39–2·27)	1·38 (0·39–4·70)	No evidence of small-study effects	Low risk
LLETZ–RD	0	0	No substantial contribution from bias	-	0·75 (0·18–3·05)	0·65 (0·15–2·89)	No evidence of small-study effects	Low risk
**Only indirect**								
CC–CKC	0	1%	No substantial contribution from bias	No bias detected	0·83 (0·09–8·46)	0·78 (0·00–2031·50)	No evidence of small-study effects	Low risk
CC–CT	0	0	No substantial contribution from bias	No bias detected	4·27 0·23–97·89)	15·76 (0·00–1485591·63)	No evidence of small-study effects	Low risk
CC–LA	0	0	No substantial contribution from bias	No bias detected	1·79 (0·17–19·47)	2·03 (0·00–5250·52)	No evidence of small-study effects	Low risk
CC–RD	0	0	No substantial contribution from bias	No bias detected	1·06 (0·08–14·44)	1·16 (0·00–3082·72)	No evidence of small-study effects	Low risk
CT–LA	0	0	No substantial contribution from bias	No bias detected	0·42 (0·04–3·50)	0·13 (0·00–47·24)	No evidence of small-study effects	Low risk
CT–RD	0	0	No substantial contribution from bias	No bias detected	0·25 (0·02–2·62)	0·07 (0·00–26·59)	No evidence of small-study effects	Low risk
CC–COLPO	0	0	No substantial contribution from bias	No bias detected	1·54 (0·17–15·00)	1·76 (0·00–4184·79)	No evidence of small-study effects	Low risk
CC–LC	0	0	No substantial contribution from bias	No bias detected	1·49 (0·14–16·39)	1·27 (0·00–3561·20)	No evidence of small-study effects	Low risk
COLPO–CT	0	0	No substantial contribution from bias	No bias detected	2·73 (0·37–27·22)	9·08 (0·03–78729·98)	No evidence of small-study effects	Low risk
CT–LC	0	0	No substantial contribution from bias	No bias detected	0·35 (0·03–2·88)	0·08 (0·00–28·65)	No evidence of small-study effects	Low risk
LC–RD	0	0	No substantial contribution from bias	No bias detected	0·71 (0·14–3·33)	0·89 (0·15–5·75)	No evidence of small-study effects	Low risk

Figure 2.14.2.3: Risk-of-bias contribution chart (percent stacked bar chart)

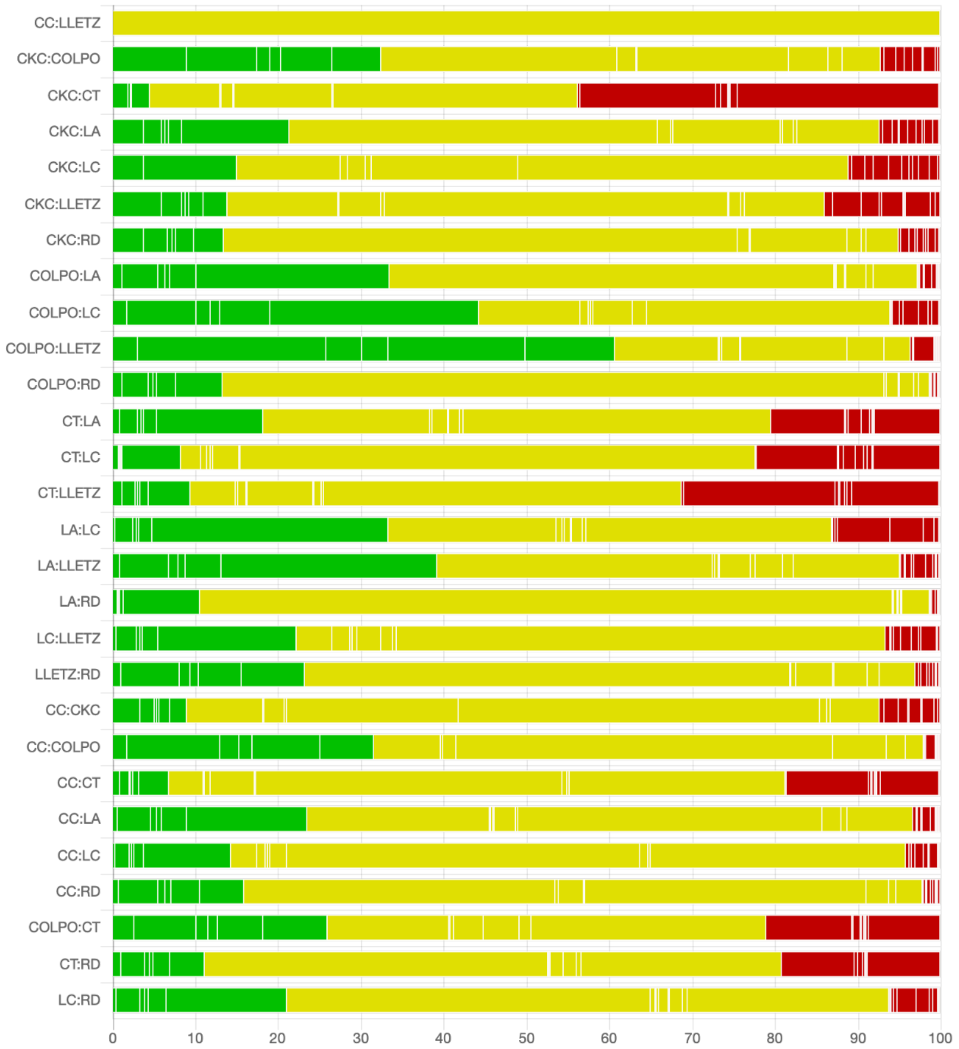

Percentage of studies at low RoB (green colour), moderate RoB (yellow colour), and high RoB (red colour) in each pairwise comparison

Table 2.14.2.3: Assessing the credibility of evidence using the CINeMA tool

Comparison	N studies	Within-study bias (Figure 2.14.2.3)	Reporting bias (Table 2.14.2.1)	Indirectness	Imprecision	Heterogeneity	Incoherence	Confidence rating
CKC–LC	3	Some concerns	Low risk	No concerns	Some concerns	Some concerns	No concerns	Low
CKC–LLETZ	13	Some concerns	Low risk	No concerns	No concerns	No concerns	No concerns	Moderate
CKC–RD	1	Some concerns	Low risk	No concerns	Major concerns	No concerns	No concerns	Very low
CKC–LA	2	Some concerns	Low risk	No concerns	No concerns	No concerns	No concerns	Moderate
CKC–CC	0	Major concerns	Low risk	No concerns	Major concerns	No concerns	No concerns	Very low
CKC–CT	3	Some concerns	Some concerns	No concerns	Major concerns	No concerns	No concerns	Very low
CKC–COLPO	2	Some concerns	Low risk	No concerns	No concerns	No concerns	No concerns	Moderate
LC–LLETZ	6	Some concerns	Low risk	No concerns	Some concerns	No concerns	No concerns	Low
LC–RD	0	Some concerns	Low risk	No concerns	Major concerns	No concerns	No concerns	Very low
LC–LA	5	Some concerns	Low risk	No concerns	No concerns	No concerns	No concerns	Moderate
LC–CC	0	Major concerns	Low risk	No concerns	Major concerns	No concerns	No concerns	Very low
LC–CT	1	Some concerns	Low risk	No concerns	Major concerns	No concerns	No concerns	Very low

LC–COLPO	1	Some concerns	Low risk	No concerns	No concerns	No concerns	No concerns	Moderate
LLETZ–RD	1	Some concerns	Low risk	No concerns	Some concerns	No concerns	No concerns	Low
LLETZ–LA	4	Some concerns	Low risk	No concerns	Some concerns	No concerns	No concerns	Low
LLETZ–CC	1	Major concerns	Low risk	No concerns	Major concerns	No concerns	No concerns	Very low
LLETZ–CT	2	Some concerns	Some concerns	No concerns	Major concerns	No concerns	No concerns	Very low
LLETZ–COLPO	10	No concerns	Low risk	No concerns	No concerns	Some concerns	No concerns	Moderate
RD–LA	1	Some concerns	Low risk	No concerns	No concerns	No concerns	No concerns	Moderate
RD–CC	0	Some concerns	Low risk	No concerns	Major concerns	No concerns	No concerns	Very low
RD–CT	0	Some concerns	Low risk	No concerns	Major concerns	No concerns	No concerns	Very low
RD–COLPO	1	Some concerns	Low risk	No concerns	No concerns	No concerns	No concerns	Moderate
LA–CC	0	Major concerns	Low risk	No concerns	Major concerns	No concerns	No concerns	Very low
LA–CT	1	Some concerns	Low risk	No concerns	Major concerns	No concerns	No concerns	Very low
LA–COLPO	2	Some concerns	Low risk	No concerns	Major concerns	No concerns	No concerns	Very low
CC–CT	0	Major concerns	Low risk	No concerns	Major concerns	No concerns	No concerns	Very low

CC–COLPO	0	Major concerns	Low risk	No concerns	Major concerns	No concerns	No concerns	Very low
CT–COLPO	0	Some concerns	Low risk	No concerns	Major concerns	No concerns	No concerns	Very low

3. Supplementary Discussion

Treatment for AIS

The data on AIS alone were limited. We found that CKC and LC were more effective in treating AIS than LLETZ, although there was some uncertainty in the estimates. This finding is in disagreement with another meta-analysis of women with AIS, which included 18 studies and reported similar rates of residual or recurrent disease between CKC and LLETZ.[209] However, this study included only observational studies and may have introduced bias by attempting to distinguish between recurrent and residual disease in only a fraction of women that required repeat treatment.

Additional Limitations

Data on radical diathermy, cold coagulation and cryotherapy were limited as these treatments are less commonly performed in high-income countries.

An analysis on the incidence of cervical cancer after treatment was not possible due to lack of studies reporting cancer incidence stratified per treatment technique in individual studies.

A subgroup analysis restricted to women treated for stage IA1 cervical cancer was not feasible due to small number of cases.

A subgroup analysis per type of TZ (type 1–3) was not possible as this was not reported in the studies due to the recent introduction of this terminology.

There was apparent imbalance in smoking distribution amongst across treatment comparisons, which could be explained by the overwhelming lack of data on smoking in most studies.

4. Abbreviations

AIS: adenocarcinoma in situ; **ASC-H**: atypical squamous cells — cannot exclude high-grade squamous intraepithelial neoplasia; **ASC-US**: atypical squamous cells of undetermined significance; **BMI**: body mass index; **CC**: cold coagulation; **CGIN:** cervical glandular intraepithelial neoplasia; **CI**: confidence interval; **CIN**: cervical intraepithelial neoplasia; **CKC**: cold knife conisation; **COLPO**: untreated colposcopy group; **CT**: cryotherapy; **DNA**: deoxyribonucleic acid; **ECC**: endocervical curettage; **FCBE**: Fischer cone biopsy excision; **f-u**: follow-up; **g**: gram(s); **GP**: general practitioner; **HIV**: human immunodeficiency virus; **HPV**: human papilloma virus; **HR**: hazard ratio; **hrHPV**: high-risk human papilloma virus; **HSIL**: high-grade squamous intraepithelial neoplasia; **IQR**: interquartile range; **ITT**: intention-to-treat; **LA**: laser ablation; **LC**: laser conisation; **LLETZ**: large loop excision of the transformation zone; **LSIL**: low-grade squamous intraepithelial neoplasia; **m**: month(s); **mcL**: microlitre(s); **mm**: millimetre(s); **mRNA**: messenger ribonucleic acid; **N**: Number; **NA**: not available; **NETZ**: needle excision of the transformation zone; **NMA**: network meta-analysis; **NRS**: non-randomised study; **OR**: odds ratio; **PB**: punch biopsy; **PI**: prediction interval; **(p)PROM**: (preterm) premature rupture of membranes; **RCT**: randomised controlled trial; **RD**: radical diathermy**;** **RoB:** risk of bias; **RR**: risk ratio; **ToC**: test of cure; **TZ**: transformation zone; **VaIN**: vaginal intraepithelial neoplasia; **vs**: versus; **w**: week(s); **y**: year(s)

5. References

1. Hutton B, Salanti G, Caldwell DM, et al. The PRISMA Extension Statement for Reporting of Systematic Reviews Incorporating Network Meta-analyses of Health Care Interventions: Checklist and Explanations. *Ann Intern Med* 2015; **162**(11): 777-84.
2. Salanti G. Indirect and mixed-treatment comparison, network, or multiple-treatments meta-analysis: many names, many benefits, many concerns for the next generation evidence synthesis tool. *Res Synth Methods* 2012; **3**(2): 80-97.
3. Athanasiou A, Veroniki AA, Efthimiou O, et al. Comparative efficacy and complication rates after local treatment for cervical intraepithelial neoplasia and stage 1a1 cervical cancer: protocol for a systematic review and network meta-analysis from the CIRCLE Group. *BMJ Open* 2019; **9**(8): e028008.
4. Athanasiou A, Veroniki AA, Efthimiou O, et al. Comparative fertility and pregnancy outcomes after local treatment for cervical intraepithelial neoplasia and stage 1a1 cervical cancer: protocol for a systematic review and network meta-analysis from the CIRCLE group. *BMJ Open* 2019; **9**(10): e028009.
5. Marujo AT, Correia L, Brito M, Paula T, Borrego J. ASC-H cytological result: clinical relevance and accuracy of colposcopy in predicting high-grade histological lesions-a 7-year experience of a single institution in Portugal. *J Am Soc Cytopathol* 2017; **6**(6): 248-53.
6. Kalliala I, Athanasiou A, Veroniki AA, et al. Incidence and mortality from cervical cancer and other malignancies after treatment of cervical intraepithelial neoplasia: a systematic review and meta-analysis of the literature. *Ann Oncol* 2020; **31**(2): 213-27.
7. Sand FL, Kjaer SK, Frederiksen K, Dehlendorff C. Risk of cervical intraepithelial neoplasia grade 2 or worse after conization in relation to HPV vaccination status. *Int J Cancer* 2020; **147**(3): 641-7.
8. Higgins JPT, Sterne JAC, Savović J, et al. A revised tool for assessing risk of bias in randomized trials. In: Chandler J, McKenzie J, Boutron I, Welch V, eds.: Cochrane Methods. Cochrane Database of Systematic Reviews; 2016.
9. Sterne JA, Hernan MA, Reeves BC, et al. ROBINS-I: a tool for assessing risk of bias in non-randomised studies of interventions. *BMJ* 2016; **355**: i4919.
10. Jackson D, Turner R. Power analysis for random-effects meta-analysis. *Res Synth Methods* 2017; **8**(3): 290-302.
11. Hartung J, Knapp G. A refined method for the meta-analysis of controlled clinical trials with binary outcome. *Stat Med* 2001; **20**(24): 3875-89.
12. Sidik K, Jonkman JN. A simple confidence interval for meta-analysis. *Stat Med* 2002; **21**(21): 3153-9.
13. Veroniki AA, Jackson D, Viechtbauer W, et al. Methods to estimate the between-study variance and its uncertainty in meta-analysis. *Res Synth Methods* 2016; **7**(1): 55-79.
14. Langan D, Higgins JPT, Jackson D, et al. A comparison of heterogeneity variance estimators in simulated random-effects meta-analyses. *Res Synth Methods* 2018.
15. Viechtbauer W. Confidence intervals for the amount of heterogeneity in meta-analysis. *Stat Med* 2007; **26**(1): 37-52.
16. Higgins JP, Thompson SG, Deeks JJ, Altman DG. Measuring inconsistency in meta-analyses. *BMJ* 2003; **327**(7414): 557-60.
17. Balduzzi S, Rücker G, Schwarzer G. How to perform a meta-analysis with R: a practical tutorial. *Evid Based Ment Health* 2019; **22**(4): 153-60.
18. R_Core_Team. R: A language and environment for statistical computing. R Foundation for Statistical Computing, Vienna, Austria. 2022. https://www.R-project.org (accessed April 2022.
19. Turner RM, Davey J, Clarke MJ, Thompson SG, Higgins JP. Predicting the extent of heterogeneity in meta-analysis, using empirical data from the Cochrane Database of Systematic Reviews. *Int J Epidemiol* 2012; **41**(3): 818-27.
20. Efthimiou O, Mavridis D, Debray TPA, et al. Combining randomized and non-randomized evidence in network meta-analysis. *Stat Med* 2017; **36**(8): 1210-26.
21. Schwarzer G, Chemaitelly H, Abu-Raddad LJ, Rücker G. Seriously misleading results using inverse of Freeman-Tukey double arcsine transformation in meta-analysis of single proportions. *Res Synth Methods* 2019.
22. Crippa A, Orsini N. Multivariate Dose-Response Meta-Analysis: The dosresmeta R Package. *Journal of Statistical Software, Code Snippets* 2016; **72** (1): 1-15.
23. Salanti G, Del Giovane C, Chaimani A, Caldwell DM, Higgins JP. Evaluating the quality of evidence from a network meta-analysis. *PLoS One* 2014; **9**(7): e99682.
24. Chiocchia V, Nikolakopoulou A, Higgins JPT, et al. ROB-MEN: a tool to assess risk of bias due to missing evidence in network meta-analysis. *BMC Med* 2021; **19**(1): 304.
25. Egger M, Davey Smith G, Schneider M, Minder C. Bias in meta-analysis detected by a simple, graphical test. *BMJ* 1997; **315**(7109): 629-34.
26. Viechtbauer W. Conducting Meta-Analyses in R with the metafor Package. *2010* 2010; **36**(3): 48.

27. White IR, Barrett JK, Jackson D, Higgins JP. Consistency and inconsistency in network meta-analysis: model estimation using multivariate meta-regression. *Res Synth Methods* 2012; **3**(2): 111-25.
28. Dias S, Welton NJ, Caldwell DM, Ades AE. Checking consistency in mixed treatment comparison meta-analysis. *Stat Med* 2010; **29**(7-8): 932-44.
29. Ang C, Mukhopadhyay A, Burnley C, et al. Histological recurrence and depth of loop treatment of the cervix in women of reproductive age: incomplete excision versus adverse pregnancy outcome: Loop treatment depth and CIN recurrence rate. *BJOG* 2011; **118**(6): 685-92.
30. Baggish MS. A comparison between laser excisional conization and laser vaporization for the treatment of cervical intraepithelial neoplasia. *Am J Obstet Gynecol* 1986; **155**(1): 39-44.
31. Baldauf JJ, Dreyfus M, Ritter J, Meyer P, Philippe E. Risk of cervical stenosis after large loop excision or laser conization. *Obstet Gynecol* 1996; **88**(6): 933-8.
32. Bornstein J, Yaakov Z, Pascal B, et al. Decision-making in the colposcopy clinic--a critical analysis. *Eur J Obstet Gynecol Reprod Biol* 1999; **85**(2): 219-24.
33. Byun JM, Jeong DH, Kim YN, et al. Persistent HPV-16 infection leads to recurrence of high-grade cervical intraepithelial neoplasia. *Medicine* 2018; **97**(51): e13606.
34. Cai DG, Zhao JY, Fan JB, Wan QY, Zhao XG. Comparison of clinical effects and pregnancy outcomes after treatment of cervical intraepithelial neoplasis by loop electrosurgical excision procedure or cold knife conization. *Journal of xi'an jiaotong university (medical sciences)* 2015; **36**(5): 677-81.
35. Dalrymple C, Valmadre S, Cook A, et al. Cold knife versus laser cone biopsy for adenocarcinoma in situ of the cervix - A comparison of management and outcome. *Int J Gynecol Cancer* 2008; **18**(1): 116-20.
36. Diakomanolis E, Rodolakis A, Dailianas S, Kalpaktsogiou K, Sakellaropoulos G, Aravantinos D. Long term results of CO2 laser treatment of low grade squamous intraepithelial lesions. *Cervix Low Female Genital Tract* 1995; **13**(4): 149-52.
37. Hansen J, Waibel J, Timme S, et al. Validity Parameters of the Human Papillomavirus Detection Test Hybrid Capture 2 With and Without Cytology After Laser Destruction and Large Loop Excision of the Transformation Zone Treatment of High-Grade Cervical Intraepithelial Neoplasia Lesions. *J Low Genit Tract Dis* 2017; **21**(4): 289-93.
38. Hellberg D, Nilsson S. 20-year experience of follow-up of the abnormal smear with colposcopy and histology and treatment by conization or cryosurgery. *Gynecol Oncol* 1990; **38**(2): 166-9.
39. Hussein IY, Galloway RK. Use of the cold coagulator in the treatment of cervical intra-epithelial neoplasia. *J Obstet Gynaecol* 1985; **6**(1): 62-4.
40. Husseinzadeh N, Rettig WL. Double-LEEP cone procedure as an alternative to cold-knife conization in management of cervical intraepithelial neoplasia. *J Low Genit Tract Dis* 2000; **4**(2): 72-5.
41. Ioffe OB, Brooks SE, De Rezende RB, Silverberg SG. Artifact in cervical LLETZ specimens: correlation with follow-up. *Int J Gynecol Pathol* 1999; **18**(2): 115-21.
42. Kietpeerakool C, Khunamornpong S, Srisomboon J, Kasunan A, Sribanditmongkol N, Siriaungkul S. Predictive value of negative cone margin status for risk of residual disease among women with cervical adenocarcinoma in situ. *Int J Gynaecol Obstet* 2012; **119**(3): 266-9.
43. Kiuchi K, Hasegawa K, Motegi E, Kosaka N, Udagawa Y, Fukasawa I. Complications of laser conization versus loop electrosurgical excision procedure in pre-And postmenopausal patients. *Eur J Gynaecol Oncol* 2016; **37**(6): 803-8.
44. Lele SB, Piver MS, Barlow JJ, McPhee ME. Treatment of cervical intraepithelial neoplasia. *N Y State J Med* 1984; **84**(5): 233-5.
45. Lu CH, Liu FS, Kuo CJ, Chang CC, Ho ES. Prediction of persistence or recurrence after conization for cervical intraepithelial neoplasia III. *Obstet Gynecol* 2006; **107**(4): 830-5.
46. Martel P, Bernard JD, Roumagnac M, Bonnet F, David JM. The treatment of severe dysplasia using CO2 laser; the respective indications for vaporisation and for conisation. Experience derived from 84 cases. *Journal de gynecologie obstetrique ET biologie de la reproduction* 1992; **21**(1): 23-9.
47. Murta EF, Conti R, Rodovalho J, Barcelos AC, Adad SJ, de Souza H. Outcome after treatment of high-grade squamous intraepithelial lesions: relation between colposcopically directed biopsy, conization and cervical loop excision. *Eur J Gynaecol Oncol* 2004; **25**(5): 587-90.
48. Omnes S, Morice P, Camatte S, et al. Modalities and limits of conservative treatment of adenocarcinoma in situ of the uterine cervix: analysis of nine cases and review of the literature. *Gynecol Obstet Fertil* 2003; **31**(11): 912-9.
49. Ostojic DV, Vrdoljak-Mozetic D, Stemberger-Papic S, Finderle A, Eminovic S. Cervical cytology and HPV test in follow-up after conisation or LLETZ. *Coll Antropol* 2010; **34**(1): 219-24.
50. Oyesanya OA, Amerasinghe C, Manning EA. A comparison between loop diathermy conization and cold-knife conization for management of cervical dysplasia associated with unsatisfactory colposcopy. *Gynecol Oncol* 1993; **50**(1): 84-8.

51. Papoutsis D, Underwood M, Parry-Smith W, Panikkar J. Comparison of cure rates in women treated with cold-coagulation versus LLETZ cervical treatment for CIN2-3 on pretreatment cervical punch biopsies: a retrospective cohort study. *Arch Gynecol Obstet* 2017; **295**(4): 979-86.
52. Park JY, Lee KH, Dong SM, Kang S, Park SY, Seo SS. The association of pre-conization high-risk HPV load and the persistence of HPV infection and persistence/recurrence of cervical intraepithelial neoplasia after conization. *Gynecol Oncol* 2008; **108**(3): 549-54.
53. Persad VL, Pierotic MA, Guijon FB. Management of cervical neoplasia: a 13-year experience with cryotherapy and laser. *J Low Genit Tract Dis* 2001; **5**(4): 199-203.
54. Serati M, Siesto G, Carollo S, et al. Risk factors for cervical intraepithelial neoplasia recurrence after conization: a 10-year study. *Eur J Obstet Gynecol Reprod Biol* 2012; **165**(1): 86-90.
55. Seshadri L, Cope I. The diagnosis, treatment and complications of cervical intraepithelial neoplasia--experience at the Royal Hospital for Women, Sydney, during the years 1972-1982. *Aust N Z J Obstet Gynaecol* 1985; **25**(3): 208-11.
56. Sideri M, Schettino F, Spolti N, Colleoni R, Negri L, Chiari S. Loop Diathermy to Replace Conization in the Conservative Treatment of In Situ Cancer of the Uterine Cervix. *J Gynecol Surg* 1994; **10**(4): 235-9.
57. Simmons JR, Anderson L, Hernandez E, Heller PB. Evaluating cervical neoplasia. LEEP as an alternative to cold knife conization. *J Reprod Med* 1998; **43**(12): 1007-13.
58. Tabor A, Berget A. Cold-knife and laser conization for cervical intraepithelial neoplasia. *Obstet Gynecol* 1990; **76**(4): 633-5.
59. Taylor JS, Panico V, Caputo T, et al. Clinical outcomes of patients with adenocarcinoma in situ of the cervix treated by conization. *Eur J Gynaecol Oncol* 2014; **35**(6): 641-5.
60. Urbaniak KJ, Widjaja A. Endocervical cell retrieval following excisional treatment of CIN: a comparison study between large loop excision of the transformation zone and laser cone excision. *Acta Obstet Gynecol Scand* 1996; **75**(1): 63-7.
61. Van Hanegem N, Barroilhet LM, Nucci MR, Bernstein M, Feldman S. Fertility-sparing treatment in younger women with adenocarcinoma in situ of the cervix. *Gynecol Oncol* 2012; **124**(1): 72-7.
62. Varawalla N, Patton P, Hodder R, Usherwood M. A comparison of cryocauterisation, laser vaporisation and large loop excision of the transformation zone for the treatment of cervical intraepithelial neoplasia. *J Obstet Gynaecol* 1996; **16**(6): 529-34.
63. Widrich T, Kennedy AW, Myers TM, Hart WR, Wirth S. Adenocarcinoma in situ of the uterine cervix: management and outcome. *Gynecol Oncol* 1996; **61**(3): 304-8.
64. Wolf JK, Levenback C, Malpica A, Morris M, Burke T, Mitchell MF. Adenocarcinoma in situ of the cervix: significance of cone biopsy margins. *Obstet Gynecol* 1996; **88**(1): 82-6.
65. Wright VC, Davies EM. The conservative management of cervical intraepithelial neoplasia: the use of cryosurgery and the carbon dioxide laser. *Br J Obstet Gynaecol* 1981; **88**(6): 663-8.
66. Zeng SY, Liang MR, Li LY, Wu YY. Comparison of the efficacy and complications of different surgical methods for cervical intraepithelial neoplasia. *Eur J Gynaecol Oncol* 2012; **33**(3): 257-60.
67. Küppers V, Eckardstein S, Koldovsky U, Somville T, Bender HG. [Treatment and after-care of HIV patients with cervix dysplasia]. *Geburtshilfe Frauenheilkd* 1994; **54**(11): 612-6.
68. Babkina N, Heller DS, Goldsmith LT, Houck KL. Cervical conization for cervical intraepithelial neoplasia (CIN) 2 and 3 in HIV-positive women: a case-control study. *J Low Genit Tract Dis* 2015; **19**(2): 110-4.
69. Gallwas J, Ditsch N, Hillemanns P, Friese K, Thaler C, Dannecker C. The significance of HPV in the follow-up period after treatment for CIN. *Eur J Gynaecol Oncol* 2010; **31**(1): 27-30.
70. Bogani G, V DID, Sopracordevole F, et al. Recurrence rate after loop electrosurgical excision procedure (LEEP) and laser Conization: A 5-year follow-up study. *Gynecol Oncol* 2020; **159**(3): 636-41.
71. Lara-Peñaranda R, Rodríguez-López PM, Plitt-Stevens J, Ortiz-González A, Remezal-Solano M, Martínez-Cendán JP. Does the trend toward less deep excisions in LLETZ to minimize obstetric risk lead to less favorable oncological outcomes? *Int J Gynaecol Obstet* 2020; **148**(3): 316-24.
72. Sun X, Lei H, Xie X, Ruan G, An J, Sun P. Risk Factors for Residual Disease in Hysterectomy Specimens After Conization in Post-Menopausal Patients with Cervical Intraepithelial Neoplasia Grade 3. *Int J Gen Med* 2020; **13**: 1067-74.
73. Zang L, Huang J, Zhu J, Hu Y. Risk factors associated with the persistence of human papillomavirus after cervical excision in patients with high-grade squamous intra-epithelial neoplasia. *Eur J Obstet Gynecol Reprod Biol* 2021; **266**: 175-81.
74. Armstrong GM, Ragupathy K. Test of cure and beyond: superiority of thermal ablation over LLETZ in the treatment of high-grade CIN. *Arch Gynecol Obstet* 2022.
75. Ferenczy A. Comparison of cryo- and carbon dioxide laser therapy for cervical intraepithelial neoplasia. *Obstet Gynecol* 1985; **66**(6): 793-8.

76. González-Bosquet J, Centeno C, Bermejo B, et al. Treatment of cervical intraepithelial neoplasia in a prospective study comparing large-loop excision of the transformation zone, laser vaporization, and knife cone biopsy. *J Low Genit Tract Dis* 1997; **1**(4): 229-33.
77. Guijon F, Paraskevas M, McNicol P. Human papillomavirus infection and the size and grade of cervical intraepithelial neoplastic lesions associated with failure of therapy. *Int J Gynaecol Obstet* 1993; **42**(2): 137-42.
78. Mariya T, Nishikawa A, Sogawa K, et al. Virological and cytological clearance in laser vaporization and conization for cervical intra-epithelial neoplasia grade 3. *J Obstet Gynaecol Res* 2016; **42**(12): 1808-13.
79. Wyse A, Seah WA, O'Neill J, Byrne P. The use of cold coagulation for the treatment of cervical intraepithelial neoplasia. *Ir Med J* 2017; **110**(5): 565.
80. Yliskoski M, Saarikoski S, Syrjanen K, Syrjanen S, Castren O. Cryotherapy and CO2-laser vaporization in the treatment of cervical and vaginal human papillomavirus (HPV) infections. *Acta Obstet Gynecol Scand* 1989; **68**(7): 619-25.
81. Zielinski GD, Rozendaal L, Voorhorst FJ, et al. HPV testing can reduce the number of follow-up visits in women treated for cervical intraepithelial neoplasia grade 3. *Gynecol Oncol* 2003; **91**(1): 67-73.
82. Alvarez RD, Helm CW, Edwards RP, et al. Prospective randomized trial of LLETZ versus laser ablation in patients with cervical intraepithelial neoplasia. *Gynecol Oncol* 1994; **52**(2): 175-9.
83. Berget A, Andreasson B, Bock JE. Laser and cryosurgery for cervical intraepithelial neoplasia. *Acta Obstet Gynecol Scand* 1991; **70**(3): 231-5.
84. Bostofte E, Berget A, Falck Larsen J, Pedersen H, Rank F. Conization by carbon dioxide laser or cold knife in the treatment of cervical intra-epithelial neoplasia. *Acta Obstet Gynecol Scand* 1986; **65**(3): 199-202.
85. Dey P, Gibbs A, Arnold DF, Saleh N, Hirsch PJ, Woodman CB. Loop diathermy excision compared with cervical laser vaporisation for the treatment of intraepithelial neoplasia: a randomised controlled trial. *BJOG* 2002; **109**(4): 381-5.
86. Duggan BD, Felix JC, Muderspach LI, et al. Cold-knife conization versus conization by the loop electrosurgical excision procedure: a randomized, prospective study. *Am J Obstet Gynecol* 1999; **180**(2 Pt 1): 276-82.
87. Giacalone PL, Laffargue F, Aligier N, Roger P, Combecal J, Daures JP. Randomized study comparing two techniques of conization: cold knife versus loop excision. *Gynecol Oncol* 1999; **75**(3): 356-60.
88. Goodman JDS, Sumner D. Patient acceptability of laser and cold coagulation therapy for pre-malignant disease of the uterine cervix. *Br J Obstet Gynaecol* 1991; **98**(11): 1168-71.
89. Gunasekera PC, Phipps JH, Lewis BV. Large loop excision of the transformation zone (LLETZ) compared to carbon dioxide treatment of CIN: a superior mode of treatment. *Br J Obstet Gynaecol* 1990; **97**(11): 995-8.
90. Helmerhorst TJM, Kwikkel HJ, Siregar E, Stolk JG. Conservative treatment of premalignant cervix anomalies: Prospective investigation. *Ned Tijdschr Geneeskd* 1985; **129**(41): 1965-7.
91. Jobson VW, Homesley HD. Comparison of cryosurgery and carbon dioxide laser ablation for treatment of cervical intraepithelial neoplasia. *Colposcopy and Gynecologic Laser Surgery* 1984; **1**(3): 173-80.
92. Kirwan PH, Smith IR, Naftalin NJ. A study of cryosurgery and the CO2 laser in treatment of carcinoma in situ (CIN III) of the uterine cervix. *Gynecol Oncol* 1985; **22**(2): 195-200.
93. Kwikkel HJ, Helmerhorst TJ, Bezemer PD, Quaak MJ, Stolk JG. Laser or cryotherapy for cervical intraepithelial neoplasia: a randomized study to compare efficacy and side effects. *Gynecol Oncol* 1985; **22**(1): 23-31.
94. Mathevet P, Chemali E, Roy M, Dargent D. Long-term outcome of a randomized study comparing three techniques of conization: cold knife, laser, and LEEP. *Eur J Obstet Gynecol Reprod Biol* 2003; **106**(2): 214-8.
95. Mitchell MF, Tortolero-Luna G, Cook E, Whittaker L, Rhodes-Morris H, Silva E. A randomized clinical trial of cryotherapy, laser vaporization, and loop electrosurgical excision for treatment of squamous intraepithelial lesions of the cervix. *Obstet Gynecol* 1998; **92**(5): 737-44.
96. Need JA, O'Shea RT. Laser vaporization versus electrocoagulation diathermy in the treatment of cervical intraepithelial neoplasia: A prospective randomized trial. *Colposcopy Gynecol Laser Surg* 1988; **4**(1): 37-40.
97. O'Shea RT, Need JA, Pomery GJ. Cryotherapy versus electrocoagulation diathermy for cervical intraepithelial neoplasia- a prospective randomised trial. *Colposcopy and gynecologic laser therapy* 1986; **2**(3): 159-61.
98. Oyesanya OA, Amerasinghe CN, Manning EA. Outpatient excisional management of cervical intraepithelial neoplasia. A prospective, randomized comparison between loop diathermy excision and laser excisional conization. *Am J Obstet Gynecol* 1993; **168**(2): 485-8.
99. Partington CK, Turner MJ, Soutter WP, Griffiths M, Krausz T. Laser vaporization versus laser excision conization in the treatment of cervical intraepithelial neoplasia. *Obstet Gynecol* 1989; **73**(5 Pt 1): 775-9.

100. Santos C, Galdos R, Alvarez M, et al. One-session management of cervical intraepithelial neoplasia: a solution for developing countries. *Gynecol Oncol* 1996; **61**(1): 11-5.
101. Singh P, Loke KL, Hii JHC, et al. Cold coagulation versus cryotherapy for treatment of cervical intraepithelial neoplasia: Results of a prospective randomized trial. *Colposcopy Gynecol Laser Surg* 1988; **4**(4): 211-21.
102. Takac I, Gorisek B. Cold knife conization and loop excision for cervical intraepithelial neoplasia. *Tumori* 1999; **85**(4): 243-6.
103. Townsend DE, Richart RM. Cryotherapy and carbon dioxide laser management of cervical intraepithelial neoplasia: a controlled comparison. *Obstet Gynecol* 1983; **61**(1): 75-8.
104. Vejerslev LO, Schouenborg L, Sørensen F, et al. Loop diathermy or laser excisional conization for cervical intraepithelial neoplasia. *Acta Obstet Gynecol Scand* 1999; **78**(10): 900-5.
105. Smith JS, Sanusi B, Swarts A, et al. A randomized clinical trial comparing cervical dysplasia treatment with cryotherapy vs loop electrosurgical excision procedure in HIV-seropositive women from Johannesburg, South Africa. *Am J Obstet Gynecol* 2017; **217**(2): 183.e1-.e11.
106. Greene SA, De Vuyst H, John-Stewart GC, et al. Effect of Cryotherapy vs Loop Electrosurgical Excision Procedure on Cervical Disease Recurrence Among Women With HIV and High-Grade Cervical Lesions in Kenya: A Randomized Clinical Trial. *JAMA* 2019; **322**(16): 1570-9.
107. Duan L, Du H, Belinson JL, et al. Thermocoagulation versus cryotherapy for the treatment of cervical precancers. *J Obstet Gynaecol Res* 2021; **47**(1): 279-86.
108. Kocken M, Helmerhorst TJ, Berkhof J, et al. Risk of recurrent high-grade cervical intraepithelial neoplasia after successful treatment: a long-term multi-cohort study. *Lancet Oncol* 2011; **12**(5): 441-50.
109. Bjorge T, Skare GB, Bjorge L, Trope A, Lonnberg S. Adverse Pregnancy Outcomes After Treatment for Cervical Intraepithelial Neoplasia. *Obstet Gynecol* 2016; **128**(6): 1265-73.
110. Jones JM, Sweetnam P, Hibbard BM. The outcome of pregnancy after cone biopsy of the cervix: a case-control study. *Br J Obstet Gynaecol* 1979; **86**(12): 913-6.
111. Praest J. [Conization of the cervix of the uterus]. *Ugeskr Laeger* 1979; **51**: 3509-11.
112. Buller RE, Jones HW. Pregnancy following cervical conization. *Am J Obstet Gynecol* 1982; **142**(5): 506-12.
113. Hemmingsson E. Outcome of third trimester pregnancies after cryotherapy of the uterine cervix. *Br J Obstet Gynaecol* 1982; **89**(8): 675-7.
114. Larsson G, Grundsell H, Gullberg B, Svennerud S. Outcome of Pregnancy After Conization. *Acta Obstet Gynecol Scand* 1982; **61**(5): 461-6.
115. Ludviksson K, Sandström B. Outcome of pregnancy after cone biopsy — A case-control study. *Eur J Obstet Gynecol Reprod Biol* 1982; **14**(3): 135-42.
116. Moinian M, Andersch B. Does Cervix Conization Increase the Risk of Complications in Subsequent Pregnancies? *Acta Obstet Gynecol Scand* 1982; **61**(2): 101-3.
117. Anderson MC, Horwell DH, Broby Z. Outcome of Pregnancy after Laser Vaporization Conization. *J Gynecol Surg* 1984; **1**(1): 35-9.
118. Kristensen GB. The outcome of pregnancy and preterm delivery after conization of the cervix. *Arch Gynecol* 1985; **236**(3): 127-30.
119. Kuoppala T, Saarikoski S. Pregnancy and delivery after cone biopsy of the cervix. *Arch Gynecol* 1986; **237**(3): 149-54.
120. Saunders N, Fenton DW, Soutter WP, Brown VA, Sharp F, Abernethy F. A case controlled study of the outcome of pregnancy following laser vaporization cone of the cervix. In: Sharp F, Jordan JA, eds. 15th Study Group: Gynaecological Laser Surgery. Ithaca, New York: Perinatalogy Press; 1986.
121. Wakita K, Izumi T, Kunamoto H, Sasaki N, Shimada N. Pregnancy After Laser Therapy for the Treatment of Uterine Cervical Neoplasia. *J Clin Laser Med Surg* 1990; **8**(5): 71-6.
122. Kasum M, Kuvačić I. [Pregnancy outcome after conization]. *Jugosl Ginekol Perinatol* 1991; **31**(1-2): 31-4.
123. Loizzi P, Carriero C, Di Gesu A, Resta L, Nappi R. Rational use of cryosurgery and cold knife conization for treatment of cervical intraepithelial neoplasia. *Eur J Gynaecol Oncol* 1992; **13**(6): 507-13.
124. Blomfield PI, Buxton J, Dunn J, Luesley DM. Pregnancy outcome after large loop excision of the cervical transformation zone. *Am J Obstet Gynecol* 1993; **169**(3): 620-5.
125. Haffenden DK, Bigrigg A, Codling BW, Read MD. Pregnancy following large loop excision of the transformation zone. *Br J Obstet Gynaecol* 1993; **100**(11): 1059-60.
126. Hagen B, Skjeldestad FE. The outcome of pregnancy after CO2 laser conisation of the cervix. *Br J Obstet Gynaecol* 1993; **100**(8): 717-20.
127. Braet PG, Peel JM, Fenton DW. A case controlled study of the outcome of pregnancy following loop diathermy excision of the transformation zone. *J Obstet Gynaecol* 1994; **14**(2): 79-82.

128. Cruickshank ME, Flannelly G, Campbell DM, Kitchener HC. Fertility and pregnancy outcome following large loop excision of the cervical transformation zone. *Br J Obstet Gynaecol* 1995; **102**(6): 467-70.
129. Sagot P, Caroit Y, Winer N, Lopes P, Boog G. Obstetrical prognosis for carbon dioxide laser conisation of the uterine cervix. *Eur J Obstet Gynecol Reprod Biol* 1995; **58**(1): 53-8.
130. Spitzer M, Herman J, Krumholz BA, Lesser M. The fertility of women after cervical laser surgery. *Obstet Gynecol* 1995; **86**(4): 504-8.
131. Bekassy Z, Iosif CS. Laser "miniconisation' and the outcome of subsequent pregnancy. *Arch Gynecol Obstet* 1996; **258**(2): 75-9.
132. Raio L, Ghezzi F, Di Naro E, Gomez R, Luscher KP. Duration of pregnancy after carbon dioxide laser conization of the cervix: influence of cone height. *Obstet Gynecol* 1997; **90**(6): 978-82.
133. Andersen ES, Pedersen B, Boris J. Pregnancy Outcome After Combination Laser Conization: A Case-Control Study. *J Gynecol Surg* 1999; **15**(1): 7-12.
134. El-Bastawissi AY, Becker TM, Daling JR. Effect of Cervical Carcinoma In Situ and Its Management on Pregnancy Outcome. *Obstet Gynecol* 1999; **93**(2): 207-12.
135. van Rooijen M, Persson E. Pregnancy outcome after laser vaporization of the cervix. *Acta Obstet Gynecol Scand* 1999; **78**(4): 346-8.
136. Sadler L, Saftlas A, Wang W, Exeter M, Whittaker J, McCowan L. Treatment for cervical intraepithelial neoplasia and risk of preterm delivery. *JAMA* 2004; **291**(17): 2100-6.
137. Tan L, Pepra E, Haloob RK. The outcome of pregnancy after large loop excision of the transformation zone of the cervix. *J Obstet Gynaecol* 2004; **24**(1): 25-7.
138. Samson SL, Bentley JR, Fahey TJ, McKay DJ, Gill GH. The effect of loop electrosurgical excision procedure on future pregnancy outcome. *Obstet Gynecol* 2005; **105**(2): 325-32.
139. Klaritsch P, Reich O, Giuliani A, Tamussino K, Haas J, Winter R. Delivery outcome after cold-knife conization of the uterine cervix. *Gynecol Oncol* 2006; **103**(2): 604-7.
140. Bruinsma F, Lumley J, Tan J, Quinn M. Precancerous changes in the cervix and risk of subsequent preterm birth. *BJOG* 2007; **114**(1): 70-80.
141. Bull-Phelps SL, Garner EI, Walsh CS, Gehrig PA, Miller DS, Schorge JO. Fertility-sparing surgery in 101 women with adenocarcinoma in situ of the cervix. *Gynecol Oncol* 2007; **107**(2): 316-9.
142. Himes KP, Simhan HN. Time From Cervical Conization to Pregnancy and Preterm Birth. *Obstet Gynecol* 2007; **109**(2): 314-9.
143. Jakobsson M, Gissler M, Sainio S, Paavonen J, Tapper AM. Preterm delivery after surgical treatment for cervical intraepithelial neoplasia. *Obstet Gynecol* 2007; **109**(2 Pt 1): 309-13.
144. Albrechtsen S, Rasmussen S, Thoresen S, Irgens LM, Iversen OE. Pregnancy outcome in women before and after cervical conisation: population based cohort study. *BMJ* 2008; **337**: a1343.
145. Patrelli TS, Anfuso S, Vandi F, et al. Preterm delivery and premature rupture of membranes after conization in 80 women. Preliminary data. *Minerva Ginecol* 2008; **60**(4): 295-8.
146. Jakobsson M, Gissler M, Paavonen J, Tapper AM. Loop electrosurgical excision procedure and the risk for preterm birth. *Obstet Gynecol* 2009; **114**(3): 504-10.
147. Michelin MA, Merino LM, Franco CA, Murta EF. Pregnancy outcome after treatment of cervical intraepithelial neoplasia by the loop electrosurgical excision procedure and cold knife conization. *Clin Exp Obstet Gynecol* 2009; **36**(1): 17-9.
148. Noehr B, Jensen A, Frederiksen K, Tabor A, Kjaer SK. Depth of cervical cone removed by loop electrosurgical excision procedure and subsequent risk of spontaneous preterm delivery. *Obstet Gynecol* 2009; **114**(6): 1232-8.
149. Noehr B, Jensen A, Frederiksen K, Tabor A, Kjaer SK. Loop electrosurgical excision of the cervix and subsequent risk for spontaneous preterm delivery: a population-based study of singleton deliveries during a 9-year period. *Am J Obstet Gynecol* 2009; **201**(1): 33.e1-6.
150. Shanbhag S, Clark H, Timmaraju V, Bhattacharya S, Cruickshank M. Pregnancy Outcome After Treatment for Cervical Intraepithelial Neoplasia. *Obstet Gynecol* 2009; **114**(4): 727-35.
151. Zornoza-García V, Luengo-Tabernero A, Reyero-Álvarez MP, Salas-Valién S, González-García C. [Obstetrics results after conization. Leon Hospital. 1999–2007 period]. *Clin Invest Ginecol Obstet* 2009; **36**(1): 9-12.
152. Nam KH, Kwon JY, Kim Y-H, Park Y-W. Pregnancy outcome after cervical conization: risk factors for preterm delivery and the efficacy of prophylactic cerclage. *J Gynecol Oncol* 2010; **21**(4): 225-9.
153. Ortoft G, Henriksen TB, Hansen ES, Petersen LK. After conisation of the cervix, the perinatal mortality as a result of preterm delivery increases in subsequent pregnancy: One or two conisations and risk of preterm delivery. *BJOG* 2010; **117**(3): 258-67.
154. van de Vijver A, Poppe W, Verguts J, Arbyn M. Pregnancy outcome after cervical conisation: a retrospective cohort study in the Leuven University Hospital: Pregnancy outcome after conisation: retrospective study from Leuven. *BJOG* 2010; **117**(3): 268-73.

155. Werner CL, Lo JY, Heffernan T, Griffith WF, McIntire DD, Leveno KJ. Loop electrosurgical excision procedure and risk of preterm birth. *Obstet Gynecol* 2010; **115**(3): 605-8.
156. Andia D, Mozo de Rosales F, Villasante A, Rivero B, Diez J, Perez C. Pregnancy outcome in patients treated with cervical conization for cervical intraepithelial neoplasia. *Int J Gynaecol Obstet* 2011; **112**(3): 225-8.
157. Lima AF, Francisco C, Julio C, Paula T, Vitorino A, Borrego J. Obstetric outcomes after treatment for cervical intraepithelial neoplasia: six years of experience. *J Low Genit Tract Dis* 2011; **15**(4): 276-9.
158. Castanon A, Brocklehurst P, Evans H, et al. Risk of preterm birth after treatment for cervical intraepithelial neoplasia among women attending colposcopy in England: retrospective-prospective cohort study. *BMJ* 2012; **345**: e5174.
159. Reilly R, Paranjothy S, Beer H, Brooks CJ, Fielder HM, Lyons RA. Birth outcomes following treatment for precancerous changes to the cervix: a population-based record linkage study: Birth outcomes following colposcopy treatments. *BJOG* 2012; **119**(2): 236-44.
160. Van Hentenryck M, Noel JC, Simon P. Obstetric and neonatal outcome after surgical treatment of cervical dysplasia. *Eur J Obstet Gynecol Reprod Biol* 2012; **162**(1): 16-20.
161. Berretta R, Gizzo S, Dall'Asta A, et al. Risk of preterm delivery associated with prior treatment of cervical precancerous lesion according to the depth of the cone. *Dis Markers* 2013; **35**(6): 721-6.
162. Frey HA, Stout MJ, Odibo AO, et al. Risk of cesarean delivery after loop electrosurgical excision procedure. *Obstet Gynecol* 2013; **121**(1): 39-45.
163. Kitson SJ, Greig E, Michael E, Smith M. Predictive value of volume of cervical tissue removed during LLETZ on subsequent preterm delivery: a cohort study. *Eur J Obstet Gynecol Reprod Biol* 2014; **180**: 51-5.
164. Sozen H, Namazov A, Cakir S, Akdemir Y, Vatansever D, Karateke A. Pregnancy outcomes after cold knife conization related to excised cone dimensions. A retrospective cohort study. *J Reprod Med* 2014; **59**(1-2): 81-6.
165. Kirn V, Geiger P, Riedel C, et al. Cervical conisation and the risk of preterm delivery: a retrospective matched pair analysis of a German cohort. *Arch Gynecol Obstet* 2015; **291**(3): 599-603.
166. Martyn FM, McAuliffe FM, Beggan C, Downey P, Flannelly G, Wingfield MB. Excisional treatments of the cervix and effect on subsequent fertility: a retrospective cohort study. *Eur J Obstet Gynecol Reprod Biol* 2015; **185**: 114-20.
167. Miller ES, Sakowicz A, Grobman WA. The association between cervical dysplasia, a short cervix, and preterm birth. *Am J Obstet Gynecol* 2015; **213**(4): 543.e1-4.
168. Aleman JM, Arlien F, Tjalma WAA. The impact of conisation on pregnancy outcome. *Eur J Gynaecol Oncol* 2016; **37**(6): 786-91.
169. Anwar A, Igbenehi C, Lindow SW, Noor N, Musa S, Saha A. Pregnancy outcome after electrosurgical cervical cone biopsy using Fischer cone biopsy excisor. *J Matern Fetal Neonatal Med* 2016; **29**(3): 477-81.
170. Brie C, Turck M, Cheret A, Morello R, Benoist G, Dreyfus M. [Case-control study of obstetrical outcomes of conisation]. *J Gynecol Obstet Biol Reprod (Paris)* 2016; **45**(2): 192-7.
171. Jancar N, Mihevc Ponikvar B, Tomsic S. Cold-knife conisation and large loop excision of transformation zone significantly increase the risk for spontaneous preterm birth: a population-based cohort study. *Eur J Obstet Gynecol Reprod Biol* 2016; **203**: 245-9.
172. van Velthoven K, Poppe W, Verschuere H, Arbyn M. Pregnancy outcome after cervical conisation: a 2nd retrospective cohort study in the Leuven University Hospital. *Eur J Obstet Gynecol Reprod Biol* 2017; **216**: 224-31.
173. Weinmann S, Naleway A, Swamy G, et al. Pregnancy Outcomes after Treatment for Cervical Cancer Precursor Lesions: an Observational Study. *PLoS One* 2017; **12**(1): e0165276.
174. Zebitay AG, Gungor ES, Ilhan G, Cetin O, Dane C, Furtuna C. Cervical Conization and the Risk of Preterm Birth: A Population-Based Multicentric Trial of Turkish Cohort. *J Clin Diagn Res* 2017; **11**(3): QC21-4.
175. Leiman G, Harrison NA, Rubin A. Pregnancy following conization of the cervix: complications related to cone size. *Am J Obstet Gynecol* 1980; **136**(1): 14-8.
176. Liverani CA, Di Giuseppe J, Clemente N, et al. Length but not transverse diameter of the excision specimen for high-grade cervical intraepithelial neoplasia (CIN 2-3) is a predictor of pregnancy outcome. *Eur J Cancer Prev* 2016; **25**(5): 416-22.
177. Chevreau J, Mercuzot A, Foulon A, et al. Impact of Age at Conization on Obstetrical Outcome: a Case-Control Study. *J Low Genit Tract Dis* 2017; **21**(2): 97-101.
178. Khalid S, Dimitriou E, Conroy R, et al. The thickness and volume of LLETZ specimens can predict the relative risk of pregnancy-related morbidity. *BJOG* 2012; **119**(6): 685-91.
179. Papoutsis D, Underwood M, Parry-Smith W, Panikkar J. Early and late pregnancy outcomes in women treated with cold-coagulation versus LLETZ cervical treatment for cervical intraepithelial neoplasia; a retrospective cohort study. *Arch Gynecol Obstet* 2018; **297**(4): 1015-25.

180. Heinonen A, Gissler M, Paavonen J, Tapper AM, Jakobsson M. Risk of preterm birth in women with cervical intraepithelial neoplasia grade one: a population-based cohort study. *Acta Obstet Gynecol Scand* 2018; **97**(2): 135-41.
181. Zhang X, Tong J, Ma X, et al. Evaluation of cervical length and optimal timing for pregnancy after cervical conization in patients with cervical intraepithelial neoplasia: A retrospective study. *Medicine (Baltimore)* 2020; **99**(49): e23411.
182. Collins A, Motiwale T, Barney O, Dudbridge F, McParland PC, Moss EL. Impact of past obstetric history and cervical excision on preterm birth rate. *Acta Obstet Gynecol Scand* 2021; **100**(11): 1995-2002.
183. Mosseri J, Hocquemiller R, Mergui JL, Uzan C, Canlorbe G. Laser conization for cervical intraepithelial neoplasia: Effectiveness and obstetric outcomes. *J Gynecol Obstet Hum Reprod* 2022; **51**(4): 102341.
184. Panelli DM, Wood RL, Elias KM, et al. The Loop Electrosurgical Excision Procedure and Cone Conundrum: The Role of Cumulative Excised Depth in Predicting Preterm Birth. *AJP Rep* 2022; **12**(1): e41-e8.
185. Wittmaack A, Dudley D, Boyle A. Maternal History of Cervical Surgery and Preterm Delivery: A Retrospective Cohort Study. *J Womens Health (Larchmt)* 2019; **28**(11): 1538-42.
186. Loopik DL, van Drongelen J, Bekkers RLM, et al. Cervical intraepithelial neoplasia and the risk of spontaneous preterm birth: A Dutch population-based cohort study with 45,259 pregnancy outcomes. *PLoS Med* 2021; **18**(6): e1003665.
187. Nitahara K, Fujita Y, Tanaka D, Magarifuchi N, Taniguchi S, Shimamoto T. Laser vaporization of the cervix is associated with an increased risk of preterm birth and rapid labor progression in subsequent pregnancies. *Arch Gynecol Obstet* 2021; **304**(4): 895-902.
188. Wiik J, Kärrberg C, Nilsson S, Strander B, Jacobsson B, Sengpiel V. Associations between cervical intraepithelial neoplasia during pregnancy, previous excisional treatment, cone-length and preterm delivery: a register-based study from western Sweden. *BMC Med* 2022; **20**(1): 61.
189. Gunasekera PC, Lewis BV, Phipps JH. Obstetric outcome after cervical surgery for intra-epithelial neoplasia. *J Obstet Gynaecol* 1992; **12**(5): 291-3.
190. Crane JMG, Delaney T, Hutchens D. Transvaginal Ultrasonography in the Prediction of Preterm Birth After Treatment for Cervical Intraepithelial Neoplasia. *Obstet Gynecol* 2006; **107**(1): 37-44.
191. Fischer RL, Sveinbjornsson G, Hansen C. Cervical sonography in pregnant women with a prior cone biopsy or loop electrosurgical excision procedure. *Ultrasound Obstet Gynecol* 2010; **36**(5): 613-7.
192. Poon LCY, Savvas M, Zamblera D, Skyfta E, Nicolaides KH. Large loop excision of transformation zone and cervical length in the prediction of spontaneous preterm delivery: LLETZ and preterm delivery. *BJOG* 2012; **119**(6): 692-8.
193. Simoens C, Goffin F, Simon P, et al. Adverse obstetrical outcomes after treatment of precancerous cervical lesions: a Belgian multicentre study: Preterm delivery after treatment of CIN. *BJOG* 2012; **119**(10): 1247-55.
194. Frega A, Sesti F, De Sanctis L, et al. Pregnancy outcome after loop electrosurgical excision procedure for cervical intraepithelial neoplasia. *Int J Gynaecol Obstet* 2013; **122**(2): 145-9.
195. Guo HJ, Guo RX, Liu YL. Effects of loop electrosurgical excision procedure or cold knife conization on pregnancy outcomes. *Eur J Gynaecol Oncol* 2013; **34**(1): 79-82.
196. Castanon A, Landy R, Brocklehurst P, et al. Risk of preterm delivery with increasing depth of excision for cervical intraepithelial neoplasia in England: nested case-control study. *BMJ* 2014; **349**: g6223.
197. Liu Y, Qiu HF, Tang Y, Chen J, Lv J. Pregnancy outcome after the treatment of loop electrosurgical excision procedure or cold-knife conization for cervical intraepithelial neoplasia. *Gynecol Obstet Invest* 2014; **77**(4): 240-4.
198. Nøhr B, Tabor A, Frederiksen K, Kjaer SK. Loop electrosurgical excision of the cervix and the subsequent risk of preterm delivery. *Acta Obstet Gynecol Scand* 2007; **86**(5): 596-603.
199. Heinonen A, Gissler M, Riska A, Paavonen J, Tapper AM, Jakobsson M. Loop electrosurgical excision procedure and the risk for preterm delivery. *Obstet Gynecol* 2013; **121**(5): 1063-8.
200. Acharya G, Kjeldberg I, Hansen SM, Sorheim N, Jacobsen BK, Maltau JM. Pregnancy outcome after loop electrosurgical excision procedure for the management of cervical intraepithelial neoplasia. *Arch Gynecol Obstet* 2005; **272**(2): 109-12.
201. Sjoborg KD, Vistad I, Myhr SS, et al. Pregnancy outcome after cervical cone excision: a case-control study. *Acta Obstet Gynecol Scand* 2007; **86**(4): 423-8.
202. Castañon A, Landy R, Brocklehurst P, et al. Is the increased risk of preterm birth following excision for cervical intraepithelial neoplasia restricted to the first birth post treatment? *BJOG* 2015; **122**(9): 1191-9.
203. Lara-Peñaranda R, Rodríguez-López PM, Plitt-Stevens J, Gómez-Leal P, Remezal-Solano M, Martínez-Cendán JP. Is large loop excision of the transformation zone depth a risk factor for affected endocervical margins? *J Obstet Gynaecol Res* 2020; **46**(10): 2100-7.

204. Adam Y, van Gelderen CJ, de Bruyn G, McIntyre JA, Turton DA, Martinson NA. Predictors of persistent cytologic abnormalities after treatment of cervical intraepithelial neoplasia in Soweto, South Africa: a cohort study in a HIV high prevalence population. *BMC Cancer* 2008; **8**: 211.
205. Chung MH, De Vuyst H, Greene SA, et al. Human Papillomavirus Persistence and Association With Recurrent Cervical Intraepithelial Neoplasia After Cryotherapy vs Loop Electrosurgical Excision Procedure Among HIV-Positive Women: A Secondary Analysis of a Randomized Clinical Trial. *JAMA Oncol* 2021.
206. Soutter WP, de Barros Lopes A, Fletcher A, et al. Invasive cervical cancer after conservative therapy for cervical intraepithelial neoplasia. *Lancet* 1997; **349**(9057): 978-80.
207. Kalliala I, Nieminen P, Dyba T, Pukkala E, Anttila A. Cancer free survival after CIN treatment: comparisons of treatment methods and histology. *Gynecol Oncol* 2007; **105**(1): 228-33.
208. Silfverdal L, Kemetli L, Sparén P, et al. Risk of invasive cervical cancer in relation to clinical investigation and treatment after abnormal cytology: a population-based case-control study. *Int J Cancer* 2011; **129**(6): 1450-8.
209. Jiang Y, Chen C, Li L. Comparison of Cold-Knife Conization versus Loop Electrosurgical Excision for Cervical Adenocarcinoma In Situ (ACIS): A Systematic Review and Meta-Analysis. *PLoS One* 2017; **12**(1): e0170587-e.
